# Risks and benefits of face masks in children

**DOI:** 10.3389/fped.2026.1679586

**Published:** 2026-03-13

**Authors:** Kai Kisielinski, Claudia Steigleder-Schweiger, Susanne Wagner, Stephan Korupp, Stefan Hockertz, Oliver Hirsch

**Affiliations:** 1 Clinical Medicine (Surgery), Emergency Medicine and Social Medicine, Private Practice, Düsseldorf, Germany; 2 Department of Paediatrics, University Hospital of Salzburg, Paracelsus Medical University, Salzburg, Austria; 3 Non Clinical Expert, Veterinarian, Wagner MSL Management, Mahlow, Germany; 4 Emergency Medicine, Private Practice, Aachen, Germany; 5 Toxicology, Pharmacology, Immunology, TPI consult AG, Baar, Switzerland; 6 FOM University of Applied Sciences, Siegen, Germany

**Keywords:** adverse effects, children, health risk assessment, long-term adverse effects, masks, MIES, N95, risk

## Abstract

**Introduction:**

Children, a significant and vulnerable portion of the global population, are particularly susceptible to environmental factors.

**Methods:**

We conducted a systematic search and scoping review of 3,144 articles, including 107 publications from medical literature, to assess mask use in children during the 2020–2023 pandemic. We examined expected viral protection vs. scientific evidence and side effects, synthesizing findings with SWiM and GRADE frameworks for evidence certainty and the Cochrane adverse effects approach.

**Results:**

Masking children lacks ecological validity, with high-quality studies showing little real-world effectiveness against viruses. On the other hand, side effects can clearly be identified. Masks contain hazardous materials (carcinogens, heavy metals, organic compounds, and microplastic), impacting childreńs health by altering inhaled air (including elevated carbon dioxide) and causing many physical symptoms and bio-psychosocial issues (MIES, mask-induced exhaustion syndrome), akin to sick building syndrome. Toxicological assessments highlight risks to biology of the young. Evidence certainty is high for non-effectiveness, moderate for risks and side effects, and low to very low for viral protection or benefits in children.

**Conclusions:**

With a negligible COVID-19 mortality rate in children (0.0003%) and no evidence of child-to-child or school-based transmission, masks offered little benefit during the pandemic. The documented adverse effects—respiratory impairment, toxicity, and health risks—outweigh any justification for their mandatory use. An individual risk–benefit analysis is essential (individual medical advice), but this review suggests avoiding this intervention in children because of its numerous downsides and the lack of proven efficacy. It is the responsibility of political leaders to address our findings.

## Introduction

The issue of mask-wearing in children has not yet been addressed in a comprehensive, systematic, and holistic manner. In particular, potential benefits and harms have rarely been weighed against each other based on the totality of the scientific literature.

Children are more vulnerable to environmental hazards, such as chemicals, due to physiological differences and unique kinds of behavior, necessitating specific risk assessment methodologies (1).

During the SARS-CoV-2 pandemic (2020–2023), mask-wearing became routine for many children on a global scale in the hope of containing the virus (2–7), despite inadequate risk assessments. Children wore masks more and longer than adults. They wore them continuously for approximately 6–7 h daily at school, while many adults could often avoid all-day use. School mandates also lasted longer than those for most adult settings.

The World Health Organization's mask guidelines from 2021 noted that improper mask handling could increase virus transmission, emphasizing strict protocols (e.g., hand hygiene, proper storage) that children struggle to follow, potentially raising transmission risks without benefits (8). Frequent violations of mask guidelines even by experts, politicians, and health officials demonstrate how difficult it is for children to follow these guidelines (see U.S. Senate Hearing, 18 March 2021, time marks 0:34:47, 2:08:09, 2:12:26, 2:23:26, 2:26:33, 2:37:30) (9).

The WHO's 2020 guidance highlighted that mask benefits in children must be weighed against potential harms, including social and communication issues (10).

Experts warned that widespread mask mandates could disrupt social, cultural, and psychological interactions (11–14). And still many countries mandated masks for children in schools (15–17), even for those as young as 2, despite reduced COVID-19 severity in children (18, 19) and no high-quality evidence of mask efficacy (15, 16, 20). The American Academy of Pediatrics recommended masks during indoor sports (21), influenced by nudging and social pressure (4, 22). In the postpandemic period, some publications, possibly due to bias or conflicts of interest (23), advocate continued mask use for children in future pandemics, often overlooking evidence of limited efficacy and proven side effects (e.g., CO_2_ toxicity, and microplastic inhalation) (24–31), such as for air pollution (32).

A balanced, evidence-based perspective on masks as a non-pharmaceutical intervention in children is needed, prioritizing medical and ethical risk–benefit assessments (15, 16, 27–29, 33–37).

With 2.4 billion children worldwide (∼30% of the 8 billion population) (38, 39), their susceptibility to environmental factors underscores the importance of this issue (40, 41). Masks pose significant risks, including side effects, requiring careful analysis (27–29, 34–36). The first narrative overview on pediatric mask use was published in 2011 (42), followed by a 2021 mini-review with limited pediatric studies (43) and a 2024 narrative review without systematic rigor and in-depth clinical and medical assessment (44). Sparse holistic data exist on pediatric mask use, particularly in the postpandemic period. Therefore, we conducted a scoping review with a systematic search to evaluate reliable scientific data, update knowledge, and provide a preliminary risk–benefit assessment, guided by evidence-based medicine principles (36, 37).

## Methods

### Review type and scope

This scoping review (45) with a systematic search adheres to PRISMA guidelines (46) to comprehensively evaluate mask use in children, capturing both positive and negative effects. Following the Cochrane Handbook's adverse effects approach (47–49), we included all study types to identify rare or long-term effects, especially from non-randomized studies. The review also assesses mask effectiveness against viral infections, a primary rationale for their use during the pandemic. Combining scoping and systematic review elements, we analyzed content and methodological quality to provide evidence-based recommendations (45).

### Aim and objectives

Using the participants, intervention, comparisons, outcome (PICO) framework (50) (Supplementary A, Table A, detailing population, intervention, comparison, and outcomes), the review question is: “In children (P) wearing face masks (I) compared to not wearing them (C), what effects, benefits, and risks (O) are described in the literature, and how strong is the evidence?”

### Outcomes

Outcomes encompassed positive and negative effects of mask use in children, including effectiveness against hazards and social, psychological, and physical impacts in accordance with the WHO biopsychosocial model.

### Search strategy and literature screening

Broad search terms reflecting the PICO framework were used in a systematic PubMed/MEDLINE search (1957–15 June 2024) for studies on mask use in individuals under 18 (Supplementary A, Table B). Two researchers independently screened titles and abstracts, with discrepancies resolved through discussion and consensus under the guidance of a senior author. Inclusion criteria covered positive effects, physiology, psychology, sociology, physical symptoms, clinical conditions, toxicology, and efficacy against respiratory agents (Supplementary A, Table C). Excluded studies and reasons were documented. The extracted data included study design, methodology, outcomes, sample size, findings, sponsorship and conclusions.

### Synthesis of evidence and certainty assessment

A narrative synthesis, guided by the SWiM guideline (51), grouped studies thematically: mask effectiveness, positive effects, psychological and sociological effects, physical symptoms, physiometabolic and toxicological effects, and claims about masks (Supplementary A, Table D).

Evidence certainty (high, moderate, low, and very low) was assessed using a GRADE-equivalent framework (52), considering bias (funding, observer, and methodological) without strictly applying Cochrane RoB tools (53).

This hybrid approach ensures a thorough evaluation of adverse effects (47–49) (Supplementary A).

### Risk assessment

A worst-case scenario approach (36) assessed mask-related risks in children, emphasizing severe, plausible adverse outcomes in accordance with European Union (EU) and US regulatory frameworks [e.g., European Medicines Agency (EMA), FDA, and ISO 14971:2019].

The precautionary principle and GRADE guidelines supported uprating observational evidence for biologically plausible, consistent, or dose-dependent adverse effects (47, 52, 54–57). This prioritizes early detection of harms (prodromal symptoms, preclinical symptoms, and early warning signs) (58, 59), adhering to the “do no harm” principle, despite RCTs (Randomized Controlled Trials) often under-reporting safety data (60–64).

## Results

Of the 3,149 articles retrieved, 107 peer-reviewed papers were analyzed (PRISMA, Supplementary B; Supplementary Figure S1), comprising 18 reviews, 76 primary studies, and 13 communications. Data extracted into Supplementary B tables cover effectiveness (16 papers, Supplementary Table S1), positive effects (13, Supplementary Table S2), psychological/sociological effects (41, Supplementary Table S3), clinical effects (8, Supplementary Table S4), physiometabolic/toxicological effects (23, Supplementary Table S5), and unproven claims (6, Supplementary Table S6).

Evidence certainty (Supplementary A, Table E) shows medium-to-high certainty for mask non-effectiveness, low-to-very-low for effectiveness, moderate for negative effects, and very low for pandemic claims (Supplementary Tables E,F).

## Discussion

The results of the evaluation of the included 107 publications are discussed below [sections Evidence for (Non-) Effectiveness, Positive Effects, Psychological and Sociological Effects, Physical Symptoms, Physiometabolic and Toxicological Effects, and Empirically/Experimentally Unproven Claims], based on the literature listed in the extraction tables (Supplementary B: Supplementary Tables S1–S6). In addition, we added an important chapter on microbiological mask contamination (Bacterial, Fungal, and Viral Contamination). Finally, the important topic of risk–benefit analysis (Risk Benefit Analysis and Risk-Assessment) is discussed, which can serve as a basis for decisions on mask obligations in children. This section also contains the summary of the scientific findings on masks, which are compared with our findings and discussed together, as well as a final risk assessment (section Risk Assessment of Masks and Children).

### Evidence for (non-)effectiveness

The effectiveness of masks in children against viral infections such as SARS-CoV-2 remains a subject of debate. Studies supporting mask use show low to very low certainty, while those refuting it demonstrate medium to high certainty (Supplementary A: Tables E,F; Supplementary B: Table S1).

#### Evidence for effectiveness

A cross-sectional study in kindergartens in Berlin in September 2021 found that neglect of facemask use was associated with a higher likelihood of previous SARS-CoV-2 infection (12.5% undiagnosed infections prevalence; 68.4% non-maskers) (65), reliability being limited by self-reporting and context. A cohort study found FFP2 masks delayed classroom SARS-CoV-2 infections (66), but not in the long term. A cross-sectional study in Somalia found that children not wearing facemasks had higher COVID-19 seroprevalence, though no separation of measures was established, and 46.9% of positive children were asymptomatic (67). A commentary noted that non-pharmaceutical interventions, including masks and reduced exposure, increased susceptibility to respiratory viruses in young children, with mask effectiveness assumptions based on modeling requiring further critical scrutiny (68). A systematic review suggested that mask mandates may reduce school transmission (69), yet methodological flaws weaken this low-certainty evidence. Another review of 14 studies linked masks to lower COVID-19 incidence (70), but credibility is reduced by bias and non-randomization.

#### Evidence for non-effectiveness

Limited efficacy is illustrated by observational data indicating poor mask compliance in children (e.g., 31.9% correct usage unsupervised) (71, 72). A systematic review of 22 studies found no high-quality evidence for mask benefits against SARS-CoV-2 (24). An editorial highlighted the lack of RCTs and potential promask publication bias (73): Children have mild symptoms, do not drive pandemic mortality, and masks distracting from better measures.

Large studies, including one with more than 1 million children, found no consistent link between mask mandates and case rates, let alone higher infection rates or a higher transmission indicator (25, 74, 75). A Cochrane systematic review based on a sound meta-analysis of only randomized trials reported no clear viral infection reduction in children (26). Studies showing no effect had stronger evidence (moderate to high certainty) than those favoring masks (low to very low), with publication bias possibly exaggerating benefits (76, 77).

### Positive effects

Positive effects of masks in children are suggested, though evidence certainty is very low (Supplementary A: Tables E,F, Supplementary B: Table S2). For COVID-19, evidence remains weak and unproven (35). In a review, it was shown that N95 respiratory masks could reduce wildfire smoke particle exposure by approximately 80% and surgical masks by approximately 20% in children, though fit issues and adverse effects limit benefits to short durations (78). Studies on non-pharmaceutical interventions (NPIs) —e.g., masks, hand hygiene, social distancing with no clear separation of measures— reported reduced infectious disease rates, including invasive pneumococcal disease (79) and notifiable infectious diseases except COVID-19, with a reduction of up to 82.1% in children aged 0–14 (80).

A 2011 trial showed that early NPI implementation, including masks, lowered influenza risk in households, though confounders were not separated (81).

A related study found that children tolerated masks in a similar way that adults did (82).

A trial reported that masks reduced hand-to-mucosa contact in children, but increased non-mucosa touching neutralized overall hand-to-face contact changes (83).

Modeling suggested that masks reduced viral transmission 8-fold in classrooms, though empirical real-world data are missing (84).

Observational data linked mask mandates to a 69% drop in aerosol concentrations and lower transmission in schools, but hard endpoints (e.g., infections) were absent. NPIs seem to have reduced disease rates, but since masks were not separated from other measures, it led to confounding specific effects (85–89).

Surveys associated frequent mask use with reduced self-reported psychological distress, though causality remains unclear (90) and the correlation described can have other reasons.

Positive effects are often hypothetical, confounded by other NPIs or limited to specific scenarios [wildfire smoke (78), or e.g., bacteria and tuberculosis control (91, 92)]. Most studies fail to isolate mask effects from measures like handwashing, undermining firm conclusions (79–82, 85, 87–89).

Altogether, evidence certainty remains low to very low (Supplementary A: Tables E,F).

### Psychological and sociological effects

Mask-wearing in children, driven by mandates and social pressure (4, 22, 32, 93), has clear adverse psychological and sociological effects, with moderate evidence certainty (Supplementary A: Tables E,F; Supplementary B: Table S3). While tolerance can be induced, even in children with autism (94–96), many observed children (96–100) reported discomfort or embarrassment (96, 99, 100), despite some viewing masks as useful (97, 98). There is no doubt about the negative effects of the pandemic NPIs, such as delays in communication, language and social development, as well as changes in the practices of care providers (101). Masks impair communication by reducing speech clarity (3–12 dB attenuation) (102–107) and obscuring facial expressions, hindering emotion recognition —especially positive emotions— critical for social and emotional development (108–128).

Masks caused difficulties in processing facial expressions, stress, anxiety, and learning challenges in children and teachers (129). Infants adapted to masks for word learning, relying on eyes when speakers were masked (130). Older students reported that masks hindered peer and teacher interactions (131). Masks impaired school performance by blocking visual cues and lip-reading (132), reducing speech perception and emotion recognition (133). Children responded slowlier and with more mistakes to masked speech (134). Teachers spoke more words but used fewer phonemes during COVID-19; children vocalized more in response (135). A trial showed slightly worse cognitive performance in mask-wearers, but claimed no significant impact, despite a flawed methodology (short observation and no appropriate control group) (136) and the fact of adaptive changes (137, 138). Studies on adults confirm cognitive decline under masks (139), with CO_2_ levels (1.41%–3.7%) impairing cognition (27, 140–154). Masks caused heat, breathing issues, and poor fit (81, 155, 156). Physician masks increased children's anxiety (157), and muffled speech led to comprehension errors (158).

### Physical symptoms

**Figure 1:**
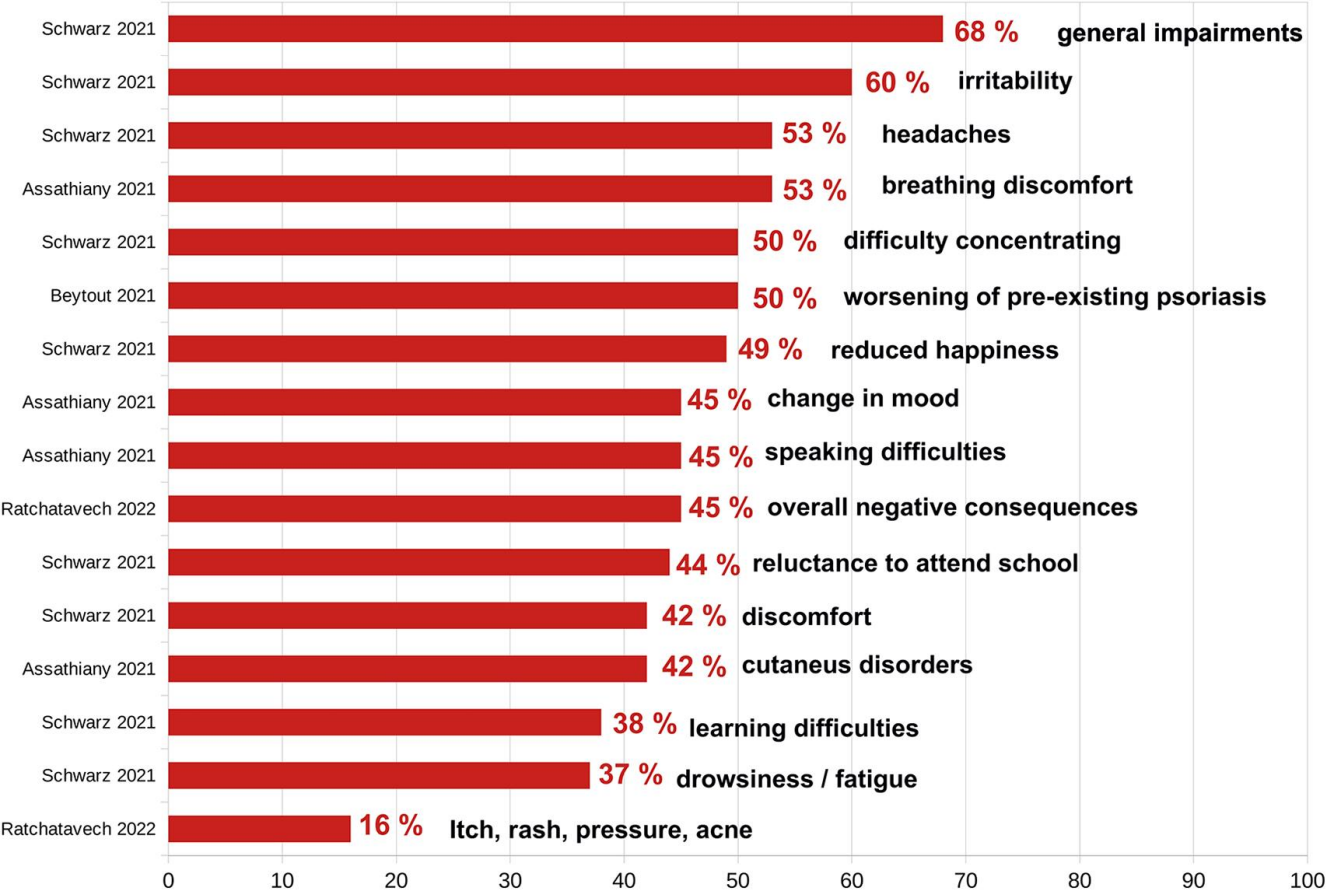
Frequency of selected symptoms in children wearing masks as determined in scientific studies (in percent). The results from cross sectional, online registry, and survey studies from the years 2021–2022 showing a very high number of adverse effects of face masks.

Mask-wearing in children causes clear adverse physical symptoms and clinical conditions, with moderate to high evidence certainty (Supplementary A: Table E, Supplementary B: Table S4). Surveys report that 44.9%–68% of children experiencing issues, including respiratory discomfort (28.1–33.9%), headaches (38.2%–53%), irritability (60%), difficulty concentrating (50%), and cutaneous effects (16.3–42.4%, e.g., itch, rash, and acne) (100, 159, 160). Psoriasis worsened in approximately 50% of affected children (161), and mask-related humidity increased facial pityriasis versicolor (162). Ocular injuries such as corneal abrasions occur due to loose adult masks (163). Prolonged mask use causes ear pressure injuries, potentially deforming underdeveloped auricular cartilage in preadolescents (164, 165). The Cochrane review notes additional underinvestigated harms (26). These symptoms conflict with the WHO health definition (166) (Figure 1), highlighting the urgent need for further research into mask-related physical impacts on children.

### Physiometabolic and toxicological effects

The literature shows clear, disturbing physiometabolic and toxicological mask effects on children (Supplementary B: Table S5), with predominantly moderate to high evidence certainty (Supplementary A: Table E).

Studies report increased breathing difficulties, elevated CO_2_ levels, anxiety, and safety issues like improper fit in children wearing masks (42, 43, 167).

Reports link sudden deaths during physical exertion with masks, especially in children with cardiac conditions (168). Short-term studies (1–15 min) found no significant changes in SpO_2_ or CO_2_ (169–171), but their brief duration limits medical validity. A study on athlete boys showed reduced performance and increased stress with masks (172), while another noted significant CO_2_ rises and O_2_ drops after 10 min, though interpreted as safe (173). Decreased breath isoprene during mask use reflects metabolic shifts due to hypoxia and hypercarbia (34, 174). In 110 children, surgical mask-wearing for 10 min significantly increased tricuspid regurgitation, pulmonary regurgitation, and pulmonary artery systolic pressure (175). In another study, however, surgical mask use showed no changes in respiratory parameters or signs of distress (176).

Both mask types, especially N95, decrease oxygenation and increase respiratory rate and CO_2_ levels, notably in overweight children (177–179).

**Figure 2:**
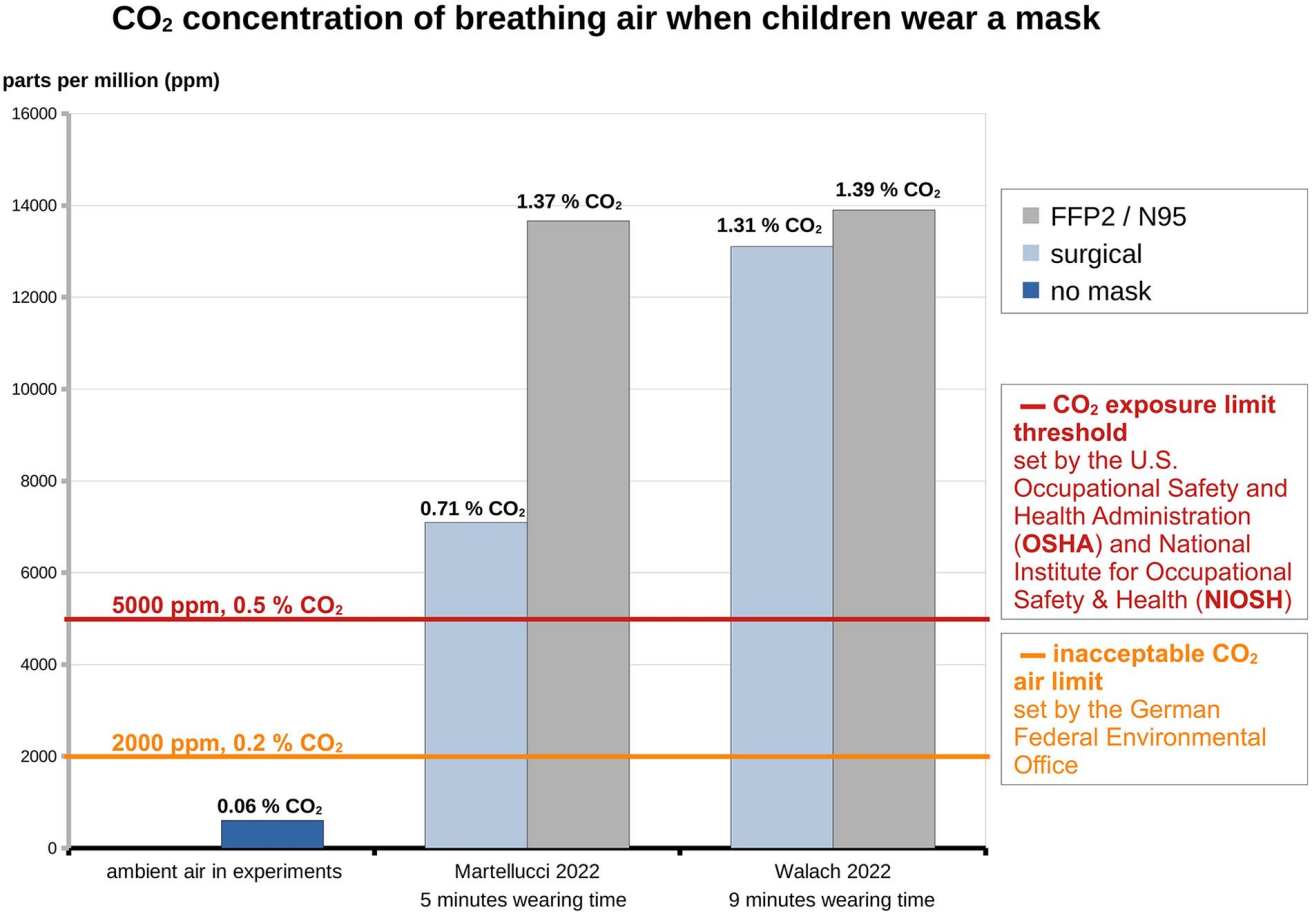
The bar charts display excess CO_2_ in children's breathing air when wearing masks (mean/median values), compared with ambient air (no mask condition as the average value of both studies). Only these studies reliably measured CO_2_ in the mask's breathing zone in children. Exceeding limit values raises concerns.

CO_2_ rises (180–182), exceeding safety limits in the mask breathing zone (1.3% in surgical and 1.39% in FFP2 masks, Figure 2), posing neurotoxicity and reproductive risks (27, 30, 31).

Even cotton masks reduce SpO_2_ (183).

**Figure 3:**
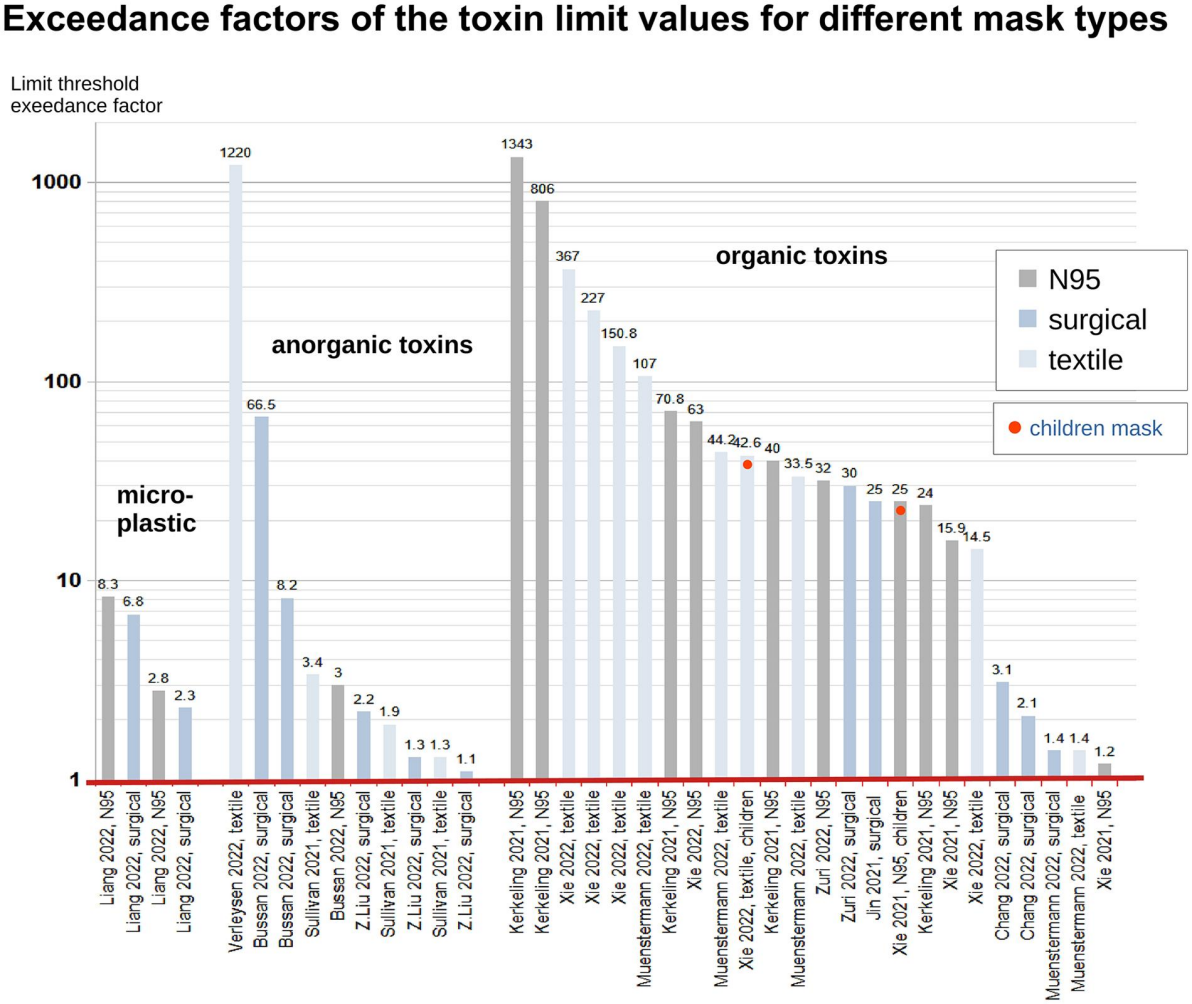
A graphical summary of studies showing that mask toxin content/release exceeds limits by up to 1,000 times. The logarithmic *Y*-axis reflects large exceedances. The red *X*-axis marks limit thresholds for microplastic, inorganic, and organic toxins, compared with WHO, EU, German, Oeko-Tex, and US EPA standards (28).

Toxicologically, masks release volatile compounds (e.g., toluene) and exceed safety limits for released microplastics and anorganic, and organic toxins (Figure 3) (28, 284, 285). These findings highlight physiological and toxicological concerns, underscoring the need for long-term research as opposed to short evaluations.

### Empirically/experimentally unproven claims

Early in the SARS-CoV-2 pandemic, unproven claims with low evidence certainty (Supplementary A: Tables E, F, Supplementary B: Table S6) promoted mask use in children to reduce transmission (186, 187), relying on weak meta-analyses, laboratory studies, or theoretical models lacking sound empirical and real-world data (7, 188–194), driven by political motives and pandemic hysteria (22, 76, 77, 195).

### Bacterial, fungal, and viral contamination

No peer-reviewed studies specifically address face mask contamination in children, although gray literature reports laboratory-confirmed cases (196). Adult studies and other research (197, 198) reveal that masks accumulate pathogens like *Aspergillus*, *Klebsiella*, *Pseudomonas*, *Staphylococcus*, and *Streptococcus (*198–213), with contamination levels being 310 times higher than German ventilation standards (VDI 6022) (198, 214).

Contamination rises with wearing time (197, 200–205, 207, 212, 215), particularly for N95/FFP2 masks (213). Prolonged use disrupts skin and respiratory microbiomes, increasing the risk of respiratory, skin, oral, and eye infections (197, 198, 201, 202, 212, 216–219). Masks trap and harbor microorganisms, enabling inhalation (220, 221) or spread via air streams (222–229), with the end result (via distribution) of potentially causing infections (227, 230–238). The polypropylene meshwork allows SARS-CoV-2 to persist infectious for up to 2 weeks (239, 240). For children, poor hygiene compliance may heighten risk. Further research is urgently required in this area.

### Risk–benefit analysis

The risk–benefit analysis must ensure that the risks of wearing masks in children are lower than they are if they do not wear them, requiring a toxicological risk assessment with a worst-case approach (36).

The WHO and United Nations Children's Fund (UNICEF) advocate the “do no harm” principle, prioritizing children's health and wellbeing (10). Physicians must align with the Hippocratic oath and the Geneva Declaration (1948, revised 2017), placing patient health and dignity first, even under pressure (241). Children's rights under the United Nations (UN) Convention on the Rights of the Child demand cautious decision-making, given the disproportionate disruptions that they faced during the pandemic (15, 16).

Comparing rigid mask policies (e.g., UK) with lenient ones (e.g., Sweden) reveals educational declines in stricter regions (242), with masks impeding learning (27, 31, 101, 102, 108, 110, 129, 160, 243, 244).

#### Unproven effectiveness of masks in children against viruses

Evidence for mask effectiveness in preventing viral transmission in children was sparse at the beginning of the pandemic (245) and remained weak, with the evidence being rated low to very low (15, 20, 25, 35, 66, 69, 71, 73–75). A postpandemic review found no scientific support for masking children against SARS-CoV-2 (24). Asymptomatic transmission lacks evidence (33, 246, 247). Some real-world data studies suggest that masks may increase the risk of infection (221, 248, 249). This contrasts with modeling and *in vitro* studies suggesting that masks reduce virus transmission (250–252), but these studies lack methodological rigor (35) and real-world applicability (20, 27, 33, 35). Errors in handling and material deficiencies diminish mask effectiveness (223, 228, 253–255), and real-world issues such as improper use by children, e.g., correct wearing rates as low as 24.5%–31.9% (71, 72), fitting issues — despite some contradictory reporting with observer bias (256) — frequent touching (10.7%–13.7% (257) undermine efficacy (13, 27, 29, 198), contradicting a falsely assumed, strong self-protection (13, 26, 29, 186, 258–260) (see Supplementary B, Table S1).

#### Lack of mask standardization (virus filtration)

There are no established standards for viral filtration in masks. Surgical and N95 masks perform poorly with regard to virus-sized particles (0.04–0.2 μm) (261), increasing transmission risk via potential nebulization of particles and leakage (27, 29, 198, 223, 225, 228, 229, 237, 238, 262, 263). SARS-CoV-2 can remain infectious on the polypropylene meshwork of masks for up to 2 weeks, longer than on many other materials (239, 240).

#### Negligible infectivity and COVID-19 course in children

Children show low SARS-CoV-2 infectivity and susceptibility, with a mortality rate of 0.0003% (54, 264, 265). Severe outcomes are rare, in contrast to the far-reaching drawbacks of mask mandates.

#### Scientifically proven adverse mask effects and mask-induced exhaustion syndrome (MIES)

##### Clinical symptoms

Surveys report mask-related impairments in children: 68% of 25,930 children experienced impairments, including irritability (60%), headaches (53%), and concentration issues (50%) (160), while 44.9% of 706 children showed respiratory discomfort (33.9%) and cutaneous repercussions (16.3%) (159). Other studies note breathing discomfort (53.1%) and mood changes (45.2%) (100), although mask utility–focused studies report lower side effects (∼20%) (97). Clinical symptoms are clearly identifiable (Figure 1).

##### Restriction of normal breathing

Masks increase breathing resistance (147, 148, 261, 266–273) and dead space volume (30, 42, 140, 180, 274–282), thereby reducing oxygen uptake and increasing CO₂ rebreathing (140, 271, 283–289).

In adults, respiratory minute volume drops by 19%–24% (266, 269, 290), most likely even more in children, who report breathing difficulties (43, 81, 97–100, 156, 159, 167, 169, 181).

##### Carbon dioxide rise

Masks elevate CO₂ levels (30, 31, 34, 148, 150, 151, 177, 179–181, 266, 269, 271, 273–276, 279, 283–286, 290–309), reaching 1.41%–3.7% (27, 30, 31, 140, 271, 274, 283, 285, 286, 288, 291, 292) and exceeding NIOSH limits (0.5%–3%) within minutes (27).

In children, CO₂ surpasses 0.5% after 5 min (30, 31). Animal studies link >0.3% CO₂ to nerve damage, toxicity, and anxiety (310–315). Low CO₂ (0.05%–0.5%) causes headaches and fatigue, difficulty breathing, and dizziness (141), while >1% induces acidosis and immune suppression among many other effects (141, 316–328).

CO₂ also risks tissue calcification via carbonic anhydrase (329–334).

##### Oxygen drop

Some studies show oxygen drops in children (170, 178, 183), while others, investigating predominantly short wear, find no change (30, 31, 95, 169, 171–173, 175–177, 179, 182).

Adult studies confirm causal oxygen drop (34, 146, 148, 149, 151, 153, 269, 271, 274, 279, 290, 293, 295, 296, 298, 299, 301, 307, 335–356), with subthreshold drops linked to stress and possible immune risks (357–364).

##### Microbial contamination

Improper mask use in children (13, 27, 29, 71, 72, 99, 100, 198, 254) increases contamination risks, potentially spreading pathogens (198).

##### Toxicity and carcinogenicity

Masks release carcinogenic microplastics, VOCs, and phthalates, exceeding safety limits (28). Children's masks show 43-fold phthalate and 25-fold VOC carcinogenic risk exceedances (28) (Figure 3).

##### Psychological and sociological symptoms

Masks impair communication (102, 105, 108–110, 115, 117, 132–134, 365–368) and emotion recognition (108, 111, 112, 115–118, 128, 133, 369), affecting learning (15, 16, 27, 101, 102, 108, 110, 129, 160).

##### MIES – mask-induced exhaustion syndrome

Prolonged mask use triggers MIES, with fatigue, headaches, and respiratory issues (27, 29, 34).

Subthreshold CO₂ and oxygen shifts cause brain metabolism changes (154), endangering the retina (370). They also lead to an increase in intraocular pressure (371), posing long-term risks (27, 29).

The literature indicates that prolonged mask-wearing can cause MIES, involving frequent, statistically significant physiological and psychological changes, including in children:

- Increase in dead space volume (30, 42, 140, 180, 274–281),
- Increase in breathing resistance (147, 148, 261, 266–272),
- Increase in carbon dioxide (30, 34, 148, 150, 151, 173, 177, 179–182, 266, 269, 271, 273–276, 279, 283, 284, 286, 290, 293–309, 345),
- Drop in blood oxygen saturation (34, 146, 148, 149, 153, 170, 178, 183, 266, 269, 274, 285, 290, 293, 295, 296, 298, 299, 301, 302, 304, 307, 335–349, 351–356, 360, 372),
- Increase in heart rate (34, 148, 151, 153, 183, 268, 271, 273, 279, 296, 300, 301, 338, 353, 372, 373),
- Decrease in cardiopulmonary capacity (175, 266, 269, 290),
- Feeling of exhaustion and fatigue (146, 149–151, 160, 169, 170, 172, 181, 266, 268, 269, 272, 273, 279, 290, 293, 296, 297, 299, 301, 307, 344, 345, 349, 359, 373, 374),
- Increase in respiratory rate (34, 42, 148, 167, 177, 273, 296, 299, 300, 344, 345),
- Changes in respiration (148, 172, 182, 266, 269, 273, 290, 296, 299, 300, 344),
- Difficulty breathing and shortness of breath (43, 81, 97–100, 148, 149, 151, 156, 159, 169, 170, 181, 266, 268, 273, 280, 296, 299, 303, 340, 341, 343–345, 351, 359, 360, 375–390),
- Headache (97, 100, 151, 152, 154, 160, 275, 291, 294, 299, 304, 335, 341, 343, 348, 350–352, 356, 359, 379, 383, 388, 391–400),
- Dizziness (148, 154, 170, 275, 291, 299, 304, 335, 343, 344, 346, 356, 383, 396, 399–404),
- Feeling of moisture and heat (42, 81, 149, 155, 156, 162, 260, 266, 268, 272, 273, 296, 345, 384, 390, 405),
- Drowsiness (with reduced ability to think and concentrate) (146–153, 160),
- Decrease in empathy perception (96, 102, 109–111, 115–118, 133, 158, 406),
- Impaired skin barrier function with acne, itching, and skin lesions (97, 148, 152, 161, 162, 164, 273, 335, 378, 380, 384, 389, 399, 407–421),
- False sense of security (13, 26, 78, 186, 258–260),
- Increase in blood pressure (34, 148, 168, 175, 266, 268, 269, 290, 296, 303, 353, 422),
- Increase in the temperature of the skin under the mask (140, 162, 262, 272, 273, 345, 390, 423),
- Increase in the humidity of the air under the mask (140, 162, 272, 345, 390, 423),
- Communication disorder (96, 106, 110, 116–118, 128, 129, 131, 133, 134, 151, 152, 368, 384, 424, 425),
- Voice disorder (100, 132, 133, 384, 426),
- Perceived discomfort (97, 98, 100, 155, 159, 160, 164, 266, 296, 380, 390),
- Increased anxiety (27, 110, 129, 160, 167, 388, 424, 426–428),
- Elevated mood swings or depressive mood (100, 110, 160, 388, 424, 426, 427),
- Toxicological risks (28),
- Biological hazards (198),
- Risk to young life, including unborn and children (nerve damage, testicular damage, and stillbirths) (27).

**Figure 4:**
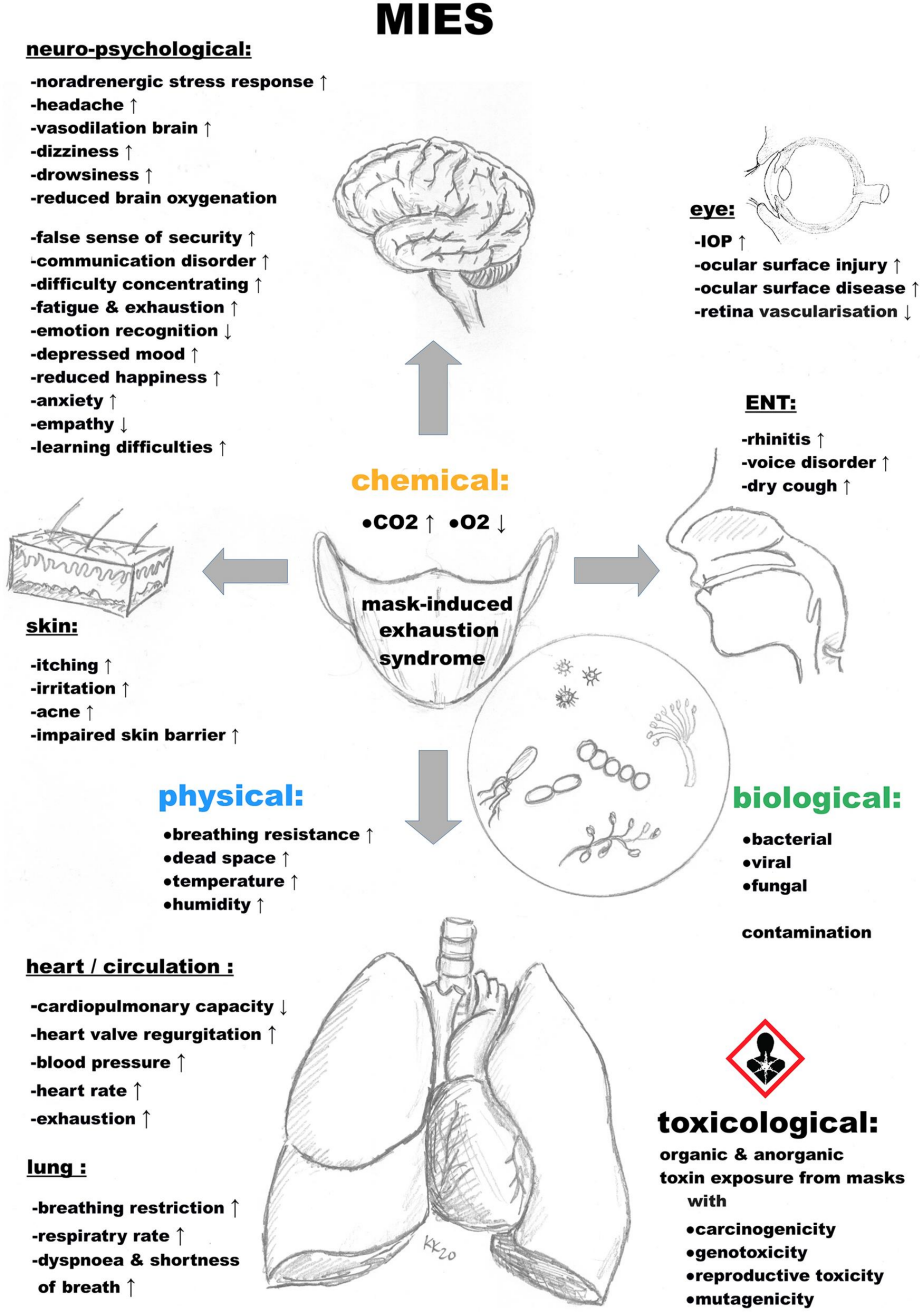
Mask-induced exhaustion syndrome (MIES) involves adverse chemical, physical, biological, and toxicological effects, impacting organ systems like eyes (e.g. increased intraocular pressure) and ENT (ear, nose, and throat). But also the nervous system and brain (e.g. noradrenergic stress response, anxiety, exhaustion and fatigue), lung (e.g. shortness of breath), heart (e.g. heart valve regurgitation), skin (e.g. irritation). These effects are documented with statistically significant results in children, as compiled from scientific literature.

Altogether, MIES can have long-term clinical consequences, especially for vulnerable groups including children (Figure 4).

### Risk assessment of masks and children

With regard to mask use in children, authorities must assess risks assuming worst-case scenarios (36). In past pandemics like SARS-CoV-2, risk–benefit assessments for children's mask use required such worst-case scenario evaluations. However, the mask mandates lacked evidence of efficacy, viral filtration standards, and relevance because of children's low infectivity and 0.0003% mortality rate (54), with improper handling increasing infection risks and outweighing benefits (13, 27, 29, 71, 72, 99, 100, 198, 254).

For comparison, chlorine dioxide, a drinking-water disinfectant used worldwide and biocide effective against SARS-CoV-2 *in vitro* (429), has a human risk assessment based on rodent studies. The No-Observed-Adverse-Effect Level (NOAEL) was 3 mg/kg/day, showing neurotoxicity above this level (430). Applying interspecies and interhuman uncertainty factors (10 each, total 100), the safe human dose (Reference Dose, RfD) is 0.03 mg/kg/day (430). This balances biocidal efficacy against safe exposure, a process absent for mask mandates.

Masks pose psychological, physiometabolic, toxicological, and clinical risks, especially for children (Figures 1, 3, 4). Notably, CO_2_ exposure from masks averages 0.7%–1.39%, with maxima of 1.52% and 2.5% (Figure 2) (30, 31), exceeding safety thresholds. Animal data show that 0.3% CO_2_ levels cause irreversible neuronal damage in juvenile rodents (27). Applying a conservative safety factor of 3–10 (431), safe CO_2_ exposure is well below 0.3%, indicating that masks pose neurodevelopmental risks (27). Global educational declines [OECD PISA 2022 (242, 432)] suggest cognitive impacts.

Considering proven mask harms (Figures 1–4; Table 1), unproven viral protection, and the lack of filtration standards, the risk–benefit ratio is unfavorable (Supplementary Tables E,F, Supplementary A). The negligible COVID-19 risk in children (Table 2) reinforces this finding (54). Overall, our results indicate that masks may pose detriments on children, their parents, and school systems. Policymakers and public health authorities may refer to our findings when considering mask policies for children.

**Table 1:** Summary of key facts (scientific evidence) on the potential adverse effects associated with the use of masks by children for risk assessment.

Masks and children risk assessment—adverse effects	Scientific evidence		
Toxicological risk (carcinogenic, mutagenic, and physiometabolic)	Yes		
Bacterial, fungal, and viral burden (contamination)		Possible	
Physical symptoms	Yes		
Exacerbation of existing diseases	Yes		
Triggering of new diseases	Yes		
Psychological symptoms	Yes		
Sociological/developmental disruptive effect		Possible	
Large absence of undesirable effects			No

**Table 2:** Summary of the most important facts on environmental influences and contextual factors related to masks and children for risk assessment, with particular consideration given to masking children during COVID-19.

Masks and children risk assessment—contextual factors	Evidence/basics		
Mask standardization (bacterial filtration)	Yes		
Mask standardization (particle filtration)	Yes		
Mask standardization (virus filtration)			No
Effectiveness of masks against viruses in children		Unproven	
Ensuring appropriate use of masks in children		Unproven	
High SARS-CoV-2 infectivity in children			No
Frequent devastating COVID-19 course in children			No

**Figure 5:**
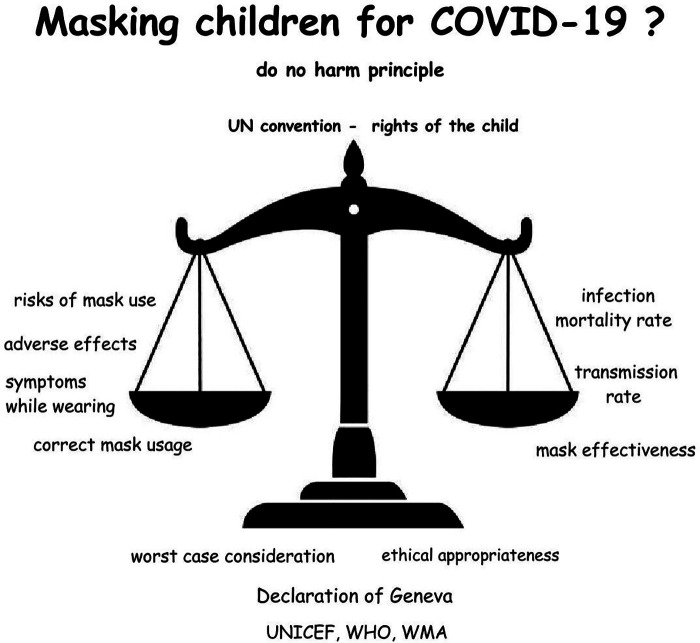
A graphical summary of mask risk assessment for children, incorporating key ethical principles, symbolized by the scales of Justitia representing justice. Mask use poses clear, non-negligible risks, while effectiveness against viruses lacks convincing empirical evidence, relying on assumptions without robust support from randomized controlled trials.

Applying key ethical and precautionary frameworks — specifically a worst-case assessment (36), WHO/UNICEF's “do no harm” principle (10), the Hippocratic oath, the Geneva Declaration (241), and the UN Convention on the Rights of the Child (16) — to our findings leads to the conclusion that masking children is not justified (Figure 5). This position is based on the unfavorable risk-benefit ratio demonstrated in our data and is supported by a growing body of scientific evidence (15, 24–31, 33–35, 44, 197, 198, 433–436).

## Limitations

This rapid review searched only one database, excluding gray literature, potentially underestimating mask-related adverse effects. It focused on individuals under 18, omitting fetal risks, although mask use in pregnant women is known to pose concerns (27). Limited literature precluded analyzing the impact of mask-wearing and excessive hygiene on immune systems, particularly reduced bacterial stimulation, which may increase susceptibility to infections like rhinoviruses, respiratory syncytial virus, and streptococci postmeasures (437–440).

## Conclusion

Masks may be justified for children in rare, short-term scenarios (e.g., bush fire smoke and tuberculosis source control) as a piece of medical advice but not as a mandate. However, mandating masks for schoolchildren e.g., during the SARS-CoV-2 pandemic, lacked scientific basis, rather driven by political motives. The risk–benefit analysis is unfavorable (Tables 1, 2; Figure 5) with significant adverse effects, including unacceptable CO_2_ (Figure 2), many clinical symptoms (Figure 1), toxicological risks and levels (Figure 3) and MIES (Figure 4, Supplementary B: Tables S3–S5), but devoid of any convincing empirical evidence of viral protection (Supplementary A, Table E, Supplementary B, Table S1).

Future interventions must prioritize evidence-based approaches in the best interests of children. Other non-pharmaceutical measures, such as hand hygiene, ventilation, and sick leave, are safer, more effective, and less legally complex than masks for reducing viral transmission. Promoting immune health through nutrition, the microbiome, and environmental factors should guide research (Supplementary B: Tables S1–S6). Based on the available evidence, the justification of mandatory mask policies for children appears weak and unsupported.

## Data Availability

Publicly available datasets were analyzed in this study. These data can be found here: all data used in the review are publicly available (referenced publications).

## Supplementary material

Data sheet 1: Supplementary A

Data sheet 2: Supplementary B

Data sheet 3: Supplementary C
