# Supplementary material for: Risks and benefits of face masks in children
Source: Front Pediatr. 2026 Mar 13;14:1679586. doi: 10.3389/fped.2026.1679586 (PMC13047964; doi:10.3389/fped.2026.1679586)
Supplement: Supplementary file 1 — Data sheet 1: Supplementary A [file Datasheet1.pdf]

Supplement A

Risks and benefits of face masks in children

Kai Kisielinski [1,*], Claudia Steigleder-Schweiger [2], Susanne Wagner [3], Stephan Korupp [4], Stefan Hockertz [5], Oliver Hirsch[6]

[1] Clinical Medicine (Surgery), Emergency Medicine and Social Medicine, Private Practice, 40212 Düsseldorf, Germany.

[2] Department of Paediatrics, University Hospital of Salzburg, Paracelsus Medical University, 5020 Salzburg, Austria.

[3] Non-Clinical Expert, Veterinarian, Wagner MSL Management, 15831 Mahlow, Germany.

[4] Surgeon, Emergency Medicine, Private Practice, 52070 Aachen, Germany.

[5] Toxicology, Pharmacology, Immunology, tpi consult AG, Haldenstr. 1, CH 6340 Baar, Switzerland.

[6] Department of Psychology, FOM University of Applied Sciences, 57078 Siegen, Germany

*** Correspondence:** *kaikisielinski@yahoo.de*

Supplement A contains the Tables A-F.

It provides the methodological framework and evidence profile for the scoping review.

Tables A–C outline the PICO framework, search strategy, and inclusion/exclusion criteria, while Tables D–F categorize studies, present the GRADE equivalent evidence profile, and summarize evidence quality.

See Supplement B for supplementary figures and extraction tables of included publications.

SUPPLEMENT A

Table A. The PICO framework (participants, intervention, comparisons, outcomes) of the scoping review.

**P: Participants**	**I: Intervention**
children	wearing different face masks:<br>medical masks (surgical mask or similar) or<br>FFP1, FFP2, N95 (including similar respirator types like KF94, KN 95 and FFP3)<br>for medical-hygienic or other protective reasons
**C: Comparison**	**O: Outcomes**
Wearing a face mask at rest or during activities compared to no mask-wearing	effectiveness against animate or inanimate hazards,<br>effects according to the WHO biopsychosocial model including physio-metabolic and toxicological as well as microbiological effects,<br>Reported claims of effects (e.g., protective efficacy, adverse effects)
**analysed study designs:** original research (e.g. randomised and non-randomised controlled trials, observational studies), reviews, case reports, technical notes	

Table B. Search strategy adopted for the scoping review.

Database	Descriptors
PubMed/MEDLINE	((mask[Title/Abstract]) OR (face mask[Title/Abstract]) OR (facemask[Title/Abstract]) OR (surgical mask[Title/Abstract]) OR (FFP1[Title/Abstract]) OR (FFP2[Title/Abstract]) OR (FFP3[Title/Abstract]) OR (N95[Title/Abstract]) OR (KF94[Title/Abstract]) OR (KN95[Title/Abstract])) AND children[Title/Abstract]

Table C. Inclusion and exclusion criteria for papers.

Criteria / PICO component	Inclusion	Exclusion
language	English and German	all other languages
study type	-systematic reviews,<br>-scoping reviews,<br>-narrative reviews,<br>-randomised controlled trials (RCT),<br>-non-randomised controlled trials (nRCT),<br>-observational studies,<br>-cohort studies,<br>-case control studies,<br>-cross-sectional,<br>-questionnaire studies,<br>-case series,<br>-case reports,<br>-expert opinions.	none
mask use	mask use evaluated in:<br>-resting conditions<br>(e.g. sitting),	studies not evaluating mask use (e.g., no mask-wearing conditions)

Criteria / PICO component	Inclusion	Exclusion
	-activities/loading<br>(standing, moving, walking, etc.).	
mask type	medical masks (surgical mask or similar) or<br>FFP1, FFP2, N95 (including similar respirator types like KF94, KN 95 and FFP3).	anaesthesiology and ventilation masks,<br>orthodontic and other masks.
population	children of all ages	adults
effects (positive)	-protective efficacy against infectious agents,<br>-psychological and sociological aspects,<br>other effects (e.g., comfort, compliance).	no effects reported,<br>focus on technical aspects (e.g. mask design),<br>focusing solely on epidemiological outcomes without assessing direct mask effects.
effects (negative)	-lack of protective efficacy against infectious agents,<br>-physiology of children,<br>-psychological and sociological aspects,<br>-physical symptoms and clinical conditions,<br>-toxicological aspects,<br>other effects (e.g., comfort, compliance).	no effects reported,<br>focus on technical aspects (e.g. mask design),<br>focusing solely on epidemiological outcomes without assessing direct mask effects<br>.

Table D. Categorisation of included studies into meaningful groups for synthesis based on the similarity of their PICO components.

Criteria / PICO component	Studies	Rationale
effectiveness of masks against SARS-CoV-2 (positive and negative effects)	Bernhard et al. 2024, Chandra et al. 2022, Coma et al. 2022, Høeg et al. 2023, Jarnig et al. 2022a, Jarnig et al. 2022b, Jefferson et al. 2023, Juutinen et al. 2023, Kosec et al. 2023, Ladhani 2022, Littlecott, et al. 2024, Ludvigsson et al. 2021, Orey et al. 2023, Sandlund et al. 2023, Satapathy et al. 2024, Svetina & Viera 2024	Publications with focus on evaluating protective efficacy of face masks against SARS-CoV-2 in children
positive effects	Banholzer et al. 2023, Holm et al. 2021, Janapatla et al. 2021, Matsuda et al. 2023, Qin et al. 2021, Science et al. 2022, Sombetzki et al. 2021, Suess et al. 2011, Suess et al. 2012, Theuring et al. 2021, Villers et al. 2022, Xiao et al. 2021	Publications with main focus on evaluating protective efficacy of face masks against diverse environmental hazards including infection except SARS-CoV2 in children and also their positive psychological aspect
psychological and sociological effects	Ammann et al. 2022, Aronu et al. 2020, Bourke et al. 2023, Carnevali et al. 2022, Charney et al. 2021, Chester et al. 2022, Chhabra et al. 2019, Coelho et al. 2022, Drössler et al. 2023, Flaherty et al. 2023, Freiberg et al. 2021, Giordano et al. 2024, Ger et al. 2023, Gil et al. 2023, Gori et al. 2021, Halbur et al. 2021, Hahn et al. 2023, Kwon et al. 2022, Kwon et al. 2023, Lalonde et al. 2022, Le et al. 2023, Liu et al. 2024, Mickells et al. 2021, Mitsven et al. 2022, Miyazaki et al. 2023, Nobrega et al. 2020, Omaleki et al. 2024, Preest et al. 2024 Schlegtendal et al. 2022, Schwarz et al. 2022, Shaw et al. 2024, Singh et al. 2023, Sivaraman et al. 2020, Smart et al. 2020, Stajduhar et al. 2022, Surrain et al. 2023, Tamon et al. 2022, Taxacher et al. 2023, Thomson et al. 2022, Wild & Kornfeld 2021, Xiang et al. 2023	Publications evaluating psychological and sociological effects of face masks in children

Criteria / PICO component	Studies	Rationale
physical symptoms and clinical conditions	Assathiany et al. 2021, Beytout et al. 2021, Kaliyadan et al. 2022, Kisielinski et al. 2021, Ratchatavech et al. 2022, Schwarz et al. 2021, Shoaib et al. 2022, Zanotti et al. 2020	Publications evaluating physical symptoms and clinical conditions in children wearing face masks
physio-metabolic and toxicological effects	Ahmadi et al. 2024, Amirav an Lavie 2022, Brooks et al. 2023, Canellas et al. 2023, Castro et al. 2023, Eberhart et al. 2021, Goh et al. 2019, Happernegg and Kerbl 2023, Hodges et al. 2024, Kisielinski et al. 2023, Kisielinski et al. 2024, Lubrano et al. 2021, Lubrano et al. 2021, Lubrano et al. 2021, Lubrano et al. 2022, Mallet et al. 2022, Martellucci et al 2022, Reychler et al. 2022, Roberge 2011, Schulte-Körne et al. 2022, Weigelt et al. 2023, Walach et al. 2022, Wang et al. 2021	Reviews, RCTs, nRCTs, observational studies, case series, communications evaluating physio-metabolic and toxicological effects in children
publications making claims about face masks (effectiveness, positive and negative effects)	Esposito and Principi 2020, Gyawali 2021, Huppertz et al. 2021, Lopes et al. 2021, Moschovis et al. 2021, Villani et al. 2020	Publications making claims about face masks (effectiveness, positive and negative effects)

Table E: GRADE equivalent evidence profile of included studies regarding their statements on masks in children (outcomes in first column).

mask outcome	study & year	n	study type (evidence level)	funding & observer bias	overall bias	down- / upgrading & reason	reasoning for rating	final rating of evidence quality
**effectiveness (SARS-CoV-2)**	Bernhard 2024	108	cross sectional	serious	serious	**down** seroprevalence but not illness evaluated, no separation of diverse measures	self-report of mask wearing (questionnaire), uncertain exclusion of other protective measures, government funding	**very low**
	Littlecott 2024	15*	systematic review	moderate	serious	**down** narrative synthesis of low-certainty evidence (heterogeneity, risk of bias, limited focus on specific measures)	narrative approach, no meta-analysis, transmission-related outcomes from heterogenous studies, government funding	**low**
	Jarnig 2022b	614	retrospective cohort	serious	serious	**down** unequal control group, calculated and extrapolated SARS-CoV-2 infections	infection rates calculated/estimated, control group questionable, small difference, government funding	**very low**
	Orey 2023	500	cross-sectional	moderate	serious	**down** antibody prevalence but not illness evaluated (herd immunity)	preventive practice questionnaire, antigen test kit results (not illness), no precision and no clear separation of diverse measures	**very low**
	Svetina & Kosec 2023	n.a.	research-based commentary	serious	serious	**down** no separation of diverse measures, no control group, correlation vs causality	assumptions of masks' effectiveness are based on the modelling of mask wearing and infection rates	**very low**

mask outcome	study & year	n	study type (evidence level)	funding & observer bias	overall bias	down- / upgrading & reason	reasoning for rating	final rating of evidence quality
**effectiveness (SARS-CoV-2)**	Viera 2024	14*	systematic review	moderate	serious	**down** narrative synthesislow-certainty evidence, no separation of diverse measures	narrative approach, no meta-analysis, heterogeneity of study designs with different populations	**low**
**non-effectiveness (SARS-CoV-2)**	Chandra 2022	1832 **	observational	low	low	**n.a.**	Large sample size with robust observational design, but potential confounding factors	**moderate**
	Coma 2022	59931 4	observational	moderate	moderate	**n.a.**	very large sample size, no associated between mask mandates and incidence or transmission	**moderate**
	Høeg 2023	n.a.	narrative review	moderate	serious	**n.a.**	nuanced narrative analysis with consideration of no clear net benefit of masking very young children	**low**
	Jarnig 2022a	881	survey	moderate	moderate	**up** adverse effects and risk approach, risk assessment with worst case scenario consideration consistent with findings across other studies	large sample size, correlation of low mask wearing accuracy and children, differences in supervised / unsupervised situation	**moderate**

mask outcome	study & year	n	study type (evidence level)	funding & observer bias	overall bias	down- / upgrading & reason	reasoning for rating	final rating of evidence quality
non-effectiveness (SARS-CoV-2)	Jefferson 2023	3*	systematic review	low	low	**n.a.**	Cochrane systematic review with meta-analysis	**high**
	Juutinen 2023	1103025	observational	low	low	**up** very huge sample size, consistency of findings with other large studies	very huge sample size, no associated between mask mandates and incidence	**high**
	Ladhani 2022	16*	narrative review	moderate	serious	**n.a.**	evidence based narrative approach	**low**
	Ludvigsson 2021	n.a.	editorial	moderate	serious	**n.a.**	evidence based narrative approach	**low**
	Sandlund 2023	22*	systematic review	low	low	**n.a.** narrative synthesis due to study quality of included studies	large amount of evaluated studies, neutral assessment with high quality Cochrane assessment tools, consisent with Cochrane review findings	**high**
	Satapathy 2024	320	observational	low	moderate	**up:** adverse effects and risk approach, consistent findings	sufficient sample size, correlation of low mask wearing accuracy and children shown	**high**
positive effects	Banholzer 2023	90	observational with modelling	serious	serious	**down:** serious limitations in terms of directness, precision, and risk of bias	absence of hard endpoints (infection and disease) empirically uncertain	**very low**
	Holm 2021	34*	narrative review	moderate	moderate	**n.a.**	nuanced narrative evaluation of scientific facts	**low**

mask outcome	study & year	n	study type (evidence level)	funding & observer bias	overall bias	down- / upgrading & reason	reasoning for rating	final rating of evidence quality
positive effects	Janapatla 2021	n.a.	observational	serious	serious	**down:** no clear separation of diverse measures and other confounders of infection rate	statistical approach with many unconsidered disturbance- and influencing variables	**very low**
	Matsuda 2023	24145	survey	serious	serious	**down:** possible retrospective recall bias, no separation of diverse measures	self-reported data (measurement bias, recall bias), inherent risk of bias and many confounding factors	**very low**
	Qin 2021	1199	survey	serious	serious	**down:** risk of bias, imprecision, indirectness, and confounding	self-reported data (measurement bias), only correlation no causality in focus	**very low**
	Science 2022	171	RCT	moderate	moderate	**down:** simulated environment, no real life school-settings	sufficient sample size,but indirect nature of the simulated school environment and possible bias issues	**low**
	Sombetzki 2021	475	observational/ modeling	serious	serious	**down:** serious risk of bias, especially with confounding and reporting bias	surveillance data used for multivariate linear regression model and two further separate regression models	**very low**
	Suess 2011	39	RCT	moderate	moderate	**down:** self-report data, no separate consideration of children and adults	hard endpoints of infection, but questionnaire design and data from self-report	**low**

mask outcome	study & year	n	study type (evidence level)	funding & observer bias	overall bias	down- / upgrading & reason	reasoning for rating	final rating of evidence quality
**positive effects**	Suess 2012	84***	RCT	moderate	moderate	**down**: no clear separation of diverse confounders and no separate consideration of children and adults, source control and self-protection could not be distinguished	hard endpoints of infection, but imprecision in examining the mask effects, unclear separation of children and adults	**low**
	Theuring 2021	1119	observational	serious	serious	**down:** very limited control for confounding factors	no separation and no individual evaluation of specific measures	**very low**
	Villers 2022	1	modelling	moderate	serious	**down:** modeling approach may not fully capture the complexities of real-world school environments or community behavior, further contributing to bias	indirectness, uncertainty in model assumptions, and lack of direct empirical evidence.	**very low**
	Xiao 2021	514314	observational	moderate	moderate	**down:** no clear separation of diverse measures, 39 different infectious diseases evaluated	very big sample size, but control group mathematically constructed with modelled incidence, indirect effect of measures	**low**

mask outcome	study & year	n	study type (evidence level)	funding & observer bias	overall bias	down- / upgrading & reason	reasoning for rating	final rating of evidence quality
**psychological and sociolo-gical effects**	Ammann 2022	595	survey	serious	serious	**down:** descriptive nature, risk of bias in participant selection and reporting, focus on perceptions	self-reported online survey data (measurement bias)	**very low**
	Aronu 2020	387	survey	moderate	serious	**n.a.**	cross-sectional study in healthcare setting	**low**
	Bourke 2023	74	observational	serious	serious	**n.a.**	less ecological validity due to artificial conditions	**low**
	Carnevali 2022	129*	systematic review	serious	serious	**down:** possible selection and participation bias	narrative approach, several limitations and risk of biases	**low**
	Charney 2021	n.a.	commentary	moderate	serious	**n.a.**	nuanced narrative evaluation of scientific facts	**low**
	Chester 2022	131	observational	moderate	moderate	**n.a.**	coss-sectional study with longitudinal subsample	**moderate**
	Chhabra 2019	378	observational	moderate	moderate	**n.a.**	cross-sectional study in healthcare setting	**moderate**
	Coelho 2022	190	observational	moderate	moderate	**n.a.**	cross-sectional study with school lesson simulation	**moderate**
	Drössler 2023	n.a.	observational	moderate	moderate	**n.a.**	qualitative observational (interview study)	**moderate**
	Flaherty 2023	30	nRCT	moderate	moderate	**n.a.**	non-randomized controlled trial with moderate risk of bias and imprecision	**moderate**
	Freiberg 2021	13*	systematic review	moderate	moderate	**n.a**	descriptive narrative synthesis	**moderate**

mask outcome	study & year	n	study type (evidence level)	funding & observer bias	overall bias	down- / upgrading & reason	reasoning for rating	final rating of evidence quality
**psychological and sociolo-gical effects**	Giordano 2024	77	observational	low	moderate	**n.a**	small sample	**moderate**
	Ger 2023	79	observational	moderate	moderate	**n.a.**	cross-sectional design with real-world relevance and cross-cultural comparison	**moderate**
	Gil 2023	54	observational	low	moderate	**n.a.**	small sample, cross-sectional	**moderate**
	Gori 2021	80	nRCT	low	moderate	**n.a.**	moderate risk of bias, moderate imprecision and low indirectness	**moderate**
	Halbur 2021	10	observational	moderate	moderate	**down:** limited generalizability, focus on a very specific population	single-case experimental design, very small sample size	**low**
	Hahn 2023	939	observational	low	moderate	**n.a.**	cross-sectional, large-scale design, big sample	**moderate**
	Kwon 2022	74	survey	serious	serious	**n.a.**	cross-sectional design, questionnaire	**low**
	Kwon 2023	67	nRCT	moderate	moderate	**n.a.**	experimental design, ecological validity concerns	**moderate**
	Lalonde 2022	18	nRCT	moderate	moderate	**n.a.**	laboratory conditions, small sample, thus lower ecological validity	**moderate**
	Le 2023	8420	survey	moderate	serious	**n.a.**	possible selection bias	**low**
	Liu 2024	72	nRCT	low	moderate	**n.a.**	laboratory conditions, low ecological validity	**moderate**
	Mickells 2021	n.a.	survey	serious	serious	**n.a.**	data from daily teacher surveys, introducing reporting bias	**low**

mask outcome	study & year	n	study type (evidence level)	funding & observer bias	overall bias	down- / upgrading & reason	reasoning for rating	final rating of evidence quality
**psychological and sociolo-gical effects**	Mitsven 2022	15	observational	serious	serious	**n.a.**	small sample size, between-cohort rather than within-cohort comparison, differences in the two cohorts	**low**
	Miyazaki 2023	282	observational	moderate	moderate	**n.a.**	lack of randomization and potential confounding factors from comparing children and adults	**moderate**
	Nobrega 2020	n.a.	letter to editor	low	moderate	**n.a.**	lack of rigorous peer-review, opinion piece based on existing research or observational data	**low**
	Omaleki 2024	648	survey-based observational	serious	serious	**n.a.**	qualitative interviews, self-reported data	**low**
	Preest 2024	45*	systematic review	moderate	serious	**n.a.**	narrative synthesis of low-certainty evidence	**low**
	Schlegtendal 2022	133	RCT	moderate	moderate	**down:** no appropriate control group, time period for recovery too short, relatively small time of evaluation	inconsistent age groups, all included evaluated children were already chronically adapted to mask wearing and experienced already chronic mask effects	**low**
	Schwarz 2022	26	observational	moderate	moderate	**n.a.**	experimental design, potential observer and sampling bias, potential imprecision	**moderate**
	Shaw 2024	39*	scoping review	moderate	moderate	**n.a.**	moderate methodological rigor rather than a high degree of methodological rigor	**moderate**

mask outcome	study & year	n	study type (evidence level)	funding & observer bias	overall bias	down- / upgrading & reason	reasoning for rating	final rating of evidence quality
psychological and sociolo-gical effects	Singh 2023	28	observational	moderate	serious	**n.a.**	limited ecological validity, small sample size and not particularly representative sample	**low**
	Sivaraman 2020	6	intervention (single-case experimental)	serious	serious	**n.a.**	non-randomized nature, very small sample size and potential observer bias	**low**
	Smart 2020	24	observational	moderate	moderate	**n.a.**	small sample size, possible Hawthorne effect	**moderate**
	Stajduhar 2022	72	RCT	moderate	moderate	**n.a.**	exclusive use of male faces, lack of ethnic diversity	**moderate**
	Surrain 2023	45	RCT	moderate	moderate	**n.a.**	potential biases and questionable generalisability (quiet setting at the child's school during assessments)	**moderate**
	Tamon 2022	102	survey	serious	serious	**n.a.**	confounding and observer bias, limited generalisability	**low**
	Taxacher 2023	40	observational	moderate	moderate	**n.a.**	observational design and moderate risk of bias	**moderate**
	Thomson 2022	n.a.	communi-cation	moderate	moderate	**n.a.**	policy analysis and theoretical paper	**low**
	Wild & Kornfeld 2021	n.a.	opinion	serious	serious	**n.a.**	theoretical review paper	**low**
	Xiang 2023	40	observational	moderate	moderate	**n.a.**	real-world data on mask-wearing compliance	**moderate**

mask outcome	study & year	n	study type (evidence level)	funding & observer bias	overall bias	down- / upgrading & reason	reasoning for rating	final rating of evidence quality
**physical symptoms and clinical conditions**	Assathiany et al. 2021	2954	survey	moderate	moderate	**up:** adverse effects and risk approach, consistent findings with other research	identifies potential adverse effects in a large cohort	**moderate**
	Beytout et al. 2021	92	survey	low	moderate	**up:** adverse effects and risk approach, consistent findings with other research	self-reported data, acceptable sample size, important hints to possible adverse effects	**moderate**
	Kaliyadan et al. 2022	7	observational case series	moderate	moderate	**up:** adverse effects and risk approach, important possible but serious adverse effect identified	smaller clinical case series illustrating possible adverse effects	**moderate**
	Kisielinski et al. 2021	65*	scoping review	low	low	**up:** adverse effects and risk approach, important risks identified	systematic literature search, also quantitative analysis, many clinical effects of the masks, including for children, are described and explained, inclusion of experimental data	**high**
	Ratchatavech et al. 2022	706	survey	moderate	moderate	**up:** adverse effects and risk approach, consistent findings with other research	self-reported data, sufficient sample size, important hints to possible adverse effects	**moderate**

**mask outcome**	**study & year**	**n**	**study type (evidence level)**	**funding & observer bias**	**overall bias**	**down- / upgrading & reason**	**reasoning for rating**	**final rating of evidence quality**
**physical symptoms and clinical conditions**	Schwarz et al. 2021	25930	survey	moderate	moderate	**up:** adverse effects and risk approach, consistent findings with other research	large sample size illustrating numerous possible adverse effects	**moderate**
	Shoaib et al. 2022	2	case study	moderate	moderate	**up:** adverse effects and risk approach, important possible but serious adverse effect identified	given the serious adverse effects identified, such as corneal abrasion in children, this study may have important public health implications that warrant an upgrade in evidence rating.	**moderate**
	Zanotti et al. 2020	n.a.	letter to editor	moderate	moderate	**up:** adverse effects and risk approach	adverse effects are causally plausible, clinically significant and widespread	**moderate**
**physio-metabolic and toxicological effects**	Ahmadi et al. 2024	110	observational	moderate	moderate	**n.a.**	design is appropriate, sample size is adequate, objective nature of the outcome measure and concern (observer bias) has less impact	**moderate**

mask outcome	study & year	n	study type (evidence level)	funding & observer bias	overall bias	down- / upgrading & reason	reasoning for rating	final rating of evidence quality
**physio-meta-bolic and toxic-ological effects**	Amirav and Lavie 2022	3	case series	moderate	moderate	**up:** severe nature of the adverse effects described and the importance of early warning	study limitations with small sample size, potential selection bias, value of the information (early warning, adverse effects and risk approach)	**moderate**
	Brooks et al. 2023	49	observational	moderate	moderate	**n.a.**	use of objective measurements, study directly measures the physiological effects	**moderate**
	Canellas et al. 2023	11****	experimental	moderate	low	**n.a.**	direct, lab-based evidence of contaminant migration from masks, large effect, but use of a simulant rather than real saliva	**moderate**
	Castro et al. 2023	50	observational	moderate	moderate	**n.a**	short evaluation time, direct measure of physiological values	**moderate**
	Eberhart et al. 2021	2*	narrative review	moderate	moderate	**n.a**	potential selection bias, potential indirectness, only two studies on children evaluated	**low**
	Goh et al. 2019	106	RCT	serious	moderate	**down**: high risk of funding bias due to industry involvement and potential conflict of interes	strong methodology, but involvement of funding company and potential conflict of interest introduce bias	**low**
	Happer-negg and Kerbl 2023	15	observational	moderate	moderate	**n.a.**	small sample size (pilot study), but methodological rigor	**moderate**

mask outcome	study & year	n	study type (evidence level)	funding & observer bias	overall bias	down- / upgrading & reason	reasoning for rating	final rating of evidence quality
**physio-metabolic and toxicological effect**	Hodges et al. 2024	50	observational	moderate	moderate	**n.a.**	short examination time (6 min) may not reflect real-world conditions or long-term effects	**moderate**
	Kisielinski et al. 2023	43*	scoping review	low	low	**up:** adverse effects and risk approach, important risks identified	focus on adverse effects, particularly the risk of CO_2 rebreathing in vulnerable populations, inclusion of experimental data	**high**
	Kisielinski et al. 2024	24*	scoping review	low	low	**up:** adverse effects and risk approach, important risks identified	toxicological approach, inclusion of experimental data, highlights significant public health implications	**high**
	Lubrano et al. 2021	47	observational	moderate	moderate	**n.a.**	small sample size and short observation period, objective measurements of respiratory function	**moderate**
	Lubrano et al. 2021	22	RCT	low	low	**n.a.**	objective measurements, but the small sample size and relatively short observation period limit its precision	**high**
	Lubrano et al. 2021	22	RCT	moderate	moderate	**n.a.**	small sample size and short observation period limit precision	**moderate**

mask outcome	study & year	n	study type (evidence level)	funding & observer bias	overall bias	down- / upgrading & reason	reasoning for rating	final rating of evidence quality
**physio-meta-bolic and toxic-ological effects**	Lubrano et al. 2022	30	observational	moderate	moderate	**n.a.**	small sample size and short observation period, objective measurements of cardio-respiratory function in a vulnerable population, and the findings have significant implications for public health	**moderate**
	Mallet et al. 2022	25	observational	moderate	moderate	**n.a.**	small sample size and short observation period, objective measurements of respiratory function	**moderate**
	Martellucci et al 2022	24	observational	moderate	moderate	**up:** adverse effects and risk approach, important risks identified	small sample size and short observation period, objective measurements, large effect, dose response gradient	**high**
	Reychler et al. 2022	38	observational	moderate	moderate	**n.a.**	small sample size and very short observation period, objective measurements	**moderate**
	Roberge 2011	68*	narrative review	low	moderate	**up:** discussion of significant safety and physiological concerns related to mask use in children	clinically relevant, scientific nuanced approach identifies important safety issues	**moderate**
	Schulte-Körne et al. 2022	11	RCT	moderate	moderate	**n.a.**	very small sample size and short evaluation duration (12 minutes)	**moderate**

mask outcome	study & year	n	study type (evidence level)	funding & observer bias	overall bias	down- / upgrading & reason	reasoning for rating	final rating of evidence quality
**physio-meta-bolic and toxic-ological effects**	Weigelt et al. 2023	20	observational	moderate	moderate	**n.a.**	small sample size and short test duration, exclusion of oxygen saturation values, limited to elite young male footballers	**moderate**
	Walach et al. 2022	45	observational	low	moderate	**up:** large effect, dose response gradient, adverse effects and risk approach, important risks identified	clinically relevant, despite small sample size and short observation period, objective measurements of large effects	**high**
	Wang et al. 2021	n.a.	communication	moderate	serious	**up:** focus on potential serious health risks, including cardiovascular and respiratory effects, with specific mention of sudden death incidents	highly clinically relevant, synthesis of existing literature to highlight potential risks	**moderate**
**claims about masks**	Esposito and Principi 2020	n.a.	opinion	moderate	serious	**n.a.**	descriptive and not providing synthesized evidence or quantitative analysis	**low**
	Gyawali 2021	n.a.	narrative review	moderate	serious	**down:** narrative, indirectness, imprecision,	does not provide or reference sufficient high-quality empirical studies to substantiate claims	**very low**

mask outcome	study & year	n	study type (evidence level)	funding & observer bias	overall bias	down- / upgrading & reason	reasoning for rating	final rating of evidence quality
**claims about masks**	Huppertz et al. 2021	n.a.	statement	serious	serious	**down:** influenced by various non-scientific factors, lack of reported adverse effects, indirectness, imprecision	lack of detailed evidence or citations to back claims, potential serious bias	**very low**
	Lopes et al. 2021	n.a.	statement	serious	serious	**down:** statements potentially swayed by organizational priorities or external pressures, claim of equal protection lacks specific evidence cited	statement does not reference adequate studies or data, lack of direct, empirical evidence supporting the specific claims	**very low**
	Moschovis et al. 2021	n.a.	opinion	serious	serious	**down:** lack of experimental data means conclusions are based on theory and assumption, potentially influenced by funding bodies' interests	the reliance on theoretical models rather than real-world evidence underscores the speculative nature of the conclusions drawn	**very low**
	Villani et al. 2020	n.a.	statement	serious	serious	**down:** inherently biased towards the group's perspective, imprecision, indirectness	citing effectiveness based on limited evidence, including a meta-analysis that found no clear, strong evidence of benefit and an artificial conditions laboratory study	**very low**

<u>Legend:</u> The rows of the equivalent of a GRADE Table (Grading of Recommendations Assessment, Development and Evaluation) contain the analysed outcomes. The last column represents the overall quality and certainty level.

Explanation of Each Column:

1) Outcome*: The specific outcome assessed.*

2) Study & year*: Leading author and year of publication.*

3) Number *of participants/individuals evaluated* ***(n):*** *n.a.= not applicable, *=n studies evaluated, **=counties evaluated (USA), ***=households in Germany (Berlin); ****= masks .*

4) Indicates the study type and the evidence level: *According to the evidence pyramid of EMB [1] with the level low (red, e.g. expert opinion, case report, case-control studies, surveys), moderate (yellow, e..g nRCTs, observational studies,cohort-studies,cross-sectional studies) and high (green, RCTs and systematic review with meta-analysis).*

RCT= randomised controlled trials, nRCT= non randomised controlled trial.

5) Funding and observer bias*: Reflects the overall assessment of funding bias and observer bias (high=red, medium=yellow, low=green) across included studies in partial accordance with the RoB and RoB-2, CASP-ratings.*

CASP=Critical Appraisal Skills Program. RoB= Risk of Bias.

6) Overall risk of bias*: Assesses the overall likelihood of bias of the studies included with consideration of columns 3), 4) and 5). This was assessed as low (green), moderate (yellow), or serious (red).*

7) Down- and upgrading of the evidence and reason*: Reflects the up- or downgrading of the evidence and the reasoning for, n.a.= not applicable,*

8) Reasoning for rating*: Specifications of the included studies, e.g. particular weaknesses or strengths.*

9) Final rating*: The final assessment of the evidence quality for each outcome.*

"High"= Further research is very unlikely to change confidence in the estimate of effect.

"Moderate"= Further research is likely to have an important impact on our confidence in the estimate of effect and may change the estimate.

"Low"= Further research is very likely to have an important impact on our confidence in the estimate of effect and is likely to change the estimate.

"Very Low"=Any estimate of effect is very uncertain.

[1] S.E.S.C.M.Ms. FRCPC, P.G.M.F. PhD, W.S.R. MD, R.B.H. MD, Evidence-Based Medicine: How to Practice and Teach EBM, 5th ed., Elsevier, Edinburgh ; London ; New York, 2018.

Table F: Summary of predominant evidence certainty of included studies **based on G**RADE equivalent evidence profile (Supplement A, Table E)

**Mask outcome**	**Predominant evidence quality**
**effectiveness against SARS-CoV-2**	**very low to low**<br>(4 very low, 2 low)
**non-effectiveness against SARS-CoV-2**	**high to moderate**<br>(4 high, 3 moderate, 3 low)<br><br>High-quality evidence from Jefferson 2023, Juutinen 2023, Sandlund 2023
**positive effects**	**very low to low**<br>(7 very low, 5 low)
**psychological and sociological effects**	**moderate to low**<br>(22 moderate, 18 low, 1 very low)
**physical symptoms, clinical conditions**	**moderate to high**<br>(7 moderate, 1high)
**physio-metabolic and toxicological effects**	**moderate to high**<br>(16 moderate, 8 high, 2 low)
empirically and experimentally unproven claims	**very low to low**<br>(5 very low, 1 low)
