# Supplementary material for: Risks and benefits of face masks in children
Source: Front Pediatr. 2026 Mar 13;14:1679586. doi: 10.3389/fped.2026.1679586 (PMC13047964; doi:10.3389/fped.2026.1679586)
Supplement: Supplementary file 2 — Data sheet 2: Supplementary B [file Datasheet2.pdf]

Supplement B

Risks and benefits of face masks in children

***Kai Kisielinski* [1,*]*, Claudia Steigleder-Schweiger* [2]*, Susanne Wagner* [3]*, Stephan Korupp* [4]*, Stefan Hockertz* [5]*, Oliver Hirsch*[6]**

[1] Clinical Medicine (Surgery), Emergency Medicine and Social Medicine, Private Practice, 40212 Düsseldorf, Germany.
[2] Department of Paediatrics, University Hospital of Salzburg, Paracelsus Medical University, 5020 Salzburg, Austria.
[3] Non-Clinical Expert, Veterinarian, Wagner MSL Management, 15831 Mahlow, Germany.
[4] Surgeon, Emergency Medicine, Private Practice, 52070 Aachen, Germany.
[5] Toxicology, Pharmacology, Immunology, tpi consult AG, Haldenstr. 1, CH 6340 Baar, Switzerland.
[6] Department of Psychology, FOM University of Applied Sciences, 57078 Siegen, Germany

*** Correspondence:** *kaikisielinski@yahoo.de*

Supplement B provides supplementary materials for the scoping review on the risks and benefits of face masks in children. Figure S1 illustrates the study selection process, while Tables S1–S6, as extraction tables of the included publications, complement the GRADE equivalent evidence profile in Supplement A.

See Supplement A for the detailed GRADE equivalent evidence profile of included studies.

Figure S1. Preferred Reporting Items for Systematic Reviews and Meta-Analyses (PRISMA) flow diagram of the scoping review.

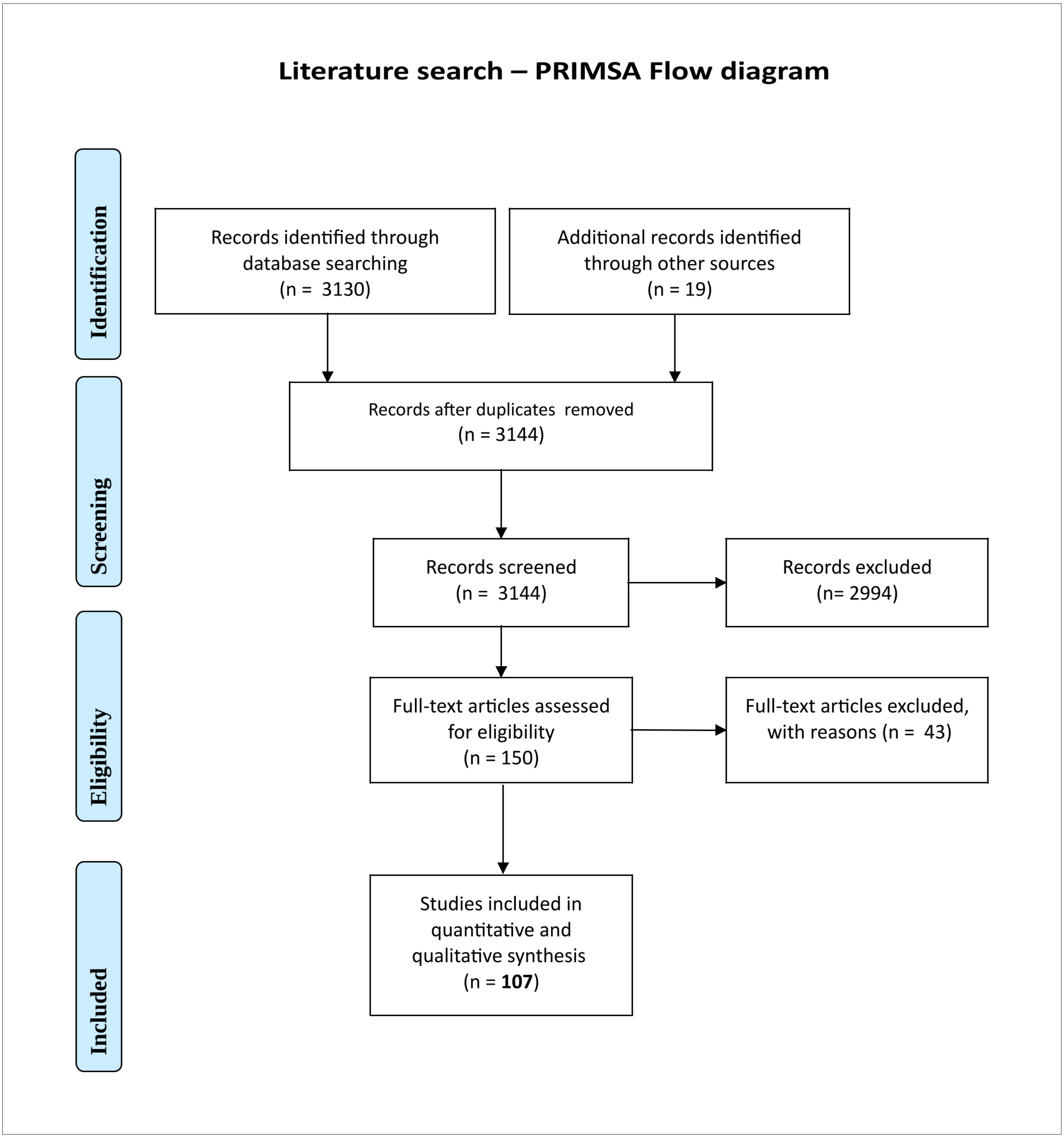

Table S1.
Included publications on evidence for (non-)effectiveness, summary of studies evaluating the protective efficacy of face masks against SARS-CoV-2 in children. Details of the studies with indicators of their quality.

Author and year	Type of study	Topic /Aim	Outcomes	Sample size	Main findings	Sponsor	Conclusions
**Bernhard et al. 2024**	Cross-sectional	To provide **descriptive data on infection risks as reflected by infection prevalence, on potential pathways and risk factors as well as on manifestation in young children.**	Seroprevalence data (reverse transcriptase–polymerase chain reaction), antibodies determined by Anti-SARS-CoV-2 test kits, electronic questionnaire.	108 children, thereof 16 pre-school children aged 2-6, 48 kindergarden children, 3-7 years and 44 school children 7-18 years old.	Undiagnosed infections were found in 12.5% of kindergarten children. 68.4 %, of them never wore a facemask. The self-reported neglect of wearing a facemask was associated with preceding infection.	Senate of Berlin	**Preceding infection was associated with never wearing a facemask, supporting a protective effect.** The high proportion of unrecognized infections in pre-school children needs to be considered when interpreting SARS-CoV-2 infections in the kindergarten context.
**Chandra & Høeg 2022**	Observational study	To build on an observational study by the Centers for Disease Control (CDC) that demonstrated a **link between school mask mandates and reduced paediatric COVID-19 cases,** it was **investigated whether this relationship holds in a larger, nationally representative dataset over an extended timeframe**.	**Relationship between school mask require-ments and paediatric cases of COVID-19.**	n=1832 counties	After nine weeks, the case rate per 100,000 people was 18.3 in counties with mask mandates, compared to 15.8 in counties without them (p = 0.12). In a larger sample of 1,832 counties, between weeks 2 and 9, the case rate per 100,000 people decreased by 38.2 in counties with mask mandates and by 37.9 in counties without them (p = 0.93).	None	In the extended sample, the link between school mask mandates and case numbers was not sustained. **Observational studies of interventions are subject to various biases and do not offer enough evidence to support the recommendation of mask mandates.**
**Coma et al. 2022**	Observational study	**To evaluate the effectiveness of**	Mask wearing vs no mask:	**n=599,314 children** aged 3 to	With **mandatory use of face masks**,	Ministerio de Ciencia, Innovación y	**Mask mandates in schools were not**

Author and year	Type of study	Topic /Aim	Outcomes	Sample size	Main findings	Sponsor	Conclusions
		**mandatory mask use in children aged six and above** with quasi-experimental comparison between 5 year-old children, as a control group, and 6 year-old children, as an interventional group.	incidence of SARS-CoV-2, secondary attack rates (SAR) and the effective reproductive number (R*).	11 years	the children have significantly **higher transmission indicators.**	Universidades and FEDER	**associated with lower SARS-CoV-2 incidence or transmission.**
**Høeg et al. 2023**	Review	To critically **examine the UnitedStates' decision to mask children** as young as two for COVID-19.	Evidence-based literature	Not applicable	Considering the best available high-quality evidence on mask effectiveness alongside potential harms, the **practice of masking children appears increasingly unfavourable**. An **increasing number of studies report adverse effects on children.**	None	With an **increasing number of high-quality studies showing no evidence of benefit,** justifying the CDC's mandate for masking children has become more challenging. From the outset, **the best available evidence has never supported a clear net benefit of masking very young children.**
**Jarnig et al. 2022**	Retrospective cohort study	To assess **if wearing face masks in the classroom is a useful mitigation measure to control SARS-CoV-2 infections.**	Comparison of cumulative SARS-CoV-2 infection rates in sports classes (with limited use of face masks) and non-sports classes (with the consequent use of face masks).	n=614 secondary school students (mean age: 15.1 ± 2.3 years, 43.2% female)	**Transmission can be reduced in school classes by mandatory FFP-2 mask use with only a small difference in cumulative SARS-CoV-2 infection rates. In many cases, however, infection appears to be**	Austrian Federal Ministry of Education, Science and Research as well as the University of Graz	**Infections with SARS-CoV-2 are delayed, but they cannot be prevented in the long run by wearing face masks. Mask use must be carefully balanced taking into account the potential negative consequences on psychosocial development and mental health.**

Author and year	Type of study	Topic /Aim	Outcomes	Sample size	Main findings	Sponsor	Conclusions
					**postponed rather than avoided.**		
**Jarnig et al. 2022**	Survey	To assess the **accuracy of face mask wearing by children and adolescents in different school situations**.	correct and incorrect wearing of face masks in classroom settings and school buildings in different situations in schools.	n=881 school students from middle school (mean 12.8 years) and high school (mean 16.7 years)	**Masks were worn correctly 63.7% of the time in the presence of a teacher. This percentage decreased to 31.9% when no teacher was present.** Students reported mouth and nose uncovered as being dominant, followed by mouth covered, nose uncovered variant.	Austrian Federal Ministry of Education, Science and Research as well as the University of Graz	**The results suggest the limited efficacy of mandatory face masks in schools. The advantage of reduced transmission must, however, be carefully balanced against the negative side effects of mandatory face mask use in schools.** Consideration should also be given to **alternative means of reducing the need to wear face masks** in classrooms. An option in this regard could be the effective ventilation and distribution of fresh air in the classroom, which has been shown to help reduce indoor virus transmission
**Jefferson et al. 2023**	Cochrane Systematic review	To assess the **effectiveness of physical interventions (including masks) to interrupt or reduce the spread of acute respiratory viruses**.	Numbers of cases of viral respiratory illness, adverse events related to the intervention.	3 studies of 43 included regarding masks and children	**The pooled results of RCTs did not show a clear reduction in respiratory viral infection with the use of medical/surgical masks.**	A. Pestryakov Development Program "Priority 2030", Tomsk Polytechnic University	**There is uncertainty about the effectiveness of face masks in the elderly and in young children. Harms associated with physical interventions were under-investigated.**
**Juutinen et al. 2023**	Observational study	To compare **COVID-19 incidence** among 10–12-year-olds between cities with	Mask wearing vs no mask: COVID-19 case numbers, defined as positive laboratory	14-day incidences per 100,000 inhabitants in a comparison among 1,103,025	**Highest incidence rates in winter months in the school mask mandated cities.**	None	**Face mask recommendations in schools did not reduce COVID-19 incidence among 10–**

Author and year	Type of study	Topic /Aim	Outcomes	Sample size	Main findings	Sponsor	Conclusions
		different **recommendations on the use of face masks in schools.**	verified SARS-CoV-2 test results, calculated cumulative incidences.	city inhabitants in Finland			**12-year-olds in Finland.**
**Ladhani 2022**	Review	To emphasise that **face masking for children should be reconsidered.**	Evidence for effectiveness of masks as an intervention to prevent infection and transmission of SARS-CoV-2 outside healthcare settings.	16 included publications	There are **no randomised controlled trials - the gold standard for determining the effectiveneess of facemasks to children**. Despite having the lowest risk of severe disease from SARS-CoV-2 infection, **children** have endured the **most dis-proportionate disruption to their lives during their most formative years.**	None	**There is no robust evidence to recommend face masks for children**. **We urgently need to return to first principles and focus on evidence-based interventions that help protect children.**
**Littlecott et al. 2024**	Systematic review, narrative synthesis	To provide an assessment of the evidence on the effectiveness of **measures implemented in the school setting** to keep schools open safely during the COVID-19 pandemic.	Measures to make contacts safer, including face masks, improved ventilation, cleaning, handwashing, or modifying activities.	15 studies	**Low-certainty evidence** showed that there **may have been a beneficial effect of mask mandates** on transmission-related outcomes.	German Federal Ministry of Education and Research, Bavarian Ministry of Research and Art, Bavarian Ministry of Science and the Arts.	**Further high-quality research into school measures is needed to develop a more evidence-based understanding**.
**Ludvigsson 2021**	Editorial	To discuss **evidence and harms of masks in children** against COVID-19.	Evidence and drawback	Not appilcable	There is **absence of evidence for masking children against COVID-19.** Studies that showed a	None	**No recommendation for masking children**, as **most of the children with COVID-19 get few symptoms and do not seem to drive the mortality** of the

Author and year	Type of study	Topic /Aim	Outcomes	Sample size	Main findings	Sponsor	Conclusions
					protective effect of face masks were more likely to be published than negative studies. Children are less affected by the virus. **Mask use reduces focus from other measures that may be more important**.		pandemic.
**Orey et al. 2023**	Cross-sectional	To assess the **COVID-19 seroprevalence** in children aged <18 years who visited a hospital in Somalia **as well as to evaluate other potential risk factors**.	COVID-19 Ag Rapid Test lateral flow immunoassay kit results, clinical characteristics, preventive practices questionnaire.	n=500 children (aged <18 y), 51.2% girls	6.4% children tested positive. 46.9% of the COVID-19–positive children were asymptomatic without any clinical signs of the disease. Children who did not wear a facemask were found to be twice as likely to be SARS-CoV-2 antibody positive.	None	**The majority of participants with positive antibodies against SARS-CoV-2 were school-aged children and children who do not wear facemasks, as well as those who had close contact with infected adults among others.**
**Sandlund et al. 2023**	Systematic review	**To evaluate the body of literature on mask wearing in children** to assess the existing evidence regarding protection offered by face masks against **SARS-CoV-2 infection or transmission.**	mask wearing vs no mask: CoV-2 infection or transmissions	22 studies included	**Real-world effectiveness** of child mask mandates against SARS-CoV-2 transmission or infection **has not been demonstrated with high-quality evidence**.	None	**The body of scientific data does not support masking children for protection against COVID-19.**
**Satapathy et al. 2024**	Observational	To ascertain the **prevalence of face**	Observed proper mask usage (cloth	n=320 children, aged 1 to 14 years	**33% of children did not wear a**	None	**Only a small proportion of children**

Author and year	Type of study	Topic /Aim	Outcomes	Sample size	Main findings	Sponsor	Conclusions
		**mask usage and the factors affecting the same.**	mask, surgical mask and N95 mask): wear, covering mouth and nose, not touching the mask, hand hygiene In case of.		**mask at all. Only 24.5% children used the mask appropriately.** Age, type of school, socio-economic status, maternal education, history of allergic diseases, COVID vaccination status of the parents, were significantly associated with the use of face masks.		**wore the mask appropriately. The effective mask usage is influenced by numerous factors** (age of children, type of school, and maternal education)**.** Age-appropriate targeted health interventions should be implemented to improve proper mask usage.
**Svetina & Kosec 2023**	Research-based commentary	To examine the **impact of non-pharmaceutical interventions (NPIs) on the resurgence of other respiratory viruse**s, particularly amid widespread vaccination fatigue, COVID-19 booster shots, and operational challenges within the healthcare system.	Not applicable	Not applicable	**Lockdowns** imposed during the pandemic in Europe, Australia, and New Zealand have **resulted in a sizeable group of susceptible young children who lack pre-existing immunity because of reduced exposure** during colder months.	None	**Prevention alone cannot serve as the sole solution for any infectious disease, and the lasting effectiveness of non-pharmaceutical interventions (NPIs) hinges on population susceptibility dynamics**. The COVID-19 pandemic has underscored the critical role of vaccination, and **integrating vaccine strategies with NPIs will be crucial** for managing both established and emerging respiratory pathogens.
**Viera 2024**	Systematic review (with narrative synthesis)	**To evaluate the effectiveness of face masks in reducing COVID-**	Reported effect direction for mask use versus no mask in school	14 studies included	Four **studies showed no difference or even worse results**	unknown	**More well-controlled and randomized studies are warranted to strengthen the**

Author and year	Type of study	Topic /Aim	Outcomes	Sample size	Main findings	Sponsor	Conclusions
		**19 incidence in school settings.**	settings for lowering COVID-19 Incidence		between mask and control, but **10 out of 14 included studies indicated** that **mask use could reduce COVID-19 incidence in schools**.		**evidence on the effectiveness of mask use for lowering the incidence of respiratory pandemic diseases in school settings.**

Table S2.
Included publications on positive effects of face masks in children (e.g., protective efficacy against non-SARS-CoV-2 hazards, psychological benefits). Details of the studies with indicators of their quality.

Author and year	Type of study	Topic /Aim	Outcomes	Sample size	Main findings	Sponsor	Conclusions
**Armero et al. 2024**	Observational	**To evaluate the effect of NPI measures (hand washing, mandatory use of face masks for children older than 5 years, social distancing) and their influence on the circulation of respiratory viruses among children** before and during the SARS-CoV-2 pandemic (January 2017 to December 2022).	Respiratory virus tests	n=2991 tests in patients aged 0-18 years residing in Catalonia	**Requiring facemasks for children aged ≥6 years resulted in reduced viral circulation**; however, a resurgence of influenza virus following the removal of facemasks was noted.	None.	A **notable effectiveness of NPIs (hand washing, mandatory use of face masks for children older than 5 years, social distancing) in lowering respiratory virus transmission among children is shown,** even when not specifically targeted at preschool-aged children. **Rhinovirus, RSV, HMPV, and influenza show particularly favorable responses to these intervention**s.
**Banholzer et al. 2023**	Observational study with mofelling	This study aimed to evaluate the **transmission of SARS-CoV-2 in schools and their association with infection control measures** using a multiple-measurement approach.	SARS-CoV-2 in aerosols in the air and saliva samples from the students	90 students in 2 secondary schools	**Aerosol and particle concentrations were reduced by an average of 70% with mask mandates and 40% with air cleaners.**	Multidisciplinary Center for Infectious Diseases, University of Bern, Bern, Switzerland. National Institute of Allergy and Infectious Diseases (NIAID), Swiss National Science Foundation	Molecular detection of airborne and human SARS-CoV-2 suggested ongoing transmission in schools. **Mask mandates were linked to more significant reductions in aerosol concentrations and lower transmission rates compared to air cleaners.**
**Holm et al. 2021**	Review	**To summarise current knowledge** in the health effects of **wildfire smoke** in children as well as	Wildfire smoke exposition protection (ultrafine particles as part of the PM2.5 or PM10 fractions)	34 studies included (mask topic)	Mask helped to lessen symptoms with exposure. **N95 masks are able to decrease outdoor wildfire**	Agency for Toxic Substances and Disease Registry (ATSDR), US Environmental Protection Agency	**Studies generally support the idea that children could see benefits from respirators in wildfire smoke scenarios** as

Author and year	Type of study	Topic /Aim	Outcomes	Sample size	Main findings	Sponsor	Conclusions
		**tools for public health response aimed at children including mask**s			**exposure by ~80% and surgical masks by ~20%.** These benefits would only be expected for short durations.	(EPA)	well, despite the concern that fit may be difficult due to more variation in facial sizes. **False sense of security, possible adverse physiologic effects have to be considered. A research gap regarding safety exists.**
**Janapatla et al. 2021**	Observational	To analyse the monthly **incidence rates of invasive pneumococcal disease (IPD)** and the distribution of serotypes **in high-risk age groups** and settings with high pneumococcal transmission from January 2020 to February 2021 and **to consider nonpharmaceutical interventions (NPIs)** interventions like facial masking, enhanced hand hygiene, social distancing, international travel controls, effective quarantine and contact-tracing policy	Incidence rates of invasive pneumococcal disease (IPD)	Not specified. Incidence rates are discussed.	**Significant reduction in** both vaccine and non-vaccine serotype **IPD incidence rates among children after the implementation of NPIs** to control COVID-19.	Ministry of Science and Technology, Taiwan, Chang Gung Memorial Hospital, Taiwan	**The significant decrease in invasive pneumococcal disease (IPD) incidence rates** for both vaccine and non-vaccine serotypes **supports the ongoing use of fundamental NPIs—such as wearing face masks, improved hand hygiene, and social distancing**—especially in settings with a high risk of transmission, to reduce respiratory tract infections caused by both viruses and bacteria, including IPD.
**Matsuda et al. 2023**	Survey	**To evaluate the impact of non-pharmaceutical interventions (NPIs) on**	Proportion of hand washing and face mask wearing.	n= 13206 Preseason; n=10939 COVID-19 season	During the COVID-19 season, 48.8% of vaccinated children practiced hand	Warabi-Toda Medical Association, Teikyo University, Japan Foundation for	The findings of this study could contribute to the development of **activities (hand washing and face**

Author and year	Type of study	Topic /Aim	Outcomes	Sample size	Main findings	Sponsor	Conclusions
		**behavior and influenza infection among children in a metropolitan area** of Tokyo, Japan, across two periods: the 2018–2019 season (pre-COVID-19) and the 2020–2021 season (during the COVID-19 pandemic).			washing and wore face masks, a notable increase from 18.2% in the Preseason**. Among children who simultaneously washed their hands and wore face masks, there was a significant reduction in influenza infections.**	Paediatric Research, Ministry of Education, Culture, Sports, Science and Technology, Japan	**masks) or preventive measures to protect children from influenza** and potentially other communicable diseases.
**Qin et al. 2021**	Internet Survey	To **understand the self-reported psychological distress status among school-aged children and adolescents** during the **COVID-19 pandemic** and to identify the **risk and protective factors** associated.	Electronic questionnaire through an online survey including general health questionnaire (GHQ-12), exercise time, COVID-19 information sources, and frequency of mask use.	n=1,199,320 children, mean age 12 years; 51.6% boys	**10.5% reported psychological distress**, among whom 51.5% were girls. Compared with students who always wore face masks, **students who rarely wore face masks had significant higher odds of self-reported psychological distress**.	Central Committee of the Communist Youth League, Special Research Project of Prevention and Control during COVID-19 Epidemic in Universities of Guangdong, Medical Scientific Research Foundation in Guangdong, National Nature Science Foundation of China	**The prevalence of psychological distress among school-aged children and adolescents during the COVID-19 pandemic was relatively high. The frequency of wearing a face mask and time spent exercising had protective associations for mental health.** It is necessary to pay attention to these facts and take appropriate countermeasures.
**Science et al. 2022**	Randomized Clinical Trial	To evaluate the **effect of wearing a face mask on hand-to-face contact by children while in a simulated school attendance.**	Number of hand-to-face contacts per student per hour and hand-to-mucosa contacts and hand-to-nonmucosa contacts.	n=171 children aged 5 to 18 years, (students)	The **rate of hand-to-face contacts did not differ significantly between the mask and the control groups**. When compared with the control group, **the rate of hand-to-mucosa contacts**	Hospital for Sick Children Foundation	As in a simulated school attendance, hand-to-face contacts did not differ among students required to wear face masks vs students not required to wear face masks and hand-to-mucosa contracts were lower in the face mask group, while the hand to non-

Author and year	Type of study	Topic /Aim	Outcomes	Sample size	Main findings	Sponsor	Conclusions
					**was significantly lower** in the mask group **while the rate of hand-to-non-mucosa contacts was higher.**		mucosa contacts was higher, **it was concluded that mask wearing is unlikely to increase infection risk through self-inoculation (mucosa).**
**Sombetzki et al. 2021**	Prospective observational study with modeling	To monitor **dynamics of COVID-19 infections in schools and preschools and identify factors influencing the extent of outbreaks**	Number of secondary cases tested PCR positive for SARS-CoV-2.	n=475 school related infections from calendar week (CW) 32 in 2020 to CW 19 in 2021 at general and private schools and pre-schools	The **teacher/ caregiver mask obligation and the face mask obligation for children showed a significant reduction in the number of secondary cases with small effect sizes.**	Ministry of Economic Affairs, Labor and Health Mecklenburg-Western Pomerania	**Requiring both adults and children to wear face masks effectively contains outbreak events in schools and preschools.**
**Suess et al. 2011**	cluster-randomized trial	To monitor **adherence and tolerability of face masks and intensified hand hygiene to prevent influenza infections in households**.	Frequency of face mask wearing, frequency of hand disinfection	147 participants in 41 households, 39 (95%) out of 41 index patients were children (aged <14 years)	In households instructed to wear facemasks, **usage peaked on day 4 after the index patient's symptom onset, reaching 73% for children and 65% for adults**.	None	**Children accepted wearing masks—even when ill—at a rate similar to that of adults.**
**Suess et al. 2012**	Cluster randomized controlled trial	To investigat**e efficacy, acceptability, and tolerability of face masks and hand hygiene in households with influenza** index patients	qRT-PCR confirmed influenza infection	n=84 households including children	Analysis of **households that fully implemented the intervention within 36 hours of symptom onset** showed that participants **had a significantly lower risk of influenza infection.** The primary	German Federal Ministry of Health	**Non-pharmaceutical interventions may be effective in preventing influenza transmission in households,** provided the intervention is implemented early and adherence is high. The findings also indicate that the use of facemasks is both tolerable and acceptable for adults and children, whether they

Author and year	Type of study	Topic /Aim	Outcomes	Sample size	Main findings	Sponsor	Conclusions
					problem associated with wearing a facemask cited by both adults and children was "**heat/humidity**" (53% of children and 35% of adults) (p = 0.1), followed by "**pain**" and "**shortness of breath**".		are household contacts or index cases.
**Theuring et al. 2021**	Cross-sectional analysis of a longitudinal study	To measure **SARS-CoV-2 infections and seroreactivity in 24 randomly selected school classes and** connected households in Berlin, Germany and to assess individual and institutional prevention measures.	SARS-CoV-2 infections and seroreactivity.	1,119 participants in 24 schools including 177 primary and 175 secondary school students, 142 staff and 625 household members	**Prevalence increased with inconsistent facemask-use in school. Mask wearing was recorded by self-reporting.**	Senate of Berlin, Germany	**Schooling itself does not necessarily result in child-to-child transmission or serve as a primary driver of the COVID-19 pandemic, as long as infection prevention and control (IPC) measures are strictly enforced.**
**Villers et al. 2022**	Modeling study	An existing **aerosol transmission model** was modified to investigate the impact of various interventions (natural ventilation, face masks, high efficiency particulate air (HEPA) filtration, and their combinations) on	cumulative dose of viruses absorbed by exposed occupants	One infectious individual	**Surgical face masks showed significant effectiveness regardless of the season,** with an 8-fold decrease in transmission. **Combined interventions (such as natural ventilation, masks, and HEPA filtration) were the most effective**, resulting in a 25-	Swiss National Science Foundation	**In the aerosol transmission model ventilating naturally, using face masks, and employing HEPA filtration are effective strategies to decrease SARS-CoV-2 aerosol transmission**. These measures should be integrated and supported by additional interventions such as physical distancing, hygiene practices, testing, contact tracing,

Author and year	Type of study	Topic /Aim	Outcomes	Sample size	Main findings	Sponsor	Conclusions
		the concentration of virus particles in a 160 m³ classroom with one infectious individual.			fold decrease and maintaining high effectiveness even in the presence of a super-spreader.		and vaccination to maximize their effectiveness.
**Xiao et al. 2021**	Cross-sectional	**To evaluate the additional benefits of non-pharmaceutical interventions (NPIs:** border restrictions, quarantine and isolation, community management, social distancing, face mask usage, and personal hygiene) against COVID-19 on notifiable infectious diseases (NIDs).	Cases of notifiable infectious diseases (NIDs) except COVID-19	514,341 cases of 39 types of notifiable infectious diseases (NIDs)	**The largest reduction (82.1%) was found for children aged 0–14 years.**	Key- Area Research and Development Program of Guangdong Province; Chinese Postdoctoral Science Foundation; the Science and Technology Pro-gram of Guangdong Province; Foshan Key Technology Project for COVID-19	**Non-pharmaceutical interventions (NPIs) aimed at COVID-19 prevention may significantly benefit the prevention of other infectious diseases** in Guangdong, China, with effects varying across different populations, diseases, and geographic and temporal contexts.

Table S3.
Included publications on psychological and sociological effects evaluating the psychological and sociological impacts of face mask use in children (positive and negative outcomes). Details of the studies with indicators of their quality.

Author and year	Type of study	Topic /Aim	Outcomes	Sample size	Main findings	Sponsor	Conclusions
**Ammann et al. 2022**	Nested online surveys between 12 January and 24 March 2021 (Q1) and 10 March and 16 May 2021 (Q2).	**Perceptions of the usefulness of masks in schools and public places among a cohort of children and adolescents** in the canton of Zurich, Switzerland, from January to May 2021	Questions on the **children's perception of the usefulness of masks and mask wearing behaviour.**	n=595 school children at Q1, n=1118 at Q2	**>50% of the school children considered masks to be useful.** Girls more frequently found masks useful compared to boys. **About 20% of children reported side effects and discomfort, such as skin irritations, headaches, or difficulties breathing during physical education.**	Swiss Federal Office of Public Health and private funders, University of Zurich Foundation	**Around 60% of school children found masks to be useful in school and public places**. However, **a small number of children reported discomfort and side effects**, which should be addressed to ensure high compliance with mask-wearing among school children.
**Aronu et al. 2020**	Cross-sectional interview study	To determine **mothers' perceptions of masking young children** as a preventive strategy for COVID-19.	Wearing face masks in children.	n=387 mothers	**A minority (44.7%) of mothers viewed masking children as an appropriate COVID-19 prevention measure. The majority (55.3%) cited reasons such as perceived breathing difficulties (38.5%) and the likelihood of the child pulling off the mask (29.3%) as reasons for considering face masks inappropriate for**	None	**Maternal perception of masking in children as an effective COVID-19 prevention strategy is low.** However, the correct perception is significantly influenced by the mother's educational level, employment status, and marital status. **The use of face masks for children under the age of two years should be discouraged.**

Author and year	Type of study	Topic /Aim	Outcomes	Sample size	Main findings	Sponsor	Conclusions
					**children.**		
**Bourke et al. 2023**	Observational	To evaluate the **effect of facemasks on children's language and emotion recognition**.	Computer-based tasks with adults wearing or not wearing surgical face masks to assess language processing skills and emotion recognition ability.	n=74 children, aged 4–8 years	**Masked angry faces more easily recognized and masked happy and sad faces less easily recognized.** Younger children were less accurate than older children.	Summer Internship Scheme and Department of Psychology Research Committee at Liverpool Hope University	**Face masks did not significantly impair basic language processing ability.** However, they **had a significant effect on the children's emotion recognition accuracy.**
**Carnevali et al. 2022**	Systematic review	**To examine behavioural studies investigating face processing in early infancy and to generate hypotheses about the effects of mask wearing on infant social development.**	Face processing parameters	129 studies	**For perceptual skills, infants progressively learn to interpret the eyes or gaze direction within the context of the entire facial configuration.** This process aids in identity recognition and emotional expression discrimination. In terms of socio-communicative skills, direct gaze and emotional facial expressions are vital for engaging attention, while eye-gaze cuing is crucial for joint attention. Additionally, focusing on the mouth is particularly important for speech learning.	Beneficentia Stiftung Foundation, MRC Programme	**The potential implications of exposure to masked faces for developmental needs and functions are discussed, and there is an encouragement for further investigation into the effects of mask-wearing on infants' perceptual and socio-communicative development.**
**Charney et al. 2021**	Commentary	To discuss **how preventative**	Not applicable	Not applicable	**Masks are known to degrade the**	None	**Mask wearing may negatively affect**

Author and year	Type of study	Topic /Aim	Outcomes	Sample size	Main findings	Sponsor	Conclusions
		**practices may negatively affect communication**.			**speech signal, functioning as a low-pass filter by reducing high-frequency sounds spoken by the wearer**. The attenuation ranges from 3 to 4 dB for simple medical masks and nearly 12 dB for N95 masks. For children with hearing loss, this seemingly minor change can significantly impact their speech comprehension compared to their peers with normal hearing. Masks can also hide social cues conveyed through facial expressions.		**communication.** While more research is needed to fully understand the pandemic's impact on paediatric speech and language development, **clinicians and parents should be aware of this issue and take steps to create an optimal communication environment for children.**
**Chester et al. 2022**	Cross-sectional with longitudinal subsample	To examine **how face masks can impact emotion recognition.**	Dynamic Affect Recognition Task (DART)	n=131 (7-12 years), n=35 pre-pandemic subsample	Covering faces with masks was associated with **poorer recognition of emotions, particularly for happy, sad, and fearful expressions compared to angry and neutral ones.** In the pre-pandemic subset, recognition of sad faces was lower during the	Institutional support from the University of Pennsylvania and Boston University, a University of Pennsylvania MindCORE Postdoctoral Fellowship, funding from the John and Polly Sparks Foundation (American Psychological Foundation), and funding from the	**Covering parts of the face and the broader social context (i.e., the global pandemic) both influence emotion-related judgments in school-aged children.**

Author and year	Type of study	Topic /Aim	Outcomes	Sample size	Main findings	Sponsor	Conclusions
					pandemic compared to before, relative to other emotions.	National Institute of Mental Health	
**Chhabra et al. 2019**	Case control	**To determine the psychophysiological response (heart rate)** of individuals with special health care needs to the use of a facemask-eyeshield and visor in a dental setting.	Heart rate	n=378 children, age 4-14 years, IQ range 50-84	The **mean heart rate** during dental examinations **while wearing a facemask and eye shield was significantly higher than wearing a visor.**	None	**Anxiety levels were higher when the dentist used a facemask and eye shield compared to when a visor during the examination was used.**
**Coelho et al. 2022**	Cross-sectional study	To explore **children's views** on returning to in-person school after pandemic lockdowns, their **opinions on wearing masks in class,** and the **mental health of both children and parents** during the pandemic.	Anxiety related to COVID-19 and children's mental health during the pandemic were evaluated using questions from the international CRISIS questionnaire.	n=190 children	Older students were more likely than younger students to report that **wearing masks hindered their ability to interact with peers and understand the teacher.** Additionally, students not required to wear masks were more likely to express **concerns about contracting COVID-19** at school and anticipated difficulties with wearing a mask.	SickKids Foundation, Ministry of Health/Ontario Together COVID-19 Rapid Research Fund, and Miner's Lamp Innovation Fund in Prevention and Early Detection of Severe Mental Illness at the University of Toronto	The findings highlight the **need to implement teaching and communication strategies that do not depend on facial expressions** in the classroom and at school. Additionally, it is **important to consider other public health measures that do not obscure facial expressions,** such as using transparent face shields or maintaining social distancing during instruction.
**Drössler et al. 2023**	Qualitative study with expert interviews	To **investigate the effects of mask-wearing during the COVID-19 pandemic on the well-being,**	Thematic analysis of verbal material	Structured interviews were conducted with various experts, including educators (n = 2),	The most frequently reported **short- and medium-term direct effects of mask-wearing**	Bundeszentrale für gesundheitliche Aufklärung	**While the effects of mask-wearing on communication and interaction for children and adolescents are relatively well-**

Author and year	Type of study	Topic /Aim	Outcomes	Sample size	Main findings	Sponsor	Conclusions
		**behaviour, and psychosocial development of children and adolescents**.		primary and secondary school teachers (n = 9), youth representatives (n = 5), primary care physicians (n = 3), and a public health service physician (n = 1).	include **reduced communication due to diminished auditory comprehension and facial expression visibility. These communication limitations impact social interaction and the quality of education**. There is an **increase in psychosomatic complaints, anxiety, depression, and eating disorders.**		**documented, the impacts on other aspects of psychosocial development are (still) not clearly identifiable.**
**Flaherty et al. 2023**	Observational	To explore the **effects of different face masks on school-age** children's and young adults' **word recognition.**	Word recognition (speech-in-speech recognition)	30 children (8–12 years), 25 adults (18–25 years)	Both children's and adults' word recognition was most negatively impacted by the face shield. Children's recognition was also impaired by the transparent mask. No negative effects were observed for the N95 or surgical mask for either age group.	Department of Speech and Hearing Sciences at University of Illinois Urbana-Champaign	**School-age children, and young adults, are negatively affected by face masks when recognising speech**, but the effects depend on the type of face mask being worn.
**Freiberg et al. 2021**	Systematic review	To evaluate **mask-wearing on the psychosocial development of children and adolescents** in the context of	Psychological development, language development, socio-emotional development, social behavior, school	13 studies	**Children and their teachers experienced difficulties in processing facial expressions due to mask-wearing,**	This study was funded by the Federal Center for Health Education.	**The limited number of studies provides only scant evidence regarding the impact of wearing mouth-nose protection on various developmental aspects**

Author and year	Type of study	Topic /Aim	Outcomes	Sample size	Main findings	Sponsor	Conclusions
		infectious disease prevention.	success, participation.		**Anxiety and stress, along with concentration and learning challenges** was associated with mask use.		**of children and adolescents.** Specifically, there is a shortage of research data on psychological development, language development, emotional development, social behavior, academic success, and participation.
**Giordano et al. 2024**	Observational	To examine **young children's ability to identify emotions** (happy, sad, angry, scared) **in an adult wearing a face mask**.	multiple-choice items	n=77 preschool children's	Children who were older, had more exposure to adults wearing masks, and attending group care were better able to identify the emotions	None	**Children were better able to identify emotions in an unmasked adult** and when the masked model explicitly stated or implied the emotion. In masked adults there is need to explain emotions by providing verbal cues stating or implying the emotion.
**Ger et al. 2023**	Observational	To investigate **whether facial masks** that cover adult faces **affect 4- to 6-year-old children's recognition of emotions** and the **duration of children's exposure to masks** is associated with **emotion recognition**.	Face stimuli from the Karolinska Directed Emotional Faces – KDEF, webcam-based eye-tracking functionality, weekly hours of mask exposure	n=79 children, 4-5 years	**Masks impair the recognition of sadness and happiness, also of anger, which was, however, somewhat easier to recognise.** The longer hours of mask exposure were associated with better emotion recognition.	Jacobs Foundation, Coordenação de Aperfeiçoamento de Pessoal de Nível Superior	**Restricting facial cues due to masks impairs kindergarten children's emotion recognition, but in the long run a compensation occurs, facilitating their broader reading of facial emotional cues.**
**Gil et al. 2023**	Observational	To explore **whether first- and fifth-graders and young adults were influenced by the**	Results of an emotional facial expressions recognition task in a physical laboratory	n=54, thereof 27 children (13 females) in first grade (mean age 6.5), 27 children	The **presence of a mask did affect the recognition of sad or fearful faces but did not**	None	**the presence of a mask has a heterogeneous impact depending on the nature of the emotion, affecting fear**

Author and year	Type of study	Topic /Aim	Outcomes	Sample size	Main findings	Sponsor	Conclusions
		**absence or presence (and color: pink, green, red, black, or white) of a face mask when asked to judge emotional facial expressions** of fear, anger, sadness, or neutrality.	setting with respond on a four 9-point Osgood-type scale (semantic differential scale).	(14 females) in fifth grade (mean age 10.6).	**influence significantly the perception of angry and neutral faces.** Mask colour slightly modulated the recognition of facial emotional expressions, without a systematic pattern.		**and sadness recognition.**
**Gori et al. 2021**	Observational	To demonstrate **that face masks influence the human ability to infer emotions by observing facial configurations**.	Internet-based questionnaire: standardized verbal-response test based on selecting an emotion's label (forced-choice) to describe static pictures of human facial configurations.	n=80 children, thereof 31 toddlers from 3 to 5 years and 49 children from 6 to 8 years of age	The main insight of the present research is that **face masks' use influences emotion inference from faces for all ages and especially for toddlers**. The capacity to read emotions when a face mask is present becomes particularly reduced in toddlers.	None	**Face masks may potentially affect the emotional understanding or developing social and interaction skills in children, such as in education, especially for those suffering from sensory or cognitive deficits.**
**Halbur et al. 2021**	Observational	To **evaluate a treatment package on tolerance of wearing a face covering for up to 15 min for 12 children with autism spectrum disorder (ASD).**	Tolerance to steps of the exposure hierarchy for the face mask and time of treatment.	n=10 children with ASD, 4-10 years old, all male	The mean durations of treatment for participants were 68 min. A treatment package composed of **graduated exposure, prompts, and differential reinforcement produced tolerance** of the application and extended use of	None	**The tolerance of face coverings treatment package was successful, with minor modifications, for children with ASD of multiple ages.** Research also is needed to determine the prevalence of touching and licking face coverings in children with ASD who tolerate their use.

Author and year	Type of study	Topic /Aim	Outcomes	Sample size	Main findings	Sponsor	Conclusions
					face coverings for nine children with ASD.		
**Hahn et al. 2023**	Cohort study	Is there an **association between government mask mandates and mask usage** among children in Alberta, Canada?	parent report of child mask use.	939 children (age, 8-13 years)	Parents were 18.3 times more likely to report that their **children often or always wore masks when a mask mandate was in place c**ompared to when it was not. Conversely, each day without the mask mandate saw a 1.6% decline in mask use.	Government of Alberta and the Women and Children'sHealth Research Institute	The **use of masks among children, as reported by parents, tends to increase when the government enforces mask mandates** and provides current health information (such as case counts). Conversely, **longer periods without a mask mandate are linked to a decline in mask usage.**
**Kwon et al. 2022**	Cross-sectional	To explore **how preschoolers (ages 4-6) perceive wearing masks.**	Interview questions regarding mask-wearing perception.	n=74 preschoolers (ages 4-6).	The majority of children indicated they were comfortable wearing masks at preschool. **Caregivers' perceptions of masks in Korea seemed to influence children's attitudes.** Younger children tended to see masks as part of their physical selves, whereas older children did not.	Basic Science Research Program of the National Research Foundation (NRF), funded by the Ministry of Education, Republic of Korea and also funded with research funds provided by Gwangju University.	**Preschoolers understood the importance of wearing a mask for their own and others' protection from the coronavirus, viewing it as necessary and positive.** The majority of children reported feeling comfortable wearing a mask throughout the entire preschool day.
**Kwon et al. 2023**	Observational	To investigate the **combined effects of face-mask usage** (no mask, surgical	Auralisation technique, speech recognition tests.	n=67 children (4–6 years old)	The **face mask and reverberation time (RT) affected pre-schoolers' speech recognition**	National Research Foundation (NRF), Ministry of Education, Republic of Korea,	**The face mask negatively affected pre-schoolers' speech recognition in a realistic classroom**

Author and year	Type of study	Topic /Aim	Outcomes	Sample size	Main findings	Sponsor	Conclusions
		and KF94 masks) **and room acoustics** on **speech recognition** in **preschool children**.			**scores.** Reducing RT in the class-room improved the pre-schoolers' **speech recognition that was reduced by face masks.** Children aged 4 and 5 years were affected by face masks and RT more significantly than those aged 6 years.	Gwangju University.	**environment,** which included reverberance and noise. Improvements for better speech recognition in preschools, which usually facilitate **children's language-and-speech development** are needed.
**Lalonde et al. 2022**	Experimental simulation study	**To examine the effects of four types of face masks** (hospital, fabric, and two transparent masks) **on auditory-only and audiovisual speech recognition**.	Auditory identification tasks	18 children with bilateral hearing loss, 16 children with normal hearing (aged 7 to 18 years), and 38 adults with normal hearing tested in their homes, 15 adults with normal hearing tested in the laboratory	**The presence of face masks affected all groups in a similar manner.** The greatest effects on place perception were due to **high-frequency attenuation and visual obstruction**.	National Institutes of Health	**The combination of noise and face masks adversely affects children's ability to understand speech**.
**Le et al. 2023**	Cross-sectional survey	**To compare the health beliefs of children and their caregivers and assessed the factors influencing children's use of masks to reduce exposure to air pollution.**	Health Belief Model (HBM)-based questionnaire	n=8420 children aged 13–14 and their caregivers.	Only **children were notably concerned about how the public perceived their appearance while wearing a mask.** Females were more likely to wear masks, and caregivers with higher education levels were more inclined to encourage mask-wearing.	Vietnam National Foundation for Science and Technology Development, National Health and Medical Research Council	**Children and their caregivers have differing views on wearing masks to protect against air pollution. Children are more sensitive to social judgments about their appearance when wearing a mask.**

Author and year	Type of study	Topic /Aim	Outcomes	Sample size	Main findings	Sponsor	Conclusions
**Liu et al. 2024**	Observational	To explore the **effect of masks on infants' abilities to fast-map and generalize new words.** All included infants were exposed to masks on a daily basis since birth.	Four familiar and four novel objects as stimuli and eye-tracking, fixation time word test trials	n=72 Chinese children, 17-18 months old, 43 boys	No significant difference in fixation time, but a tendency to look more to the with mask condition. The proportion of looking time to the eyes was larger than that of the mouth. Infants looked significantly longer at the eyes and at the mouth than at the hand in the no mask condition,	Not mentioned	**18-month-olds can fast-map new words and generalize these words across people and objects of the same category when words were taught with or without a mask.** However, infants can flexibly **rely more on the eyes** to achieve fast-mapping and word generalization when the speaker is masked. All included infants were exposed to masks on a daily basis since birth.
**Mickells et al. 2021**	online, prospective, observational, survey	**To evaluate the use of cloth face masks among children in pre-kindergarten** (pre-K) through second grade, identifying related characteristics and adverse events.	Percent of the day that the entire class was wearing their masks appropriately.	Not explicitly mentioned.	Out of nearly 1,000 students and 1,048 classroom days **reported, the average percentage of the school day with proper mask usage was 76.9%**.	Wilbur and Hilda Glenn Family Foundation, Paediatric Biostatistics Core, Emory University and Children's Healthcare of Atlanta.	**For most of the day during in-person instruction, children in grades pre-K through 2 can consistently wear masks,** serving as a key strategy to limit the spread of SARS-CoV-2 and its potential future use.
**Mitsven et al. 2022**	Longitudinal cohort study. The first group (Cohort 1) was observed before the onset of the COVID-19 pandemic, between October 2019 and February 2020. The second group (Cohort 2) was observed in their classroom during the	**To assess the impact of face masks examining speech-related vocalizations of children and teachers in two cohorts** of 3.5–4.5-year-old children enrolled in the same oral language classroom, which included both	Duration and rate of vocalizations; phonemic diversity of vocalizations.	Cohort 1: n=20, cohort 2: n=15	**During COVID-19, teachers spoke more words per minute than those observed before the pandemic, but their vocalizations included fewer unique phonemes.** Children observed during COVID-19 showed no deficits in the duration,	Grants from the National Science Foundation, the Institute for Education Sciences, and the National Institutes of Health, USA	**Children's language production in the classroom is largely unaffected by mask use**, and they continue to benefit from the language exposure even when teachers are wearing masks

Author and year	Type of study	Topic /Aim	Outcomes	Sample size	Main findings	Sponsor	Conclusions
	COVID-19 pandemic, when mask-wearing was mandatory for both teachers and children.	children with and without hearing loss.			rate, or phonemic diversity of their vocalizations compared to those observed before the pandemic. **In fact, children during COVID-19 produced longer vocalizations**. Additionally, during the pandemic (but not before), children exposed to a higher number of words per minute from teachers produced more speech-related vocalizations themselves.		
**Miyazaki et al 2023**	Observational	**To determine the negative influence of mask-wearing on reading emotions of adult faces by Japanese school-aged children**. We also examined **whether this negative influence could be alleviated by using a transparent face mask i**nstead of an opaque one (surgical mask).	Emotion categorization and emotion intensity rating tasks .	n=282 children (141 girls, 138 boyos, and three did not specify their sex; 9–12 years9	The accuracy of **emotion recognition in children was impaired for various facial expressions (disgust, fear, happy, neutral, sad, and surprise faces), except for angry faces.** A negative influence of wearing surgical masks was generally not observed for faces wearing a transparent mask in	Unicharm Corporation.	**Negative side effects of mask-wearing on reading emotions are observed for more facial expressions in children than in adults; transparent masks can help remedy these.**

Author and year	Type of study	Topic /Aim	Outcomes	Sample size	Main findings	Sponsor	Conclusions
					both children and adults.		
**Nobrega et al. 2020**	Letter to the editor	To state **how face masks can affect school performance**.	Acoustic parameters	Not appilcable	Negative aspects of face masks: **The child cannot access visual cues because the speaker's face is hidden, preventing lip reading. The teacher's voice is diminished and distorted.**	None	**Recommendations to parents and teachers are given, e.g. using portable microphones or repeating instructions.**
**Omaleki et al. 2024**	Mixed-methods study	To investigate **attitudes towards masking by focusing on the perspectives of parents and children** at historically marginalized, predominantly Hispanic schools in Southern California.	Freelist of words associated with masking; parent-child interviews (PCI).	648 participants provided 1118 unique freelist items in English and Spanish. 19 parent-child pairs were interviewed	The most prominent words were “safety”, “protection”, “prevention”, “health”, “good”, **“can’t breathe”,** “necessary”, “care”, “precaution**”**, **“uncomfortable”** and **“unnecessary”.** Spanish speakers had a more positive view of masking compared to English speakers, especially in terms of “protection” and “prevention”.	National Institute of Mental Health, National Institute of Child Health & Human Development, National Institute of Allergy and Infectious Diseases, San Diego County HHSA.	**It is recommended that policymakers prioritize the perspectives of those most impacted when making decisions about risk mitigation policies, such as school masking.**
**Preest et al. 2024**	Systematic review and narrative synthesis	**To evaluate children's perceptions and experiences of wearing masks**, based on existing	Different parameters of children´s experiences of mask-wearing.	45 studies of children 4 to 14 years	Children's attitudes towards wearing masks were shaped by their perceived importance, social norms, and parental	None.	**There is a clear necessity for manufacturers to enhance available masks for children, possibly by offering**

Author and year	Type of study	Topic /Aim	Outcomes	Sample size	Main findings	Sponsor	Conclusions
		evidence that examines mask use in various contexts such as air pollution and disease prevention.			attitudes. They faced **various challenges, such as difficulty interpreting facial expressions and physical discomfor**t. **Children frequently express physical discomfort associated with wearing masks,** particularly due to **poor fit and heat.**		**more size options or adjustable features that allow children to customize the fit.** Guidelines should include strategies to improve children's overall experience with mask-wearing.
**Schlegtendal et al. 2022**	Randomised controlled trial	To analyse the **influence of face masks on cognitive performance of pupils** during two regular school lessons.	Digital computer-based test tool for cognitive performance regarding attention and executive functions in masked and unmasked children that continuos have worn mask during the pandemic.	n=133 children (11 to 14 years)	**No significant differences in cognitive performance between both groups.** Tendencies regarding a slightly worse cognitive performance in the group of mask wearers found.	None	**Wearing face masks has no significant influence on attention and executive functions of pupil**s during two school lessons.
**Schwarz et al. 2022**	Observational	To investigate the **acoustic and visual effects of face masks on speech intelligibility** and processing speed under varying semantic predictability **in classroom settings.**	internet-based cued shadowing task	n=26 children (aged 8-12), 26 adults	**Children** and adults **made more mistakes and responded more slowly when listening to face mask speech** compared to speech produced without a face mask.	Cambridge Language Sciences Incubator Fund	**In classroom settings,** strategies that increase **contextual information** such as building on students' prior knowledge, using keywords, and providing visual aids**, are likely to help overcome** any **adverse face mask effects like adverse acoustic and visual language comprehension.**

Author and year	Type of study	Topic /Aim	Outcomes	Sample size	Main findings	Sponsor	Conclusions
**Shaw et al. 2024**	Scoping review	**To determine effective communication techniques when wearing facemasks.**	Speech perception, recognition of emotions.	39 articles, including 22 non-research articles mainly with adult samples	**Surgical masks minimally affect speech perception compared to other** non-transparent mask types. **Emotion recognition is less accurate when wearing facemasks**, highlighting the need for compensatory measures such as reducing background noise, using microphones to amplify voices, and employing clear speech.	National Institute on Aging of the National Institutes of Health, USA	**Communication difficulties associated with facemask use are evident.** Consequently, ongoing research to develop and improve strategies to overcome these challenges is vital.
**Singh et al. 2023**	Observational	To investigate **effects of different face coverings** (surgical masks and transparent face shields) **on language comprehension in bilingual children.**	Acoustic analyses, word recognition task.	n=28 children, three-year-old English-Mandarin bilingual children	**Children recognized words across all trial types** in Mandarin and in the no mask and surgical mask conditions in English.	ODPRT (Office of the Deputy President Research and Technology) research excellence grant, additionally supported by National Science Foundation BCS-2141326.	Children were able to recognize words in both of their native languages when presented through surgical masks. **At a young age, bilingual children are able to recover familiar words from masked language input**.
**Sivaraman et al. 2020**	Case series	To evaluate **graduated exposure and shaping to teach mask wearing** and (indirectly) **abate mask-induced problem behavior in children with autism spectrum**	Tolerance of graduated exposure of wearing a mask, blood oxygen saturation.	n=6 children, 5 boys, 1 girl, 6-8 years old	**Challenging behavior associated with mask wearing is surmountable with tolerance training.** which did not affect the percentage of oxyhemoglobin saturation of	None	Finally, all participants wore a face mask for a period of 10 min without exhibiting challenging behaviour after previous tolerance training. **Tolerance training treatment in children with developmental disorders exhibiting resistance to healthcare**

Author and year	Type of study	Topic /Aim	Outcomes	Sample size	Main findings	Sponsor	Conclusions
		**disorder.**			participants within 10 minutes, and caregivers found the intervention useful.		**routines is effective.**
**Smart et al. 2020**	Communication of observational study	To a**ssess the perceived wearability of three facemasks types** marketed in the UK to protect children against exposure to air pollution.	Mask ratings after a standardised walking and running activity	n=24 children, aged 8–11 years, 11 girls	The main complaints about the facemasks were being **too hot, to be hard to breathe through.and difficult to adjust.**	Bristol Medical School, University of Bristol	**Children's perceptions of facemasks are affected by the design, hotness and perceived breathability.**
**Stajduhar et al. 2022**	Randomized experimental study with random assignment to mask/no-mask condition of the CFMT-K.	To examine **if mask-wearing hinders face recognition abilities in school-age children.**	Cambridge Face Memory Test – Kids (CFMT-K).	n=72 children, ages 6-14 years	The use of face masks resulted in a **significant decline in face perception abilities**. The **holistic processing,** which is a key aspect of face perception, **was impaired for masked faces**, as indicated by a diminished face-inversion effect.	Supported by the Vision Science to Applications (VISTA) program funded by the Canada First Research Excellence Fund (CFREF).	**There are significant quantitative and qualitative changes in how school-age children process masked faces.**
**Surrain et al. 2023**	Observational	**To evaluate whether assessor masking impacts children's performance** on a widely used, individually administered **oral language assessment.**	Scores according to the Clinical Evaluation of Language Fundamentals Preschool–Second Edition Recalling Sentences subtest.	n = 45 children, 5–7 years old	**No evidence that students scored systematically differently in the masked condition.** Children with a home language other than English scored lower overall.	Institute of Education Sciences (ED)	**Children's performance on oral language measures is not adversely affected by assessor masking** and imply that valid measurements of students' language skills may be obtained in masked conditions. verbal information.
**Tamon et al. 2022**	Cross-sectional multisite survey	To investigate the **relationships between restricted**	Standardized questionnaires to assess daily life	n=102 children and adolescents with a clinical	Children and adolescents who exhibited lower-	JSPS KAKENHI, Japan Agency for Medical Research	Sensory-seeking behaviours, repetitive motor mannerisms and

Author and year	Type of study	Topic /Aim	Outcomes	Sample size	Main findings	Sponsor	Conclusions
		**interest and repetitive behaviour (RRB) characteristics and the effects of mask-wearing on social communicatio**n during the pandemic.	behaviours and regarding mask wearing.	diagnosis of autism spectrum disorder	order RRB before the pandemic experienced more difficulties with going out while wearing masks, **greater challenges with mask-wearing, and trouble interpreting others**' emotions while masked.	and Development (AMED)	movements, and adherence to rituals and routines prior to the pandemic could be key predictors of **challenges with mask-wearing and social communication for autistic children** and adolescents during the pandemic.
**Taxacher et al. 2023**	Observational	To examine the **impact of a face mask, worn by the speaker, on the speech intelligibility** of normal hearing **children and adolescents**.	Speech reception measured by the Freiburg monosyllabic test for sound field audiometry in silence and with background noise.	n=40 children and adolescents, aged 10 to 18	Speaking while wearing a face mask with background noise leads to a distinct **impairment of speech intelligibility**.	None	The results could help to improve the quality of future decision-making processes regarding masks. The results could be taken as baseline for comparison with vulnerable parts of society like hearing-impaired children and adults.
**Thomson 2022**	Communication	Emphasising **human rights** In addition to the isolated consideration of **epidemiological and physical safety issues** in child mask mandates	Do no harm principle by WHO and UNICEF, effectiveness of face masks against adverse effects (physical, psychological and psychosocial).	Not applicable	Reduction in viral transmission is not a pre-eminent cause that eclipses all other potential harms, including to **children's physical, psychological and psychosocial well-being**	None	**Other non-pharmaceutical interventions for children,** such as physical distancing, good hand hygiene and improved indoor ventilation do not engage the legal complexities of mask-wearing and **are a safer policy option for reducing SARS-CoV-2 transmission.**

Author and year	Type of study	Topic /Aim	Outcomes	Sample size	Main findings	Sponsor	Conclusions
**Wild & Kornfeld 2021**	Opinion	To emphasize the **impact of masks on physician paediatric patient relationships** and communication.	Not applicable	Not applicable	**Physicians with established relationships with their patients face a greater challenge in expressing empathy while wearing a mask. Masks can muffle speech and cause comprehension errors for both paediatric patients and physicians.**	None	**Speak clearly, describe your actions and expressions, and "smile with your eyes" during extended conversations to mitigate the impact of masks on the doctor-paediatric patient relationship.**
**Xiang et al. 2023**	Observational	To evaluate the **proportion of and factors influencing face mask use** and related hygiene practices.	Observations on the use of masks	n=40 children (2-5 years)	The **overall correct mask use was 84.5%, the incorrect mask use was 12.9% and absent mask use was 2.6%,. Face or mask touching behaviour was observed in 10.7% and 13.7% of individuals, respectively**..	National Centre for Infectious Diseases, Singapore	**Male gender, fabric mask usage and crowded indoor venues - were associated with lower mask compliance. A targeted public health messaging is recommendable.**

Table S4.

Included publications on physical symptoms and clinical conditions associated with face mask use in children. Details of the studies with indicators of their quality.

Author and year	Type of study	Topic /Aim	Outcomes	Sample size	Main findings	Sponsor	Conclusions
**Assathiany et al. 2021**	Survey	To evaluate the **acceptability and tolerance of mandatory face masks by children** as well as both **parents and paediatricians**	Questionnaires	2,954 parents, 663 paediatricians.	Parents **applied this measure because it was mandatory** (93.4%) even if they **disagreed** (63.3%). **Children said they were usually (80.9%) embarrassed by the mask.** The main symptoms were **headache (49.0%), speaking difficulties (45%), change in mood (45.2%) and breathing discomfort (28.1%)**. The paediatricians reported **fog on glasses (68.2%**), **breathing discomfort (53.1%), cutaneous disorders (42.4%) and headaches (38.2%)**.	Association Nationale pour la Formation Professionelle des Adultes (AFPA)	The **moderate adherence of parents to mask-wearing, was better in children. The side-effects noted by parents were frequent (headache, speaking difficulties, mood change, breathing discomfort).** Parents must be motivated by constantly renewing the explanations to their children and the justification for this strategy.
**Beytout et al. 2021**	Cross-sectional survey	To examine the **effects of the COVID-19 pandemic on children with psoriasis.**	30-question survey	n=92 children	22.8% of patients reported **difficulties in adhering to hygiene measures** due to their psoriasis, including the use of alcohol-	None	About half of the patients reported **worsening of their psoriasis. One third of the children reported difficulties due to wearing a mask.** It is crucial to use this data

Author and year	Type of study	Topic /Aim	Outcomes	Sample size	Main findings	Sponsor	Conclusions
					based hand sanitizers (47.6%), handwashing routines (42.9%), and **wearing a mask (28.6%).**		to enhance and adapt the monitoring of **chronic skin conditions** in both **children** and adults for future health crises.
**Kaliyadan et al. 2022**	Case reports	To report the **skin effects of increased use of face masks by children**	Fungal infection of facial skin after prolonged mask wearing.	n=7 children (2–10 years)	Mask wearing in children might be responsible for an increased presentation of **facial pityriasis versicolor** due to a **humid environmen**t and associated **sweating**	None	The **lesions on the face** were **specifically within the areas corresponding to the mask**, year-round persistent **humidity** and **high temperatures** may explain the higher incidence.
**Kisielinski et al. 2021**	Scoping review	The objective was **to identify, test, assess, and compile scientifically proven side effects associated with wearing masks.**	Physiological and subjective parameters.	65 studies	**Psychological and physical deteriorations and the multiple symptoms consistently observed are described as Mask-Induced Exhaustion Syndrome (MIES).** In children, in addition to **blood gas changes (rise in CO_2, fall in O_2), humidity, heat and exhaustion,** there is disturbance of verbal and non-verbal communication with blocking of positive perceptions	None	**Prolonged mask-wearing by the general population could result in significant effects and consequences across various medical fields.** **It is necessary to protect children in particular from harm caused by uncertified masks and improper use.**

Author and year	Type of study	Topic /Aim	Outcomes	Sample size	Main findings	Sponsor	Conclusions
					(smiling and laughing) and emotional mimicry.		
**Ratchatavech et al. 2022**	Survey	To **investigate of facemask wearing in children, including the benefits, drawbacks, and negative consequences.**	Structured internet questionnaire (parents , caregivers)	n=706 children, between 4 months and 18 years old.	**44.9% children showed negative consequences from wearing facemask. Respiratory discomfort/breathing difficulty) were found in 33.99% besides cutaneous adverse effects up to 16.3 % (itch symptoms, rash, pressure effects, and acne).** A slight decrease in allergic symptoms (14.45%) respiratory infections (5.95%) was observed.	Khon Kaen University, Faculty of Medicine in Thailand.	When there is an indication to wear a facemask in children, **selecting an appropriate size of facemask will reduce the risk of facemask adverse reactions in the paediatric population.**
**Schwarz et al. 2021**	Online registry	To assess the **effects of wearing a mask in children and adolescents**.	Demographic data, pre-existing conditions, circumstances and duration of mask wearing, type of mask, presence of complaints from the child about mask discomfort, symptoms, behavioral issues, personal attitudes toward government COVID-19 protective	n=17854 parents with n=25930 children	**68% of children complain about impairments caused by wearing masks.** Reported side effects include **irritability (60%), headaches (53%), difficulty concentrating (50%), reduced happiness (49%), reluctance to attend school or**	None	This is the **worldwide first registry to capture the effects of wearing face masks in children and adolescents**. The frequency of use and range of symptoms underscore the importance of the topic and **call for representative surveys, randomized controlled trials comparing different types of**

Author and year	Type of study	Topic /Aim	Outcomes	Sample size	Main findings	Sponsor	Conclusions
			measures.		**kindergarten (44%), discomfort (42%), learning difficulties (38%), and drowsiness /fatigue (37%).**		**masks, and a risk-benefit assessment of mask mandates for the vulnerable group of children**.
**Shoaib et al. 2022**	Case study	Case series of 2 patients with **superficial eye injury due to face mask use** in children.	Corneal injuries	n=2 children	**Corneal abrasions while wearing adult face masks in childre**n are due to increasingly higher chances of **direct trauma to the eyes** as these masks are loose and the hard ends of the upper margin are raised to eye level.	None.	**There is potential of the face masks causing ocular surface injury, especially in children**
**Zanotti et al. 2020**	Letter to the editor	To highlight **potential complication of incorrect development of the auricle by permanently modifying the cartilage of the concha in growing children.**	Discomfort associated with ear loops, discussion of elastic and non-elastic deformation.	Not applicable	The **elastic loops** cause constant **compression on the skin and on the cartilage** of the auricle, which leads to **lesions** in the retroauricular region **if the masks are worn for many hours a day**.	None	Pre-adolescent children have undeveloped auricular cartilage with less resistance to deformation; **prolonged pressure from the elastic loops of the mask at the hollow or, even worse, at the anthelix level can influence the correct growth and angulation of the outer ear.**

Table S5.

Included publications on physiological, metabolic, and toxicological effects. Details of the studies with indicators of their quality.

Author and year	Type of study	Topic /Aim	Outcomes	Sample size	Main findings	Sponsor	Conclusions
**Ahmadi et al. 2024**	Case control	To evaluate the **effect of surgical face masks on pulmonary artery systolic pressure (PASP) in children and adolescents**	Echocardiography, heart rate and blood oxygen saturation (SpO_2).	n=110 children and adolescents, boys and girls aged 3-18 years. Two groups of 60 healthy subjects and 50 with congenital heart disease)	**During 10 minutes surgical mask wearing there was rise in tricuspid regurgitation, pulmonary regurgitation and pulmonary artery systolic pressur**e in both groups. There were no significant changes in heart rate or SpO_2.	None	**Face masks should be removed during echocardiography in children and adolescents due to rise in pulmonary artery systolic pressure.**
**Amirav an Lavie 2022**	Case reports	To report **respiratory symptoms in children while wearing a mask.**	Respiratory symptoms	n=3 children, two girls, one boy, 9 to 12 years	**Hyperventilation** mimicking asthma while wearing a mask.	None	Potential **anxiety and hyperventilation** while the mask is being worn, are **symptoms that can mimic common respiratory disorders, such as asthma.**
**Brooks et al. 2023**	Observational	To explore **effects of masking on ventilation in children** and adults **during 15 minutes of mask wearing**	End-tidal CO_2 ($ETCO_2$), inspired CO_2 (ICO_2), and respiratory rate were measured by nasal cannula attached to an anesthesia machine D-fend module. Pulse oximetry and heart rate were also followed.	n=49 children, 2-14 years (71 adults)	**Masking resulted in a statistically significant rise in $ETCO_2$ levels and ICO_2** in adults and in **children**. The final respective $ETCO_2$ levels remained within normal limits. Pulse oximetry, heart rate, and respiratory rate were not significantly affected.	None	**Wearing of surgical masks results in a statistically significant rise in ICO2 and a smaller rise in ETCO2.** Because ETCO2 and other variables remain well within normal limits **while wearing a mask for 15 minutes**, these changes are interpreted as clinically insignificant..

Author and year	Type of study	Topic /Aim	Outcomes	Sample size	Main findings	Sponsor	Conclusions
**Canellas et al. 2023**	Experimental	To study the **contaminants from printed and unprinted children`s masks** to saliva.	migration of volatile compounds, identification of non-volatile migrants with mass spectrometry.	11 masks,<br>3 FFP2,<br>8 surgical	It is **possible for hazardous substances to migrate from the masks to the saliva**	University of Zaragoza/Ibercaja and the Gobierno de Arag´on and Fondo Social Europeo, “European Union Next Generation EU/PRTR.	**Thirteen volatile compounds were found in the masks.** Toluene, chlorobenzene, irganox 1076 and 2-(2-butoxyethoxy)ethyl acetate were all found to migrate to a saliva simulant from the masks studied. **Toluene** and **tris(3 methylphenyl)phosphate** (which migrated only from printed masks), have been listed as **hazardous priority substances.**
**Castro et al. 2023**	Observational	To evaluate the **effect of cotton face mask use** on partial **blood oxygen saturation and pulse rate at rest**.	Five minutes mask wearing:<br>Pulse rate,<br>oxygen saturation.	n=50 children<br>(25 boys, 25 girls)<br>4-9 years old	Face masks: **increased pulse rate** and **reduced arterial blood oxygen saturation**	Fundação de Amparo à Pesquisa do Estado de Minas Gerais (FAPEMIG),<br>the Conselho Nacional de Desenvolvimento Científico e Tecnológico (CNPq) and the Dean’s Office for Research (Pró-reitoria de Pesquisa) at Universidade Federal de<br>Minas Gerais.	Two-layered cotton tissue face masks were associated with a **higher pulse rate** and **reduced arterial blood oxygen saturation without clinical disorders within 5 minutes of wear.**
**Eberhart et al. 2021**	Narrative review	To describe the **existing knowledge about children wearing face masks**.	Physiological effects	Two<br>children studies	Face masks **increase carbon dioxide and breathing difficulties in children**	None	Informations on physiological variables in children wearing masks is very limited. **Further studies are needed, particularly to explore the impact on pre-existing respiratory**

Author and year	Type of study	Topic /Aim	Outcomes	Sample size	Main findings	Sponsor	Conclusions
							**problems in children.**
**Goh et al. 2019**	Randomised, two-period cross-over self-control trial	To evaluate **15 min** of **wearing special N95 respirator vs wearing N95 respirator with microfan vs wearing no facemask during common physical activities**.	Endtidal CO_2 ($ETCO_2$), comfort level with visual analogue scale (VAS)	n=106 children, aged 7–14 (59 boys)	**Wearing a mask increased the $ETCO_2$ when at rest and with brisk walkin**g. The VAS showed that 93% of the children experienced no breathing difficulty when using the mask while **7% (4 males and 3 female subjects) indicated that they experienced mild breathing difficulty.**	**Innosparks Pte Ltd, Singapore. Two of the authors (Meng Wai Mun and Wei Liang Jerome Lee) included on the authorship list, are staff of the Innosparks Pte. Ltd, the company whose product was used in the study.**	**15 minutes of special N95 respirator use (with or without microfan) appears safe for use in children 7 to 14 years old with no underlying medical conditions and in the setting of routine daily activities including brisk walking.**
**Happernegg and Kerbl 2023**	Observational study	To answer the question of **whether a mask requirement has a negative impact on physiological processes in children and adolescents.**	Inspiratory and expiratory CO_2 (capnography), heart rate, respiratory rate , the oxygen saturation SpO_2 and the transcutaneous CO_2.	n=15 children, 6-17 years (5 girls, 10 boys)	10 minutes wearing an FFP2 or surgical mask mask showed a **significant increase in inspiratory and expiratory CO_2** values. **All other measured parameters were largely unaffected**.	Medical University of Graz	**Mask indications should be evidence-based and well-considered.**
**Hodges et al. 2024**	Two-stage cross-sectional study	To evaluate the effect of **mask use** on **peripheral oxygen saturation (SpO_2)** in those with and those without **asthma**.	SpO_2 levels, Paediatric Dyspnoea Scale (PDS)	n=50 paediatric subjects, 25 with and 25 without asthma	**No differences in oxygen saturation or reported levels of dyspnoea** were observed between children with asthma and those without **when performing the 6-minute walk test**	None.	**The use of masks did not impact SpO_2 levels in children with or without asthma during 6 minutes,** whether at rest or during low-to-moderate intensity exercise.

Author and year	Type of study	Topic /Aim	Outcomes	Sample size	Main findings	Sponsor	Conclusions
					while wearing masks. The median SpO_2 levels for both the asthma and non-asthma groups remained at or near 99% throughout all time points.		
**Kisielinski et al. 2023**	Scoping review	To investigate the **toxicological effects of wearing masks in terms of carbon dioxide (CO_2) rebreathing on developing life**.	CO_2 inhaled air (mask breathing zone)<br><br>CO_2 body content ($PETCO_2$, $PtCO_2$, $FeCO_2$, $PaCO_2$)<br><br>experimenteal animal data	43<br>included studies	In the inhaled air.from masks **carbon dioxide rises above NIOSH norms for 15 min and 3 hours**.<br><br>**Toxic CO_2-levels from animal studies exceeded.**<br><br>Statistically **significant body CO_2 rise.**	None	Extended mask use may be related to observations of **stillbirths** and to **reduced verbal motor** and overall **cognitive performance in children** born during the pandemic.<br>**In adolescents** possible **neuron destruction**, which includes **less activity**, **increased anxiety** and **impaired learning and memory**.<br>There is also data indicating **testicular toxicity in adolescents.**
**Kisielinski et al. 2024**	Scoping review	To study the **potential of face masks for the content and release of inanimate toxins**.	Release of<br><br>micro- and nanoplastics (MP, NP),<br><br>organic toxins<br><br>anorganic toxins	24<br>included studies	**Mask content and release show exceedances of USEPA, WHO, EU Air Quality and Oeko-Tex® Standard 100 limits for Micro- and Nanoplastics (MP, NP), organic toxins and anorganic toxins.**	None	**Masks** are a **source of potentially harmful exposition to toxins with health threatening and carcinogenic properties** at population level with almost zero distance to the airways.

Author and year	Type of study	Topic /Aim	Outcomes	Sample size	Main findings	Sponsor	Conclusions
					**Phthalates: 43 fold exceedance of the carcinogenic risk in children masks.**<br><br>**Volatile Organic Compounds: 25 fold exceedance of the carcinogenic risk in children masks.**		
**Lubrano et al. 2021**	Cohort study	To examine **whether the use of surgical facial masks** among children **is associated with episodes of oxygen desaturation or respiratory distress**	Partial pressure of end-tidal carbon dioxide ($PETCO_2$), oxygen saturation (SaO_2), pulse rate (PR), and respiratory rate (RR) and perfusion index (PI).	n=47 healthy children (10 months to 10 years)	Wearing surgical face masks for **30 minutes** was **not associated with changes in respiratory parameters or clinical signs of respiratory distress.**	None	**The use of surgical face masks for 30 minutes among children was not associated with changes in respiratory function, including among children aged 24 months or younger.**
**Lubrano et al. 2021**	Randomised controlled trial	To assess whether **use of a N95 mask by children** is associated with episodes of desaturation or respiratory distress.	42 minutes of mask use in children, with N95 (30 min), without vs N95 with exhalation valve including 12-minute walking test: carbon dioxide ($PETCO_2$), oxygen saturation, pulse rate and respiratory rate.	n=22 healthy children	Significant **increases in respiratory rate and** $\mathbf{PETCO_2}$ in the children wearing a N95 mask without an exhalation valve.	Not mentioned	**The use of a N95 mask could potentially cause breathing difficulties in children**, particularly during a physical activity. Wearing a surgical mask may be more appropriate for children.
**Lubrano et al. 2021**	Dataset	**Children use of a N95 mask** with and without an exhalation valve	Mask use in children (30 min), with N95 without vs N95 with exhalation valve with additional 12-minute walking test: carbon dioxide	n=22 healthy children	**No data given for pulse rate and** $\mathbf{PETCO_2}$ in the children wearing a N95 mask without an exhalation valve,	Approved by Latina local health authority (Italy)	**The use of a typical N95 mask causes a significant blood oxygen saturation drop**, particularly during physical activity.

Author and year	Type of study	Topic /Aim	Outcomes	Sample size	Main findings	Sponsor	Conclusions
			($PETCO_2$), oxygen saturation, pulse rate and respiratory rate.		**significant decrease in oxygenation after 60 minutes and during walking test.**		
**Lubrano et al. 2022**	Observational study	To evaluate **respiratory distress under use of a surgical or N95 mask for overweight and obese children**.	Partial pressure of end-tidal carbon dioxide ($PETCO_2$), oxygen saturation (SaO_2), pulse rate (PR), and respiratory rate (RR) during a 72 min test: 30 min without a mask, 30 min wearing a mask, and then during a 12 min walking test.	N=30 children, 15 healthy, 14 obese (6 to 12 years old)	**A significant increase in PR and RR after the walking test with both the masks. In overweight children, there was no significant change in SaO_2 during the study period; there was a significant increase in $PETCO_2$ and an increase in PR, and RR after walking test.**	None	**There was a significant correlation between $PETCO_2$ and body mass index. Overweight or Obese children who wear a mask are more prone to developing respiratory distress, which causes them to remove it frequently.**
**Mallet et al. 2022**	cross-sectional study	To determine whether **wearing a surgical facemask during intense physical exercise** of **6 minutes** results in abnormal gas exchange in **children with underlying respiratory issue**s.	The forced expiratory volume in 1 second (FEV1) was measured before 6 minutes of exercise and at 2, 5, 10, and 15 minutes post-exercise. Oxygen saturation (SpO2) was recorded before and after exercise using a fingertip oximeter. Capillary blood gas analysis was conducted immediately after exercise to evaluate gas exchange.	n=25 children, median age 14 years	Fifteen children (60%) experienced a **decrease in FEV1 of more than 10%,** indicating **exercise-induced airway obstruction.** There were no instances of oxygen desaturation observed after exercise, with **the lowest SpO2 recorded at 93%**.. **The highest carbon dioxide**	Swiss National Science Foundation	**Surgical facemasks in children with exercise-induced symptoms (EIS) during submaximal treadmill exercise of 6 minutes testing did not show evidence of abnormal gas exchange (oxygen saturation and carbon dioxide retention)** Controlled studies with a larger number of children are needed to confirm the preliminary findings.

Author and year	Type of study	Topic /Aim	Outcomes	Sample size	Main findings	Sponsor	Conclusions
					**tension measured was 5.67 kPa**, well below the upper limit of normal (6.0 kPa).		
**Martellucci et al 2022**	Observational pilot study	**To explore excess CO_2 as a consequence of 5 minutes wearing surgical masks or FFP2 respirators, among adults, children, and the elderly.**	Inhaled air CO_2 in ppm (capnography), respiratory rate, blood oxygen saturation and endtidal CO_2 ($ETCO_2$).	N=24 children	**Mean CO_2 detected inside the mask in children was significantly elevated:** Surgical mask 7091 ± 2491 ppm, FFP2 respirator 13665 ± 3655 ppm. **The CO_2 concentration was significantly higher among minors and the subjects with high respiratory rate.** Respiratory rate and blood oxygen saturation did not differ substantially without or with the masks.	National Special Integrative Fund for Research (FISR—Fondo Integrativo Speciale per la Ricerca) of the University of Ferrara.	**The occupational exposure limit of 5000 ppm threshold was largely exceeded in children wearing surgical masks and in all age classes when wearing FFP2 respirators. If these findings are confirmed, the current guidelines on masks use should be reevaluated.**
**Reychler et al. 2022**	Observational	To evaluate the **effect of wearing a surgical facemask on perceived exertion, dyspnoea, physical performance, and cardiorespiratory response** during a **one minute** submaximal exercise test in children	Perceived exertion (modified Borg scale), dyspnoea (Dalhousie scale), heart rate, and pulsed oxygen saturation.	n=38 healthy children, 8 to 11 years,	The perceived exertion and breathing effort was higher with the mask. The cardio-respiratory demand was not clinically modified.	Institut de Recherche Expérimentale et Clinique (Université Catholique de Louvain, Brussels, Belgium)	**The surgical facemask had no impact on dyspnoea, cardiorespiratory parameters, and exercise performance during a one minute submaximal exercise in healthy children.**

Author and year	Type of study	Topic /Aim	Outcomes	Sample size	Main findings	Sponsor	Conclusions
**Roberge 2011**	Review	To give an overview of literature on the **use of face masks by children** to protect against respiratory infections, including **effects on children's respiratory physiology**	Face mssk use, respiratory physiology (dead space, tidal volume, respiratory rate, breathing resistance, carbon dioxide), efficacy, tolerance, safety issues.	68 publictions	Mask use (surgical, respirators) by children has some **safety issues (proper use), fit problems and lack of tolerance.** Also **higher breathing resistance, dead space volume and inspiratory CO_2 levels** are also important.	None	**Little is known about the physiological and psychological burdens imposed by masks and children's ability to correctly use and tolerate them. Future research should address such issues.**
**Schulte-Körne et al. 2022**	Prospective randomised cross-over study	To investigate the **influence of masks on peak physical performance in 11-year-old male football players.**	Heart rate, rate off perceived exertion (Borg scale), plasma lactate, SpO_2 (oxygen saturation) during a gradual load pattern on the treadmill (12 minutes)	n=11 healthy boys from a german professional soccer club, 11 years old	In the maximum load range there was a **significant reduction in the total running time and a significant increased subjective stress perception** due to changed breathing pattern and increased airway resistance.	DEAL-consortium	**12 minutes surgical mask use is generally possible even during sporting activities, especially as long as the exercise is primarily performed in the aerobic intensity range. However, it should be borne in mind that maximum performance in children can be negatively affected by masks.**
**Weigelt et al. 2023**	Observational	To evaluate **the effects of wearing FFP2/N95 face masks on cardiopulmonary function in children.**	A maximum of 10 minutes of cardiopulmonary exercise tests as an incremental step test on a treadmill: End-expiratory gas exchange for oxygen and carbon dioxide ($petO_2$ and $petCO_2$), cardiac, ventilatory, and metabolic parameters.	n=20 healthy children , aged 8–10 years	**The end-tidal values for CO_2 increased significantly and the end-tidal values for O_2 decreased significantly without reaching levels of hypercapnia or hypoxia during 10 test minutes.**	DEAL-consortium	**Even though the end-tidal values for CO_2 were significantly higher and for end-tidal O_2 significantly lower during the whole 10 minute exercise test, these values did not reach pathological values.** Stopping physical exercise lessons because of the minor

Author and year	Type of study	Topic /Aim	Outcomes	Sample size	Main findings	Sponsor	Conclusions
			**values for oxygen saturation were not included**				physiological effects of wearing masks instead of simply stopping pushing children to perform at their best seems premature and should be reconsidered in the future.
**Walach et al. 2022**	Observational	To measure the **CO_2 content of inhaled air under surgical and FFP2 masks in children during 6 minutes wear.**	The CO_2.content in ambient air and inhaled air while wearing masks (9 minutes), measured with a G100 CO2 incubator analyzer and PCE-CMM 10 by PCE, oxygen saturation of the blood, breathing frequency and pulse	n=45, 25 boys, 20 girls, with a mean age of 10.7, range 6–17 years	**Nine minutes mask wearing significantly resulted in 13,100 ppm under surgical mask and 13,900 ppm under FFP2 mask in inhaled air.** which is **by a factor 6 higher than the "inacceptable" 2000 ppm air limit** by the German Federal Environmental office, being at least 5 times higher than the CO_2 content in normal air.	None	**The carbon dioxide rise in inhaled air under masks (surgical and FFP2) is far beyond the level of 2,000 ppm considered the limit of acceptability and beyond the 1,000 ppm that are normal for air in closed rooms. Decision makers and law courts should take into consideration when establishing rules and guidance to fight infections.**
**Wang et al. 2021**	Communication	To discuss the **hypothetical mechanisms by which exercise with face masks or respirators can induce detrimental effects** on the cardiovascular system in students.	Physiological effects	Not applicable	**Mean carbon dioxide levels rise and the mean oxygen levels drop in the mask breathing space**, along with glucose breakdown, a rise in lactic acid levels, the **sympathetic nervous system is stimulated** (blood	None	**Several students without documented cardiac issues experienced sudden death when they were running in physical training classes with surgical masks or N95 masks in China.** Although a rarity, this risk is higher, especially in people with existing cardiac comorbidities.

Author and year	Type of study	Topic /Aim	Outcomes	Sample size	Main findings	Sponsor	Conclusions
					pressure rise) with the possibility of induced respiratory sinus arrhythmia.		

Table S6. Included publications containing claims about face mask effects. Details of the studies with indicators of their quality.

Author and year	Type of study	Topic /Aim	Outcomes	Sample size	Main findings	Sponsor	Conclusions
**Esposito and Principi 2020**	Opinion	Answering the question: **To mask or not to mask children to overcome COVID-19?**	Mask wearing and types	Not applicable	**Cloth masks, surgical masks and N95 recommended for children older than 2 years of age.**	None	It is **necessary that the use of masks in children is preceded by a strong parental work and school lessons.**
**Gyawali 2021**	Narrative review	To discuss **the effectiveness of face mask usage in children and its associated challenges, myths, advantages, and disadvantages.**	Advantages and disadvantages, challenges“myths” and “facts” regarding masks and children,	Not applicable	Use of masks is not associated with a change in respiratory function, no gas exchange abnormalities. Masks can provide protection against other various respiratory viruses like Influenza or Rhinovirus.	None	**Masking is a useful practices. As children learn very quickly, they can adapt to masking with careful guidance from parents and teachers. Wearing a proper mask by encouraging children will help prevent and control the spread of the coronavirus and make all our lives safer.**
**Huppertz et al. 2021**	Statement by the German Society for Paediatric Infectiology (DGPI)	**To propagate the use of masks in children to prevent infection with SARS-CoV-2**	Not applicable	Not applicable	**masks are an important means of restricting the spread of SARS-CoV-2. Despite being cumbersome and inconvenient, in children relevant adverse effects have not been reported and are not to be expected.**	None	**Children 10 years or older can use masks efficiently and children in primary schools may use masks.**
**Lopes et al. 2021**	Statement by the Association of Schools of Public Health in the European Region (ASPHER) and the	**Consensus statement for the use of masks by children.**	Not applicable	Not applicable	**Masks can offer the same level of protection against COVID-19 for children as they do for adults**.	None	**Recommendations are provided regarding the size, material, and ergonomics of children's masks.** The authors also address the

Author and year	Type of study	Topic /Aim	Outcomes	Sample size	Main findings	Sponsor	Conclusions
	European Academy of Paediatrics (EAP).				The availability of masks sized for children is rare. **For children, masks hold significant psychological implications**. Children should not use respirators (FFP2/3, N95). Special consideration is necessary when deciding to have children with pre-existing disabilities wear masks.		**psychological impact** on children when required to wear masks, as well as the **challenges faced by children with disabilities**.
**Moschovis et al. 2021**	Opinion	Defining the **factors driving infectivity and transmission for infection control and containment of the pandemic.**	Aerosol science, transmission via droplets and aerosols.<br><br>Mask use to mitigate the risk of transmission.	Not applicable	**SARS-CoV-2 can be transmitted via Droplets and aerosol**s.. Understanding the behavior of virus-laden droplets and aerosols in confined spaces is urgently needed for schools and workplaces to open safely.	National Institutes of Health and the Cystic Fibrosis Foundation	**The usage of masks is critical for limiting the aerosol spread of SARS-CoV-2 among children and adults.**
**Villani et al. 2020**	Statement by the Italian Paediatric Society	To face **misconception towards the use of face masks** and to spread **scientific trustable information.**	Face masks in children	Not applicable	The **Italian Paediatric Society promotes the use of masks among the paediatric population**, **acknowledging that they are effective** and explaining the importance of their	None	**The Italian Paediatric Society statement faces misconception towards the use of face masks and highlights scientific trustable information on facial masks.**

Author and year	Type of study	Topic /Aim	Outcomes	Sample size	Main findings	Sponsor	Conclusions
					proper use along with other hygiene measures.		
