# Supplementary material for: Risks and benefits of face masks in children
Source: Front Pediatr. 2026 Mar 13;14:1679586. doi: 10.3389/fped.2026.1679586 (PMC13047964; doi:10.3389/fped.2026.1679586)
Supplement: Supplementary file 3 — Data sheet 3: Supplementary C [file Datasheet3.pdf]

Supplement C

Risks and benefits of face masks in children

Kai Kisielinski [1,*], Claudia Steigleder-Schweiger [2], Susanne Wagner [3], Stephan Korupp [4], Stefan Hockertz [5], Oliver Hirsch[6]

[1] Clinical Medicine (Surgery), Emergency Medicine and Social Medicine, Private Practice, 40212 Düsseldorf, Germany.

[2] Department of Paediatrics, University Hospital of Salzburg, Paracelsus Medical University, 5020 Salzburg, Austria.

[3] Non-Clinical Expert, Veterinarian, Wagner MSL Management, 15831 Mahlow, Germany.

[4] Surgeon, Emergency Medicine, Private Practice, 52070 Aachen, Germany.

[5] Toxicology, Pharmacology, Immunology, tpi consult AG, Haldenstr. 1, CH 6340 Baar, Switzerland.

[6] Department of Psychology, FOM University of Applied Sciences, 57078 Siegen, Germany

*** Correspondence:** ***kaikisielinski@yahoo.de***

Supplement C contains the unshortened version of the original manuscript for the scoping review on the risks and benefits of face masks in children, which served as the template for the shortened published version. This comprehensive manuscript includes the full methodology, results, discussion, and references, providing detailed context and analyses that were condensed in the final publication. See Supplement A for the methodological framework and GRADE equivalent evidence profile, and Supplement B for the PRISMA flow diagram and extraction tables of included publications.

Risks and benefits of face masks in children

***Kai Kisielinski* [1,*], *Claudia Steigleder-Schweiger* [2], *Susanne Wagner* [3], *Stephan Korupp* [4], *Stefan Hockertz* [5], *Oliver Hirsch*[6]**

[1] Clinical Medicine (Surgery), Emergency Medicine and Social Medicine, Private Practice, 40212 Düsseldorf, Germany.
[2] Department of Paediatrics, University Hospital of Salzburg, Paracelsus Medical University, 5020 Salzburg, Austria.
[3] Non-Clinical Expert, Veterinarian, Wagner MSL Management, 15831 Mahlow, Germany.
[4] Surgeon, Emergency Medicine, Private Practice, 52070 Aachen, Germany.
[5] Toxicology, Pharmacology, Immunology, tpi consult AG, Haldenstr. 1, CH-6340 Baar, Switzerland.
[6] Department of Psychology, FOM University of Applied Sciences, 57078 Siegen, Germany

* Correspondence: *kaikisielinski@yahoo.de*

Abstract

Background: Children, a significant and vulnerable portion of the global population, are particularly susceptible to environmental factors.
Methods: We conducted a systematic search and scoping review of 3,149 articles, including 107 publications from medical literature, to assess mask use in children also during the 2020–2023 pandemic. We examined expected (viral) protection versus scientific evidence and side effects, synthesising findings with SWiM and GRADE frameworks for evidence certainty and the Cochrane adverse effects approach.
Results: Masking children lacks ecological validity, with high-quality studies showing little real-world effectiveness against viruses. Conversely, side effects are evident. Masks contain hazardous materials (carcinogens, heavy metals, organic compounds, microplastic), impacting children's health by altering inhaled air (including elevated carbon dioxide) and causing many physical symptoms and bio-psychosocial issues (MIES syndrome), akin to sick building syndrome. Toxicological assessments highlight risks to young biology. Evidence certainty is high for non-effectiveness, moderate for risks and side effects, and low to very low for viral protection or benefits in children.
Conclusion: With a negligible COVID-19 mortality rate in children (0.0003%) and no evidence of child-to-child or school-based transmission, masks offer little benefit. The documented adverse effects—respiratory impairment, toxicity, and health risks—outweigh any justification for their use. An individual risk-benefit analysis is essential, but this review suggests avoiding this intervention in children due to its numerous downsides and lack of proven efficacy.

Keywords:
Children; masks; N95; surgical mask; risk; adverse effects; long-term adverse effects; health risk assessment; toxicity; MIES syndrome

1. Background

Children can be more vulnerable than adults to environmental hazards, such as those presented by chemicals, due to their physiological differences and unique behaviour. Risk assessment methodologies that specifically consider children are required to ensure that potential risks are addressed [1]. However, during the SARS-CoV-2 pandemic this risk assessment has not been performed adequately.

Since 2020 until 2023 during the SARS-CoV-2 pandemic wearing coverings of mouth and nose has become a new normal part of everyday life for many children around the world [2,3] because there was belief that masks could help contain the virus [4–7]. The World Health Organisation stated in the 2021 Mask Guidelines, that virus spreading may even increase, if mask management guidelines are not strictly followed [8]. For example, the WHO urged people to clean their hands before putting on a mask, as well as before and after taking it off and after putting it on. When the mask is removed, it should be stored in a clean plastic bag or disposed of in a waste bin [8]. There is no question that children are unable to follow these instructions when even experts and high ranking politicians are unable to, infringing every single point of the mask management guidelines nearly by the minute, especially high-ranking health experts and the chair (see online Video from the U.S. Senate Committee on Health, Education, Labor and Pensions;
March 18, 2021, 10:00 a.m., 216 Hart Senate Office Building; Full Committee Hearing "Examining Our COVID-19 Response: An Update from Federal Officials", especially at the time markings 0:34:47, 2:08:09, 2:12:26; 2:23:26, 2:26:33 and 2:37:30) [9]. Even medical personnel needs training in so-called donning and doffing to prevent spreading and self-infection. It could never be proven that children follow such instructions by the majority and with respect to worst case risk assessment it has to be assumed that mask mandates in children are associated with increased risk in transmission because of improper handling while being of no benefit at the same time. But what about the risk of wearing such devices for this vulnerable group? The WHO guidance on the use of masks in children in the community, published in August 2020, already pointed out that the benefits of mask use in children must be weighed up against the potential harms, including social and communicational concerns [10].

Fears that widespread pandemic measures would lead to dysfunctional social life with degraded social, cultural and psychological interactions had also been expressed by other experts [11–14]. However, in many countries children had been mandated to wear masks in schools and comparable facilities for large proportions of the day [15–17]. Even children down to 2 years of age were forced to do so despite diminishing disease severity in children [18,19], despite widespread infection with COVID-19, while there was no confirmatory, high-quality evidence of benefits of face masks [20]. For example, children were instructed by the American Academy of Paediatrics to wear masks even when playing sports indoors [21]. During the pandemic, nudging [22] as well as direct social pressure also had influence on children mask wearing habits [4].

After the pandemic, the use of face masks by children became less common. But some apparently incomplete and biased publications [23] do unfortunately conceal large scientific evidence on lacking effectiveness against viruses [24–26] and conceal clearly proven side effects [27–31]. Moreover, those concept and assumption driven publications are trying to propagate face mask wearing also in children, especially in future virus pandemics [23] and also for other purposes like air pollution [32]. Apart from an obvious conflict of interest, which is partly responsible for the bias of these studies, these studies overemphasize the positive effects, although there is a lack of empirical certainty for these. At the same time, they neglect the negative effects. However, regarding face masks as a non-pharmaceutical intervention in children the establishment of a balanced and scientific view without any interference of politics appears to be necessary [15,16,27–29,33,34] and there should be no doubt that a medical and ethical risk-benefit assessment is recommended [35]. Therefore, our approach aims at a holistic medical multidisciplinary view with rigorous empirical evaluation from a risk assessment perspective [36], in the spirit of evidence-based medicine (EBM)[37].

According to UNICEF, the global population under the age of 18 is around 2.4 billion while writing this review [38]. Taking into account the total world population of approximately 8 billion [39], children make up just under a third of the world's population, which emphasises the importance of the children mask topic. Children are a subgroup more susceptible to potential negative environmental factors because they have early life conceptual tissues with less well-developed protective/conjugative pathways [40,41].

Therefore, a careful risk-benefit analysis appears to be necessary, especially when children use masks, as masks show significant undesirable side effects and risks [27–29,34,35].

To the best of our knowledge, the first holistic narrative literature overview on face masks and children appeared in 2011 and was written by Raymond Roberge [42], nearly ten years before the SARS-CoV-2-pandemic 2020-2023. Another overviewing narrative mini-review was performed ten years later [43], during the pandemic in 2021 including only two children studies. After the pandemic Sandlund et al performed a narrative review aiming at the assessment of benefits and harms of masks in children [44]. However, without a systematic literature search and and without a very in-depth clinical-medical assessment of the risks and adverse effects.

So there is a need for a systematic update, particularly as new and more data emerged during and after the SARS-CoV-2 pandemic. Altogether, there is sparse published scientific data specifically and holistically summarising the issue of the use of protective face masks in children. With the aim to shed light on this important topic we decided to conduct a scoping review with systematic search on this topic in order to evaluate reliable scientific data and to establish an evidence update of the knowledge including a preliminary risk-benefit assessment.

2. Material and Methods

2.1. Review type and scope

Our scoping review [45] with systematic search according to PRISMA (Preferred Reporting Items for Systematic Reviews and Meta-Analyses) [46], serves as an overview of the literature. To provide an objective and holistic view, it includes reported positive as well as negative aspects and diverse effects reported regarding mask use in children. It is essential that adverse effects are also addressed to avoid one-sided summaries of the evidence. According to the Cochrane Handbook chapter on adverse effects of interventions, it is preferabe to overestimate rather than underestimate a risk [47–49]. Additionally, the Cochrane Handbook for Systematic Reviews of Interventions states that inconsistent and poor reporting of adverse effects is a problem, and rare or long-term adverse effects may only be observed in non-randomised studies such as large cohort studies or case-control studies [47]. For this reason, we have not imposed any restrictions on the selection of studies based on their methodology or type.

In our scoping review, we also aimed to discuss the effectiveness of face masks in children, particularly with respect to viral infections and transmission, which was the primary argument for their use during the pandemic.

Our scoping review includes elements of a critical review to develop a comprehensive and in-depth understanding of the research field concerning masks and children. This approach allows us to consider both the breadth and depth of the evidence and derive sound recommendations [45]. In this respect, we extended beyond the classic approach of a scoping review by not only analysing the content of the literature but also assessing its methodological quality to make well-founded statements about the level of evidence. This brings our scoping review somewhat closer to a systematic review in terms of methodology [45].

2.2. Aim and Objectives

The PICO framework (participants, intervention, comparisons, outcomes) [50] is given in Supplement A, Supplementary Table A .

The PICO statement for the review is: "In children (P) wearing face masks (I) compared to not wearing them (C), what effects, benefits and risks (O) are described in the available literature, and how strong is the evidence?"

2.3. Outcomes

Effects, both positive and negative, in children who wear masks were the outcomes of interest, including effectiveness against animate and inanimate hazards as well as the social, psychological and physical effects according to the WHO biopsychosocial model.

2.4. Search Strategy

The comprehensive set of search terms was developed by experienced researchers according to the criteria defined in the PICO scheme. In particular, the interventions and outcomes are reflected in the search terms. The search terms are kept simple, broad and general in order to find as much data as possible. Following the clear research question according to PICO, we executed a systematic title and abstract-specific search using the PubMed/MEDLINE database to identify articles (reviews, case reports, original research, technical notes) related to face masks and children in relation to surgical, N95, and other mask types (as these have been mandated by law during the pandemic, e.g., in schools in many countries).
The time range of our search was from 1957 to 15 June 2024.
A specific age range was included as a selection criterion; our search included only data that were extracted from or related to individuals under 18 years of age.
The search strategy adopted for the scoping review is shown in the Supplement A, supplementary Table B.

2.5. Literature screening and data extraction

Articles selected for inclusion in the review were those that included information relating to the use of protective facemasks by children, including articles relating on the positive effects, the physiology of children, psychological and sociological aspects, physical symptoms, clinical conditions, toxicological aspects, as well as the protective efficacy against respiratory infectious agents. For further information regarding inclusion and exclusion criteria, see Supplementary Table C, Supplement A.
Two independent researchers identified and screened the eligible studies. If discrepancies arose during these process steps or if the authors disagreed, a senior author (the last author) was involved to achieve consensus. The selected papers were checked by all authors for final eligibility. Study design, methodology, primary and secondary outcomes were evaluated. Exclusions and reasons for them were documented.
Concerning the included studies, the following data were extracted into tables: Author and year, type of study, topic / aim, outcomes, sample size, main findings, sponsor and conclusions.

2.6. Synthesis of the evidence

Consistent with our PICO scheme [50] and also in the spirit of the adverse effects approach in reviews [47–49], we adopted a narrative approach. In implementing this approach, we also followed the reporting guideline “Synthesis Without Meta-analysis” (SWiM) in

reviews [51]. Our narrative approach involves thematic analysis where the studies were grouped based on the common theme of the effect of the mask, so that a coherent narrative emerges from the different data sources.
Studies were compiled according to the common contents associated with mask wearing. As it was a key argument in favour of mask use, the effectiveness against infection was taken into account. It has been one of the hoped-for effects. The negative and positive effects were categorized into psychological, physical and social according to the WHO biopsychosocial model. In addition, the social and political dimension was examined by looking at the claims made about masks.
The items were:

-Effectiveness of masks (1),
-positive effects (2),
-psychological and sociological effects (3),
-physical symptoms (4),
-physio-metabolic and toxicological effects (5) ,
and
-claims about masks (6).

The grouping decisions and groupings are presented in Supplement A (Table D).

2.7 Certainty of evidence assessment

We assessed the certainty of evidence for an association between mask use and the aforementioned items 1-6 evaluated as high, moderate, low or very low using an equivalent of the GRADE (Grading of Recommendations Assessment, Development and Evaluation) framework [52]. For this, we also assessed the bias of the included studies (high, medium, and low), comprising the funding-, observer- and overall bias and the methodological limitations of each study based on data provided also in the extraction tables and described in the discussion and summarised in the GRADE table, particularly in rows seven and eight there. The Cochrane Risk of Bias Tool RoB and RoB-2 as well as the Cochrane Critical Appraisal Skills Programme (CASP), were only partially applied as it was neither mandatory nor required for a scoping review [53]. Since we are not conducting a quantitative meta-analysis, but a qualitative narrative synthesis, we consider this reduced approach to be sufficient.
In any case, our certainty of evidence assessment goes beyond the methodology typically required for a scoping review and is closer to a systematic review (hybrid approach). We consider this to be extremely important for the specific topic of masks and children. Especially since we are following the Cochrane framework of adverse-effects approach [47–49].
Therefore, we have created a GRADE-equivalent evidence table and provided it in Supplement A.

2.8. Risk assessment

Our approach assesses potential risks to children´s safety by considering the most severe, yet plausible, scenarios of mask-wearing in which adverse outcomes may occur. Therefore, applying a worst-case scenario approach appears adequate here [36]. Both the European Union (EU) and United States (US) regulatory frameworks support the use of a worst-case risk assessment approach in the evaluation and management of medical interventions. The European Medicines Agency (EMA), Federal Drug Administration (FDA), and related agencies require to consider the most severe, though unlikely, adverse events when assessing safety risks, for medical interventions. The EU supports this approach through regulations like the EU Medical Device Regulation (MDR) including ISO 14971:2019, EMA guidelines, and Good Pharmacovigilance Practices (GVP) modules. Similarly, in the US, the FDA integrates worst-case risk assessments into their Risk Evaluation and Mitigation Strategies (REMS) also strongly aligning with and encouraging adherence the principles for risk management due to ISO 14971:2019 (Medical Device Risk Management), and clinical trial guidelines. Our approach, such as this regulatory guidance above, help ensure that the risks associated with medical interventions (here wearing masks by children) are properly identified and communicated to protect public health and human safety.
During the coronavirus pandemic - when the real and low mortality rates of approximately 0.07% for 0- to 69-year-olds and 0.0003% for children were unknown [54] – a similar approach with the precautionary principle [55] was also used to legitimize the pandemic measures. For example, the exaggeratedly assumed high risk of coronavirus infection and death was used as a justification for mask mandates [56]. Now that the mortality rates and the risk associated with the SARS-CoV2 virus are known to be drastically lower as formerly assumed, the risk-benefit assessment is shifting towards considering the risks and side effects of the interventions. Away from the overemphasis on the assumed protective but empirically unproven effect of masks.
The principle of using worst-case assumptions when evidence is weak is common in public health risk assessments [55]. This approach is not only justified but necessary in situations where uncertainty can lead to dire consequences.

For risk assessment, the GRADE table helps stakeholders understand the reliability of the evidence when making decisions. Uprating is particularly relevant in risk assessment because it allows observational data (common in public health and epidemiology) to be given more weight when the evidence for possible adverse effects is exceptionally compelling. In the context of adverse effects, the GRADE approach specifically permits uprating of evidence certainty [47,52,57]. Firstly, if there is a plausible (biological) mechanism for the adverse effect (e.g., transient hypoxaemia, transient hypercarbia, disturbance of non-verbal and verbal communication); secondly, if there are consistent findings from multiple studies showing similar adverse effects; and thirdly if evidence exists that adverse effects increase with increasing use and there is a large effect (e.g. mask

wearing duration, N95 versus surgical, etc.). For these reasons, when evaluating the mask use outcomes evidence (particularly for the psychological and sociological effects, physical symptoms, physio-metabolic and toxicological effects), we sometimes, in justified cases, rated studies that indicate potential risks and symptoms up. Because early symptoms or complaints may signal emerging health concerns that are or can become clinically relevant. Symptoms can already appear before morphologically and laboratory detectable progressive and serious damage such as decompensation, occurs, which is well known from clinical medicine and toxicology (prodromal symptoms, preclinical symptoms and early warning signs) [58,59].

In general, symptoms and complaints, even if supported by low levels of empirical evidence (e.g. from questionnaire studies), should generally be taken seriously. Indeed, questionnaire studies are subject to bias, such as recall bias, social desirability bias, and non-response bias, which can reduce their reliability. But in the absence of numerous randomized trials, real-world evidence (such as observational studies) can provide important insights, particularly for understanding the population level impact of interventions, including non-pharmaceutical interventions (NPIs) [60]. If the non-pharmaceutical intervention (NPI) is assumed to be less invasive, it may be justified to take symptoms more seriously, even if empirical evidence is limited. Raising the level of evidence may not always seem fully justified based on the classical framework of hierarchy of research design, but with careful clinical judgment, the risks of ignoring complaints and symptoms may outweigh the uncertainties associated with the low level of evidence. In evaluating the risks and adverse effects of an intervention, traditional high-quality RCTs may not provide sufficient safety data [61]. Funded high-impact RCTs often neglect safety reporting [62], particularly when focused on efficacy [63]. Therefore, when assessing harms, evidence from studies that prioritize safety reporting—even if methodologically less rigorous—should be uprated to ensure a comprehensive understanding of risks [47–49]. This approach aligns with the need for a balanced evidence base that addresses both efficacy and safety. We have therefore uprated the certainty of evidence based on the assessment of risks associated with the mask-wearing intervention in justified cases. However, we have always handled this transparently. For further details see Supplement A .

The principle of "*first, do no harm*" (*primum non nocere*) indeed necessitates a nuanced approach to evaluating evidence, particularly concerning adverse effects. On one hand, the threshold for proving the effectiveness of an intervention is usually higher due to the need to establish that benefits outweigh potential harms. For proving efficacy, we often look for high-quality, randomized controlled trials (RCTs) or systematic reviews of RCTs to show that an intervention like mask wearing works. On the other hand, for evaluating adverse effects of interventions, the threshold for accepting evidence might be lower because the implications of not recognizing a harmful effect could be catastrophic. Even lower-quality evidence like case reports or -series can be crucial in alerting to potential dangers, prompting further investigation or immediate action to protect users. The preference for and higher ranking of a study design should be matched to the scientific question at hand,

rather than adhering strictly to a hierarchy: For example, depending on the focus of medical science, prioritiation and uprating of study designs in terms of evidence can vary and even be opposite. While the approach of detecting and explaining side effects and risks of an intervention favours observational studies and case series, the approach of confirming/evaluating the effectiveness of an intervention favours RCTs [64].

Early detection of adverse effects, even with less certain evidence, aligns with the precautionary principle in medicine. If there's a signal that an intervention might cause harm, even with low certainty, it's often better to err on the side of caution to prevent harm. Indeed, it is entirely possible and often necessary to consider adverse effect evidence with a different lens than evidence for efficacy, particularly under the ethical "do no harm" principle. This doesn't mean lowering scientific standards but rather recognizing that in medicine, the implications of harm might justify acting on less certain evidence with the caveat that such evidence must be quickly followed by more definitive research with even higher standards.

3. Results

Of the 3130 total articles retrieved from medical literature and 19 additional records identified through other sources, 107 articles published in peer-reviewed journals directly or indirectly addressed the topic and thus serve as the database for this review (PRISMA flow diagram of the scoping review, Supplement B, Figure S1). These include 18 reviews (7 systematic reviews including one Cochrane review), 76 primary studies including randomised controlled trials, randomized clinical trials, cluster-randomized trials, observational studies, cross-sectional, cohort studies, case control studies, case series, surveys and modelling studies. Additionally, 13 communications (commentaries, statements, editorials, opinions and letters to the editor) are included.

We extracted the most important data from the 107 papers in tables.
The tables can be found in the Supplement B and contain the following topics :

-Evidence for (non-)effectiveness (14.9%, 16 papers, Supplement B: Table S1),
-Positive effects (12.2%, 13 papers, Supplement B: Table S2),
-Psychological and sociological effects (38.3%, 41 papers, Supplement B: Table S3),
-Physical symptoms, clinical conditions (7.5%, 8 papers, Supplement B: Table S4),
-Physio-metabolic and toxicological effects (21.5%, 23 papers, Supplement B: Table S5),
-Empirically and experimentally unproven claims – no ecological validity from a strictly empirical view (5.6%, 6 papers, Supplement B: Table S6).

The corresponding evidence certainty table equivalent to GRADE can be found in Supplement A (Table E). While the evidence for non-effectiveness of face masks against

viruses was predominantly medium to high, the evidence for their effectiveness was low to very low (Table E, F). The negative effects of masks, including the psychological, sociological, physiological and physical effects, as well as the physio-metabolic and toxicological effects, were demonstrated overall with moderate certainty of evidence (Table E, F). The claims made about masks during the pandemic proved to be very low in terms of evidence certainty (Table E, F).

4. Discussion

The results of the evaluation of the 107 publications included are discussed below (sections 4.1-4.6), based on the literature listed in the extraction tables (Supplement B: Tables S1-S6). In addition, we added an important chapter on microbiological mask contamination (4.7). Finally, the important topic of risk-benefit analysis (4.8.) is discussed, which can serve as a basis for decisions on mask obligations in children. This section also contains the summary of the scientific findings on masks, which are compared with our findings and discussed together, as well as a final risk assessment (4.8.5).

4.1. Evidence for (non-) effectiveness

The literature evaluation reveals that there are some studies claiming to show the effectiveness of masks (with low to very low certainty of evidence), but the majority of studies clearly prove that face masks are not effective against viruses and impress with their better quality and good design, with significantly higher certainty of evidence from medium to high (Supplement A: Table E and F, Supplement B: Table S1).

Evidence for effectiveness

In a cross-sectional study aiming to provide descriptive data on infection risks as reflected by infection prevalence, on potential pathways and risk factors as well as on manifestations in young children, undiagnosed infections were found in 12.5% of kindergarten children and 68.4 % of them never wore a facemask. The self-reported neglect of wearing a facemask was associated with preceding infection, supporting a protective effect [65]. Weaknesses of this study are the weak endpoint of self-report and the uncertain exclusion of further protective measures due to unclear contextual factors such as family of origin and hygiene measures within these families.

The results of a retrospective cohort study with the aim to assess if wearing face masks in the classroom is a useful mitigation measure to control SARS-CoV-2 infections showed that transmission can be reduced in school classes by mandatory FFP-2 mask use with

only a small difference in cumulative SARS-CoV-2 infection rates [66]. In many cases, however, infection appears to be postponed rather than avoided.
The authors´ conclusion was that infections with SARS-CoV-2 are delayed, but they cannot be prevented in the long run by wearing face masks [66]. However, the endpoint was a comparison of cumulative SARS-CoV-2 infection rates in sports classes (with limited use of face masks) and non-sports classes (with the consequent use of face masks).

A cross-sectional study to assess potential risk factors for the COVID-19 seroprevalence in children aged <18 years who visited a hospital in Somalia, evaluated COVID-19 Ag Rapid Test lateral flow immunoassay kit results, clinical characteristics, as well as a preventive practices questionnaire [67]. The majority of participants with positive antibodies against SARS-CoV-2 were school-aged children and children, who did not wear facemasks, as well as those who had close contact with infected adults among others. However, in that study no separation of diverse measures was established. Correlation is not causality and the detection of antibodies is not principally linked to an undesirable condition, e.g. herd immunity. Interestingly, 46.9% of the COVID-19–positive children were asymptomatic without any clinical signs of the disease [67].

A research-based commentary to examine the impact of non-pharmaceutical interventions (NPIs) on the resurgence of other respiratory viruses found lockdowns imposed during the pandemic in Europe, Australia, and New Zealand have resulted in a sizeable group of susceptible young children who lack pre-existing immunity because of reduced exposure during colder months [68]. However, the assumptions of masks' effectiveness are based on the modelling of mask wearing and infection rates and thus have to be viewed critically.

A systematic review to provide an assessment of the evidence on the effectiveness of measures implemented in the school setting to keep schools open safely during the COVID-19 pandemic found low-certainty evidence from 7 studies that there may have been a beneficial effect of mask mandates on transmission-related outcomes [69]. However, the authors concluded, that further high-quality research into school measures is needed to develop a more evidence-based understanding [69].

A systematic review (with narrative synthesis) to evaluate the effectiveness of face masks in reducing COVID-19 incidence in school settings found four studies that showed no difference or even worse results between mask and control, but 10 out of 14 included studies indicated that mask use could reduce COVID-19 incidence in schools [70]. As no randomised controlled trial could be identified, only 12 observational studies have been included and their overall risk of bias was moderate to serious. Weakening the conclusion are different methodologies adopted in included studies and the lack of randomization as well as no clear separation of mask wearing from other contextual factors and measures implemented at the same time.

Evidence for non-effectiveness

A survey to assess the accuracy of face mask wearing by children and adolescents in different school situations showed that masks were worn correctly in only 63.7% of the time in the presence of a teacher, but this percentage decreased to 31.9% when no teacher was present [71]. Students reported mouth and nose uncovered as being dominant, followed by mouth covered, nose uncovered variant. So even if the mask had an effect, incorrect use by children would jeopardize its effectiveness.

Correspondingly, an observational study to ascertain the prevalence of face mask usage and the factors affecting the same found only 24.5% of children using the mask appropriately, and 33% of children did not wear a mask at all [72].

Many studies show that masks used by children are not effective against viruses.

A systematic review with the aim to evaluate the body of literature on mask wearing in children to assess the existing evidence regarding protection offered by face masks against SARS-CoV-2 infection or transmission with 22 studies included showed that real-world effectiveness of child mask mandates against SARS-CoV-2 transmission or infection could not be demonstrated with high-quality evidence [24]. Sandlund et al. concluded, that the body of scientific data does not support masking children for protection against COVID-19.

In an editorial the evidence and harms of masks in children against COVID-19 were discussed. There is absence of evidence for masking children against COVID-19. Studies showing a protective effect of face masks were more likely to be published than negative studies [73]. Children are less affected by the virus. Mask use reduces focus from other measures that may be more important. The conclusion was no recommendation for masking children, as most of the children with COVID-19 get few symptoms and do not seem to drive the mortality of the pandemic [73].

In a research which aimed to build on an observational study by the Centers for Disease Control (CDC) that demonstrated a link between school mask mandates and reduced paediatric COVID-19 cases, it was investigated whether this relationship holds in a larger, nationally representative dataset over an extended time-frame [25]. Interestingly, after nine weeks, the case rate per 100,000 was 18.3 in counties with mask mandates, compared to 15.8 in counties without them ($p = 0.12$). In a larger sample of 1,832 counties, between weeks 2 and 9, the case rate per 100,000 people decreased by 38.2 in counties with mask mandates and by 37.9 in counties without them ($p = 0.93$). Thus, in the extended sample, the link between school mask mandates and case numbers was not sustained [25]. The author´s conclusion was that observational studies of interventions are subject to various biases and do not offer enough evidence to support the recommendation of mask mandates.

An observational study aimed to evaluate the effectiveness of mandatory mask use in 599314 children with quasi-experimental comparison between 5-year-old children, as a control group, and 6-year-old children, as an interventional group [74]. Mask wearing vs no mask was compared with help of incidence of SARS-CoV-2, secondary attack rates (SAR) and the effective reproductive number (R*). The results show, that with mandatory use of face masks, the children have a significantly higher transmission indicator [74]. Altogether, mask mandates in schools were not associated with lower SARS-CoV-2 incidence or transmission.

A narrative review which critically examined the United States' decision to mask children as young as two for COVID-19 concluded that considering the best available high-quality evidence on mask effectiveness alongside potential harms, the practice of masking children appears increasingly unfavourable [20]. With an increasing number of high-quality studies showing no evidence of benefit and an increasing number of studies reporting adverse effects on children, justifying the CDC's mandate for masking children has become more difficult. From the outset, the best available evidence has never supported a clear net benefit of masking very young children [20].

An observational study to compare COVID-19 incidence among over one million 10–12-year-olds between cities with different recommendations on the use of face masks in schools found the highest incidence rates in winter months in the school mask mandated cities [75]. The authors conclusion was that face mask recommendations in schools did not reduce COVID-19 incidence among 10–12-year-olds in Finland [75].

One review emphasised that face masking for children should be reconsidered [15]. The author discussed, that there are no randomised controlled trials - the gold standard for determining the effectiveness of facemasks on children. Despite having the lowest risk of severe disease from SARS-CoV-2 infection, children have endured the most disproportionate disruption to their lives during their most formative years. The conclusion was, that there is no robust evidence to recommend face masks for children, and a need to return to other first principles and focus on evidence-based interventions that help protect children [15].

In a Cochrane systematic review to assess the effectiveness of physical interventions (including masks) to interrupt or reduce the spread of acute respiratory viruses the pooled results of RCTs did not show a clear reduction in respiratory viral infection with the use of medical/surgical masks [26]. This was consistent with 3 studies of 43 included regarding masks and children. Jefferson et al. concluded, that there is uncertainty about the effectiveness of face masks in the elderly and in young children [26]. Altogether, harms associated with physical interventions were under-investigated.

For the most part, masks show no empirically proven effectiveness in studies.

The certainty of evidence assessment shows low to very low evidence quality for SARS-CoV2 effectiveness of face masks in children (Supplement A, Table E and F).
The studies that attempt to prove effectiveness have weak endpoints, e.g. self-reports and the uncertain exclusion of further protective measures, unclear contextual factors, modelling of mask wearing and infection rates, and many authors interpret correlation as causality [35]. In contrast, the studies that refute the antiviral efficacy of the masks are convincing due to the design and the higher-quality data collection. The certainty of evidence assessment shows predominantly moderate to high evidence quality for their conclusion of non-effectiveness of face masks in children (Supplement A, Table E and F). One of the reasons for this better quality of mask critical papers could be that publications critical of the pandemic measures only got through peer review with considerable effort and were only approved for publication under massive methodological scrutiny, while studies representing the ubiquitous opinion got through peer review very easily. This publication bias is known from the pandemic [76,77].
Further high-quality research is needed to develop a more evidence-based understanding.

4.2. Positive effects

There is literature reporting positive mask effects on children (Supplement B: Table S2). However, this must be viewed and discussed in a differentiated manner, as there is an overall very low evidence certainty for it (Supplement A: Table E and F).

A review of health effects of wildfire smoke and public health response aimed at children including masks showed that N95 respirators are able to decrease outdoor wildfire particle exposure by ~80% and surgical masks by ~20% [78]. These benefits would only be expected for short durations. The evaluated studies generally support the idea that children could see benefits from respirators in wildfire smoke scenarios, despite the concern that fit may be difficult due to more variation in facial sizes [78]. However, the false sense of security and the possible adverse physiologic effects have to be considered. The authors of the paper remind that there is a research gap regarding mask safety in children [78].

An analysis of the monthly incidence rates of invasive pneumococcal disease (IPD) and the distribution of serotypes in high-risk age groups between 2020 and 2021 during non-pharmaceutical interventions (NPIs) like face masking, enhanced hand hygiene, social distancing, international travel controls, effective quarantine and contact-tracing policy to control COVID-19 found a significant reduction in IPD incidence rates among children. However, no separation of face masks- and other measures was established [79].

In a cross-sectional study with the aim to evaluate the additional benefits of non-pharmaceutical interventions (NPIs) like border restrictions, quarantine and isolation, community management, social distancing, face mask usage, and personal hygiene against

COVID-19 on notifiable infectious diseases (NIDs) except COVID-19 the largest reduction (82.1%) was found for children aged 0–14 years [80]. The authors´ conclusion was, that non-pharmaceutical interventions (NPIs) aimed at COVID-19 prevention may significantly benefit the prevention of other infectious diseases [80]. However, no separation between the different measures (border restrictions, quarantine and isolation, community management, social distancing, face mask usage, and personal hygiene) was established [80].

A 2011 study to investigate efficacy, acceptability, and tolerability of face masks and hand hygiene in households with influenza index patients showed that participants of households which fully implemented the intervention within 36 hours of symptom onset had a significantly lower risk of influenza infection. The authors´ conclusion was, that non-pharmaceutical interventions may be effective in preventing influenza transmission in households also including children. However, the authors did not ensure a clear separation of diverse confounders [81]a separate consideration of children and adults [68].

A cluster-randomized trial from the same research group with the aim to monitor adherence and tolerability of face masks and intensified hand hygiene to prevent influenza infections in households which objectified the frequency of wearing face masks and frequency of hand disinfection showed that children accepted wearing masks – even when ill – at a rate similar to that of adults [82]. However, also this study did not ensure a clear separation of diverse confounders and a separate consideration of children and adults.

In a randomized clinical trial to evaluate the effect of wearing a face mask on hand-to-face contact by children while in a simulated school attendance, the rate of hand-to-mucosa contacts was significantly lower in the mask group, while the rate of hand-to-non-mucosa contacts was higher [83]. Therefore, the rate of hand-to-face contacts did not differ significantly between the mask and the control groups [83]. But as face touching was higher in the mask group (rate of hand-to-non-mucosa contacts) potentially the positive effect of lower mucosa contact rate was possibly neutralised.

In a modelling study a mathematical transmission model was used to investigate the impact of various interventions (natural ventilation, face masks, high efficiency particulate air (HEPA) filtration, and their combinations) on the concentration of virus particles in a classroom with one infectious individual [84]. The outcome was the cumulative dose of viruses absorbed by exposed occupants. In the hypothetical calculations surgical face masks showed significant effectiveness regardless of the season, with an 8-fold decrease in transmission [84]. Combined interventions (such as natural ventilation, masks, and HEPA filtration) were the most effective in the calculation, resulting in a 25-fold decrease and maintaining high effectiveness even in the presence of a super-spreader. However, the results of this modelling study should be interpreted with caution as it does not represent empirical experimental evidence.

An observational study evaluated the effect of NPI measures (hand washing, mandatory use of face masks for children older than 5 years, social distancing) and their influence on the circulation of respiratory viruses among children before and during the SARS-CoV-2 pandemic [85]. A notable effectiveness of NPIs (hand washing, mandatory use of face masks for children older than 5 years, social distancing) in lowering respiratory virus transmission among children was shown, even when not specifically targeted at preschool-aged children [85]. Rhinovirus, RSV, HMPV, and influenza showed particularly favourable responses to these interventions [85]. Unfortunately, no separation of face masks and other measures could be ensured. It is unclear what role the masks alone played among the many other measures. An isolated positive evaluation of masks is therefore not permissible.

Another study aimed to evaluate the transmission of SARS-CoV-2 in schools and their association with infection control measures using a multiple-measurement approach [86]. Outcomes were SARS-CoV-2 in aerosols in the air and saliva samples from the students. Daily average aerosol number concentrations decreased by 69% with mask mandates and 39% with air cleaners [86]. Compared to no intervention, the transmission risk was lower with mask mandates and comparable with air cleaners. The authors concluded from their results, that mask mandates were linked to more significant reductions in aerosol concentrations and lower transmission rates compared to air cleaners [86]. However, the results of this study should be interpreted with caution. The authors modelled disease transmission using a semi-mechanistic Bayesian hierarchical model, adjusting for absent students and community transmission (probabilistic simulation approach) [86]. Furthermore, an airborne detection of pathogens document exposure but not necessarily transmission [86]. The absence of hard endpoints such as infection and disease does not allow for any empirically relevant conclusions.

A survey to evaluate the impact of non-pharmaceutical interventions (NPIs) on behaviour and influenza infection among children in a metropolitan area comparing two periods (pre-COVID-19 and the 2020–2021 season during the COVID-19 pandemic) revealed that among children who simultaneously washed their hands and wore face masks, there was a significant reduction in influenza infections [87]. However, in the evaluations no separation of face masks and other measures was ensured.

In a prospective observational study aiming to track the progression of COVID-19 infections in schools and preschools and identify the factors (hygiene measures including keeping a distance with at least 1.5 m, proper coughing, sneezing and thorough hand washing, wearing a mask and frequent ventilation) affecting the severity of outbreaks, the teacher/caregiver mask obligation and the face mask obligation for children showed a significant reduction in the number of secondary cases with small effect sizes [88]. However, as no separation of face masks and other measures was ensured, no valid conclusions regarding masks could be made.

A cross-sectional analysis of a longitudinal study with the aim to measure SARS-CoV-2 infections and seroactivity in 24 randomly selected school classes and connected households found that the prevalence increased with inconsistent facemask-use in school. However, mask wearing was recorded by self-reporting and there was no methodically safe separation of mask wearing from other measures such as distancing and hygiene rules [89].

In a survey of school-aged children and adolescents during the COVID-19 pandemic, evaluating the self-reported psychological distress status, students who rarely wore face masks had significant higher odds of self-reported psychological distress [90]. The frequency of wearing a face mask and time spent exercising apparently had a protective association for mental health [90]. Students who wear face masks frequently, might have the feeling to less likely contract COVID-19, which could further reduce worry and anxiety levels and promote mental well-being. Therefore, the authors concluded, that wearing a mask may be more conducive to mental health [90]. However, such a positive mask effect should be interpreted with caution. A more distressed person might be less inclined to take self-protective steps. Students with pre-existing mental health problems may pay less attention to COVID-19 and therefore be less likely to be concerned about mask use.

Altogether, many studies reporting positively on masks against viruses in children did not separate the covering of mouth and nose from other more effective measures like hand washing and others [79–82,85,87–89].

Indeed, for short application periods, when justified by a specific risk scenario, e.g. during wildfire smoke, the use of face mask in children can have positive effects [78].
The use of a mask as an infection control measure in a healthcare setting to reduce the transmission of Mycobacterium tuberculosis also appears to be justifiable [91], even if masks are only the third level of the infection prevention and control hierarchy [92]. However, other scenarios like masking children for COVID-19 appear hypothetical and not proven by empirical evidence [35]. due to low to very low evidence certainty (Supplement A: Table E and F).

4.3. Psychological and sociological effects

The literature shows clear adverse psychological and sociological mask effects for children (Supplement B: Table S3). And the overall certainty of the evidence for this is predominantly moderate (Supplement A: Table E and F).

Children were often nudged to wear masks during the COVID-19 pandemic [22]. Correspondingly in interview questions regarding mask-wearing, caregivers' perceptions of masks seemed to influence children's attitudes [93]. But health perceptions regarding wearing a facemask to prevent exposure differ between children and their caregivers [32].

There has been a clear association between government mask mandates and mask usage among children during the pandemic [4].

Of course, habituation effects, graduated exposure, prompts, and differential reinforcement, it was possible to induce tolerance to mask-wearing, even in children with autism spectrum disorder for short periods of time [94,95]. Among all children, these had particular problems with mask wearing [96].

Despite some studies showing that large amounts of school children considered mask use to be useful [97,98], a majority of parents did not view masking young children as an appropriate COVID-19 prevention measure [99]. And children of all ages above 6 years say they were usually (80.9%) embarrassed by the mask [100]. Among the most prominent words in the free-list of words associated with masking belong also "can't breathe", "uncomfortable" and "unnecessary [98].

Some authors are emphasising human rights in addition to the isolated consideration of epidemiological and physical safety issues in child mask mandates [16]. Even during the pandemic, there were warnings to reconsider wearing masks for children [15].
In retrospect, there is no doubt that mask-wearing, enforced by law, had detrimental psychological and sociological consequences on all children around the world and not only in Europe [101].

Indeed, masks are a barrier to communication and may negatively affect communication and education [102]. They degrade the speech signal, serving as a low-pass filter by attenuating high frequencies spoken by the wearer: the decibel level of attenuation ranges from 3 to 4 dB for surgical masks and close to 12 dB for N95 masks [103].

Experiments prove that the face mask negatively affects pre-schoolers' speech recognition in a realistic classroom environment [104]. Also in 7–18-year old children, both normal and hearing impaired, the combination of noise and face masks adversely affects children's ability to understand speech [105]. In line with this, a speech reception measurements study found that speaking while wearing a face mask with background noise leads to a distinct impairment of speech intelligibility [106]. School-age children, and young adults, are negatively affected by face masks when recognising speech [107],

Masks can also obscure social cues provided through facial expressions.
According to a developmental psychology and socialization review, for perceptual skills, infants progressively learn to interpret the eyes or gaze direction within the context of the entire facial configuration [108]. This process aids in identity recognition and emotional expression discrimination. In terms of socio-communicative skills, direct gaze and emotional facial expressions are vital for engaging attention, while eye-gaze cuing is crucial for joint attention. Additionally, focusing on the mouth is particularly important for speech learning [108].

Undoubtedly, masks are capable to hinder this development by covering large parts of the face. In a randomized experimental study, the use of face masks resulted in a significant decline in face perception abilities. The holistic processing, which is a key aspect of face perception, was impaired for masked faces [109].

The most frequently reported short- and medium-term direct effects of mask-wearing during the pandemic affecting the well-being, behaviour, and psychosocial development of children and adolescents included reduced communication due to diminished auditory comprehension and facial expression visibility [110]. These communication limitations impacted social interaction and the quality of education. Additionally, there was an increase in psychosomatic complaints, anxiety, depression, and eating disorders [110].

In a standardized verbal-response test study it could be shown, that face masks' use influenced emotion inference from faces for all ages and especially for toddlers [111]. The conclusion was, that face masks may potentially affect the emotional understanding or developing social and interaction skills in children, such as in education, especially for those suffering from sensory or cognitive deficits.

However, according to some authors, face masks did not significantly impair basic language processing ability [112,113], also in bilingual children [114], but they had a significant effect on the children's emotion recognition accuracy [112].

As masked angry faces are more easily recognized and masked happy and sad faces less easily recognized [112], a distorted perception of emotions and a deprivation of positive influences during the pandemic have been made possible. Experiments proved, that covering faces with masks was associated with poorer recognition of emotions, particularly for happy, sad, and fearful expressions compared to angry and neutral ones [115]. Matching these findings, a group of researchers found that masks impair the recognition of sadness and happiness, also of anger, which was, however, somewhat easier to recognise [116]. The longer the mask exposure lasted, the better the initially poorer emotion recognition was [116]. In emotion categorization and emotion intensity rating tasks the accuracy of emotion recognition in children was impaired for various facial expressions (disgust, fear, happy, neutral, sad, and surprise faces), except for angry faces [117]. Interestingly, negative side effects of mask-wearing on reading emotions were observed for more facial expressions in children than in adults; transparent masks could help remedy these [117]. A researcher group found also that the presence of a mask did affect the recognition of sad or fearful faces by children (no positive emotions like happy faces, smiling, were evaluated), but did not influence significantly the perception of angry and neutral faces [118]. These results are in line with the fact that cues in the mouth region of the face seem crucial for the recognition of happiness [119–122], while cues in the eye region seem crucial for the recognition of anger [120,121,123,124]. For recognising sadness, the findings are less coherent, with studies on adults finding the eye region to be essential [119,120,122], whereas for children the mouth region may be more essential [121]. Recent

studies examining the impact of face masks on adults' emotion recognition in children have also consistently shown that anger is the least impaired emotion by masks [125–127]. Fittingly, in an observational study, children were better able to identify emotions in an unmasked adult and when the masked model explicitly stated or implied the emotion [128].

A systematic review finds out that children and their teachers experienced difficulties in processing facial expressions due to mask-wearing. Also stress and anxiety, along with concentration and learning challenges was associated with mask use [129]. Eighteen-month-old infants who were exposed to masks on a daily basis since birth could fast-map new words and generalize these words across people and objects of the same category when words were taught with or without a mask. However, those infants could flexibly rely more on the eyes to achieve fast-mapping and word generalization when the speaker was masked [130].

Interestingly, older students were more likely than younger students to report that wearing masks hindered their ability to interact with peers and understand the teacher [131]. Some authors state how face masks can affect school performance and complain, that the child cannot access visual cues because the speaker's face is hidden, preventing lip reading [132]. Communication difficulties associated with facemask use are evident and surgical masks affect speech perception compared to other non-transparent mask types, also emotion recognition is less accurate when wearing facemasks [133].

In an investigation of the acoustic and visual effects of face masks on speech intelligibility, children made more mistakes and responded more slowly when listening to face mask speech compared to speech produced without a face mask [134].

As a compensation, during COVID-19, teachers spoke more words per minute than those observed before the pandemic, but their vocalizations included fewer unique phonemes [135]. Fittingly, during the pandemic, children exposed to a higher number of words per minute from teachers produced more speech-related vocalizations themselves [135].
A randomised controlled trial found that wearing face masks has tendencies to a slightly worse cognitive performance in the group of mask wearers [136]. However, the authors concluded, that wearing face masks has no significant influence on attention and executive functions of pupils during two school lessons [136]. This conclusion should be seen very critically, as the experimental trial was performed without an appropriate control group and the time period for recovery without a mask worn of 15 minutes appear too short from a physiological, toxicological or psychological point of view. Additionally, the trial results were obtained within a relatively small time of evaluation (two school lessons instead of an entire school day), in addition with inconsistent age groups [136]. Moreover, it has to be considered, that all included evaluated children were already chronically adapted to mask wearing and experienced already chronic mask effects [137,138], due to the pandemic which had been taking place for more than 2 years at the time of the trial, as

well as due to the two preceding school lessons with face masks on before the trial evaluations.

Many studies show detrimental mask effects, among others a very sophisticated evaluation of cognitive performance of adult chess players wearing masks showing significant decreased quality of players decisions [139], therefore the claim that masks have no effect on cognitive and school performance appears not to be reliable. The single factor of higher CO_2 exposure under masks of 1.41-3.7% [27,140] is likely to lead to cognitive impairment [27,141]. While the effects of short-term exposure on cognitive performance start at 0.1% CO_2 and manifest as reduced cognitive performance, impaired decision-making and reduced speed of cognitive solutions, many other long-term effects are known at concentrations above 0.5% [27,141–145]. Correspondingly numerous studies examining diverse mask types in adults point to a reduced ability to think and concentrate [146–154].

Taking into account the literature, an impairment of school lessons not only due to acoustic and visual impairments as mentioned above, but also due to e.g. carbon dioxide breathing or oxygen depletion while wearing a mask appears comprehensible (see sections 4.5. and 4.8.4.).

A systematic review reminds that children faced various challenges while using face masks, besides the difficulty interpreting facial expressions, such as physical discomfort, also due to poor fit and heat [155]. The main complaints about the facemasks in this regard were being too hot, too hard to breathe through and difficult to adjust [156]. In a cluster randomized controlled trial the primary problem associated with wearing a facemask cited by children was "heat/humidity" in 53%, followed by "pain" and "shortness of breath" [81].

Additionally, the wearing of face masks by physicians results in higher anxiety levels in children mirrored by higher mean heart rates [157]. Moreover, masks can muffle speech and cause medical comprehension errors for both paediatric patients and physicians [158]. All in all, the masks had a negative effect on the perception of positive signals and at the same time caused stress, learning and developmental disabilities in the children during the pandemic.

4.4. Physical symptoms

The literature shows clear adverse clinical mask effects with physical symptoms and clinical conditions for children (Supplement B: Table S4).
The certainty of evidence for these effects is predominantly moderate to high (Supplement A: Table E).

In a survey, 44.9% of children using face masks reported negative consequences: Respiratory discomfort/breathing difficulty were found in 33.99% besides cutaneous adverse effects up to 16.3 % (itch symptoms, rash, pressure effects and acne) [159].

The main symptoms of mask wearing reported in another survey were headache (49.0%), speaking difficulties (45%), change in mood (45.2%) and breathing discomfort (28.1%) [100]. The paediatricians reported fog on glasses (68.2%), breathing discomfort (53.1%), cutaneous disorders (42.4%) and headaches (38.2%) [100].

Alike, 68% of children complained about impairments caused by wearing masks. Reported side effects include irritability (60%), headaches (53%), difficulty concentrating (50%), reduced happiness (49%), reluctance to attend school or kindergarten (44%), discomfort (42%), learning difficulties (38%), and drowsiness/fatigue (37%) in a large registry [160].

About half of the children patients with psoriasis reported worsening of their symptoms, and one third of the children reported difficulties due to wearing a mask [161].

Moreover, mask wearing in children might be responsible for an increased presentation of facial pityriasis versicolor due to a humid environment and associated sweating [162].

Besides skin problems, there is the potential of the face masks causing ocular surface injuries, especially in children. Corneal abrasions while wearing adult face masks in children have been reported due to increasingly higher chances of direct trauma to the eyes as these masks are loose and the hard ends of the upper margin are raised to eye level [163].

Regarding the ear, the elastic loops cause constant pressure on the skin and on the cartilage of the auricle, which leads to lesions in the retro-auricular region if the masks are worn for many hours a day. Pre-adolescent children have undeveloped auricular cartilage with less resistance to deformation; prolonged pressure from the elastic loops of the mask at the hollow or, even worse, at the anthelix level can influence the correct growth and angulation of the outer ear [164].

Typical ear injuries or pressure injuries on the ear have been additionally mentioned in the literature regarding prolonged mask usage [165].

Jefferson et al already stated in their Cochrane systematic review, that harms associated with mask use are under-investigated [26].

Altogether, further research and analysis of the physical stress and damage caused by the pandemic measures, including masks on children, is of urgent necessity.

Undisputedly, the documented symptoms while wearing a mask by children (Figure 1) contradict the WHO definition of health [166] .

Figure 1. Frequency of selected symptoms in children wearing masks as determined in scientific studies (in per cent). Results from cross sectional-, online registry- and survey studies from the years 2021-2022 showing a very high number of adverse effects of face masks.

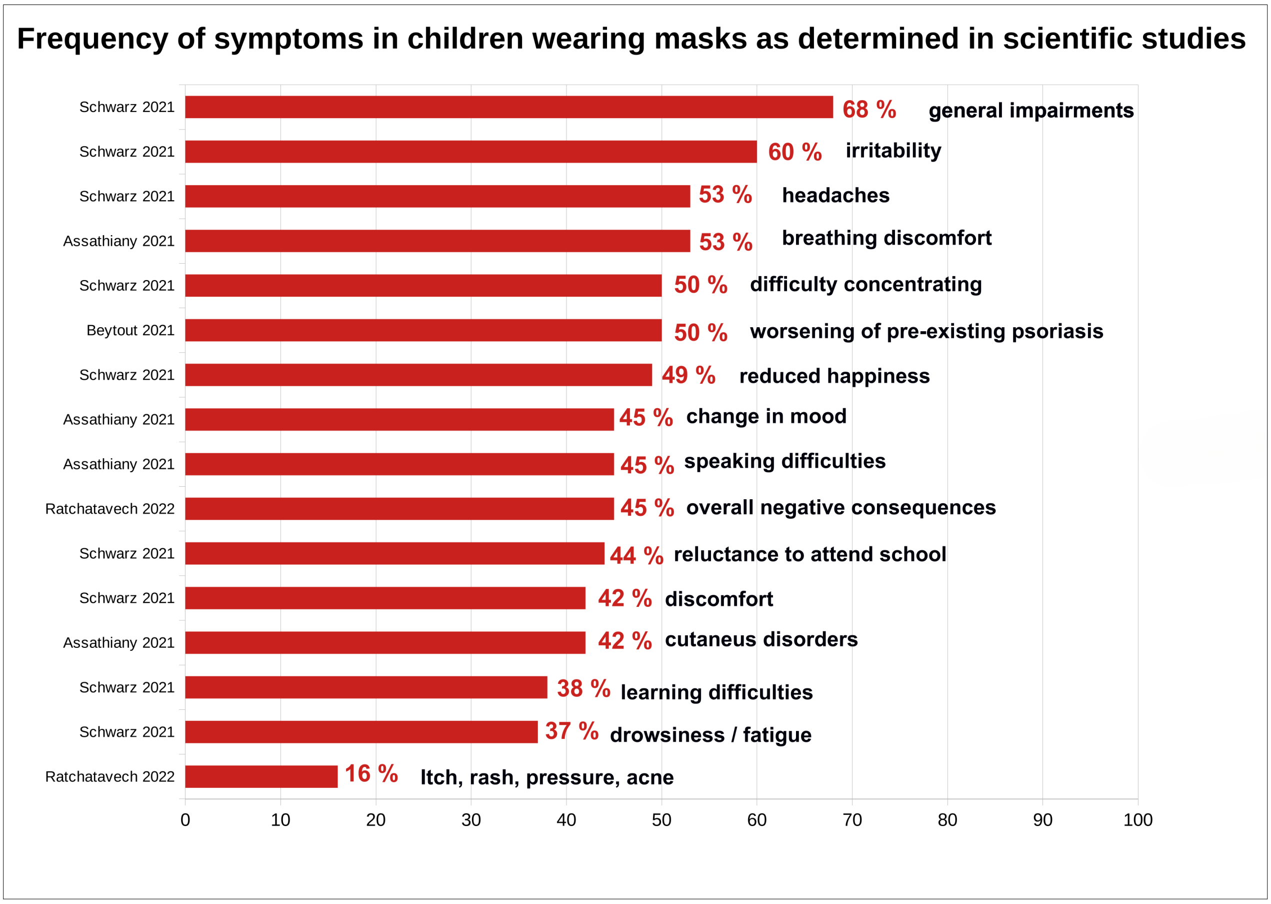

4.5. Physio-metabolic and toxicological effects

The literature shows clear, disturbing physio-metabolic and toxicological mask effects on children (Supplement B: Table S5). The certainty of evidence for these effects is predominantly moderate to high (Supplement A: Table E).

A narrative review states that face masks increase breathing difficulties and carbon dioxide in children [43]. In some children, anxiety and hyperventilation mimicking asthma while wearing a mask was observed [167]. Roberge already stated in his 2011 review, that mask use (surgical, N95-respirators) by children has some safety issues including proper use, fit problems and a lack of tolerance [42]. Due to higher breathing resistance, dead space volume and elevated inspiratory CO_2 levels he called for further research to address such face mask issues [42].

A communication at the beginning of the pandemic describes, that several students without documented cardiac issues experienced sudden death when they were running in

physical training classes with surgical masks or N95 masks in China [168]. The authors argued, that although a rarity, this risk could be higher especially in children with existing cardiac co-morbidities [168].

Contrary, some newer studies with a very short mask evaluation time, e.g. one minute, did not find significant physiological changes while wearing a mask [169].
Six minutes evaluations of surgical facemasks in children with exercise-induced symptoms (EIS) during sub-maximal treadmill exercise testing did not show evidence of acute gas exchange restrictions with pathological fall in oxygen saturation SpO_2 and pathological rise in carbon dioxide tension [170].

During another short evaluation of 6 minutes, the use of masks did not impact SpO_2 levels in children with or without asthma, whether at rest or during low-to-moderate intensity exercise [171].
The short and unrepresentative observation periods are noteworthy, so the transferability of the results to everyday situations remains questionable.

It is also questionable, whether the 12-minute results from a prospective randomised cross-over study of boys from a top-tier professional football club can be used to conclude that masks are safe for all children in the general population including girls [172].
However, in the maximum load range the authors of this paper found a significant reduction in the total running time and a significant increased subjective stress perception due to changed breathing pattern and increased airway resistance [172].
One needs to remain sceptical regarding the ecological validity of many such studies with very short examination times of the masks [169–173]. Although many authors of such studies conclude that mask use is harmless to children, some of their results indicate potential clinical consequences. For example, the perceived exertion and breathing effort was higher with the mask [169] and the performance decreased, while stress levels increased [172].
In an observational study on healthy children aged 8–10 years, during 10 test minutes the end-tidal values for CO_2 increased significantly and the end-tidal values for O_2 decreased significantly. However, since levels of hypercapnia or hypoxia were not reached in their experiments, the authors' conclusion was that physical exercise lessons with masks on should be recommended due to the minor physiological effects of wearing masks [173].
But, as far as the phenomena of transient hypoxia and transient hypercarbia are concerned, these do not need to exceed the standard values to be clinically relevant [27,29,34], see section 4.8.4, oxygen drop and carbon dioxide rise.

The breath isoprene concentrations significantly decreased throughout mask wearing experiments by Sukul et al [34]. Sukul et al recently revealed the origin of breath isoprene in humans via multi-omic investigations. Isoprene is derived from human muscular lipolytic cholesterol metabolism [174]. Thus, the decrease in breath isoprene during mask wearing is due to sympathetic vasoconstriction (deoxygenation- and hypercarbidaemia

induced) in muscle compartments [34]. This bad compensation during mask wearing triggered by the phenomena of transient hypoxia and transient hypercarbia apparently causes a shift of the blood stream towards vital organs such as the brain at the expense of the gut and skeletal muscles, which are thus disadvantaged. Undoubtedly, there is a need for research into further consequences.

In children, besides impaired breathing and gas exchange there is a measurable significant effect on the cardiovascular system. Despite no significant changes in the heart rate or oxygen saturation (SpO_2), during 10 minutes, surgical mask wearing caused a significant rise in tricuspid regurgitation, pulmonary regurgitation and pulmonary artery systolic pressure in 110 children [175].

On the one hand, a research group found that wearing surgical face masks for 30 minutes was not associated with changes in respiratory parameters or clinical signs of respiratory distress [176]. But on the other, the same group found significant increases in respiratory rate and partial pressure of end-tidal carbon dioxide ($PETCO_2$) in children wearing a N95 mask [177]. And in yet another experiment, significant decreases in oxygenation after 30 minutes and during the 12 minutes walking test were found [178]. These results show, that the use of a typical N95 mask causes a significant blood oxygen saturation drop [178] and a significant carbon dioxide rise in children, particularly during physical activity [177]. The same research group found, that there was a significant correlation between carbon dioxide rise ($PETCO_2$) and body mass index [179]. Overweight or obese children who wear a mask are more prone to developing respiratory distress, which causes them to remove it frequently [179].

In a short evaluation time of 15 minutes the wearing of surgical masks results in a statistically significant rise in inspired CO_2 and a smaller rise in end-tidal CO_2 ($ETCO_2$) [180].

Wearing a mask for 15 minutes increased the $ETCO_2$ when at rest and with brisk walking in children [181]. Seven percent of the children in that study indicated that they experienced mild breathing difficulty [181].

Ten minutes wearing a FFP2 or surgical mask showed a significant increase in inspiratory and expiratory CO_2 values [182].

Not only surgical and N95 masks, but also two-layered cotton tissue face masks showed physiological effects with increased pulse rate and reduced arterial blood oxygen saturation already within 5 minutes of wearing [183].

In an observational pilot study by Martellucci et al., after only 5 minutes of wearing a mask, the mean CO_2 detected inside the mask breathing zone in children was significantly elevated, being 7091 ppm (0.7% CO_2) in a surgical mask, and 13665 ppm (1.37% CO_2) in FFP2 respirators [30]. The CO_2 concentration was significantly higher among children

compared to adults and also in the subjects with high respiratory rates. The occupational exposure limit threshold of 5000 ppm (0.5% CO_2) was largely exceeded in children wearing surgical masks and in all age classes when wearing FFP2 respirators [30].

Fittingly, in another experimental study by Walach et al. nine minutes mask wearing by children significantly resulted in 13100 ppm (1.3%) carbon dioxide (CO_2) in the inhaled air under surgical masks and 13900 ppm (1.39% CO_2) under FFP2 masks in inhaled air which is by a factor 6 higher than the "inacceptable" 2000 ppm air limit by the German Federal Environmental Office [31]. The authors of the study stated, that decision makers and law courts should take this into consideration when establishing rules and guidance to fight infections (Figure 2).

Figure 2. Bar charts showing the excess carbon dioxide in the breathing air when children wear masks (mean/median values). The ambient air (no mask) is given as the average value of both studies. Among those included, the scientific studies used here are the only ones that have reliably measured the CO_2 content in the breathing zone of the face mask. The exceeding of the limit values is a cause of concern.

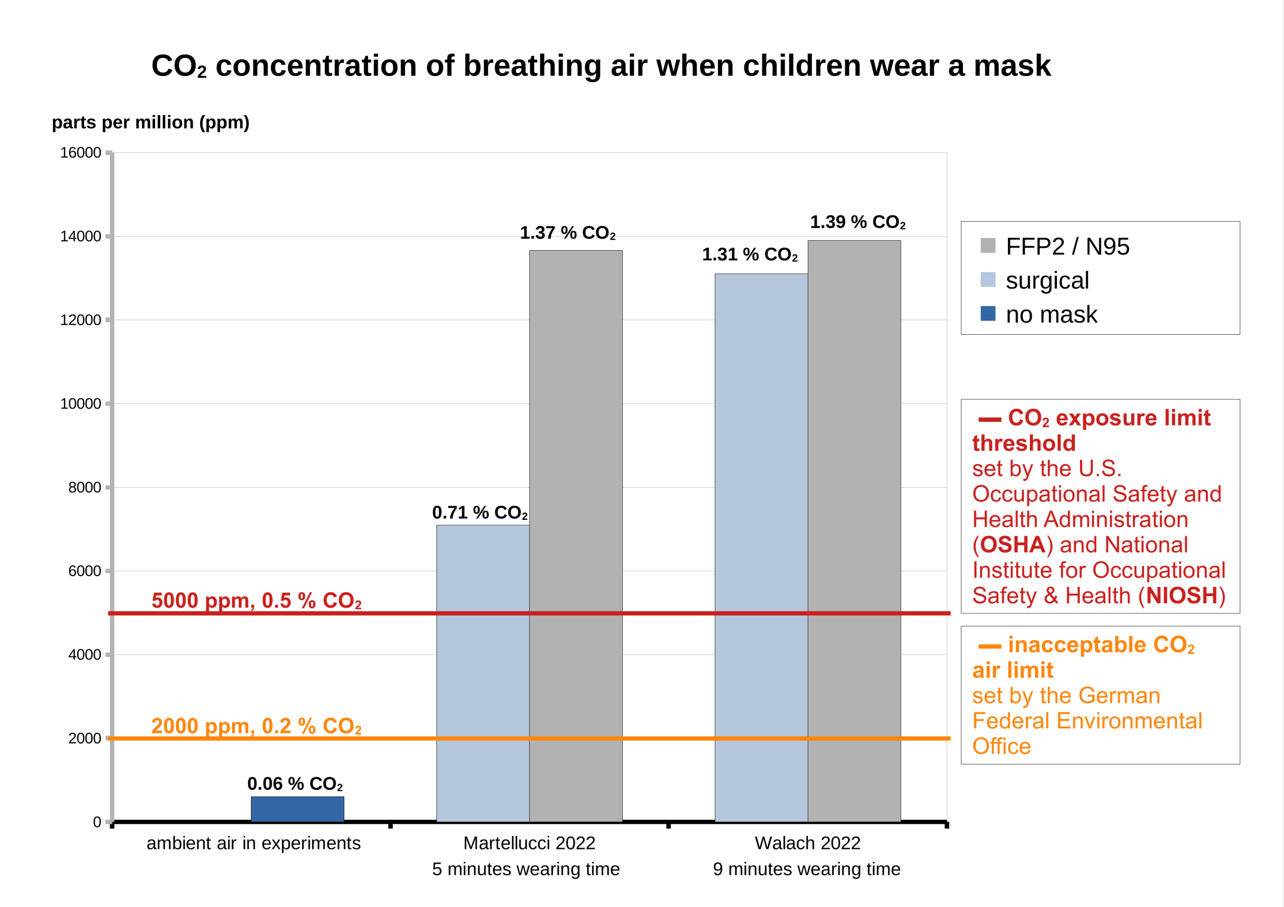

According to a review of 43 studies, in the inhaled air from masks carbon dioxide rises above the US-NIOSH threshold limit norms shortly after donning (30 minutes) [27]. Also, toxic CO_2-levels in breathing air may be exceeded while wearing masks, which can lead to neurotoxicity and reproductive toxicity referring to animal studies [27]. This statistically significant CO_2 rise, can be particularly toxic for children, adolescents and early life [27].

In addition to the physio-metabolic problems discussed, there are other chemical-toxicological risks associated with children wearing masks. It is possible for hazardous substances contained to migrate from the masks to the saliva. In an experimental study of the contaminants from printed and unprinted children`s masks to saliva, thirteen volatile compounds including toluene, chlorobenzene, irganox 1076 and 2-(2-butoxyethoxy)ethyl acetate were found to migrate to saliva [184].
Due to a review of 24 studies, masks are also a source of potentially harmful inhalation exposition to toxins with health threatening and carcinogenic properties at population level with almost zero distance to the airways and predominant oral breathing while wearing a mask [28]. Mask content and release show exceedances of USEPA,- WHO-, EU-Air Quality and Oeko-Tex® Standard 100 limits for micro- and nanoplastics (MP, NP), organic toxins and anorganic toxins. Phthalates showed a 43-fold exceedance of the carcinogenic risk value in children masks. Volatile Organic Compounds had a 25-fold exceedance of the carcinogenic risk value in children masks [28]. For further details see Figure 3. These findings are confirmed by additional studies [185].

Figure 3. Graphical summary of numerous studies showing that the mask content/release exceeds the prescribed limits for various toxins by a factor of up to a thousand times. Logarithmic scale of the Y-axis due to the large differences in limit value exceedances. Red X-axis as limit value threshold comparison of the exceedance of microplastic, anorganic and organic toxins broken down by toxin class and using the threshold limits of WHO Air Quality Guideline (AQG), EU target guideline, German Federal Environmental Agency, the Oeko-Tex Standard 100, and the U.S. Environmental Protection Agency (US EPA) levels. Data and limit thresholds taken from tables from a recent review [28].

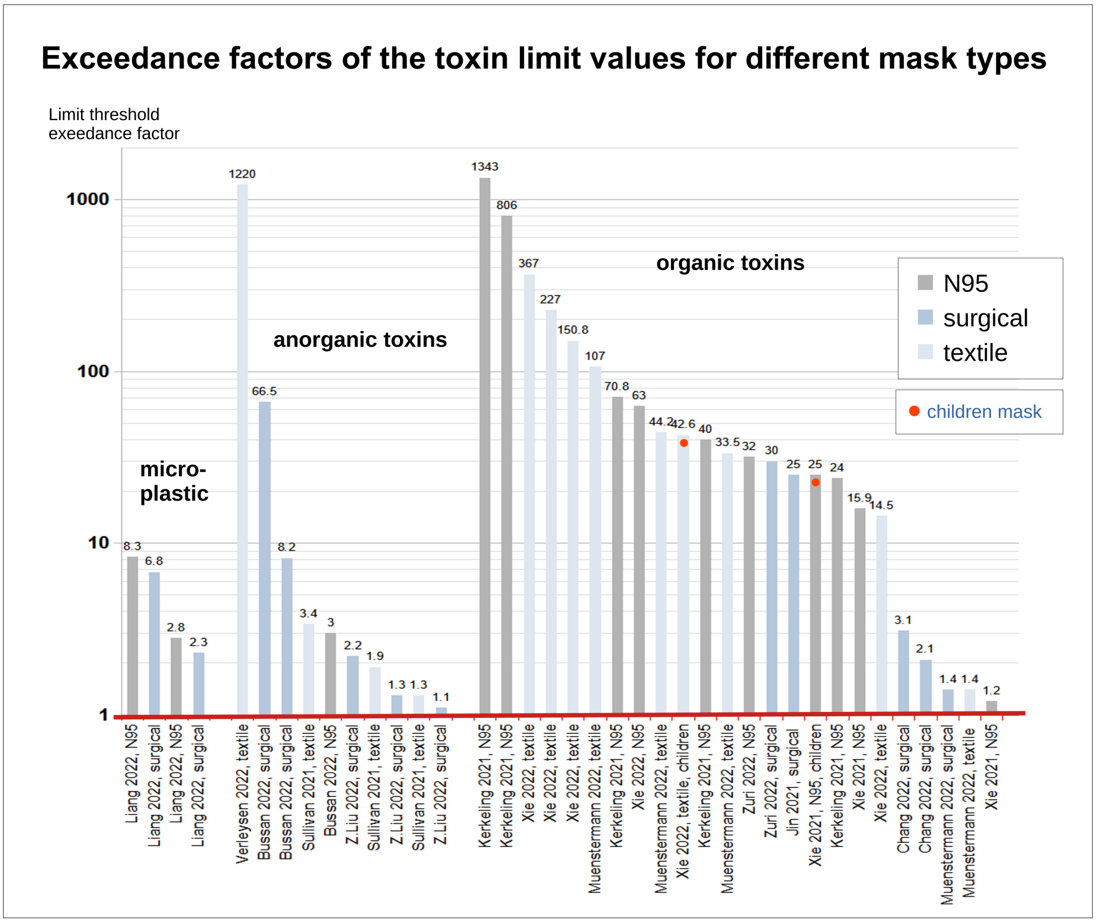

Altogether, it appears obvious, that the physiological primary mask studies in children having short experiment/evaluation times, which are most often restricted to few minutes wearing time with a median of 10 minutes, are not able to explore adverse effects in longer exposition periods. Some studies have small experimental groups and partially selected parts of the population and no appropriate control groups. Therefore, conclusions that masks generally are physiologically and clinically safe for children are not tenable, as these statements are not generalisable and also have no ecological validity.
On the other hand, the toxicological mask effects with regard to CO_2 breathing (Figure 2), organic and inorganic toxins, including carcinogenic compounds and elements (Figure 3), are undisputed [27,28].

4.6. Empirically/experimentally unproven claims

During the first and second year of the SARS-CoV-2 pandemic some scientifically and empirically non-proven statements were made regarding face masks use in children to mitigate the risk of transmission (Supplement B: Table S6), although there was no clear scientific and empirical evidence for such an antiviral (virus protective) effect [186,187]. Accordingly, the evidence certainty for those statements was found to be predominantly very low (Supplement A: Table E and F).

For example, in an opinion paper published in 2020, cloth masks, surgical and N95 masks were recommended for children older than 2 years of age [7]. The authors suggested using parents and schools to enforce the demand [7].
A statement by the Italian Paediatric Society in 2020 promoted the use of masks among the paediatric population, stating that they are effective and explaining the importance of their proper use [188]. As proof for this claim two papers were cited, one meta-analysis study commissioned by the WHO (evidence level Ia), with no clear, scientifically graspable benefit of moderate or strong evidence from wearing mask against virus transmission [189], and one breath sample collection with surgical mask under an artificial laboratory condition and very short mask wearing time, which is not representative for longer mask wearing and comparable to real-life conditions [190].
A narrative review in 2021 claimed that the use of masks is not associated with changes in respiratory function or gas exchange abnormalities [191]. According to the authors masks would also offer protection against other various respiratory viruses like Influenza or Rhinovirus [191]. However, no higher quality empirical evidence has been presented to support these claims.
A statement by the German Society for Paediatric Infectiology (DGPI) claimed in 2021 that masks are an important measure of restricting the spread of SARS-CoV-2 and despite being cumbersome and inconvenient, in children relevant adverse effects had not been reported and are not to be expected [192].
A statement by the Association of Schools of Public Health in the European Region (ASPHER) and the European Academy of Paediatrics (EAP) claimed in 2021 that masks

can offer the same level of protection against COVID-19 for children as they do for adults [193].
Sponsored by the United States National Institutes of Health and the Cystic Fibrosis Foundation an opinion paper claimed, that the usage of masks is critical for limiting the aerosol spread of SARS-CoV-2 [194]. As with all other statements, there is no experimental data with ecological validity to support this assertion, only hypotheses from aerosol science, and the theory of transmission via droplets and aerosols [194].

Overall, these premature assessments at the beginning of the pandemic appear to be less scientifically based and more politically motivated and in parts fuelled by conflicts of interest as often happened in the context of mass hysteria during the COVID-19 pandemic [195].

4.7. Bacterial, fungal and viral contamination

Regarding face mask contamination in children no citable literature was found in our search. However, grey literature exists with case series of laboratory proven face mask contamination in children [196]. Omitting the contamination topic would be neglecting an important aspect and would also contradict a holistic view. Therefore, we discuss this item with existing literature regarding adults.
Both our own experimental studies [197,198] and the published literature show that masks enrich/host numerous microorganisms, including pathogens [198–213].

The microbial contamination of mask surfaces is up to several hundred times higher than the limit value of the German standard for ventilation system surfaces VDI 6022 [198,214]. Contamination increases with longer exposure times [197,200–205,207,212,215] and is demonstrably higher for N95/FFP2 masks than for surgical masks [213].

The literature proves contamination and colonisation of the masks by bacterial and fungal opportunists of the genera *Acinetobacter, Aspergillus, Alternaria, Bacillus, Cadosporium, Candida, Escherichia, Enterobacter, Enterococcus, Klebsiella* (including *K. pneumoniae*), *Micrococcus, Microsporum, Mucor, Pseudomonas, Staphylococcus* and *Streptococcus*.
The number of bacteria increases linearly with the length of time the masks are worn [198,201], and prolonged wearing can also have a consequent effect on the microbiome, the natural bacterial colonization, of the skin and respiratory tract which in turn can lead to eye, skin, oral and respiratory diseases [197,198,201,202,212,216–219]. These microorganisms trapped in the meshwork of the mask can then be inhaled, causing infections of the respiratory tract [220,221] or, if they are distributed via air streams [222–229], even infections of the eye [227,230–234]. In addition, the microbiome of the skin is disrupted, which can lead to or promote other infections and allergic diseases [202,205,219,235,236]. Finally, the microorganisms accumulated in the mask can be

distributed through leakage [223,228,229], additionally enhanced by the nebulizer effect of the mask [27,29,225,237,238].
One of the causes for concern is that there is hardly any surface, any material, not even the bare skin, which ensures the viruses such a survival probability and long-term maintenance of infectivity as the polypropylene meshwork of the masks, in which SARS-CoV-2 viruses accumulate and remain infectious for up to 2 weeks, even when dried [239,240].

4.8. Risk benefit analysis and risk-assessment

As the risk of wearing a mask for children must be lower than not wearing one, a preliminary, also toxicological risk assessment must be ensured, using a worst-case consideration which is necessary in this kind of protective approach [36].

In 2020, the WHO and UNICEF have jointly advocated the 'do no harm' principle with regard to the use of masks for children, with prioritisation of the best interest, health and wellbeing of the child [10].

In the meantime, many new facts have emerged and from a physicians´ perspective, the use of masks must be appropriately assessed in accordance with the Hippocratic oath and the principle of '*primum nihil nocere*' (first do no harm). It must be avoided at all costs that the damage caused by the preventive measure becomes greater than that caused by the disease itself. To protect the health of their patients, doctors should also base their actions on the guiding principles of the Geneva Declaration of 1948 as revised in 2017. According to this, every doctor undertakes to put the health and dignity of his patient's health first and, even under threat (or under political pressure), not to use their medical knowledge to violate human and civil rights [241].

It has been stated, that children's human rights under the UN convention on the rights of the child and other treaties require decision makers to tread particularly carefully when deciding whether to mandate mask-wearing for children or not [16], as in fact, children have been enduring the most disproportionate disruption to their lives in their most formative years during the pandemic [15].
When comparing countries with rigid measures including face mask mandates like the UK to others with weaker measures, like Sweden, harms for children due to the COVID-19 policies become obvious. The authors of a corresponding review found a drop in educational attainment in countries with rigid measures including face mask mandates [242]. Indeed, among many other risks and adverse effects masks impede learning, especially for children [27,31,101,102,108,110,129,160,243,244].

4.8.1. Unproven effectiveness of masks in children against viruses

At the beginning of the pandemic there was sparse data regarding the effectiveness of measures implemented in the school setting to contain the COVID-19 pandemic especially regarding masks [245]. However, even after the pandemic the data appears not convincing [15,20,25,35,66,69,71,73–75], see also section 4.1. and Supplement B (Table S1) for further information. And a post-pandemic systematic review, which evaluates the body of literature on mask wearing exclusively in children to assess the existing evidence regarding protection offered by face masks against SARS-CoV-2 infection or transmission states, that the body of scientific data does not support masking children for protection against COVID-19 [24].
Additionally, one has to consider, that there is no proven SARS-CoV-2-transmission by symptomless individuals [33,246,247].
Therefore, the requirement to wear masks, which is based on the argument of asymptomatic spread, remains dubious at best for healthy children.
Respiratory disease experts have already discussed the insufficient evidence in favour of widespread use of masks against viruses in detail [33]. The mask theme for children was also evaluated by experts [35].
In a recent study in the general population, it was even found that wearing masks is associated with an increased risk of COVID-19 infection albeit with several possible confounding factors [248]. This corresponds with earlier findings of other authors, who found significantly higher SARS-CoV-2 infection and mortality rates in the mask-wearing cohorts [221,249].

Indeed, some studies, including modelling and in vitro laboratory simulation studies (artificial conditions) aim to demonstrate lower virus transmission when using masks [250–252]. However, they evince several methodological weaknesses [35] and lack ecological validity (generalisability of the test results to the real world, e.g. to situations or environments typical of everyday life). In real-world scenarios, there are many problems with application errors/material deficiencies that reduce the modelled or assumed effectiveness of protective masks [223,228,253]. Specifically, those problems of the studies aimed at proving the effectiveness of masks against viruses have been discussed in detail [20,27,33,35].
In addition, the correct use of the masks in order to fulfil the desired protection purposes in the general population and in children is not viable, in particular due to the many possible application errors [13,27,29,198,254,255]. Correspondingly, in children, the correct wearing rate of masks was only 31.9% without a supervisor in one study [71]. In another observational study, only 24.5% children used the mask appropriately [72]. This is mirrored by surveys of parents [99]. Parents often applied masks in children because it was mandatory (93.4%) even if they disagreed with it (63.3%) [100]. Interestingly, teachers report a higher percentage of proper use of the mask by their students than in other surveys, such as those mentioned above [256].

Even when masks are donned/used correctly in higher percentages, face or mask touching behaviour was observed in 10.7% and 13.7% of individuals, respectively [257].

A significant risk of mask use in the general population and also among elder children is the creation of a false sense of security with regard to protection against viral infections, especially in the sense of a falsely assumed, strong self-protection [13,26,29,186,258–260]. This results in distorted prioritisation in the general population and in elder children, counteracting the recommended measures in connection with the COVID-19 pandemic: The WHO prioritises social distancing and hand hygiene with medium evidence and recommends the wearing of face masks with weak evidence, particularly in situations where individuals are unable to maintain a physical distance of at least 1 metre [189].

Masks cannot be effective against COVID for these two reasons: the viruses on plastic-polypropylene meshwork of the masks can stay infectious for days up to two weeks [239,240] and children are unable to follow the guidelines for wearing them appropriately [8,13,27,71,72,254]. This was proven even for adults [255].

4.8.2. Lack of mask standardisation (virus filtration)

From a standardisation perspective, the filtration efficacy of masks for viruses remains hypothetical and does not comply with established standards. For medical masks, there have been national and international standards only for bacterial filtration efficiency (BFE), e.g. the EU-EN 14683 or the USA-ASTM F2101. They are the prerequisite for general authorisation. However, since 2020 (i.e. for almost five years) there has been no comparable standard/testing of masks for viruses.

Accordingly, in an important evaluation on human subjects with NaCl aerosol, which represented the size range of bacterial and viral particles, the overall filtration performance (protection factor) of surgical and FFP2/N95 masks for larger, bacteria-sized particles (0.5 to 5.0 µm diameter) was better than for smaller ones [261]. Interestingly, most of the FFP2/N95 respirators and surgical masks tested performed worst with particles between 0.04 and 0.2 µm in diameter, which corresponds to the size of coronaviruses and influenza viruses [261].

In view of the fact that medical masks (surgical masks and FFP2/N95 masks) increase the exhalation of particles in the smallest size range of 0.3 - 0.5 µm and therefore shift the geometric mean diameter to smaller sizes (longer in the air) than would be the case without a mask [27,29,198,237], this raises major doubts concerning filtration effects of masks.
These scientific facts point to the nebulisation effect of masks, which is an additional reason for their weakness against virus transmission in general.

Ultimately diverse microorganisms (viruses, bacteria, fungi) can be transmitted through leakage [223,228,229,262,263] and the nebulising effect of the masks [27,29,225,237,238].

Moreover, there is hardly any surface, any material, not even the bare skin that ensures viruses such a high survival rate and long-term preservation of infectivity as the plastic-polypropylene meshwork of the masks, in which SARS-CoV-2 viruses can accumulate and remain infectious for up to 2 weeks, even after drying of the masks [239,240].

4.8.3. Negligible infectivity and COVID-19 course in children

There is currently no evidence of COVID-19 transmission through child-to-child interaction or via schools: Children are less infectious than adults, in addition, children appear less susceptible to infection when exposed to SARS-CoV-2 [264].

If infected, most of the young individuals were either asymptomatic or showed mild symptoms, resulting in fewer hospitalizations [265].

One also has to consider, that the negligible, very low mortality rate for children is 0.0003% (3 in 1 million), when infected with SARS-CoV-2 [54]. But the psychological and physiological burden that comes with mask obligations impacts health in young individuals with more probable wide-ranging and numerous negative effects with much higher percentages than the risks of not wearing a mask.

4.8.4. Scientifically proven adverse mask effects and MIES

Clinical symptoms

In a survey in 25,930 children it was found that 68% of children complain about impairments caused by wearing masks. Reported side effects include irritability (60%), headaches (53%), difficulty concentrating (50%), reduced happiness (49%), reluctance to attend school or kindergarten (44%), discomfort (42%), learning difficulties (38%), drowsiness and fatigue (37%) [160].
Other authors found breathing discomfort in 53.1%, headache in 49%, speaking difficulties in 45%, change in mood in 45.2%, cutaneous disorders in 42.4% and fog on glasses in 68.2% [100].
In one survey in 706 children, 44.9% showed negative consequences from wearing facemasks with respiratory discomfort/breathing difficulty in 33.9% besides cutaneous adverse effects in up to 16.3%, including itching symptoms, rash, pressure effects, and acne [159]. However, studies with focus on perceptions of the usefulness of masks in schools report lower side effects of mask use of approximately 20% [97].

Altogether, clinical symptoms, while wearing a face mask are undisputable. For more details see section 4.4. and Figure 1.

Restriction of normal breathing

According to the literature, masks restrict normal breathing and interfere with O_2-uptake and CO_2-release.
On one hand this is due to an increase in breathing resistance when wearing a mask [147,148,261,266–273] which can be up to twice the normal value in adults [267], well more pronounced in children.
On the other hand, there is the problem with the dead space while wearing a mask. Masks extend the natural dead space (nose, throat, trachea, bronchial tubes) to the outside, beyond the mouth and nose, up to a doubling in size [30,42,140,180,274–282] .
Due to an increased dead space volume, masks can cause increased rebreathing of exhaled gas with an accumulation of carbon dioxide in the wearer [140,271,283–289]. Additionally, the resulting increased pendulum breathing volume with a mask goes hand in hand with a relative reduction in the volume of gas exchange available to the lungs per breathtake up to 37 % in adults [267], well more pronounced in children.
The mask thus clearly acts as a disruptive factor in breathing [29,266,267,269,290].
Undoubtedly, masks compromise respiratory compensation in the wearer with reduced ventilation and tidal volume [266,269,290]. Though evaluated wearing durations were shorter than daily/prolonged use in studies [266,269,290], outcomes independently validate down-stream physio-metabolic dysfunctions. Meta-analytical analyses of primary studies show that masks reduce the respiratory minute volume in adults by an average of 19%, with N95 masks (FFP2) even by 24 % [266,269,290], which will surely be more pronounced in children.
Indeed, a lot of studies report breathing difficulties in children due to mask wearing [43,81,97–100,156,159,167,169,181].

Carbon dioxide rise

Numerous scientific studies describe in detail the causality of mask wearing and CO_2 enrichment/re-breathing [30,31,34,148,150,151,177,179–181,266,269,271,273–276,279,283–286,290–309]. A significant increase in carbon dioxide in the mask breathing zone has been scientifically proven in many studies [27,30,31,140,274,285,291,292]. A recent paper on the topic depicts a massive increase of up to 80 times the normal value of carbon dioxide in the breathing air while wearing a mask [27].
Fresh air has a CO_2 content of approximately 0.04% (400 ppm), but in reliable human experiments masks show a possible chronic exposure to a content of 1.41-3.2% CO_2 in the inhaled air, and even 2.8-3.7% carbon dioxide within the FFP2/N95 mask [140,271,283,285,286,288]. Thus, wearing masks has the potential to exceed the acute and chronic safety limits for CO_2 concentration in the breathing air [27]. In the inhaled air from masks carbon dioxide can rise above the NIOSH 15 minute limit (3% CO_2) and above the NIOSH 8 hour limit (0.5% CO_2) already shortly after donning (30 minutes) [27].
These exceedances of CO_2-levels in breathing air could also be proven by reliable measurements in children while wearing masks [30,31].

Martellucci et al. could show, that the occupational exposure limit of 5000 ppm threshold (0,5% CO_2) was largely exceeded in children wearing surgical masks or FFP2/N95 respirators for 5 minutes [30].
According to the experiments of Walach et al. the carbon dioxide rise in inhaled air under masks (surgical 1.31% CO_2 and FFP2 1.39% CO_2) in children was far beyond the level of 2,000 ppm (0.2% CO_2), considering the limit of acceptability and drastically beyond the 1,000 ppm (0.1% CO_2) that are normal for air in closed rooms [31].
Alarmingly, referring to these measurements, not only the NIOSH limits, but even the toxic CO_2 breathing-air limits for early life gained from animal studies [27] are exceeded. Experimental animal data show demonstrably harmful long-term effects of elevated CO_2 in breathing air at threshold levels above 0.3% (3,000 ppm), 0.5% (5,000 ppm) and 0.8% (8,000 ppm) [27]. Already 0.3% CO_2 in breathing air can be responsible for destruction of nerve cells, impairment of memory and learning ability, increased anxiety in early life [310,311]. Carbon dioxide levels of 0.5% in breathing air are capable of a destruction of cells in the testicles in adolescents [312]. Breathing of 0.8% CO_2 by pregnants may cause stillbirths and birth defects [313–315].
A statistically significant CO_2 rise in children while wearing a mask could also be shown by other authors in their studies despite only short wearing times of predominantly 10 to 15 minutes [173,177,179–182], which is far lower than the average school time of 6 hours. However, due to the lack of a toxicological approach those studies failed to draw appropriate conclusions regarding the possible harmfulness of mask wearing for children [173,177,179–182]. Due to the absence of hypercarbidaemia, an all-clear signal was falsely given, but without considering the breathing air concentration. From a toxicological and medical point of view it makes little sense to consider only standard values in the blood (e.g. hypercarbia), which only occur when compensation mechanisms fail, because the toxicity of CO_2 does not depend exclusively on the blood concentration but primarily and rather on the concentration in the air which is breathed [27]. Otherwise, standards such as the NIOSH would be pointless.
The studies of Walach et al. [31] and Martellucci et al.[30] should be regarded as a clear warning as far as this is concerned (Figure 2).
All in all, despite compensatory metabolic and equalization mechanisms, the rebreathing of CO_2 from the dead space of the masks is also noticeable in the blood of the wearer, even if only subliminally [27].
CO_2 inhalation in low concentrations has several clinically significant short-term effects on human health, despite the fact that blood levels do not exceed the limit values: Physiological changes already occur at concentration levels of 0.05 % to 0.5 % for CO_2 in the air, which are expressed in an increased heart rate, increased blood pressure and an overall increased circulation with the symptoms of headaches, fatigue, difficulty concentrating, dizziness, rhinitis and dry cough [141].
While the effects of short-term exposure on cognitive performance begin at 0.1% CO_2 levels in the air we breathe and result in reduced mental performance, impaired decision-making and reduced speed of cognitive reasoning, many other long-term effects are known at carbon dioxide concentrations above 0.5% breath content:

If the limit value of 1% CO_2 in the air breathed is exceeded, the harmful effects include respiratory acidosis/metabolic stress, increased blood flow and overall reduced exercise tolerance [141]. These clinical consequences occur without blood CO_2 threshold exceedances, but they are related to the CO_2 content in the air we breathe.
There is also a confirmed immune system-inhibiting effect of increased CO_2 respiration, mediated by a change in gene expression/nuclear metabolism [316].

Blood carbon dioxide concentration exerts an important influence on intra- and extracellular pH. CO_2 (which is a fat-soluble small molecular gas with a strong diffusion capacity and can cross the blood-brain barrier) quickly passes through the cell membranes to form carbonic acid with H_2O inside of the cell, releasing H^+ ions and, when in excess, causes acidosis [317–319]. One of the consequences of acidosis is a decrease in transmembrane Ca^{2+} conductivity and as a result a decline in the excitability of cells [320,321]. In neurons, calcium overload causes excitotoxicity and apoptosis during hypoxia [322].

Furthermore, carbon dioxide is also known to play a role in oxidative stress caused by reactive oxygen species (ROS) [323] and an oxidative damage to cellular DNA can trigger mutations in many cells [323,324].
Moreover, an inflammatory process, which can lead to serious illness is known to be caused by low-level CO_2 exposure in humans and animals [324–328]. This is because increases of CO_2 result in higher levels of pro-inflammatory Interleukin-1β (IL-1β), a protein involved in regulating immune responses causing inflammation and vascular damage [326]. Significant upregulation of IL-1β may be associated with an imbalanced immune system and a procoagulant state in the body.

To counteract respiratory acidosis while wearing face masks the kidneys retain bicarbonate to normalize the pH of the blood. With ongoing increase of the CO_2 burden, the body starts using its' bones to regulate the acid levels in the blood. Bicarbonate and a positive ion (Ca^{2+}, K^+, Na^+) are exchanged for H^+ [329].
The Carbonic Anhydrase (CA) enzyme [330] participates in metabolic reactions that convert CO_2 and result in the precipitation of calcium carbonate [331–333]. CA is involved in the calcification of human tissues including bone and soft-tissue calcification [331]. Carbon dioxide conversion by the CA enzyme provides bicarbonate and hydrogen ions that fuel the uptake of ionised calcium, which is then deposited in the body tissues as calcium carbonate. Increased CO_2 in the blood caused by breathing elevated levels of the gas could lower the pH enough to increase the activity of CA, thereby potentially increasing calcium carbonate deposits [332], also while wearing a mask for longer periods of time [27]. Significant tissue calcification has been observed in animals after a 2-week exposure to 1% CO_2 or an 8-week exposure to 0.5% CO_2 with only slight reductions in pH [334]. This could also occur, if a face mask is worn for a prolonged period, through CA activity at sites where tissues are in contact with the plasma, e.g. arteries and kidneys.

There is a particular need for research on increased CO_2 breathing when wearing a mask, as this phenomenon appears to be clinically relevant with pathological CO_2 concentrations in the air breathed, even when these concentrations are not found to be elevated in standard blood test results for carbon dioxide.

Oxygen drop

Some scientists found a significant oxygen drop in children wearing masks [170,178,183], despite other authors finding no changes in a few children studies with predominantly very short mask wearing times (1-12 min) [30,31,95,169,171–173,175,182] and no significant SpO_2 changes in slightly longer mask wearing times (42 minutes) in relatively small experimental children groups (22-47) [176,177,179]. This is hinting at an oxygen drop belonging to the cornerstones of the so-called mask induced exhaustion syndrome (MIES, see referring section below) also in children.
And different types of masks are capable of causing this. Lubrano et al. found a significant decrease in oxygenation after 30 minutes and during 12 minutes walking test with N95 masks in children [178].
Castro et al. found increased pulse rate and reduced arterial blood oxygen saturation SpO_2 in children wearing cotton face masks [183].
Mallet et al. could evidence in children wearing surgical masks for 6 minutes the lowest SpO_2 recorded at 93%, while the highest carbon dioxide tension measured was 5.67 kPa [170].
A lot of scientific papers describe the causality of mask wearing and measured O_2 drop in adults in detail [34,146,148,149,151,153,269,271,274,279,290,293,295,296,298,299,301,307,335–356].
On the one hand, this is due to the lower oxygen content in the mask dead space [140,274,285], but also due to the reduced respiratory minute volume under mask wearing [266,269,290] with a relative reduction in the gas exchange volume available to the lungs per breathtake [267]. The fact that adult mask wearers have significantly lower oxygen saturation values (SpO_2 %) at rest as well as during exercise can also be explained by the increased dead space volume discussed above and by an increased breathing resistance [29]. In some experiments, the measured oxygen saturation values in the adult mask wearer group fell significantly below the normal limit [34,285,342,344,349], which clearly demonstrates a clinical relevance of this phenomenon.
However, subthreshold decreases in oxygen saturation when wearing a mask also show clinically relevant effects. For example, the resulting drop in oxygen (O_2) saturation of the blood on the one hand, and the increase in carbon dioxide (CO_2) on the other, contribute to an increased vegetative noradrenergic stress response [357,358].
Depending on the duration of wearing a mask, a progressive decrease in blood oxygen (SpO_2) in adults is observed [34,148,285,296,298,300,338,341,345,348,350,359,360]. This decrease in the SpO_2 level confirms the reported progression of oxidative stress (measured by significantly increased aldehydes in exhaled air, which originate from lipid peroxidation) by Sukul et al. [34]. In addition, the isoprene concentration in the breathing

air decreased significantly during mask wearing [34]. However, the origin of isoprene in human breath is lipolytic cholesterol metabolism in muscles [174]. Therefore, the decrease in isoprene in breath while wearing the mask is due to sympathetic vasoconstriction in the muscle compartments caused by deoxygenation and hypercarbidaemia [34].
Some studies have shown that oxidative stress (under hypoxic conditions) can inhibit the cell-mediated immune response (e.g. T-lymphocytes, TCR-CD4 complex, etc.) in the fight against viral infections, which can gradually lead to a general suppression of the immune system [361,362]. The mechanism responsible for this is the following: arterial hypoxaemia increases the level of hypoxia-inducible factor-1α (HIF-1α), which further inhibits T cells and stimulates regulatory T cells [362]. This HIF-1α modulating effect has already been shown for masks [304].

Mask-related unfavourable reductions in immune blood cells, including defence cells, have also been observed and described [363]. This may favour the conditions for infections, including SARS-CoV-2, and also exacerbate the consequences of this infection. In essence, due to the molecular mechanisms they trigger, in the long term, masks can even expose the wearer to an increased risk of infection and its' severity – in contrast to the initially intended effect [361,362,364].

The transient hypoxia topic therefore requires further research in children. Also with regard to the increased vegetative noradrenergic stress response when wearing a mask for a longer period of time.

Microbial contamination

Particularly for children, who often do not use/wear mask appropriately [13,27,29,71,72,99,100,198,254], there may be a higher microbial contamination risk. Both bacterial, fungal and viral contamination of the masks can contribute to a distribution of microorganisms and pathogens and reduce the desired effect, and can even lead to health risks for the wearer [198], see section 4.7.

Toxicity and carcinogenicity

In addition to the clinical symptoms, the carcinogenic and toxicological risks of wearing a mask should be discussed.
Of course, masks filter bacteria, dirt and plastic particles and fibres from the air we breathe, but according to comprehensive data, they also harbour the risk of inhaling microplastic and nanoplastic particles and potentially toxic substances from the mask material itself [28]. Undoubtedly, the results of a recent review show that worldwide during the SARS-CoV-2 pandemic, mask mandates have been an additional source of potentially harmful exposure of the population to toxins at a distance of almost zero to the respiratory tract (predominantly oral inhalation route), and to the gastrointestinal tract [28]. Of the 24 studies included 63 % showed disturbing high values and possible

exceedances for substances such as micro- and nanoplastics (MP and NP), volatile organic compounds (VOC), xylene, acrolein, per- and polyfluoroalkyl substances (PFAS), phthalates including DEHP and heavy metals such as Pb, Cd, Co, Cu, Sb and TiO_2. For the N95/FFP2 mask, the MP release was 831 μg in 24 hours and up to 4400 particles within 4 hours (with a predominant size <1 μm) and up to 6×10^9 NP in 4 hours [28].
Surgical masks released up to 3152 microfibres in <1 hour. The worst-case estimates show that the breathing air can exceed the limits of the WHO Air Quality Guideline (AQG) [28]. There were also exceedances of the total volatile organic compounds (TVOC) with 403mg/m^3 within only 17 minutes for the FFP2/N95 mask, and >1000μg within the first hour for the surgical mask, which is above the limit values of the EU target guideline, the German Federal Environment Agency and the Oeko-Tex Standard 100. The textile standards were also exceeded for the toxic PFAS (FFP2/N95, surgical mask, textile mask), DEHP, phthalates, flurotelomer alcohol, FTOH (each textile mask), naphthalene (FFP2/N95), Pb (surgical mask, textile mask), Cu (surgical mask), Sb (FFP2/N95, textile mask), Cd and Co (each surgical mask). In addition, acrolein (surgical) and xylene (FFP2/N95) exceeded the limits set by the USA and the German Federal Environment Agency.
Some of the immediate complaints when wearing a mask (headache, (dry) cough, rhinitis, runny nose and skin irritation) could be related to the potential short- and long-term negative effects of the above-mentioned toxins released [28].
Altogether, mask content and release show exceedances of USEPA, WHO, EU Air Quality and Oeko-Tex® Standard 100 limits for Micro- and Nanoplastics (MP, NP), organic toxins and anorganic toxins [28].

Undoubtedly, there is a toxicological and carcinogenic risk of mask use for children, even in special masks dedicated for children use: For phthalates there was a 43-fold exceedance of the carcinogenic risk in children masks and for volatile organic compounds (VOC) there was a 25-fold exceedance of the carcinogenic risk in children masks [28], for further details see Figure 3.

Altogether the possible toxicological mask effects including inhalation of plastic particles (high micro- and nanoplastic release and content) and exceedances for toxic substances (volatile organic compounds, acrolein, phthalates. xylene, per/polyfluoroalkyl substances and for Pb, Cd, Co, Cu, Sb and TiO2) with carcinogenic potential and the exceedance of carbon dioxide in the inhaled air already make the face masks obsolete for children.
For more details see section 4.5. and Figure 3.
It is necessary to protect children in particular from harm caused by uncertified masks and improper use.

Psychological and sociological symptoms

The simplest and most obvious damage caused by masks, apart from the physiological, clinical, toxicological and microbiological ones discussed here, is the psychological and

social burden with impaired visual and verbal communication [102,105,108–110,115,117,132–134,365–368] impaired facial expressions and misinterpretation of emotions [108,111,112,115–118,128,133,369], also resulting in impaired early childhood learning [15,16,27,101,102,108,110,129,160].
Altogether, psychological and sociological symptoms, while wearing a face mask are undisputable. For more details see section 4.3.

MIES -mask induced exhaustion syndrome

All the mentioned psychological and physical deteriorations as well as symptoms that frequently occur simultaneously following longer mask use belong to a special face mask driven syndrome. In 2021 it was already shown that face masks can be responsible for the so-called Mask-Induced Exhaustion-Syndrome (MIES) when worn for longer periods like it was the case during the pandemic [27,29,34]. In 2021 the authors could objectify evident changes in respiratory physiology of mask wearers with significant correlation of O_2 drop and fatigue ($p < 0.05$). The authors could also show a clustered co-occurrence of respiratory impairment, O_2 drop and CO_2 rise, as well as headache, respiratory impairment, temperature rise and moisture under the masks [29].
The physical and chemical parameters in wearers do not usually exceed the normal values, but when wearing a mask often they tend to be statistically significantly measurable in the direction of pathological ranges. They are often accompanied by physical impairments. Depending on the activity profile, wearing time and constitution of the wearer pathological values can also be reached, exceeding normal values [29]. But even if this does not happen, risks remain. Consistent with the conclusions of Sukul et al, Fikenzer et al and Zhang et al. [34,266,290], there is evidence of adverse effects of face masks even without exceeding physiological thresholds/normal values in the mask wearers.
Even with CO_2 and SpO_2 values for example, which do not exceed the limit values, many clinical researchers have also found worrying results in mask wearers.
For example, a neuro-radiological study in adults showed a pathological and altered brain metabolism while wearing a N95 mask for 6 hours [154]. The MRI imaging showed a significant decrease in brain oxygenation with a more than 50% decline in oxygen supply in the cingular gyrus, an area responsible for the cognitive circuit.
An ophthalmological study indicates a risk of retinal damage with long-term use of masks. Face masks reduced the vascular density in the vascular plexus of the retina even under resting conditions after only 60 min [370]. Although the decrease in SpO_2 and the increase in blood pressure were significant, but still within normal physiological ranges (normal values), i.e. sub-threshold, these clear clinical effects occurred.
Another study reported a significant mask-induced increase in intraocular pressure (IOP) after only approx. 5 min of N95 mask wearing time [371].
Wearing masks can therefore counteract a therapy reducing intraocular pressure and thus contribute to irreversible long-term visual problems in children with e.g. glaucoma. In accordance with scientific analyses and reviews [27,29], even subliminal, but

chronic changes due to masks can lead to illness and clinically relevant secondary conditions in the long term.

Overall, even sub-threshold changes caused by the prolonged use of masks can therefore become clinically relevant. This so called low-dose long-term effect has been elaborated in some papers on adverse mask effects also in children [27,29].

Based on available literature, the so called MIES can comprise the following frequent, predominantly statistically significant physiological and psychological changes while wearing a mask for longer times also in children:

- Increase in dead space volume [30,42,140,180,274–281].

- Increase in breathing resistance [147,148,261,266–272].

- Increase in carbon dioxide [30,34,148,150,151,173,177,179–182,266,269,271,273–276,279,283,284,286,290,293–309,345].

- Drop in blood oxygen saturation [34,146,148,149,153,170,178,183,266,269,274,285,290,293,295,296,298,299,301,302,304,307,335–349,351–356,360,372].

- Increase in heart rate [34,148,151,153,183,268,271,273,279,296,300,301,338,353,372,373].

- Decrease in cardiopulmonary capacity [175,266,269,290].

- Feeling of exhaustion and fatigue [146,149–151,160,169,170,172,181,266,268,269,272,273,279,290,293,296,297,299,301,307,344,345,349,359,373,374].

- Increase in respiratory rate [34,42,148,167,177,273,296,299,300,344,345].

- Changes in respiration [148,172,182,266,269,273,290,296,299,300,344].

- Difficulty breathing and shortness of breath [43,81,97–100,148,149,151,156,159,169,170,181,266,268,273,280,296,299,303,340,341,343–345,351,359,360,375–390].

- Headache [97,100,151,152,154,160,275,291,294,299,304,335,341,343,348,350–352,356,359,379,383,388,391–400].

- Dizziness [148,154,170,275,291,299,304,335,343,344,346,356,383,396,399–404].

- Feeling of moisture and heat [42,81,149,155,156,162,260,266,268,272,273,296,345,384,390,405].

- Drowsiness (reduced ability to think and concentrate) [146–153,160].

- Decrease in empathy perception [96,102,109–111,115–118,133,158,406].

- Impaired skin barrier function with acne, itching and skin lesions [97,148,148,152,161,162,164,273,335,378,380,384,389,399,407–421].

-False sense of security [13,26,78,186,258–260].

-Increase in blood pressure [34,148,168,175,266,268,269,290,296,303,353,422].

-Increase in the temperature of the skin under the mask [140,162,262,272,273,345,390,423].

-Increase in the humidity of the air under the mask [140,162,272,345,390,423].

-Communication disorder [96,106,110,116–118,128,129,131,133,134,151,152,368,384,424,425].

-Voice disorder [100,132,133,384,426].

-Perceived discomfort [97,98,100,155,159,160,164,266,296,380,390].

-Increased anxiety [27,110,129,160,167,388,424,426–428].

-Elevated mood swings or depressive mood [100,110,160,388,424,426,427].

-Toxicological risks, including harmful and carcinogenic substances that are inhaled at almost zero distance from the respiratory tract [28].

-Biological hazards due to bacterial, fungal and viral contamination and possible spread [198]

-Risk to young life, including unborn and children, with potential nerve damage, testicular damage and stillbirths [27].

Altogether, MIES can have long-term clinical consequences, especially for vulnerable groups including children (Figure 4).

Figure 4. Potential unfavourable mask effects (adverse effects) as components of the mask-induced exhaustion-syndrome (MIES) during use. The chemical, physical, biological and toxicological effects, as well as the organ system consequences mentioned, are all documented with predominantly statistically significant results in the scientific literature found and compiled, also in children (see section 4.8.4). IOP = intra-ocular pressure, ENT= ear, nose and throat.

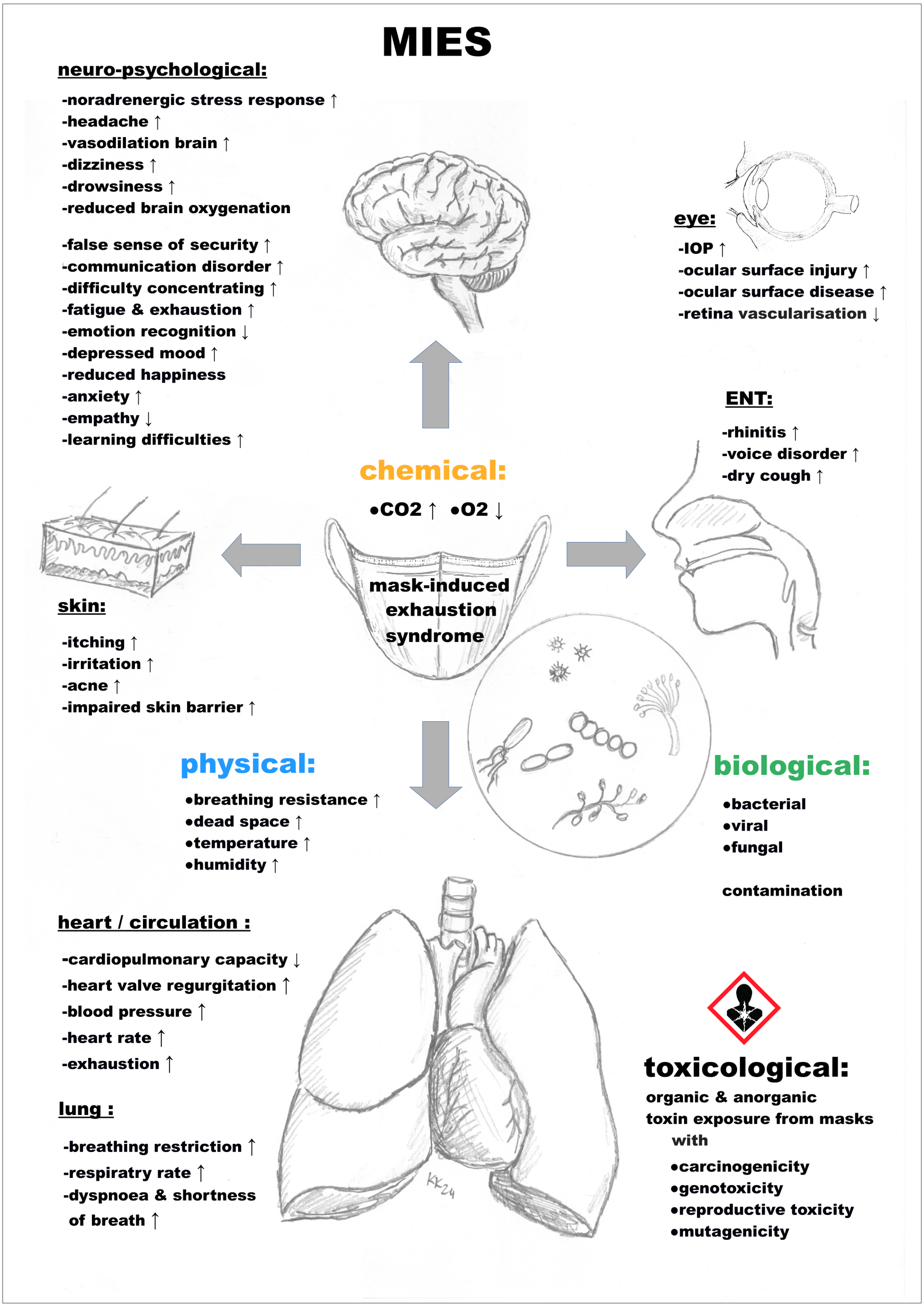

Extended mask-wearing by the general population and by children can lead to relevant effects and consequences in many medical fields. In any case, the MIES potentially triggered by masks contrasts with the WHO definition of health. As health is a state of complete physical, mental and social well-being and not merely the absence of disease or infirmity [166].

4.8.5. Risk assessment of masks and children

There was always the worst-case risk assumption for spreading and severe outcome associated with SARS-CoV-2. This is the way authorities are mandated to perform a risk assessment [36]. But the lack of efficacy of mask mandates (section 4.8.1.), the lack of mask standardisation for virus filtration (section 4.8.2.), the negligible infectivity and COVID-19 course in children (section 4.8.3.) and even the increased risk of infection and spreading mainly due to improper handling by children needs to be assessed in relation to the risks of unwanted and even possible serious adverse effects to health by these devices (section 4.8.4.).

Looking at chlorine dioxide (a drinking water disinfectant used worldwide), as possible biocide against SARS-CoV-2, whose function has been demonstrated in vitro [429], there is a risk assessment for humans available based on toxicity in rodents. This is according to international rules and regulations. The No Observed Adverse Effect Level – NOAEL in a rodent tox study was identified with 3 mg/kg bodyweight per day. At these concentrations there was neurotoxicity with neurodevelopmental effects [430]. Therefore, permitted daily exposure for humans with chlorine dioxide as biocide for the whole population (Reference Dose for Chronic Oral Exposure – RfD) was set to 0.03 mg/kg per day by applying an interspecies uncertainty factor of 10 and an interhuman uncertainty factor of 10 [430]. This uncertainty factor, which totals 100 is a factor used by risk assessors to derive a reference dose that is considered safe or below which an adverse effect is unlikely to occur. This approach based on animal data is always the basis risk assessment, which is mandatory for decision making by authorities implementing measures to prevent spreading of infectious diseases. For chlorine dioxide on the one hand the biocide activity and need for sufficient concentrations and on the other the safe amount of exposure to humans have to be balanced against each other.

The same would have been necessary for implementing mask mandates especially for children. But authorities refused to perform an appropriate risk assessment.

What is the risk getting severe COVID-19 infections for children? What is worst case risk of using this device with lack in proper handling? What is the worst case risk for the children using this device?

It is undisputed, that masks pose numerous psychological, physio-metabolic, toxicological and clinical risks, especially for children (Section 4.8.4.). It is also undisputed that children are not able to adequately follow the guidelines for the correct wearing of masks (section 4.8.1). The negligible infectivity and harmless course of COVID-19 with a mortality rate of 0.0003 % (3 in 1 million) in children is also a fact (section 4.8.3). But even when only looking at one single parameter, namely the proven carbon dioxide exposure of 0.7% to 1.39% in breathing air, while children are wearing a mask (Figure 2), the risk assessment is unfavourable.

Elevated carbon dioxide in the breathing air while wearing a mask implies on its' own clearly a high risk of irreversible damage to children health. Based on animal data carbon dioxide exposure is associated with complete loss of spatial orientation and irreversible loss of brainstem neurons at an exposure of only 0.3% CO_2 to juvenile rodents [27]. This asks for a mandatory definition of a limit exposure by applying interspecies and interhuman uncertainty factors. Such factors are an attempt to account for uncertainties and interspecies variability from animal to human. But when applying the same factors like it was done for chlorine dioxide this would result in a possible safe exposure of far less than 0.3 % carbon dioxide. Maybe these factors could be reduced because rodents in these safety studies had been exposed 24/7 till study end points [27]. Nevertheless, based on rules and regulations mandatory for such a chemical risk assessment we need to ask at least for a safety factor of between 3 to 10 [431]. Even if factor 3 is applied, it cannot be ruled out that low air quality due to increased carbon dioxide breathing while wearing a mask of 0.7 % to 1.39 % [30,31] can lead to irreversible damage to the brains of children and adolescents [27]. The values taken from the studies are only median/mean values [30,31]. Of course, the values may be lower, but an appropriate risk assessment is always based on the worst case, not the best case, using a worst-case scenario [36]. And our approach is quite conservative, as we don't even use the maximum values from the children studies. The maximum values were even as high as 1.52% [30] and 2.5% CO_2 [31] in the inhaled air.
Also the significant mask-related reproductive risk with possible testicular damage in adolescents is not considered in this analysis, as neurotoxicity involves more extensive damage with relatively lower carbon dioxide exposure [27].

Germany had the worst outcome of international children education testing in OECD Pisa 2022. We simply cannot exclude an irreversible substantial damage to childrens´ brains by these unproven mask mandates. And not only in Germany there was an unprecedented average drop in maths and reading but also all over the world [432].

Carbon dioxide breathing when children wear a mask is on average 0.7 % to 1.39 % with maximum values of 1.52% and 2.5% [30,31] and thus significantly above the safety reference dose that can be calculated on the basis of animal experiments [27,431].

If we consider the scientifically proven adverse mask effects and MIES on the one hand (section 4.8.4., Table 1) and the unproven effectiveness of masks in children against viruses (section 4.8.1.), the lack of mask standardisation regarding virus filtration (section 4.8.2.), the negligible infectivity and COVID-19 course in children (section 4.8.3., Table 2) on the other, the risk-benefit assessment becomes even clearer.

Table 1. Summary of key facts (scientific evidence) on the potential adverse effects associated with the use of masks by children for risk assessment.

**masks and children risk assessment – adverse effects**	**scientific evidence**		
toxicological risk (carcinogenic, mutagenic, physio-metabolic)	**yes**		
bacterial, fungal and viral burden (contamination)		**possible**	
physical symptoms	**yes**		
exacerbation of existing diseases	**yes**		
triggering of new diseases	**yes**		
psychological symptoms	**yes**		
sociological/developmental disruptive effect		**possible**	
large absence of undesirable effects			**no**

Table 2. Summary of the most important facts on environmental influences/contextual factors in relation to masks and children for a risk assessment. Consideration also in particular with regard to masking children for COVID-19.

**masks and children risk assessment – contextual factors**	**evidence/basics**
mask standardisation (bacterial filtration)	**yes**
mask standardisation (particle filtration)	**yes**
mask standardisation (virus filtration)	**no**
effectiveness of masks against viruses in children	**unproven**
ensuring appropriate use of masks in children	**unproven**
high SARS-CoV-2 infectivity in children	**no**
frequent devastating COVID-19 course in children	**no**

There are clear, non-negligible risks accompanying mask use in children (sections 4.3., 4.4., 4.5., 4,7. and 4.8.) with at least moderate evidence certainty in contrast to unclear effectiveness against viruses, which is predominantly based on assumptions and consists of empirically unproven claims with very low to low evidence certainty (section 4.6., Table E and F, Supplement A).

A risk assessment is always based on the worst case, not the best case.

Using a worst-case consideration [36] and taking into account the WHO and UNICEF, who have jointly advocated the 'do no harm' principle with regard to the use of masks for children, with prioritisation of the best interest, health and wellbeing of the child [10] in accordance with the Hippocratic oath and the principle of 'primum nihil nocere' (first do no harm) with the aim to avoid a damage caused by the preventive measure, being greater than that caused by the disease itself, as well as the guiding principle of the Geneva Declaration of 1948 as revised in 2017 [241] and the children's human rights under the UN Convention on the Rights of the Child [16], it becomes obvious to decide against face masks in children (Figure 5). Unfortunately, this was not common practice during the pandemic [15].

In recent expert book chapters they came to the same conclusion and therefore argued against masks in children and adolescents [433].

Figure 5. Graphical representation summarising the risk assessment in relation to masks and children taking into account the key ethical principles. Justitia, the goddess of justice in ancient Roman mythology is the symbol of applied justice and the upholding of all ethical principles. There are clear, non-negligible risks of mask use in children in contrast to unclear effectiveness against viruses which is predominantly based on assumptions and empirically unproven claims without convincing supreme empirical evidence, e.g. randomised controlled trials, etc..

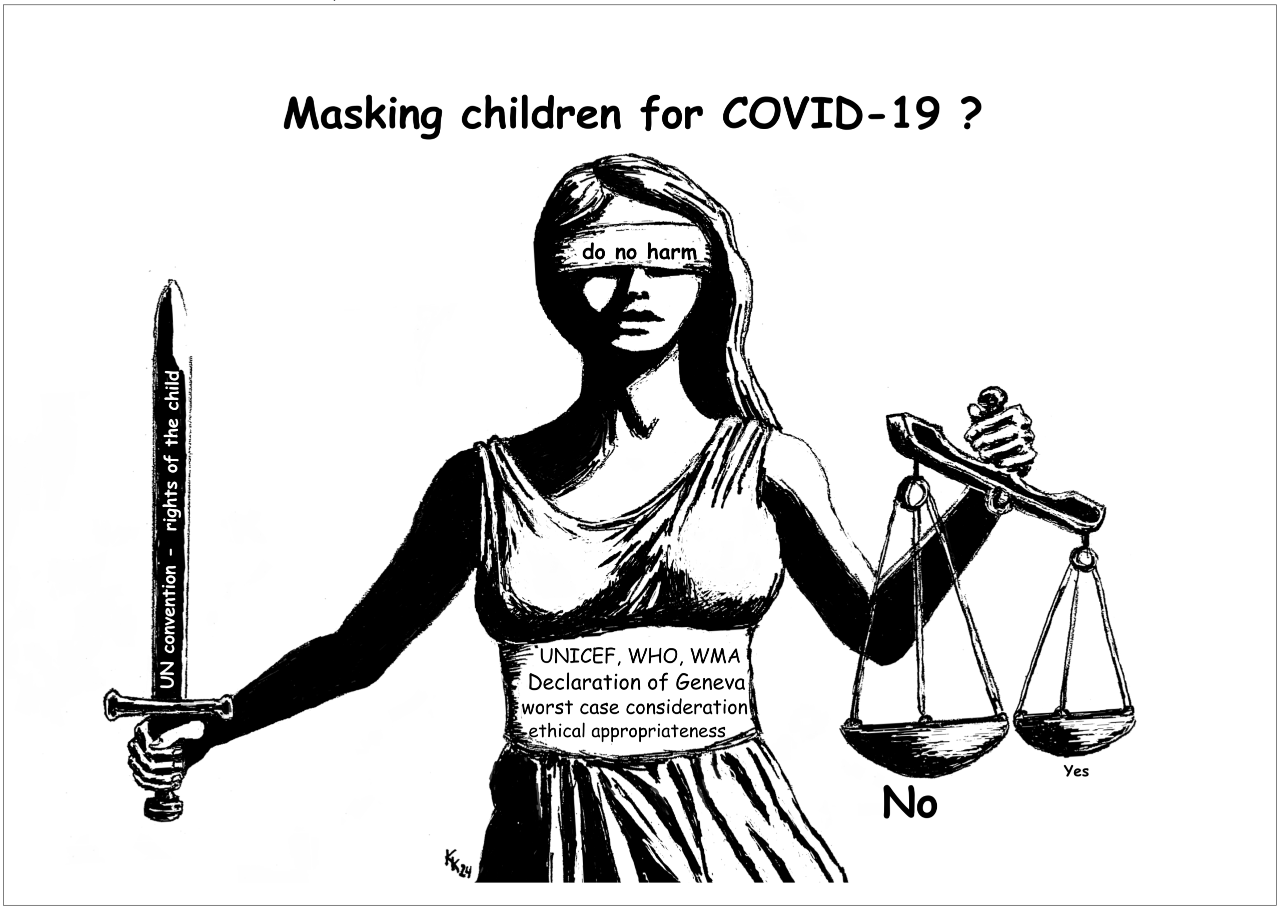

4.8.6. Limitations

As our review was intended to be a rapid literature overview, only one database was searched and no grey literature included in the discussion. Thus, the adverse mask effects and risks could be underestimated in our evaluation. Additionally, as we included only data that were extracted from or with individuals under 18 years of age who had already been born, our review could not go into further detail on early life such as fetuses in the womb. However, from a scientific point of view, a risk for mask-bearing pregnant women is known [27].

Due to a lack of further literature, we were unable to discuss the effects of mask wearing and excessive hygiene on the immune system in relation to the deprivation of natural bacterial stimulation. Possible consequences, however, are apparently a higher susceptibility to infections, also in the form of the well-described waves of infections with rhinoviruses, RSV and streptococci following such measures [434–437].

5. Conclusions

Of course, some individual and rare scenarios exist that justify the use of masks for children as part of a balanced risk-benefit analysis with a short period of a few minutes wearing the mask. For example, as a short-term protection in the event of a bush fire with strong smoke development, or as a source control in a child suffering from open tuberculosis. However, a mask recommendation should always remain a medical advice, in the sense of balanced prevention, not more. But the decision to mandate masks by law for schoolchildren and even younger during the SARS-CoV-2-pandemic, e.g. for entire school days was driven solely by political motives, rather by scientific and medical ones as there is no scientifically convincing empirical evidence to favour such a use of masks against viruses in children (section 4.8.1).

Moving forward, we must learn from past mistakes and must prioritise an evidence-based approach: ensuring interventions involving children are always made in their best interests.

In view of the available literature (Supplement B: Tables S1-S6) and section 4.8., the risk-benefit analysis (section 4.8.5.) is clearly unfavourable for masks in viral pandemics (Figure 5), as masks bear a lot of adverse effects and risks (Figures 2-4) including inacceptable carbon dioxide breathing and the MIES (Figure 4 and section 4.8.4., Supplement B: Tables S3, S4, S5), but do not offer convincing protection against viruses in real-life scenarios from an empirical view (Table E, Supplement A, Table S1, Supplement B).

Other non-pharmaceutical health preserving interventions for children, such as good hand hygiene, improved indoor ventilation and absence from school due to illness, do not engage the legal complexities of mask-wearing and are a safer policy option for reducing SARS-CoV-2 and other virus transmissions. Especially as there is more evidence for effectiveness of these measures together with a lower overall risk for adverse effects.
The promotion of a healthy immune system and herd immunity via environmental factors (nutrition, microbiome, physical- and chemical-, as well as psychological- and social-environmental influences) should also be the focus of future research.

Abbreviations

AQG Air Quality Guideline
ASPHER Association of Schools of Public Health in the European Region
BFE bacterial filtration efficiency
CASP Cochrane critical appraisal skills programme
CDC Centers for Disease Control
Cd Cadmium
Co Cobalt
COVID-19 Coronavirus disease of 2019
Cu Copper
DEHP= bis(2-Ethylhexyl)phthalate
DGPI German Society for Paeditric Infectiology
EAP European Academy of Paediatrics
EIS Exercise-Induced Symptoms
EMB Evidence-based Medicine
EMA European Medicines Agency
$ETCO_2$ End-Tidal carbon dioxide
EU European Union
EU-EN European Union - European Norm
FDA Federal Drug Administration
FFP2 Filtering Face Piece 2
FTOH Flurotelomer alcohol
GRADE Grading of Recommendations Assessment, Development and Evaluation
GVP Good Pharmacovigilance Practices
HEPA High Efficiency Particulate Air
HIF-1α hypoxia-inducible factor-1α
IL-1β Interleukin-1β
IOP Intraocular pressure
IPD Invasive Pneumococcal Disease
MDR Medical Device Regulation
MEDLINE medical literature analysis and retrieval system online
MIES Mask Induced Exhaustion Syndrome
MP Microplastics
NID Notifiable Infectious Diseases
NP Nanoplastics
NPI Non-Pharmaceutical Intervention
UNICEF United Nations International Children's Emergency Fund
Pb Lead
$PETCO_2$ Pressure of End-Tidal carbon dioxide
PICO Participants, Intervention, Comparisons, Outcome
PFAS Per- and Polyfluoroalkyl Substances
PRISMA Preferred Reporting Items for Systematic Reviews and Meta-analyses

RCT Randomized Controlled Trial
REMS Risk Evaluation and Mitigation Strategies
ROS Reactive Oxygen Species
SAR Secondary Attack Rates
SARS-CoV-2 Severe acute respiratory syndrome coronavirus 2
Sb Antimony
SwiM Synthesis without Meta-analysis
TiO_2Titanium dioxid
TVOC Total Volatile Organic Compounds
UN United Nations
US United States
US EPA U.S. Environmental Protection Agency
USA United States of America
USA-ASTM United States of America - American Society for Testing and Material
VOC Volatile Organic Compounds
WHO World Health Organisation

Declarations

Consent for publication
All authors have read and agreed to the published version of the manuscript.

Author Contributions
Conceptualization, K.K. and O.H.; methodology, K.K , C.S., S.W., S.K., S.H. and O.H.; software, K.K..; formal analysis, K.K., C.S., S.W., S.K., S.H. and O.H.; investigation, K.K., C.S., S.W., S.K., S.H., and O.H..; writing—original draft preparation, K.K., C.S., S.W., S.K. S.H and O.H.; writing—review and editing K.K., C.S., S.W., S.K., S.H. and O.H.

Availability of Data and materials
All data used originates from accessible publications (referenced publications).

Competing Interests
The authors declare no conflict of interest.

Funding
This research received no external funding.

Institutional Review Board Statement
Not applicable.

Informed Consent Statement

Not applicable, as this paper is a review article.

Clinical trial number
Not applicable, as this paper is a review article.

Human Ethics and Consent to Participate declarations
Not applicable, as this paper is a review article.

Compliance with the Helsinki Declaration
Not applicable, as this paper is a review article.

Literature

1. OECD Environment, Health and Safety Publications. Assessing the Risk of Chemicals to Children's Health: OECD-Wide Survey 2021 Survey Report. *Series on Testing and Assessment, No 376, 27. April* **2023**, *ENV/CBC/MONO(2023)8*, 1–66.
2. Face Covering Policies during the COVID-19 Pandemic Available online: https://ourworldindata.org/grapher/face-covering-policies-covid (accessed on 29 December 2022).
3. Coronavirus Disease (COVID-19): Children and Masks Available online: https://www.who.int/news-room/questions-and-answers/item/q-a-children-and-masks-related-to-covid-19 (accessed on 28 December 2022).
4. Hahn, L.M.; Manny, E.; Dhaliwal, G.; Chikuma, J.; Robinson, J.; Lou, W.; Subbarao, P.; Turvey, S.E.; Simons, E.; Bell, R.C.; et al. Association of COVID-19 Government-Instituted Mask Mandates With Incidence of Mask Use Among Children in Alberta, Canada. *JAMA Netw Open* **2023**, *6*, e2317358, doi:10.1001/jamanetworkopen.2023.17358.
5. Corpuz, J.C.G. Adapting to the Culture of "New Normal": An Emerging Response to COVID-19. *J Public Health (Oxf)* **2021**, *43*, e344–e345, doi:10.1093/pubmed/fdab057.
6. Rowland, L.C.; Klinkhammer, M.D.; Ramirez, D.W.E. Dynamic Masking: A Proposal of Burden-Based Metrics for Masking in K-12 Schools During the COVID-19 Pandemic. *Journal of School Health* **2022**, *92*, 11–19, doi:10.1111/josh.13099.
7. Esposito, S.; Principi, N. To Mask or Not to Mask Children to Overcome COVID-19. *Eur J Pediatr* **2020**, *179*, 1267–1270, doi:10.1007/s00431-020-03674-9.
8. World Health Organization (WHO) Mask Use in the Context of COVID-19: Interim Guidance, 1 December 2020. WHO/2019-nCoV/IPC_Masks/2020.5. **2020**.
9. Examining Our COVID-19 Response: An Update from Federal Officials | The U.S. Senate Committee on Health, Education, Labor & Pensions Available online: https://www.help.senate.gov/hearings/examining-our-covid-19-response-an-update-from-federal-officials (accessed on 26 August 2024).
10. World Health Organization; Fund (UNICEF), U.N.C. WHO - Advice on the Use of Masks for Children in the Community in the Context of COVID-19: Annex to the Advice on the Use of Masks in the Context of COVID-19, 21 August 2020. **2020**.
11. De Brouwer, C. Wearing a Mask, a Universal Solution Against COVID-19 or an Additional Health Risk? **2020**, doi:10.13140/RG.2.2.32273.66403.
12. Ewig, S.; Gatermann, S.; Lemmen, S. Die Maskierte Gesellschaft. *Pneumologie* **2020**, *74*, 405–408, doi:10.1055/a-1199-4525.
13. Kappstein, I. Mund-Nasen-Schutz in der Öffentlichkeit: Keine Hinweise für eine Wirksamkeit. *Krankenhaushygiene up2date* **2020**, *15*, 279–295, doi:10.1055/a-1174-6591.
14. Spitzer, M. Masked Education? The Benefits and Burdens of Wearing Face Masks in Schools during the Current Corona Pandemic. *Trends Neurosci Educ* **2020**, *20*, 100138, doi:10.1016/j.tine.2020.100138.
15. Ladhani, S.N. Face Masking for Children - Time to Reconsider. *Journal of Infection* **2022**, *85*, 623–624, doi:10.1016/j.jinf.2022.09.020.
16. Thomson, S. Mask Mandates for Children during the COVID-19 Pandemic: An International Human Rights Perspective. *Scand J Public Health* **2022**, *50*, 683–685, doi:10.1177/14034948221081087.
17. Gimma, A.; Lal, S. Considerations for Mitigating COVID-19 Related Risks in Schools. *The Lancet Regional Health – Americas* **2021**, *2*, doi:10.1016/j.lana.2021.100077.
18. Qiu, H.; Wu, J.; Hong, L.; Luo, Y.; Song, Q.; Chen, D. Clinical and Epidemiological Features of 36 Children with Coronavirus Disease 2019 (COVID-19) in Zhejiang, China: An Observational

Cohort Study. *The Lancet Infectious Diseases* **2020**, *20*, 689–696, doi:10.1016/S1473-3099(20)30198-5.
19. Dong, Y.; Mo, X.; Hu, Y.; Qi, X.; Jiang, F.; Jiang, Z.; Tong, S. Epidemiology of COVID-19 Among Children in China. *Pediatrics* **2020**, *145*, e20200702, doi:10.1542/peds.2020-0702.
20. Høeg, T.B.; González-Dambrauskas, S.; Prasad, V. The United States' Decision to Mask Children as Young as Two for COVID-19 Has Been Extended into 2023 and beyond: The Implications of This Policy. *Paediatric Respiratory Reviews* **2023**, *47*, 30–32, doi:10.1016/j.prrv.2023.04.004.
21. McBride, D.L. New Guidelines for Children Returning to Sports after Covid-19. *Journal of Pediatric Nursing: Nursing Care of Children and Families* **2021**, *59*, 196–197, doi:10.1016/j.pedn.2021.01.013.
22. Junger, N.; Hirsch, O.; Junger, N.; Hirsch, O. Ethics of Nudging in the COVID-19 Crisis and the Necessary Return to the Principles of Shared Decision Making: A Critical Review. *Cureus* **2024**, *16*, doi:10.7759/cureus.57960.
23. Greenhalgh, T.; MacIntyre, C.R.; Baker, M.G.; Bhattacharjee, S.; Chughtai, A.A.; Fisman, D.; Kunasekaran, M.; Kvalsvig, A.; Lupton, D.; Oliver, M.; et al. Masks and Respirators for Prevention of Respiratory Infections: A State of the Science Review. *Clin Microbiol Rev* **2024**, e0012423, doi:10.1128/cmr.00124-23.
24. Sandlund, J.; Duriseti, R.; Ladhani, S.N.; Stuart, K.; Noble, J.; Høeg, T.B. Child Mask Mandates for COVID-19: A Systematic Review. *Archives of Disease in Childhood* **2023**, doi:10.1136/archdischild-2023-326215.
25. Chandra, A.; Høeg, T.B. Lack of Correlation between School Mask Mandates and Paediatric COVID-19 Cases in a Large Cohort. *Journal of Infection* **2022**, *85*, 671–675, doi:10.1016/j.jinf.2022.09.019.
26. Jefferson, T.; Dooley, L.; Ferroni, E.; Al-Ansary, L.A.; Driel, M.L. van; Bawazeer, G.A.; Jones, M.A.; Hoffmann, T.C.; Clark, J.; Beller, E.M.; et al. Physical Interventions to Interrupt or Reduce the Spread of Respiratory Viruses. *Cochrane Database of Systematic Reviews* **2023**, doi:10.1002/14651858.CD006207.pub6.
27. Kisielinski, K.; Wagner, S.; Hirsch, O.; Klosterhalfen, B.; Prescher, A. Possible Toxicity of Chronic Carbon Dioxide Exposure Associated with Face Mask Use, Particularly in Pregnant Women, Children and Adolescents – A Scoping Review. *Heliyon* **2023**, *9*, doi:10.1016/j.heliyon.2023.e14117.
28. Kisielinski, K.; Hockertz, S.; Hirsch, O.; Korupp, S.; Klosterhalfen, B.; Schnepf, A.; Dyker, G. Wearing Face Masks as a Potential Source for Inhalation and Oral Uptake of Inanimate Toxins – A Scoping Review. *Ecotoxicology and Environmental Safety* **2024**, *275*, 115858, doi:10.1016/j.ecoenv.2023.115858.
29. Kisielinski, K.; Giboni, P.; Prescher, A.; Klosterhalfen, B.; Graessel, D.; Funken, S.; Kempski, O.; Hirsch, O. Is a Mask That Covers the Mouth and Nose Free from Undesirable Side Effects in Everyday Use and Free of Potential Hazards? *International Journal of Environmental Research and Public Health* **2021**, *18*, 4344, doi:10.3390/ijerph18084344.
30. Acuti Martellucci, C.; Flacco, M.E.; Martellucci, M.; Violante, F.S.; Manzoli, L. Inhaled CO2 Concentration While Wearing Face Masks: A Pilot Study Using Capnography. *Environ Health Insights* **2022**, *16*, 11786302221123573, doi:10.1177/11786302221123573.
31. Walach, H.; Traindl, H.; Prentice, J.; Weikl, R.; Diemer, A.; Kappes, A.; Hockertz, S. Carbon Dioxide Rises beyond Acceptable Safety Levels in Children under Nose and Mouth Covering: Results of an Experimental Measurement Study in Healthy Children. *Environmental Research* **2022**, *212*, 113564, doi:10.1016/j.envres.2022.113564.
32. Le, H.H.T.C.; Vien, N.T.; Dang, T.N.; Ware, R.S.; Phung, D.; Thai, P.K.; Ranganathan, S.; Vinh, N.N.; Dung, P.H.T.; Thanh, H.N.; et al. Wearing Masks as a Protective Measure for Children against Traffic-Related Air Pollution: A Comparison of Perceptions between School Children

and Their Caregivers in Ho Chi Minh City, Vietnam. *Tropical Medicine & International Health* **2023**, *28*, 753–762, doi:10.1111/tmi.13923.
33. Beauchamp, J.D.; Mayhew, C.A. Revisiting the Rationale of Mandatory Masking. *J. Breath Res.* **2023**, *17*, 042001, doi:10.1088/1752-7163/acdf12.
34. Sukul, P.; Bartels, J.; Fuchs, P.; Trefz, P.; Remy, R.; Rührmund, L.; Kamysek, S.; Schubert, J.K.; Miekisch, W. Effects of COVID-19 Protective Face Masks and Wearing Durations on Respiratory Haemodynamic Physiology and Exhaled Breath Constituents. *European Respiratory Journal* **2022**, *60*, doi:10.1183/13993003.00009-2022.
35. Høeg, T.B.; González-Dambrauskas, S.; Prasad, V. Does Equipoise Exist for Masking Children for COVID-19? *Public Health in Practice* **2023**, *6*, 100428, doi:10.1016/j.puhip.2023.100428.
36. Directorate-General for Health and Consumers (European Commission) Now known as *Making Risk Assessment More Relevant for Risk Management*; Publications Office of the European Union: LU, 2013; ISBN 978-92-79-31205-2.
37. FRCPC, S.E.S.C.M.Ms.; PhD, P.G.M.F.; MD, W.S.R.; MD, R.B.H. *Evidence-Based Medicine: How to Practice and Teach EBM*; 5th ed.; Elsevier: Edinburgh ; London ; New York, 2018; ISBN 978-0-7020-6296-4.
38. UNICEF. How Many Children Are There in the World? Available online: https://data.unicef.org/how-many/how-many-children-under-18-are-in-the-world/ (accessed on 26 August 2024).
39. World Population Clock: 8.2 Billion People (LIVE, 2024) - Worldometer Available online: https://www.worldometers.info/world-population/ (accessed on 26 August 2024).
40. Faustman, E.M.; Silbernagel, S.M.; Fenske, R.A.; Burbacher, T.M.; Ponce, R.A. Mechanisms Underlying Children’s Susceptibility to Environmental Toxicants. *Environ Health Perspect* **2000**, *108 Suppl 1*, 13–21, doi:10.1289/ehp.00108s113.
41. Scheuplein, R.; Charnley, G.; Dourson, M. Differential Sensitivity of Children and Adults to Chemical Toxicity: I. Biological Basis. *Regulatory Toxicology and Pharmacology* **2002**, *35*, 429–447, doi:10.1006/rtph.2002.1558.
42. Roberge, R. Facemask Use by Children during Infectious Disease Outbreaks. *Biosecur Bioterror* **2011**, *9*, 225–231, doi:10.1089/bsp.2011.0009.
43. Eberhart, M.; Orthaber, S.; Kerbl, R. The Impact of Face Masks on Children-A Mini Review. *Acta Paediatr* **2021**, *110*, 1778–1783, doi:10.1111/apa.15784.
44. Sandlund, J.; Duriseti, R.; Ladhani, S.N.; Stuart, K.; Noble, J.; Beth Høeg, T. Face Masks and Protection against COVID-19 and Other Viral Respiratory Infections: Assessment of Benefits and Harms in Children. *Paediatric Respiratory Reviews* **2024**, doi:10.1016/j.prrv.2024.08.003.
45. Grant, M.J.; Booth, A. A Typology of Reviews: An Analysis of 14 Review Types and Associated Methodologies. *Health Information & Libraries Journal* **2009**, *26*, 91–108, doi:10.1111/j.1471-1842.2009.00848.x.
46. Shamseer, L.; Moher, D.; Clarke, M.; Ghersi, D.; Liberati, A.; Petticrew, M.; Shekelle, P.; Stewart, L.A.; PRISMA-P Group Preferred Reporting Items for Systematic Review and Meta-Analysis Protocols (PRISMA-P) 2015: Elaboration and Explanation. *BMJ* **2015**, *350*, g7647, doi:10.1136/bmj.g7647.
47. *Cochrane Handbook for Systematic Reviews of Interventions*; Julian P. T. Higgins, J.P.T., James Thomas, Jacqueline Chandler, Miranda Cumpston, Tianjing Li, Matthew J. Page, Vivian A. Welch, Eds.; Wiley Cochrane Series; 2nd ed.; WILEY Blackwell, 2019; ISBN 978-1-119-53662-8.
48. Loke, Y.K.; Price, D.; Herxheimer, A.; Cochrane Adverse Effects Methods Group Systematic Reviews of Adverse Effects: Framework for a Structured Approach. *BMC Med Res Methodol* **2007**, *7*, 32, doi:10.1186/1471-2288-7-32.
49. Zorzela, L.; Loke, Y.K.; Ioannidis, J.P.; Golder, S.; Santaguida, P.; Altman, D.G.; Moher, D.; Vohra, S.; Group, P. harms PRISMA Harms Checklist: Improving Harms Reporting in Systematic Reviews. *BMJ* **2016**, *352*, i157, doi:10.1136/bmj.i157.

50. Huang, X.; Lin, J.; Demner-Fushman, D. Evaluation of PICO as a Knowledge Representation for Clinical Questions. *AMIA Annu Symp Proc* **2006**, *2006*, 359–363.
51. Campbell, M.; McKenzie, J.E.; Sowden, A.; Katikireddi, S.V.; Brennan, S.E.; Ellis, S.; Hartmann-Boyce, J.; Ryan, R.; Shepperd, S.; Thomas, J.; et al. Synthesis without Meta-Analysis (SWiM) in Systematic Reviews: Reporting Guideline. *BMJ* **2020**, *368*, l6890, doi:10.1136/bmj.l6890.
52. Morgan, R.L.; Thayer, K.A.; Bero, L.; Bruce, N.; Falck-Ytter, Y.; Ghersi, D.; Guyatt, G.; Hooijmans, C.; Langendam, M.; Mandrioli, D.; et al. GRADE: Assessing the Quality of Evidence in Environmental and Occupational Health. *Environment international* **2016**, *92–93*, 611, doi:10.1016/j.envint.2016.01.004.
53. Tricco, A.C.; Lillie, E.; Zarin, W.; O'Brien, K.; Colquhoun, H.; Kastner, M.; Levac, D.; Ng, C.; Sharpe, J.P.; Wilson, K.; et al. A Scoping Review on the Conduct and Reporting of Scoping Reviews. *BMC Medical Research Methodology* **2016**, *16*, 15, doi:10.1186/s12874-016-0116-4.
54. Pezzullo, A.M.; Axfors, C.; Contopoulos-Ioannidis, D.G.; Apostolatos, A.; Ioannidis, J.P.A. Age-Stratified Infection Fatality Rate of COVID-19 in the Non-Elderly Population. *Environ Res* **2023**, *216*, 114655, doi:10.1016/j.envres.2022.114655.
55. Aven, T. A Risk and Safety Science Perspective on the Precautionary Principle. *Safety Science* **2023**, *165*, 106211, doi:10.1016/j.ssci.2023.106211.
56. Greenhalgh, T.; Schmid, M.B.; Czypionka, T.; Bassler, D.; Gruer, L. Face Masks for the Public during the Covid-19 Crisis. *BMJ* **2020**, *369*, m1435, doi:10.1136/bmj.m1435.
57. Balshem, H.; Helfand, M.; Schünemann, H.J.; Oxman, A.D.; Kunz, R.; Brozek, J.; Vist, G.E.; Falck-Ytter, Y.; Meerpohl, J.; Norris, S.; et al. GRADE Guidelines: 3. Rating the Quality of Evidence. *Journal of Clinical Epidemiology* **2011**, *64*, 401–406, doi:10.1016/j.jclinepi.2010.07.015.
58. *Haschek and Rousseaux's Handbook of Toxicologic Pathology*; 3rd ed.; Elsevier: Amsterdam, 2013; ISBN 978-0-12-415759-0.
59. *Robbins & Cotran Pathologic Basis of Disease*; 10th ed.; Elsevier, 2020; ISBN 978-0-323-53113-9.
60. Concato, J.; Shah, N.; Horwitz, R.I. Randomized, Cotrolled Trials, Observational Studies, and the Hierarchy of Research Design. *N Engl J Med* **2000**, *342*, 1887–1892.
61. Ioannidis, J.P.A.; Lau, J. Completeness of Safety Reporting in Randomized TrialsAn Evaluation of 7 Medical Areas. *JAMA* **2001**, *285*, 437–443, doi:10.1001/jama.285.4.437.
62. Gøtzsche, P.C.; Hróbjartsson, A.; Johansen, H.K.; Haahr, M.T.; Altman, D.G.; Chan, A.-W. Constraints on Publication Rights in Industry-Initiated Clinical Trials. *JAMA* **2006**, *295*, 1641–1646, doi:10.1001/jama.295.14.1645.
63. Pitrou, I.; Boutron, I.; Ahmad, N.; Ravaud, P. Reporting of Safety Results in Published Reports of Randomized Controlled Trials. *Archives of Internal Medicine* **2009**, *169*, 1756–1761, doi:10.1001/archinternmed.2009.306.
64. Vandenbroucke, J.P. Observational Research, Randomised Trials, and Two Views of Medical Science. *PLoS Med* **2008**, *5*, e67, doi:10.1371/journal.pmed.0050067.
65. Bernhard, J.; Theuring, S.; van Loon, W.; Mall, M.A.; Seybold, J.; Kurth, T.; Rubio-Acero, R.; Wieser, A.; Mockenhaupt, F.P. SARS-CoV-2 Seroprevalence in a Berlin Kindergarten Environment: A Cross-Sectional Study, September 2021. *Children* **2024**, *11*, 405, doi:10.3390/children11040405.
66. Jarnig, G.; Kerbl, R.; van Poppel, M.N.M. Effects of Wearing FFP2 Masks on SARS-CoV-2 Infection Rates in Classrooms. *International Journal of Environmental Research and Public Health* **2022**, *19*, 13511, doi:10.3390/ijerph192013511.
67. Orey, F.A.H.; Sodal, A.M.; Mohamoud, J.H.; Garba, B.; Mohamed, I.H.; Adam, M.H.; Dahie, H.A.; Nur, M.A.S.; Dirie, N.I. Investigation of Severe Acute Respiratory Syndrome Coronavirus 2 Antibodies among the Paediatric Population in Mogadishu, Somalia. *Trans R Soc Trop Med Hyg* **2023**, *117*, 139–146, doi:10.1093/trstmh/trac088.

68. Svetina, L.; Košec, A. Wearing Masks to Prevent One Epidemic May Mask Another. *Journal of Infection Prevention* **2023**, *24*, 228–231, doi:10.1177/17571774231191335.
69. Littlecott, H.; Krishnaratne, S.; Burns, J.; Rehfuess, E.; Sell, K.; Klinger, C.; Strahwald, B.; Movsisyan, A.; Metzendorf, M.-I.; Schoenweger, P.; et al. Measures Implemented in the School Setting to Contain the COVID-19 Pandemic. *Cochrane Database Syst Rev* **2024**, *5*, CD015029, doi:10.1002/14651858.CD015029.pub2.
70. Viera, L. Effect of Face Mask on Lowering COVID-19 Incidence in School Settings: A Systematic Review. *Journal of School Health* **2024**, *94*, 878–888, doi:10.1111/josh.13483.
71. Jarnig, G.; Kerbl, R.; van Poppel, M.N.M. How Middle and High School Students Wear Their Face Masks in Classrooms and School Buildings. *Healthcare* **2022**, *10*, 1641, doi:10.3390/healthcare10091641.
72. Satapathy, D.; Babu, T.A.; Bommidi, S.; Marimuthu, Y.; Bhavana, A.M. Prevalence and Predictors of Effective Face Mask Usage Among Children During the COVID-19 Pandemic. *Indian Pediatr* **2024**, *61*, 66–68.
73. Ludvigsson, J.F. Little Evidence for Facemask Use in Children against COVID-19. *Acta Paediatr* **2021**, *110*, 742–743, doi:10.1111/apa.15729.
74. Coma, E.; Català, M.; Méndez-Boo, L.; Alonso, S.; Hermosilla, E.; Alvarez-Lacalle, E.; Pino, D.; Medina, M.; Asso, L.; Gatell, A.; et al. Unravelling the Role of the Mandatory Use of Face Covering Masks for the Control of SARS-CoV-2 in Schools: A Quasi-Experimental Study Nested in a Population-Based Cohort in Catalonia (Spain). *Archives of Disease in Childhood* **2022**, doi:10.1136/archdischild-2022-324172.
75. Juutinen, A.; Sarvikivi, E.; Laukkanen-Nevala, P.; Helve, O. Face Mask Recommendations in Schools Did Not Impact COVID-19 Incidence among 10–12-Year-Olds in Finland – Joinpoint Regression Analysis. *BMC Public Health* **2023**, *23*, 730, doi:10.1186/s12889-023-15624-9.
76. Schonhaut, L.; Costa-Roldan, I.; Oppenheimer, I.; Pizarro, V.; Han, D.; Díaz, F. Scientific Publication Speed and Retractions of COVID-19 Pandemic Original Articles. *Rev Panam Salud Publica* **2023**, *46*, e25, doi:10.26633/rpsp.2022.25.
77. Clark, J. How Covid-19 Bolstered an Already Perverse Publishing System. *BMJ* **2023**, *380*, p689, doi:10.1136/bmj.p689.
78. Holm, S.M.; Miller, M.D.; Balmes, J.R. Health Effects of Wildfire Smoke in Children and Public Health Tools: A Narrative Review. *J Expo Sci Environ Epidemiol* **2021**, *31*, 1–20, doi:10.1038/s41370-020-00267-4.
79. Janapatla, R.P.; Chen, C.-L.; Dudek, A.; Li, H.-C.; Yang, H.-P.; Su, L.-H.; Chiu, C.-H. Serotype Transmission Dynamics and Reduced Incidence of Invasive Pneumococcal Disease Caused by Different Serotypes after Implementation of Non-Pharmaceutical Interventions during COVID-19 Pandemic. *Eur Respir J* **2021**, *58*, 2100978, doi:10.1183/13993003.00978-2021.
80. Xiao, J.; Dai, J.; Hu, J.; Liu, T.; Gong, D.; Li, X.; Kang, M.; Zhou, Y.; Li, Y.; Quan, Y.; et al. Co-Benefits of Nonpharmaceutical Intervention against COVID-19 on Infectious Diseases in China: A Large Population-Based Observational Study. *The Lancet Regional Health - Western Pacific* **2021**, *17*, 100282, doi:10.1016/j.lanwpc.2021.100282.
81. Suess, T.; Remschmidt, C.; Schink, S.B.; Schweiger, B.; Nitsche, A.; Schroeder, K.; Doellinger, J.; Milde, J.; Haas, W.; Koehler, I.; et al. The Role of Facemasks and Hand Hygiene in the Prevention of Influenza Transmission in Households: Results from a Cluster Randomised Trial; Berlin, Germany, 2009-2011. *BMC Infect Dis* **2012**, *12*, 26, doi:10.1186/1471-2334-12-26.
82. Suess, T.; Remschmidt, C.; Schink, S.; Luchtenberg, M.; Haas, W.; Krause, G.; Buchholz, U. Facemasks and Intensified Hand Hygiene in a German Household Trial during the 2009/2010 Influenza A(H1N1) Pandemic: Adherence and Tolerability in Children and Adults. *Epidemiol Infect* **2011**, *139*, 1895–1901, doi:10.1017/S0950268810003006.
83. Science, M.; Caldeira-Kulbakas, M.; Parekh, R.S.; Maguire, B.R.; Carroll, S.; Anthony, S.J.; Bitnun, A.; Bourns, L.E.; Campbell, D.M.; Cohen, E.; et al. Effect of Wearing a Face Mask on Hand-to-Face Contact by Children in a Simulated School Environment: The Back-to-School

COVID-19 Simulation Randomized Clinical Trial. *JAMA Pediatr* **2022**, *176*, 1169–1175, doi:10.1001/jamapediatrics.2022.3833.

84. Villers, J.; Henriques, A.; Calarco, S.; Rognlien, M.; Mounet, N.; Devine, J.; Azzopardi, G.; Elson, P.; Andreini, M.; Tarocco, N.; et al. SARS-CoV-2 Aerosol Transmission in Schools: The Effectiveness of Different Interventions. *Swiss Med Wkly* **2022**, *152*, w30178, doi:10.4414/smw.2022.w30178.
85. Armero, G.; Guitart, C.; Soler-Garcia, A.; Melé, M.; Esteva, C.; Brotons, P.; Muñoz-Almagro, C.; Jordan, I.; Launes, C. Non-Pharmacological Interventions During SARS-CoV-2 Pandemic: Effects on Pediatric Viral Respiratory Infections. *Arch Bronconeumol* **2024**, S0300-2896(24)00183-2, doi:10.1016/j.arbres.2024.05.019.
86. Banholzer, N.; Zürcher, K.; Jent, P.; Bittel, P.; Furrer, L.; Egger, M.; Hascher, T.; Fenner, L. SARS-CoV-2 Transmission with and without Mask Wearing or Air Cleaners in Schools in Switzerland: A Modeling Study of Epidemiological, Environmental, and Molecular Data. *PLOS Medicine* **2023**, *20*, e1004226, doi:10.1371/journal.pmed.1004226.
87. Matsuda, A.; Asayama, K.; Obara, T.; Yagi, N.; Ohkubo, T. Behavioral Changes of Preventive Activities of Influenza among Children in Satellite Cities of a Metropolitan Area of Tokyo, Japan, by the COVID-19 Pandemic. *BMC Public Health* **2023**, *23*, 727, doi:10.1186/s12889-023-15606-x.
88. Sombetzki, M.; Lücker, P.; Ehmke, M.; Bock, S.; Littmann, M.; Reisinger, E.C.; Hoffmann, W.; Kästner, A. Impact of Changes in Infection Control Measures on the Dynamics of COVID-19 Infections in Schools and Pre-Schools. *Front. Public Health* **2021**, *9*, doi:10.3389/fpubh.2021.780039.
89. Theuring, S.; Thielecke, M.; Loon, W. van; Hommes, F.; Hülso, C.; Haar, A. von der; Körner, J.; Schmidt, M.; Böhringer, F.; Mall, M.A.; et al. SARS-CoV-2 Infection and Transmission in School Settings during the Second COVID-19 Wave: A Cross-Sectional Study, Berlin, Germany, November 2020. *Eurosurveillance* **2021**, *26*, 2100184, doi:10.2807/1560-7917.ES.2021.26.34.2100184.
90. Qin, Z.; Shi, L.; Xue, Y.; Lin, H.; Zhang, J.; Liang, P.; Lu, Z.; Wu, M.; Chen, Y.; Zheng, X.; et al. Prevalence and Risk Factors Associated With Self-Reported Psychological Distress Among Children and Adolescents During the COVID-19 Pandemic in China. *JAMA Network Open* **2021**, *4*, e2035487, doi:10.1001/jamanetworkopen.2020.35487.
91. Fox, G.J.; Redwood, L.; Chang, V.; Ho, J. The Effectiveness of Individual and Environmental Infection Control Measures in Reducing the Transmission of Mycobacterium Tuberculosis: A Systematic Review. *Clinical Infectious Diseases* **2021**, *72*, 15–26, doi:10.1093/cid/ciaa719.
92. Sarkar, M. Tuberculosis Infection Prevention and Control. *Indian Journal of Tuberculosis* **2024**, doi:10.1016/j.ijtb.2024.08.011.
93. Kwon, M.; Jang, E.-M.; Yang, W. Mask-Wearing Perception of Preschool Children in Korea during the COVID-19 Pandemic: A Cross-Sectional Study. *International Journal of Environmental Research and Public Health* **2022**, *19*, 11443, doi:10.3390/ijerph191811443.
94. Halbur, M.; Kodak, T.; McKee, M.; Carroll, R.; Preas, E.; Reidy, J.; Cordeiro, M.C. Tolerance of Face Coverings for Children with Autism Spectrum Disorder. *J Appl Behav Anal* **2021**, *54*, 600–617, doi:10.1002/jaba.833.
95. Sivaraman, M.; Virues-Ortega, J.; Roeyers, H. Telehealth Mask Wearing Training for Children with Autism during the COVID-19 Pandemic. *J Appl Behav Anal* **2021**, *54*, 70–86, doi:10.1002/jaba.802.
96. Tamon, H.; Itahashi, T.; Yamaguchi, S.; Tachibana, Y.; Fujino, J.; Igarashi, M.; Kawashima, M.; Takahashi, R.; Shinohara, N.A.; Noda, Y.; et al. Autistic Children and Adolescents with Frequent Restricted Interest and Repetitive Behavior Showed More Difficulty in Social Cognition during Mask-Wearing during the COVID-19 Pandemic: A Multisite Survey. *BMC Psychiatry* **2022**, *22*, 608, doi:10.1186/s12888-022-04249-8.

97. Ammann, P.; Ulyte, A.; Haile, S.R.; Puhan, M.A.; Kriemler, S.; Radtke, T. Perceptions towards Mask Use in School Children during the SARS-CoV-2 Pandemic: Descriptive Results from the Longitudinal Ciao Corona Cohort Study. *Swiss Med Wkly* **2022**, *152*, w30165, doi:10.4414/smw.2022.w30165.
98. Omaleki, V.; Gonzalez, A.F.; Hassani, A.; Flores, M.; Streuli, S.; Guerra, A.W.; Fielding-Miller, R. “They Protect Us as If They Were Our Mom” Masking Attitudes from Freelist Survey Data and Qualitative Interviews in San Diego School Communities. *J Community Health* **2024**, *49*, 17–25, doi:10.1007/s10900-023-01245-1.
99. Aronu, A.E.; Chinawa, J.M.; Nduagubam, O.C.; Ossai, E.N.; Chinawa, A.T.; Igwe, W.C. Maternal Perception of Masking in Children as a Preventive Strategy for COVID-19 in Nigeria: A Multicentre Study. *PLOS ONE* **2020**, *15*, e0242650, doi:10.1371/journal.pone.0242650.
100. Assathiany, R.; Salinier, C.; Béchet, S.; Dolard, C.; Kochert, F.; Bocquet, A.; Levy, C. Face Masks in Young Children During the COVID-19 Pandemic: Parents’ and Pediatricians’ Point of View. *Front. Pediatr.* **2021**, *9*, doi:10.3389/fped.2021.676718.
101. Education Recovery in Early Years Providers: Spring 2022 Available online: https://www.gov.uk/government/publications/education-recovery-in-early-years-providers-spring-2022/education-recovery-in-early-years-providers-spring-2022 (accessed on 1 February 2023).
102. Charney, S.A.; Camarata, S.M.; Chern, A. Potential Impact of the COVID-19 Pandemic on Communication and Language Skills in Children. *Otolaryngol Head Neck Surg* **2021**, *165*, 1–2, doi:10.1177/0194599820978247.
103. Goldin, A.; Weinstein, B.; Shiman, N. How Do Medical Masks Degrade Speech Reception? *The Hearing Review* **2020**, *27*, 8–9.
104. Kwon, M.; Yang, W. Effects of Face Masks and Acoustical Environments on Speech Recognition by Preschool Children in an Auralised Classroom. *Appl Acoust* **2023**, *202*, 109149, doi:10.1016/j.apacoust.2022.109149.
105. Lalonde, K.; Buss, E.; Miller, M.K.; Leibold, L.J. Face Masks Impact Auditory and Audiovisual Consonant Recognition in Children With and Without Hearing Loss. *Front. Psychol.* **2022**, *13*, doi:10.3389/fpsyg.2022.874345.
106. Taxacher, T.; Rupp, M.; Pauli, C.; Profanter, C.; Dejakum, K.; Steindl, R.; Ostertag, P. [Impact of face masks on speech intelligibility of normal hearing children]. *Laryngorhinootologie* **2023**, *102*, 669–674, doi:10.1055/a-2013-2888.
107. Flaherty, M.M.; Arzuaga, B.; Bottalico, P. The Effects of Face Masks on Speech-in-Speech Recognition for Children and Adults. *Int J Audiol* **2023**, *62*, 1014–1021, doi:10.1080/14992027.2023.2168218.
108. Carnevali, L.; Gui, A.; Jones, E.J.H.; Farroni, T. Face Processing in Early Development: A Systematic Review of Behavioral Studies and Considerations in Times of COVID-19 Pandemic. *Front. Psychol.* **2022**, *13*, doi:10.3389/fpsyg.2022.778247.
109. Stajduhar, A.; Ganel, T.; Avidan, G.; Rosenbaum, R.S.; Freud, E. Face Masks Disrupt Holistic Processing and Face Perception in School-Age Children. *Cognitive Research: Principles and Implications* **2022**, *7*, 9, doi:10.1186/s41235-022-00360-2.
110. Drössler, S.; Horvath, K.; Freiberg, A.; Kämpf, D.; Spura, A.; Buhs, B.; Seidler, A. [Effects of Wearing Face Masks to Prevent Infectious Diseases On Children’s and Adolescents’ Well-Being and Behavior: An Interview Study]. *Gesundheitswesen* **2023**, *85*, 688–696, doi:10.1055/a-2075-7814.
111. Gori, M.; Schiatti, L.; Amadeo, M.B. Masking Emotions: Face Masks Impair How We Read Emotions. *Front Psychol* **2021**, *12*, 669432, doi:10.3389/fpsyg.2021.669432.
112. Bourke, L.; Lingwood, J.; Gallagher-Mitchell, T.; López-Pérez, B. The Effect of Face Mask Wearing on Language Processing and Emotion Recognition in Young Children. *J Exp Child Psychol* **2023**, *226*, 105580, doi:10.1016/j.jecp.2022.105580.

113. Surrain, S.; Mesa, M.P.; Assel, M.A.; Zucker, T.A. Does Assessor Masking Affect Kindergartners' Performance on Oral Language Measures? A COVID-19 Era Experiment With Children From Diverse Home Language Backgrounds. *Lang Speech Hear Serv Sch* **2023**, *54*, 1323–1332, doi:10.1044/2023_LSHSS-22-00197.
114. Singh, L.; Quinn, P.C. Effects of Face Masks on Language Comprehension in Bilingual Children. *Infancy* **2023**, *28*, 738–753, doi:10.1111/infa.12543.
115. Chester, M.; Plate, R.C.; Powell, T.; Rodriguez, Y.; Wagner, N.J.; Waller, R. The COVID-19 Pandemic, Mask-Wearing, and Emotion Recognition during Late-Childhood. *Social Development* **2023**, *32*, 315–328, doi:10.1111/sode.12631.
116. Ger, E.; Manfredi, M.; Osório, A.A.C.; Ribeiro, C.F.; Almeida, A.; Güdel, A.; Calbi, M.; Daum, M.M. Duration of Face Mask Exposure Matters: Evidence from Swiss and Brazilian Kindergartners' Ability to Recognise Emotions. *Cognition and Emotion* **2024**, *38*, 857–871, doi:10.1080/02699931.2024.2331795.
117. Miyazaki, Y.; Kamatani, M.; Tsurumi, S.; Suda, T.; Wakasugi, K.; Matsunaga, K.; Kawahara, J.I. Effects of Wearing an Opaque or Transparent Face Mask on the Perception of Facial Expressions: A Comparative Study between Japanese School-Aged Children and Adults. *Perception* **2023**, *52*, 782–798, doi:10.1177/03010066231200693.
118. Gil, S.; Le Bigot, L. Emotional Face Recognition When a Colored Mask Is Worn: A Cross-Sectional Study. *Sci Rep* **2023**, *13*, 174, doi:10.1038/s41598-022-27049-2.
119. Boucher, J.D.; Ekman, P. Facial Areas and Emotional Information. *Journal of Communication* **1975**, *25*, 21–29, doi:10.1111/j.1460-2466.1975.tb00577.x.
120. Eisenbarth, H.; Alpers, G.W. Happy Mouth and Sad Eyes: Scanning Emotional Facial Expressions. *Emotion* **2011**, *11*, 860–865, doi:10.1037/a0022758.
121. Guarnera, M.; Hichy, Z.; Cascio, M.I.; Carrubba, S. Facial Expressions and Ability to Recognize Emotions From Eyes or Mouth in Children. *Europe's Journal of Psychology* **2015**, *11*, 183–196, doi:10.5964/ejop.v11i2.890.
122. Wegrzyn, M.; Vogt, M.; Kireclioglu, B.; Schneider, J.; Kissler, J. Mapping the Emotional Face. How Individual Face Parts Contribute to Successful Emotion Recognition. *PLOS ONE* **2017**, *12*, e0177239, doi:10.1371/journal.pone.0177239.
123. Gagnon, M.; Gosselin, P.; Maassarani, R. Children's Ability to Recognize Emotions From Partial and Complete Facial Expressions. *The Journal of Genetic Psychology* **2014**, *175*, 416–430, doi:10.1080/00221325.2014.941322.
124. Kestenbaum, R. Feeling Happy versus Feeling Good: The Processing of Discrete and Global Categories of Emotional Expressions by Children and Adults. *Developmental Psychology* **1992**, *28*, 1132–1142, doi:10.1037/0012-1649.28.6.1132.
125. Proverbio, A.M.; Cerri, A. The Recognition of Facial Expressions Under Surgical Masks: The Primacy of Anger. *Frontiers in Neuroscience* **2022**, *16*.
126. Rinck, M.; Primbs, M.A.; Verpaalen, I.A.M.; Bijlstra, G. Face Masks Impair Facial Emotion Recognition and Induce Specific Emotion Confusions. *Cognitive Research: Principles and Implications* **2022**, *7*, 83, doi:10.1186/s41235-022-00430-5.
127. Tsantani, M.; Podgajecka, V.; Gray, K.L.H.; Cook, R. How Does the Presence of a Surgical Face Mask Impair the Perceived Intensity of Facial Emotions? *PLOS ONE* **2022**, *17*, e0262344, doi:10.1371/journal.pone.0262344.
128. Giordano, K.; Palmieri, C.S.; LaTourette, R.; Godoy, K.M.; Denicola, G.; Paulino, H.; Kosecki, O. Face Masks and Emotion Literacy in Preschool Children: Implications During the COVID-19 Pandemic. *Early Child Educ J* **2022**, 1–9, doi:10.1007/s10643-022-01400-8.
129. Freiberg, A.; Horvath, K.; Hahne, T.M.; Drössler, S.; Kämpf, D.; Spura, A.; Buhs, B.; Reibling, N.; De Bock, F.; Apfelbacher, C.; et al. [Impact of wearing face masks in public to prevent infectious diseases on the psychosocial development in children and adolescents: a systematic review]. *Bundesgesundheitsblatt Gesundheitsforschung Gesundheitsschutz* **2021**, *64*, 1592–1602, doi:10.1007/s00103-021-03443-5.

130. Liu, S.; Li, X.; Sun, R. The Effect of Masks on Infants' Ability to Fast-Map and Generalize New Words. *J Child Lang* **2024**, *51*, 637–655, doi:10.1017/S0305000923000697.
131. Coelho, S.G.; Segovia, A.; Anthony, S.J.; Lin, J.; Pol, S.; Crosbie, J.; Science, M.; Matava, C.T.; Parekh, R.S.; Caldeira-Kulbakas, M.; et al. Return to School and Mask-Wearing in Class during the COVID-19 Pandemic: Student Perspectives from a School Simulation Study. *Paediatrics & Child Health* **2022**, *27*, S15–S21, doi:10.1093/pch/pxab102.
132. Nobrega, M.; Opice, R.; Lauletta, M.M.; Nobrega, C.A. How Face Masks Can Affect School Performance. *Int J Pediatr Otorhinolaryngol* **2020**, *138*, 110328, doi:10.1016/j.ijporl.2020.110328.
133. Shaw, C.A.; Lee, K.R.; Williams, A.; Shaw, N.A.; Weeks, D.; Jackson, L.; Williams, K.N. Best Practices for Communication While Wearing Facemasks: A Scoping Review. *J Nurs Scholarsh* **2024**, *56*, 227–238, doi:10.1111/jnu.12939.
134. Schwarz, J.; Li, K.K.; Sim, J.H.; Zhang, Y.; Buchanan-Worster, E.; Post, B.; Gibson, J.L.; McDougall, K. Semantic Cues Modulate Children's and Adults' Processing of Audio-Visual Face Mask Speech. *Front. Psychol.* **2022**, *13*, doi:10.3389/fpsyg.2022.879156.
135. Mitsven, S.G.; Perry, L.K.; Jerry, C.M.; Messinger, D.S. Classroom Language during COVID-19: Associations between Mask-Wearing and Objectively Measured Teacher and Preschooler Vocalizations. *Front. Psychol.* **2022**, *13*, doi:10.3389/fpsyg.2022.874293.
136. Schlegtendal, A.; Eitner, L.; Falkenstein, M.; Hoffmann, A.; Lücke, T.; Sinningen, K.; Brinkmann, F. To Mask or Not to Mask—Evaluation of Cognitive Performance in Children Wearing Face Masks during School Lessons (MasKids). *Children* **2022**, *9*, 95, doi:10.3390/children9010095.
137. Rodeheffer, C.D.; Chabal, S.; Clarke, J.M.; Fothergill, D.M. Acute Exposure to Low-to-Moderate Carbon Dioxide Levels and Submariner Decision Making. *Aerospace Medicine and Human Performance* **2018**, *89*, 520–525, doi:10.3357/AMHP.5010.2018.
138. Hurley, R.F.; Belyamani, M.A.; Djamasbi, S.; Somasse, G.B.; Strauss, S.; Zhang, H.; Zhang, J. (Jensen); Liu, S. High CO2 Exposure Due to Facemask Wear Is Unlikely to Impair Cognition Even in a Warm Environment after a Long-Term Adaptation. *Energy and Built Environment* **2024**, doi:10.1016/j.enbenv.2024.05.005.
139. Smerdon, D. The Effect of Masks on Cognitive Performance. *Proceedings of the National Academy of Sciences* **2022**, *119*, e2206528119, doi:10.1073/pnas.2206528119.
140. Zhong, Q.; Song, J.; Shi, D.; Dung, C.-H. Protective Facemask-Induced Facial Thermal Stress and Breathing Burden during Exercise in Gyms. *Building and Environment* **2023**, *244*, 110840, doi:10.1016/j.buildenv.2023.110840.
141. Azuma, K.; Kagi, N.; Yanagi, U.; Osawa, H. Effects of Low-Level Inhalation Exposure to Carbon Dioxide in Indoor Environments: A Short Review on Human Health and Psychomotor Performance. *Environment International* **2018**, *121*, 51–56, doi:10.1016/j.envint.2018.08.059.
142. Allen, J.G.; MacNaughton, P.; Cedeno-Laurent, J.G.; Cao, X.; Flanigan, S.; Vallarino, J.; Rueda, F.; Donnelly-McLay, D.; Spengler, J.D. Airplane Pilot Flight Performance on 21 Maneuvers in a Flight Simulator under Varying Carbon Dioxide Concentrations. *J Expo Sci Environ Epidemiol* **2019**, *29*, 457–468, doi:10.1038/s41370-018-0055-8.
143. Pang, L.; Zhang, J.; Cao, X.; Wang, X.; Liang, J.; Zhang, L.; Guo, L. The Effects of Carbon Dioxide Exposure Concentrations on Human Vigilance and Sentiment in an Enclosed Workplace Environment. *Indoor Air* **2021**, *31*, 467–479, doi:10.1111/ina.12746.
144. Du, B.; Tandoc, M.C.; Mack, M.L.; Siegel, J.A. Indoor CO2 Concentrations and Cognitive Function: A Critical Review. *Indoor Air* **2020**, *30*, 1067–1082, doi:10.1111/ina.12706.
145. Zhang, X.; Wargocki, P.; Lian, Z.; Thyregod, C. Effects of Exposure to Carbon Dioxide and Bioeffluents on Perceived Air Quality, Self-Assessed Acute Health Symptoms, and Cognitive Performance. *Indoor Air* **2017**, *27*, 47–64, doi:10.1111/ina.12284.
146. Jagim, A.R.; Dominy, T.A.; Camic, C.L.; Wright, G.; Doberstein, S.; Jones, M.T.; Oliver, J.M. Acute Effects of the Elevation Training Mask on Strength Performance in Recreational

Weight Lifters. *J Strength Cond Res* **2018**, *32*, 482–489, doi:10.1519/JSC.0000000000002308.
147. Johnson, A.T. Respirator Masks Protect Health but Impact Performance: A Review. *J Biol Eng* **2016**, *10*, doi:10.1186/s13036-016-0025-4.
148. Kyung, S.Y.; Kim, Y.; Hwang, H.; Park, J.-W.; Jeong, S.H. Risks of N95 Face Mask Use in Subjects With COPD. *Respir Care* **2020**, *65*, 658–664, doi:10.4187/respcare.06713.
149. Liu, C.; Li, G.; He, Y.; Zhang, Z.; Ding, Y. Effects of Wearing Masks on Human Health and Comfort during the COVID-19 Pandemic. *IOP Conf. Ser.: Earth Environ. Sci.* **2020**, *531*, 012034, doi:10.1088/1755-1315/531/1/012034.
150. Lang, X.; Vasquez, N.G.; Liu, W.; Wyon, D.P.; Wargocki, P. Effects of Wearing Masks Indoors on the Cognitive Performance and Physiological and Subjective Responses of Healthy Young Adults. *Building and Environment* **2024**, *252*, 111248, doi:10.1016/j.buildenv.2024.111248.
151. Rebmann, T.; Carrico, R.; Wang, J. Physiologic and Other Effects and Compliance with Long-Term Respirator Use among Medical Intensive Care Unit Nurses. *Am J Infect Control* **2013**, *41*, 1218–1223, doi:10.1016/j.ajic.2013.02.017.
152. Rosner, E. Adverse Effects of Prolonged Mask Use among Healthcare Professionals during COVID-19. *Journal of Infectious Diseases and Epidemiology* **2020**, *6:130*, doi:10.23937/2474-3658/1510130.
153. Tornero-Aguilera, J.F.; Clemente-Suárez, V.J. Cognitive and Psychophysiological Impact of Surgical Mask Use during University Lessons. *Physiol Behav* **2021**, 113342–113342.
154. Vakharia, R.J.; Jani, I.; Yadav, S.; Kurian, T. To Study Acute Changes in Brain Oxygenation on MRI in Healthcare Workers Using N95 Mask and PPE Kits for Six Hours a Day. *Indian J Radiol Imaging* **2021**, *31*, 893–900, doi:10.1055/s-0041-1741086.
155. Preest, E.; Greenhalgh, T.; Farrier, C.; van der Westhuizen, H.-M. Children’s Experiences of Mask-Wearing: A Systemic Review and Narrative Synthesis. *Journal of Evaluation in Clinical Practice* **2024**, *30*, 585–621, doi:10.1111/jep.13982.
156. Smart, N.R.; Horwell, C.J.; Smart, T.S.; Galea, K.S. Assessment of the Wearability of Facemasks against Air Pollution in Primary School-Aged Children in London. *International Journal of Environmental Research and Public Health* **2020**, *17*, 3935, doi:10.3390/ijerph17113935.
157. Chhabra, K.; Sood, S.; Bhatia, H.P.; Sharma, N.; Singh, A. Comparative Evaluation of Psychophysiological Response of Children with Special Health Care Needs to Use of Facemask-Eyeshield and Visor in a Dental Setting-A Cross-Sectional Study. *Spec Care Dentist* **2020**, *40*, 145–150, doi:10.1111/scd.12451.
158. Wild, B.M.; Kornfeld, B. Facial Recognition: More Than Just a Phone Problem. *Pediatr Ann* **2021**, *50*, e52–e54, doi:10.3928/19382359-20210118-01.
159. Ratchatavech, K.; Techasatian, L.; Panombualert, S.; Uppala, R. The Adverse and Advantage Effects of Wearing a Facemask in Thai Children: A Survey During the COVID-19 Pandemic. *J Prim Care Community Health* **2022**, *13*, 21501319221131704, doi:10.1177/21501319221131704.
160. Schwarz, S.; Jenetzky, E.; Krafft, H.; Maurer, T.; Martin, D. Corona child studies “Co-Ki”: first results of a Germany-wide register on mouth and nose covering (mask) in children. *Monatsschr Kinderheilkd* **2021**, *169*, 353–365, doi:10.1007/s00112-021-01133-9.
161. Beytout, Q.; Pepiot, J.; Maruani, A.; Devulder, D.; Aubert, R.; Beylot-Barry, M.; Amici, J.-M.; Jullien, D.; Mahé, E.; Association France Psoriasis; et al. Impact of the COVID-19 Pandemic on Children with Psoriasis. *Ann Dermatol Venereol* **2021**, *148*, 106–111, doi:10.1016/j.annder.2021.01.005.
162. Kaliyadan, F.; Ashique, K.T.; Jayasree, P. Increased Incidence of Facial Pityriasis Versicolor in Children during the COVID-19 Pandemic-A Consequence of Mask Usage? *Pediatr Dermatol* **2022**, *39*, 834–835, doi:10.1111/pde.15043.

163. Shoaib, M.; Chomayil, Y.; Zafar, M. Corneal Abrasion Due to Face Mask in Children: A Novel and Potential Mechanism of Injury Related to COVID 19 Pandemic. *Vis J Emerg Med* **2022**, *27*, 101290, doi:10.1016/j.visj.2022.101290.
164. Zanotti, B.; Parodi, P.C.; Riccio, M.; De Francesco, F.; Zingaretti, N. Can the Elastic of Surgical Face Masks Stimulate Ear Protrusion in Children? *Aesth Plast Surg* **2020**, *44*, 1947–1950, doi:10.1007/s00266-020-01833-9.
165. Ali, F.M.; Wasli, A.S.; Hobani, A.H.; Faraj, S.H.A.; Mashiakhy, E.H.; Alkhayrat, A.M.; Khawaji, R.A. Prevention of Ear-looped Face Mask-induced Pressure Injury on Ears: A Technical Report with Review of Literature. *Advancements in Life Sciences* **2024**, *11*, 539–543, doi:10.62940/als.v11i3.2636.
166. WHO Conference, I.H. WHO - Constitution of the World Health Organization. 1946. *Bulletin of the World Health Organization* **2002**, *80*, 983–984.
167. Amirav, I.; Lavie, M. Spurious Asthma Presentation during COVID-19. *Children* **2022**, *9*, 5, doi:10.3390/children9010005.
168. Wang, Y.; Tse, G.; Li, G. Running with Face Masks or Respirators Can Be Detrimental to the Respiratory and Cardiovascular Systems. *Cardiovascular Innovations and Applications* **2021**, *6*, 63, doi:10.15212/CVIA.2021.0010.
169. Reychler, G.; Standaert, M.; Audag, N.; Caty, G.; Robert, A.; Poncin, W. Effects of Surgical Facemasks on Perceived Exertion during Submaximal Exercise Test in Healthy Children. *Eur J Pediatr* **2022**, *181*, 2311–2317, doi:10.1007/s00431-022-04430-x.
170. Mallet, M.C.; Hitzler, M.; Lurà, M.; Kuehni, C.E.; Regamey, N. Facemasks Do Not Lead to Abnormal Gas Exchange during Treadmill Exercise Testing in Children. *ERJ Open Res* **2022**, *8*, 00613–02021, doi:10.1183/23120541.00613-2021.
171. Hodges, M.; Freigeh, G.E.; Troost, J.; Baptist, A.P.; Gupta, M. Assessment of Mask Use on Oxygen Saturation in Adults and Children with Asthma. *Allergy Asthma Proc* **2024**, *45*, 24–32, doi:10.2500/aap.2024.45.230078.
172. Schulte-Körne, B.; Hollmann, W.; Vassiliadis, A.; Predel, H.-G. [Effects of surgical face masks on exercise performance and perceived exertion of exercise in well-trained healthy boys]. *Wien Med Wochenschr* **2022**, *172*, 59–62, doi:10.1007/s10354-021-00851-9.
173. Weigelt, A.; Schöffl, I.; Rottermann, K.; Wällisch, W.; Müller, S.K.; Dittrich, S.; Hübner, M.J. Sports despite Masks: No Negative Effects of FFP2 Face Masks on Cardiopulmonary Exercise Capacity in Children. *Eur J Pediatr* **2024**, *183*, 639–648, doi:10.1007/s00431-023-05316-2.
174. Sukul, P.; Richter, A.; Junghanss, C.; Schubert, J.K.; Miekisch, W. Origin of Breath Isoprene in Humans Is Revealed via Multi-Omic Investigations. *Commun Biol* **2023**, *6*, 1–12, doi:10.1038/s42003-023-05384-y.
175. Ahmadi, A.; Sabri, M.R.; Navabi, Z.S. Effect of Face Mask on Pulmonary Artery Pressure during Echocardiography in Children and Adolescents. *Clin Exp Pediatr* **2024**, *67*, 161–167, doi:10.3345/cep.2023.01172.
176. Lubrano, R.; Bloise, S.; Testa, A.; Marcellino, A.; Dilillo, A.; Mallardo, S.; Isoldi, S.; Martucci, V.; Sanseviero, M.; Del Giudice, E.; et al. Assessment of Respiratory Function in Infants and Young Children Wearing Face Masks During the COVID-19 Pandemic. *JAMA Netw Open* **2021**, *4*, e210414, doi:10.1001/jamanetworkopen.2021.0414.
177. Lubrano, R.; Bloise, S.; Marcellino, A.; Ciolli, C.P.; Testa, A.; De Luca, E.; Dilillo, A.; Mallardo, S.; Isoldi, S.; Martucci, V.; et al. Effects of N95 Mask Use on Pulmonary Function in Children. *J Pediatr* **2021**, *237*, 143–147, doi:10.1016/j.jpeds.2021.05.050.
178. Lubrano, R.; Bloise, S.; Marcellino, A.; Proietti Ciolli, C.; Testa, A.; De Luca, E.; Dilillo, A.; Mallardo, S.; Isoldi, S.; Martucci, V.; et al. Assessment of Respiratory Function in Children Wearing a N95 Mask with or without an Exhalation Valve: Data Compared. *Data Brief* **2021**, *39*, 107550, doi:10.1016/j.dib.2021.107550.

179. Lubrano, R.; Bloise, S.; Sanseviero, M.; Marcellino, A.; Proietti Ciolli, C.; De Luca, E.; Testa, A.; Dilillo, A.; Mallardo, S.; Isoldi, S.; et al. Assessment of Cardio-Respiratory Function in Overweight and Obese Children Wearing Face Masks during the COVID-19 Pandemic. *Children (Basel)* **2022**, *9*, 1053, doi:10.3390/children9071053.
180. Brooks, J.P.; Layman, J.; Willis, J. Physiologic Effects of Surgical Masking in Children versus Adults. *PeerJ* **2023**, *11*, e15474, doi:10.7717/peerj.15474.
181. Goh, D.Y.T.; Mun, M.W.; Lee, W.L.J.; Teoh, O.H.; Rajgor, D.D. A Randomised Clinical Trial to Evaluate the Safety, Fit, Comfort of a Novel N95 Mask in Children. *Scientific Reports* **2019**, *9*, 18952, doi:10.1038/s41598-019-55451-w.
182. Happernegg, R.; Kerbl, R. The Influence of Wearing Surgical and FFP2 Face Masks on Physiological Parameters in Children and Adolescents - a Pilot Study. *Klin Padiatr* **2023**, *235*, 101–102, doi:10.1055/a-1976-1520.
183. Castro, P.A.S.V. de; Freire, B.R.; Petroianu, A. Effects of Face Mask on Pulse Rate and Blood Oxygenation. *Einstein (Sao Paulo)* **2023**, *21*, eAO0349, doi:10.31744/einstein_journal/2023AO0349.
184. Canellas, E.; Vera, P.; Nerin, C.; Goshawk, J.; Dreolin, N. Migration of Contaminants from Printed Masks for Children to Saliva Simulant Using Liquid Chromatography Coupled to Ion Mobility-Time of Flight-Mass Spectrometry and Gas Chromatography-Mass Spectrometry. *Ecotoxicol Environ Saf* **2023**, *267*, 115644, doi:10.1016/j.ecoenv.2023.115644.
185. Ryu, H.; Kim, Y.-H. Measuring the Quantity of Harmful Volatile Organic Compounds Inhaled through Masks. *Ecotoxicology and Environmental Safety* **2023**, *256*, 114915, doi:10.1016/j.ecoenv.2023.114915.
186. Jefferson, T.; Mar, C.B.D.; Dooley, L.; Ferroni, E.; Al-Ansary, L.A.; Bawazeer, G.A.; Driel, M.L. van; Jones, M.A.; Thorning, S.; Beller, E.M.; et al. Physical Interventions to Interrupt or Reduce the Spread of Respiratory Viruses. *Cochrane Database of Systematic Reviews* **2020**, doi:10.1002/14651858.CD006207.pub5.
187. World Health Organization Non-Pharmaceutical Public Health Measures for Mitigating the Risk and Impact of Epidemic and Pandemic Influenza: Annex: Report of Systematic Literature Reviews. **2019**.
188. Villani, A.; Bozzola, E.; Staiano, A.; Agostiniani, R.; Del Vecchio, A.; Zamperini, N.; Marino, F.; Vecchio, D.; Corsello, G. Facial Masks in Children: The Position Statement of the Italian Pediatric Society. *Italian Journal of Pediatrics* **2020**, *46*, 132, doi:10.1186/s13052-020-00898-1.
189. Chu, D.K.; Akl, E.A.; Duda, S.; Solo, K.; Yaacoub, S.; Schünemann, H.J.; Chu, D.K.; Akl, E.A.; El-harakeh, A.; Bognanni, A.; et al. Physical Distancing, Face Masks, and Eye Protection to Prevent Person-to-Person Transmission of SARS-CoV-2 and COVID-19: A Systematic Review and Meta-Analysis. *The Lancet* **2020**, *395*, 1973–1987, doi:10.1016/S0140-6736(20)31142-9.
190. Leung, N.H.L.; Chu, D.K.W.; Shiu, E.Y.C.; Chan, K.-H.; McDevitt, J.J.; Hau, B.J.P.; Yen, H.-L.; Li, Y.; Ip, D.K.M.; Peiris, J.S.M.; et al. Respiratory Virus Shedding in Exhaled Breath and Efficacy of Face Masks. *Nat Med* **2020**, *26*, 676–680, doi:10.1038/s41591-020-0843-2.
191. Gyawali, M. Protection of Our Future Generation: Use of Face Masks in Children amidst COVID-19 Era. *JNMA J Nepal Med Assoc* **2021**, *59*, 818–820, doi:10.31729/jnma.6705.
192. Huppertz, H.-I.; Berner, R.; Schepker, R.; Kopp, M.; Oberle, A.; Fischbach, T.; Rodeck, B.; Knuf, M.; Keller, M.; Simon, A.; et al. [Use of masks by children to prevent infection with SARS-CoV-2]. *Monatsschr Kinderheilkd* **2021**, *169*, 52–56, doi:10.1007/s00112-020-01090-9.
193. Lopes, H.; Middleton, J.; De Guchtenaere, A.; Hadjipanayis, A. COVID-19 and the Use of Masks by Children. Statement From the Association of Schools of Public Health in the European Region and the European Academy of Paediatrics. *Front. Pediatr.* **2021**, *9*, doi:10.3389/fped.2021.580150.

194. Moschovis, P.P.; Yonker, L.M.; Shah, J.; Singh, D.; Demokritou, P.; Kinane, T.B. Aerosol Transmission of SARS-CoV-2 by Children and Adults during the COVID-19 Pandemic. *Pediatr Pulmonol* **2021**, *56*, 1389–1394, doi:10.1002/ppul.25330.
195. Bagus, P.; Peña-Ramos, J.A.; Sánchez-Bayón, A. COVID-19 and the Political Economy of Mass Hysteria. *International Journal of Environmental Research and Public Health* **2021**, *18*, 1376, doi:10.3390/ijerph18041376.
196. Cabrera, J. Dangerous Pathogens Found on Local Residents' Face Masks. *Alachua Chronicle* 2021.
197. Kisielinski, K.; Wojtasik, B. Suitability of Rose Bengal Sodium Salt Staining for Visualisation of Face Mask Contamination by Living Organisms. *AIMSES* **2022**, *9*, 218–231, doi:10.3934/environsci.2022015.
198. Kisielinski, K.; Wojtasik, B.; Zalewska, A.; Livermore, D.M.; Jurczak-Kurek, A. The Bacterial Burden of Worn Face Masks -Observational Research and Literature Review. *Front. Public Health* **2024**, *12*, doi:10.3389/fpubh.2024.1460981.
199. Amara, H.; Tadj, A.; Mahdad, Y.M.; Seddiki, S.M.L. Risques Infectieux de l'utilisation Répétée et Prolongée Des Masques Chirurgicaux Pendant La Covid-19: Surgical Masks and Covid-19. Utility. *Genetics & Biodiversity Journal* **2024**, *8*, 85–97, doi:10.46325/gabj.v8i1.385.
200. Checchi, V.; Montevecchi, M.; Valeriani, L.; Checchi, L. Bioburden Variation of Filtering Face Piece Respirators over Time: A Preliminary Study. *Materials* **2022**, *15*, 8790, doi:10.3390/ma15248790.
201. Chhakchhuak, Z.; Chhabra, M.C.; Panesar, S.; Duggal, N. Microbial Detection from Used Face Masks and Hygiene Practices. *Journal of Communicable Diseases (E-ISSN: 2581-351X & P-ISSN: 0019-5138)* **2023**, *55*, 111–118.
202. Delanghe, L.; Cauwenberghs, E.; Spacova, I.; De Boeck, I.; Van Beeck, W.; Pepermans, K.; Claes, I.; Vandenheuvel, D.; Verhoeven, V.; Lebeer, S. Cotton and Surgical Face Masks in Community Settings: Bacterial Contamination and Face Mask Hygiene. *Front Med (Lausanne)* **2021**, *8*, 732047, doi:10.3389/fmed.2021.732047.
203. Gund, M.P.; Naim, J.; Hannig, M.; Halfmann, A.; Gärtner, B.; Boros, G.; Rupf, S. CHX and a Face Shield Cannot Prevent Contamination of Surgical Masks. *Front Med (Lausanne)* **2022**, *9*, 896308, doi:10.3389/fmed.2022.896308.
204. Keri, V.C.; Kumar, A.; Singh, G.; Mandal, A.; Ali, H.; Ranjan, P.; Wig, N. Pilot Study on Burden of Fungal Contamination in Face Masks: Need for Better Mask Hygiene in the COVID-19 Era. *Infez Med* **2021**, *29*, 557–561, doi:10.53854/liim-2904-8.
205. Liu, Z.; Chang, Y.; Chu, W.; Yan, M.; Mao, Y.; Zhu, Z.; Wu, H.; Jie, Z.; Dai, K.; Li, H.; et al. Surgical Masks as Source of Bacterial Contamination during Operative Procedures. *Journal of Orthopaedic Translation* **2018**, *14*, 57–62, doi:10.1016/j.jot.2018.06.002.
206. Luksamijarulkul, P.; Aiempradit, N.; Vatanasomboon, P. Microbial Contamination on Used Surgical Masks among Hospital Personnel and Microbial Air Quality in Their Working Wards: A Hospital in Bangkok. *Oman Med J* **2014**, *29*, 346–350, doi:10.5001/omj.2014.92.
207. Merad, Y.; Belmokhtar, Z.; Hadjazi, O.; Belkacemi, M.; Matmour, D.; Merad, Z.; Bassaid, A.; Megherbi, O. Fungal Contamination of Medical Masks among Forensic Healthcare Workers in the COVID19 Era. *New Microbes and New Infections* **2023**, *53*, 101134, doi:10.1016/j.nmni.2023.101134.
208. Monalisa, D.; Aruna, C.N.; Padma, K.B.; Manjunath, K.; Hemavathy, E.; Varsha, D. Microbial Contamination of the Mouth Masks Used By Post- Graduate Students in a Private Dental Institution: An In-Vitro Study. *Journal of Dental and Medical Sciences* **2017**, *16*, 61–67.
209. Nightingale, M.; Mody, M.; Rickard, A.H.; Cassone, M. Bacterial Contamination on Used Face Masks among Nursing Home Healthcare Personnel. *Antimicrob Steward Healthc Epidemiol* **2023**, *3*, e54, doi:10.1017/ash.2023.130.
210. Park, A.-M.; Khadka, S.; Sato, F.; Omura, S.; Fujita, M.; Hashiwaki, K.; Tsunoda, I. Bacterial and Fungal Isolation from Face Masks under the COVID-19 Pandemic. *Sci Rep* **2022**,

12, 11361, doi:10.1038/s41598-022-15409-x.
211. Sachdev, R.; Garg, K.; Singh, G.; Mehrotra, V. Is Safeguard Compromised? Surgical Mouth Mask Harboring Hazardous Microorganisms in Dental Practice. *J Family Med Prim Care* **2020**, *9*, 759–763, doi:10.4103/jfmpc.jfmpc_1039_19.
212. Yang, Q.; Li, H.; Shen, S.; Zhang, G.; Huang, R.; Feng, Y.; Yang, J.; Ma, S. Study of the Micro-Climate and Bacterial Distribution in the Deadspace of N95 Filtering Face Respirators. *Sci Rep* **2018**, *8*, 17382, doi:10.1038/s41598-018-35693-w.
213. Yousefimashouf, M.; Yousefimashouf, R.; Alikhani, M.S.; Hashemi, H.; Karami, P.; Rahimi, Z.; Hosseini, S.M. Evaluation of the Bacterial Contamination of Face Masks Worn by Personnel in a Center of COVID 19 Hospitalized Patients: A Cross-Sectional Study. *New Microbes New Infect* **2023**, *52*, 101090, doi:10.1016/j.nmni.2023.101090.
214. VDI 6022 Available online: https://www.vdi.de/richtlinien/unsere-richtlinien-highlights/vdi-6022 (accessed on 14 October 2023).
215. Gund, M.P.; Boros, G.; Hannig, M.; Thieme-Ruffing, S.; Gärtner, B.; Rohrer, T.R.; Simon, A.; Rupf, S. Bacterial Contamination of Forehead Skin and Surgical Mask in Aerosol-Producing Dental Treatment. *J Oral Microbiol* **2021**, *13*, 1978731, doi:10.1080/20002297.2021.1978731.
216. Lee, Y.-H.; Kim, H.; Heo, D.W.; Ahn, I.-S.; Park, H.-K. Oral Microbiome of the Inner Surface of Face Masks and Whole Saliva during the COVID-19 Pandemic. *Frontiers in Oral Health* **2023**, *4*.
217. Szostak-Kotowa, J. Biodeterioration of Textiles. *International Biodeterioration & Biodegradation* **2004**, *53*, 165–170, doi:10.1016/S0964-8305(03)00090-8.
218. Szunerits, S.; Dörfler, H.; Pagneux, Q.; Daniel, J.; Wadekar, S.; Woitrain, E.; Ladage, D.; Montaigne, D.; Boukherroub, R. Exhaled Breath Condensate as Bioanalyte: From Collection Considerations to Biomarker Sensing. *Anal Bioanal Chem* **2023**, *415*, 27–34, doi:10.1007/s00216-022-04433-5.
219. Xiang, G.; Xu, K.; Jian, Y.; He, L.; Shen, Z.; Li, M.; Liu, Q. Prolonged Mask Wearing Changed Nasal Microbial Characterization of Young Adults during the COVID-19 Pandemic in Shanghai, China. *Frontiers in Immunology* **2023**, *14*.
220. Ahmad, E.F.E.M.; Mohammed, M.; Al Rayes, A.A.; Al Qahtani, A.; Elzubier, A.G.; Suliman, F.A.E. The Effect of Wearing the Veil by Saudi Ladies on the Occurrence of Respiratory Diseases. *Journal of Asthma* **2001**, *38*, 423–426, doi:10.1081/JAS-100001497.
221. Fögen, Z. The Foegen Effect: A Mechanism by Which Facemasks Contribute to the COVID-19 Case Fatality Rate. *Medicine (Baltimore)* **2022**, *101*, e28924, doi:10.1097/MD.0000000000028924.
222. Burgos-Blasco, B.; Arriola-Villalobos, P.; Fernandez-Vigo, J.I.; Oribio-Quinto, C.; Ariño-Gutierrez, M.; Diaz-Valle, D.; Benitez-del-Castillo, J.M. Face Mask Use and Effects on the Ocular Surface Health: A Comprehensive Review. *The Ocular Surface* **2023**, *27*, 56–66, doi:10.1016/j.jtos.2022.12.006.
223. Drewnick, F.; Pikmann, J.; Fachinger, F.; Moormann, L.; Sprang, F.; Borrmann, S. Aerosol Filtration Efficiency of Household Materials for Homemade Face Masks: Influence of Material Properties, Particle Size, Particle Electrical Charge, Face Velocity, and Leaks. *Aerosol Science and Technology* **2021**, *55*, 63–79, doi:10.1080/02786826.2020.1817846.
224. D'Souza, S.; Vaidya, T.; Nair, A.P.; Shetty, R.; Kumar, N.R.; Bisht, A.; Panigrahi, T.; J, T.S.; Khamar, P.; Dickman, M.M.; et al. Altered Ocular Surface Health Status and Tear Film Immune Profile Due to Prolonged Daily Mask Wear in Health Care Workers. *Biomedicines* **2022**, *10*, 1160, doi:10.3390/biomedicines10051160.
225. Huber, C. Masks, False Safety and Real Dangers, Part 4: Proposed Mechanisms by Which Masks Increase Risk of COVID-19. *Primary Doctor Medical Journal* **2020**, *1*, 1–9.
226. Mastropasqua, L.; Lanzini, M.; Brescia, L.; D'Aloisio, R.; Nubile, M.; Ciancaglini, M.; D'Amario, C.; Agnifili, L.; Mastropasqua, R. Face Mask-Related Ocular Surface Modifications

During COVID-19 Pandemic: A Clinical, In Vivo Confocal Microscopy, and Immune-Cytology Study. *Translational Vision Science & Technology* **2021**, *10*, 22, doi:10.1167/tvst.10.3.22.
227. Schultheis, W.G.; Sharpe, J.E.; Zhang, Q.; Patel, S.N.; Kuriyan, A.E.; Chiang, A.; Garg, S.J.; Hsu, J. Effect of Taping Face Masks on Quantitative Particle Counts Near the Eye: Implications for Intravitreal Injections in the COVID-19 Era. *American Journal of Ophthalmology* **2021**, *225*, 166–171, doi:10.1016/j.ajo.2021.01.021.
228. Shah, Y.; Kurelek, J.W.; Peterson, S.D.; Yarusevych, S. Experimental Investigation of Indoor Aerosol Dispersion and Accumulation in the Context of COVID-19: Effects of Masks and Ventilation. *Physics of Fluids* **2021**, *33*, 073315, doi:10.1063/5.0057100.
229. Viola, I.M.; Peterson, B.; Pisetta, G.; Pavar, G.; Akhtar, H.; Menoloascina, F.; Mangano, E.; Dunn, K.E.; Gabl, R.; Nila, A.; et al. Face Coverings, Aerosol Dispersion and Mitigation of Virus Transmission Risk. *IEEE Open J Eng Med Biol* **2021**, *2*, 26–35, doi:10.1109/OJEMB.2021.3053215.
230. Akioud, W.; Sebbata, S.; Mozarie, Y.; Oubaaz, A. Chalazion and Face Mask Wear during COVID-19 Pandemic: Is There A Link? *European Journal of Medical and Health Sciences* **2023**, *5*, 17–19, doi:10.24018/ejmed.2023.5.2.1641.
231. Hadayer, A.; Zahavi, A.; Livny, E.; Gal-Or, O.; Gershoni, A.; Mimouni, K.; Ehrlich, R. PATIENTS WEARING FACE MASKS DURING INTRAVITREAL INJECTIONS MAY BE AT A HIGHER RISK OF ENDOPHTHALMITIS. *Retina* **2020**, *40*, 1651–1656, doi:10.1097/IAE.0000000000002919.
232. Molero-Senosiain, M.; Tiew, S.; Patel, A.; Houben, I.; Dhillon, N. Impact of Face Mask Wear on Bacterial Keratitis. *Journal Français d'Ophtalmologie* **2023**, *46*, e37–e39, doi:10.1016/j.jfo.2022.04.028.
233. Sakamoto, T.; Terasaki, H.; Yamashita, T.; Shiihara, H.; Funatsu, R.; Uemura, A. Increased Incidence of Endophthalmitis after Vitrectomy Relative to Face Mask Wearing during COVID-19 Pandemic. *British Journal of Ophthalmology* **2023**, *107*, 1472–1477, doi:10.1136/bjophthalmol-2022-321357.
234. Silkiss, R.Z.; Paap, M.K.; Ugradar, S. Increased Incidence of Chalazion Associated with Face Mask Wear during the COVID-19 Pandemic. *Am J Ophthalmol Case Rep* **2021**, *22*, 101032, doi:10.1016/j.ajoc.2021.101032.
235. Brooks, J.K.; Sultan, A.S.; Jabra-Rizk, M.A. Prolonged Facial Mask Wear Is a Concern for the Development of Dysbiotic Microbiome. *Respiratory Medicine and Research* **2022**, *81*, 100877, doi:10.1016/j.resmer.2021.100877.
236. Sakr, A.; Brégeon, F.; Mège, J.-L.; Rolain, J.-M.; Blin, O. Staphylococcus Aureus Nasal Colonization: An Update on Mechanisms, Epidemiology, Risk Factors, and Subsequent Infections. *Frontiers in Microbiology* **2018**, *9*.
237. Asadi, S.; Cappa, C.D.; Barreda, S.; Wexler, A.S.; Bouvier, N.M.; Ristenpart, W.D. Efficacy of Masks and Face Coverings in Controlling Outward Aerosol Particle Emission from Expiratory Activities. *Scientific Reports* **2020**, *10*, 15665, doi:10.1038/s41598-020-72798-7.
238. Bagchi, S.; Basu, S.; Chaudhuri, S.; Saha, A. Penetration and Secondary Atomization of Droplets Impacted on Wet Facemasks. *Phys. Rev. Fluids* **2021**, *6*, 110510, doi:10.1103/PhysRevFluids.6.110510.
239. Kasloff, S.B.; Leung, A.; Strong, J.E.; Funk, D.; Cutts, T. Stability of SARS-CoV-2 on Critical Personal Protective Equipment. *Sci Rep* **2021**, *11*, 984, doi:10.1038/s41598-020-80098-3.
240. Córdoba-Lanús, E.; García-Pérez, O.; Cazorla-Rivero, S.; Rodríguez-Esparragón, F.; Piñero, J.-E.; Clavo, B.; Lorenzo-Morales, J. Persistence of SARS-CoV-2 Infection on Personal Protective Equipment (PPE). *BMC Infect Dis* **2021**, *21*, 1169, doi:10.1186/s12879-021-06861-7.
241. WMA - The World Medical Association-WMA Declaration of Geneva 2018.
242. Goldsmith, D.J.A.; Orlowski, E.J.W. Don't Forget About the Children - A Narrative Review of How COVID-19 Pandemic Policy in the UK and Sweden Impacted Children's Wellbeing.

Medical Research Archives **2023**, *11*, doi:10.18103/mra.v11i11.4652.
243. Shobako, N. Lessons from the Health Policies for Children during the Pandemic in Japan. *Frontiers in Public Health* **2022**, *10*.
244. Sezer, H.; Çınar, D.; Kılıç Akça, N. The Effect of Prolonged Use of Surgical Masks during Face-to-Face Teaching on Cognitive and Physiological Parameters of Nursing Students: A Cross-Sectional and Descriptive Study. *Nurse Education in Practice* **2023**, *72*, 103779, doi:10.1016/j.nepr.2023.103779.
245. Krishnaratne, S.; Pfadenhauer, L.M.; Coenen, M.; Geffert, K.; Jung-Sievers, C.; Klinger, C.; Kratzer, S.; Littlecott, H.; Movsisyan, A.; Rabe, J.E.; et al. Measures Implemented in the School Setting to Contain the COVID-19 Pandemic: A Rapid Scoping Review - Krishnaratne, S - 2020 | Cochrane Library.
246. Cao, S.; Gan, Y.; Wang, C.; Bachmann, M.; Wei, S.; Gong, J.; Huang, Y.; Wang, T.; Li, L.; Lu, K.; et al. Post-Lockdown SARS-CoV-2 Nucleic Acid Screening in Nearly Ten Million Residents of Wuhan, China. *Nat Commun* **2020**, *11*, 5917, doi:10.1038/s41467-020-19802-w.
247. Wei, W.E.; Li, Z.; Chiew, C.J.; Yong, S.E.; Toh, M.P.; Lee, V.J. Presymptomatic Transmission of SARS-CoV-2 - Singapore, January 23-March 16, 2020. *MMWR Morb Mortal Wkly Rep* **2020**, *69*, 411–415, doi:10.15585/mmwr.mm6914e1.
248. Elgersma, I.H.; Fretheim, A.; Elstrøm, P.; Aavitsland, P. Association between Face Mask Use and Risk of SARS-CoV-2 Infection: Cross-Sectional Study. *Epidemiol Infect* **2023**, *151*, e194, doi:10.1017/S0950268823001826.
249. Spira, B. Correlation Between Mask Compliance and COVID-19 Outcomes in Europe. *Cureus* **2022**, *14*, e24268, doi:10.7759/cureus.24268.
250. Cheng, Y.; Ma, N.; Witt, C.; Rapp, S.; Wild, P.S.; Andreae, M.O.; Pöschl, U.; Su, H. Face Masks Effectively Limit the Probability of SARS-CoV-2 Transmission. *Science* **2021**, *372*, 1439–1443, doi:10.1126/science.abg6296.
251. Leech, G.; Rogers-Smith, C.; Monrad, J.T.; Sandbrink, J.B.; Snodin, B.; Zinkov, R.; Rader, B.; Brownstein, J.S.; Gal, Y.; Bhatt, S.; et al. Mask Wearing in Community Settings Reduces SARS-CoV-2 Transmission. *Proc Natl Acad Sci U S A* **2022**, *119*, e2119266119, doi:10.1073/pnas.2119266119.
252. Ueki, H.; Furusawa, Y.; Iwatsuki-Horimoto, K.; Imai, M.; Kabata, H.; Nishimura, H.; Kawaoka, Y. Effectiveness of Face Masks in Preventing Airborne Transmission of SARS-CoV-2. *mSphere* **2020**, *5*, doi:10.1128/mSphere.00637-20.
253. Knobloch, J.K.; Franke, G.; Knobloch, M.J.; Knobling, B.; Kampf, G. Overview of Tight Fit and Infection Prevention Benefits of Respirators (Filtering Face Pieces). *Journal of Hospital Infection* **2023**, *134*, 89–96, doi:10.1016/j.jhin.2023.01.009.
254. Gralton, J.; McLaws, M.-L. Protecting Healthcare Workers from Pandemic Influenza: N95 or Surgical Masks? *Crit Care Med* **2010**, *38*, 657–667, doi:10.1097/ccm.0b013e3181b9e8b3.
255. Machida, M.; Nakamura, I.; Saito, R.; Nakaya, T.; Hanibuchi, T.; Takamiya, T.; Odagiri, Y.; Fukushima, N.; Kikuchi, H.; Amagasa, S.; et al. Incorrect Use of Face Masks during the Current COVID-19 Pandemic among the General Public in Japan. *Int J Environ Res Public Health* **2020**, *17*, 6484, doi:10.3390/ijerph17186484.
256. Mickells, G.E.; Figueroa, J.; West, K.W.; Wood, A.; McElhanon, B.O. Adherence to Masking Requirement During the COVID-19 Pandemic by Early Elementary School Children. *J Sch Health* **2021**, *91*, 555–561, doi:10.1111/josh.13033.
257. Xiang Ong, S.W.; Tang, Y.W.; Linn, K.Z.; Huan, X.W.; Lim, A.; Poon, C.Y.; Ru Tan, D.H.; Binte Hamed, N.H.; Syed Husen, S.F.B.; Hui Ong, C.C.; et al. Compliance with Face Mask Use during the COVID-19 Pandemic: A Community Observational Study in Singapore. *Singapore Med J* **2023**, doi:10.4103/singaporemedj.SMJ-2021-010.
258. Luckman, A.; Zeitoun, H.; Isoni, A.; Loomes, G.; Vlaev, I.; Powdthavee, N.; Read, D. *Risk Compensation during COVID-19: The Impact of Face Mask Usage on Social Distancing.*; OSF Preprints, 2020;

259. Szczesniak, D.; Ciulkowicz, M.; Maciaszek, J.; Misiak, B.; Luc, D.; Wieczorek, T.; Witecka, K.-F.; Rymaszewska, J. Psychopathological Responses and Face Mask Restrictions during the COVID-19 Outbreak: Results from a Nationwide Survey. *Brain Behav Immun* **2020**, *87*, 161–162, doi:10.1016/j.bbi.2020.05.027.
260. Sharma, I.; Vashnav, M.; Sharma, R. COVID-19 Pandemic Hype: Losers and Gainers. *Indian Journal of Psychiatry* **2020**, *62*, 420, doi:10.4103/psychiatry.IndianJPsychiatry_1060_20.
261. Lee, S.-A.; Grinshpun, S.A.; Reponen, T. Respiratory Performance Offered by N95 Respirators and Surgical Masks: Human Subject Evaluation with NaCl Aerosol Representing Bacterial and Viral Particle Size Range. *Ann Occup Hyg* **2008**, *52*, 177–185, doi:10.1093/annhyg/men005.
262. Barari, K.; Si, X.; Xi, J. Impacts of Mask Wearing and Leakages on Cyclic Respiratory Flows and Facial Thermoregulation. *Fluids* **2024**, *9*, 9, doi:10.3390/fluids9010009.
263. Jia, Z.; Ai, Z.; Cao, S.; Bekö, G. Effectiveness of Respiratory Protective Equipment on Source Control of Exhaled Pollutants. *Journal of Building Engineering* **2024**, *86*, 108742, doi:10.1016/j.jobe.2024.108742.
264. Zhu, Y.; Xia, Y.; Pickering, J.; Bowen, A.C.; Short, K.R. The Role of Children in Transmission of SARS-CoV-2 Variants of Concern within Households: An Updated Systematic Review and Meta-Analysis, as at 30 June 2022. *Euro Surveill* **2023**, *28*, 2200624, doi:10.2807/1560-7917.ES.2023.28.18.2200624.
265. Felsenstein, S.; Hedrich, C.M. SARS-CoV-2 Infections in Children and Young People. *Clin Immunol* **2020**, *220*, 108588, doi:10.1016/j.clim.2020.108588. https://doi.org/10.1016/j.clim.2020.108588
266. Fikenzer, S.; Uhe, T.; Lavall, D.; Rudolph, U.; Falz, R.; Busse, M.; Hepp, P.; Laufs, U. Effects of Surgical and FFP2/N95 Face Masks on Cardiopulmonary Exercise Capacity. *Clin Res Cardiol* **2020**, 1–9, doi:10.1007/s00392-020-01704-y.
267. Lee, H.P.; Wang, D.Y. Objective Assessment of Increase in Breathing Resistance of N95 Respirators on Human Subjects. *Ann Occup Hyg* **2011**, *55*, 917–921, doi:10.1093/annhyg/mer065.
268. Li, Y.; Tokura, H.; Guo, Y.P.; Wong, A.S.W.; Wong, T.; Chung, J.; Newton, E. Effects of Wearing N95 and Surgical Facemasks on Heart Rate, Thermal Stress and Subjective Sensations. *Int Arch Occup Environ Health* **2005**, *78*, 501–509, doi:10.1007/s00420-004-0584-4.
269. Mapelli, M.; Salvioni, E.; Martino, F.D.; Mattavelli, I.; Gugliandolo, P.; Vignati, C.; Farina, S.; Palermo, P.; Campodonico, J.; Maragna, R.; et al. “You Can Leave Your Mask on”: Effects on Cardiopulmonary Parameters of Different Airway Protection Masks at Rest and during Maximal Exercise. *European Respiratory Journal* **2021**, doi:10.1183/13993003.04473-2020.
270. Roberge, R.J.; Bayer, E.; Powell, J.B.; Coca, A.; Roberge, M.R.; Benson, S.M. Effect of Exhaled Moisture on Breathing Resistance of N95 Filtering Facepiece Respirators. *Ann Occup Hyg* **2010**, *54*, 671–677, doi:10.1093/annhyg/meq042.
271. Roberge, R.J.; Coca, A.; Williams, W.J.; Powell, J.B.; Palmiero, A.J. Physiological Impact of the N95 Filtering Facepiece Respirator on Healthcare Workers. *Respir Care* **2010**, *55*, 569–577.
272. Roberge, R.; Benson, S.; Kim, J.-H. Thermal Burden of N95 Filtering Facepiece Respirators. *Ann Occup Hyg* **2012**, *56*, 808–814, doi:10.1093/annhyg/mes001.
273. Roberge, R.J.; Kim, J.-H.; Benson, S.M. Absence of Consequential Changes in Physiological, Thermal and Subjective Responses from Wearing a Surgical Mask. *Respiratory Physiology & Neurobiology* **2012**, *181*, 29–35, doi:10.1016/j.resp.2012.01.010.
274. de Yzaguirre i Maura, I.; Zabala, D.D.; Brotons i Cuixart, D.; Gutierrez Rincon, J.A.; Vives i Turcó, J.; Grazioli, G. Physiological Impact of Different Types of Mask at Rest. *Apunts Sports Medicine* **2022**, *57*, 100389, doi:10.1016/j.apunsm.2022.100389.
275. Elbl, C.; Brunner, J.X.; Schier, D.; Junge, A.; Junge, H. Protective Face Masks Add Significant Dead Space. *European Respiratory Journal* **2021**, *58*, doi:10.1183/13993003.01131-

2021.
276. Epstein, D.; Korytny, A.; Isenberg, Y.; Marcusohn, E.; Zukermann, R.; Bishop, B.; Minha, S.; Raz, A.; Miller, A. Return to Training in the COVID-19 Era: The Physiological Effects of Face Masks during Exercise. *Scandinavian Journal of Medicine & Science in Sports* **2020**, *n/a*, doi:10.1111/sms.13832.
277. Johnson, A.T.; Scott, W.H.; Lausted, C.G.; Coyne, K.M.; Sahota, M.S.; Johnson, M.M. Effect of External Dead Volume on Performance While Wearing a Respirator. *AIHAJ - American Industrial Hygiene Association* **2000**, *61*, 678–684, doi:10.1080/15298660008984577.
278. Ngo, H.; Spaeth, J.; Schumann, S. Effective Volume of Rebreathed Air during Breathing with Facepieces Increases with Protection Class and Decreases with Ambient Airflow. *PLOS ONE* **2024**, *19*, e0299919, doi:10.1371/journal.pone.0299919.
279. Roberge, R.J.; Kim, J.-H.; Powell, J.B. N95 Respirator Use during Advanced Pregnancy. *Am J Infect Control* **2014**, *42*, 1097–1100, doi:10.1016/j.ajic.2014.06.025.
280. Shui, L.; Yang, B.; Tang, H.; Luo, Y.; Hu, S.; Zhong, X.; Duan, J. Physiological Effects of Surgical and N95 Masks During Exercise in the COVID-19 Era. *The American Journal of the Medical Sciences* **2022**, *363*, 411–419, doi:10.1016/j.amjms.2022.02.006.
281. Xu, M.; Lei, Z.; Yang, J. Estimating the Dead Space Volume Between a Headform and N95 Filtering Facepiece Respirator Using Microsoft Kinect. *Journal of Occupational and Environmental Hygiene* **2015**, *12*, 538–546, doi:10.1080/15459624.2015.1019078.
282. Arce, S.C.; Chiodetti, F.; De Vito, E.L. Dead Space Volume in N95 Masks. *Arch Bronconeumol* **2021**, *57*, 434, doi:10.1016/j.arbr.2020.11.016.
283. Butz, U. Rückatmung von Kohlendioxid bei Verwendung von Operationsmasken als hygienischer Mundschutz an medizinischem Fachpersonal, Universitätsbibliothek der Technischen Universität München, 2005.
284. Laferty, E.A.; McKay, R.T. Physiologic Effects and Measurement of Carbon Dioxide and Oxygen Levels during Qualitative Respirator Fit Testing. *J. Chem. Health Saf.* **2006**, *13*, 22–28, doi:10.1016/j.jchas.2005.11.015.
285. Pifarré, F.; Zabala, D.D.; Grazioli, G.; de Yzaguirre i Maura, I. COVID 19 and Mask in Sports. *Apunts Sports Medicine* **2020**, doi:10.1016/j.apunsm.2020.06.002.
286. Rhee, M.S.M.; Lindquist, C.D.; Silvestrini, M.T.; Chan, A.C.; Ong, J.J.Y.; Sharma, V.K. Carbon Dioxide Increases with Face Masks but Remains below Short-Term NIOSH Limits. *BMC Infect Dis* **2021**, *21*, 354, doi:10.1186/s12879-021-06056-0.
287. Salati, H.; Khamooshi, M.; Vahaji, S.; Christo, F.C.; Fletcher, D.F.; Inthavong, K. N95 Respirator Mask Breathing Leads to Excessive Carbon Dioxide Inhalation and Reduced Heat Transfer in a Human Nasal Cavity. *Phys Fluids (1994)* **2021**, *33*, 081913, doi:10.1063/5.0061574.
288. Sinkule, E.J.; Powell, J.B.; Goss, F.L. Evaluation of N95 Respirator Use with a Surgical Mask Cover: Effects on Breathing Resistance and Inhaled Carbon Dioxide. *Ann Occup Hyg* **2013**, *57*, 384–398, doi:10.1093/annhyg/mes068.
289. Zhang, X.; Li, H.; Shen, S.; Cai, M. Investigation of the Flow-Field in the Upper Respiratory System When Wearing N95 Filtering Facepiece Respirator. *J Occup Environ Hyg* **2016**, *13*, 372–382, doi:10.1080/15459624.2015.1116697.
290. Zhang, G.; Li, M.; Zheng, M.; Cai, X.; Yang, J.; Zhang, S.; Yilifate, A.; Zheng, Y.; Lin, Q.; Liang, J.; et al. Effect of Surgical Masks on Cardiopulmonary Function in Healthy Young Subjects: A Crossover Study. *Front Physiol* **2021**, *12*, 710573, doi:10.3389/fphys.2021.710573.
291. Akhondi, H.; Kaveh, S.; Kaufman, K.; Danai, T.; Ayutyanont, N. CO2 Levels Behind and in Front of Different Protective Mask Types. *HCA Healthcare Journal of Medicine* **2022**, *3*, doi:10.36518/2689-0216.1321.
292. Balkir, B.; Lankar, V.; Hangun, Y. Comparison of Face Masks by Arduino Sensors. *Cornell Undergraduate Research Journal* **2023**, *2*, 63–73, doi:10.37513/curj.v2i2.733.

293. Bar-On, O.; Goldberg, O.; Stafler, P.; Levine, H.; Jacobi, E.; Shmueli, E.; Rothschild, B.; Prais, D.; Mei-Zahav, M. Wearing Face Masks While Climbing Stairs Influences Respiratory Physiology. *J. Breath Res.* **2024**, doi:10.1088/1752-7163/ad3fde.
294. Bharatendu, C.; Ong, J.J.Y.; Goh, Y.; Tan, B.Y.Q.; Chan, A.C.Y.; Tang, J.Z.Y.; Leow, A.S.; Chin, A.; Sooi, K.W.X.; Tan, Y.L.; et al. Powered Air Purifying Respirator (PAPR) Restores the N95 Face Mask Induced Cerebral Hemodynamic Alterations among Healthcare Workers during COVID-19 Outbreak. *J Neurol Sci* **2020**, *417*, 117078, doi:10.1016/j.jns.2020.117078.
295. Decha, C.; Sonthaya, S. A Long-Term Effects of Wearing a Reusable Mask While Running Exercises on Blood Gas Levels in Adults. *Journal of Physical Education and Sport* **2023**, 255–263.
296. Dirol, H.; Alkan, E.; Sindel, M.; Ozdemir, T.; Erbas, D. The Physiological and Disturbing Effects of Surgical Face Masks in the COVID-19 Era. *BLL* **2021**, *122*, 821–825, doi:10.4149/BLL_2021_131.
297. Engeroff, T.; Heinsel, K.; Niederer, D.; Nienhaus, A.; Groneberg, D.A.; Vogt, L. Investigating Effects of FFP2 Wearing during Physical Activity on Gas Exchange, Metabolism and Affective State Using a Randomized Controlled Trial. *Sci Rep* **2024**, *14*, 6278, doi:10.1038/s41598-024-56560-x.
298. Fantin, R. The Effect of Wearing an FFP3 Mask (3M TM Aura TM) with an Exhalation Valve on Gas Exchange in Medical Staff. *Int J Occup Med Environ Health* **2021**, doi:10.13075/ijomeh.1896.01809.
299. Georgi, C.; Haase-Fielitz, A.; Meretz, D.; Gäsert, L.; Butter, C. The Impact of Commonly-Worn Face Masks on Physiological Parameters and on Discomfort During Standard Work-Related Physical Effort. *Dtsch Arztebl Int* **2020**, *117*, 674–675, doi:10.3238/arztebl.2020.0674.
300. Kim, J.-H.; Benson, S.M.; Roberge, R.J. Pulmonary and Heart Rate Responses to Wearing N95 Filtering Facepiece Respirators. *Am J Infect Control* **2013**, *41*, 24–27, doi:10.1016/j.ajic.2012.02.037.
301. Kim, J.-H.; Roberge, R.J.; Powell, J.B. Effect of External Airflow Resistive Load on Postural and Exercise-Associated Cardiovascular and Pulmonary Responses in Pregnancy: A Case Control Study. *BMC Pregnancy and Childbirth* **2015**, *15*, 45, doi:10.1186/s12884-015-0474-7.
302. Marek, E.-M.; van Kampen, V.; Jettkant, B.; Kendzia, B.; Strauß, B.; Sucker, K.; Ulbrich, M.; Deckert, A.; Berresheim, H.; Eisenhawer, C.; et al. Effects of Wearing Different Face Masks on Cardiopulmonary Performance at Rest and Exercise in a Partially Double-Blinded Randomized Cross-over Study. *Sci Rep* **2023**, *13*, 6950, doi:10.1038/s41598-023-32180-9.
303. Mo, Y. Risk and Impact of Using Mask on COPD Patients with Acute Exacerbation during the COVID-19 Outbreak: A Retrospective Study. **2020**, doi:10.21203/rs.3.rs-39747/v1.
304. Patel, S.; Mohapatra, E.; Suganthy, A.K.; Shah, S.; Abraham, J.; Nanda, R.; Behera, A.K.; Gupta, A. A Pilot Study to Evaluate the Changes in Venous Blood Gas Parameters and Hypoxia Biomarkers in Health Care Workers Using Different Kinds of Masks. *Lung India* **2023**, *40*, 134–142, doi:10.4103/lungindia.lungindia_343_22.
305. Shechtman, L.; Ben-Haim, G.; Ben-Zvi, I.; Steel, L.; Ironi, A.; Huszti, E.; Chatterji, S.; Levy, L. Physiological Effects of Wearing N95 Respirator on Medical Staff During Prolong Work Hours in Covid-19 Departments. *J Occup Environ Med* **2022**, *64*, e378–e380, doi:10.1097/JOM.0000000000002542.
306. Sofonova, D.; Angelova, R.; Sofronov Measuring the Carbon Dioxide Concentration under Protective Face Masks with a Sensor System. *Applied Ecology and environmental research* **2023**, *21(4)*, 2775–2792, doi:: http://dx.doi.org/10.15666/aeer/2104_27752792.
307. Tong, P.S.Y.; Kale, A.S.; Ng, K.; Loke, A.P.; Choolani, M.A.; Lim, C.L.; Chan, Y.H.; Chong, Y.S.; Tambyah, P.A.; Yong, E.-L. Respiratory Consequences of N95-Type Mask Usage in Pregnant Healthcare Workers—a Controlled Clinical Study. *Antimicrobial Resistance & Infection Control* **2015**, *4*, 48, doi:10.1186/s13756-015-0086-z.

308. Vogt, G.; Radtke, K.; Jagim, A.; Peckumn, D.; Lee, T.; Mikat, R.; Foster, C. Effect of Face Masks on Physiological and Perceptual Responses during 30 Minutes of Self-Paced Exercise in Older Community Dwelling Adults. *International Journal of Environmental Research and Public Health* **2022**, *19*, 12877, doi:10.3390/ijerph191912877.
309. Wangsan, K.; Sapbamrer, R.; Sirikul, W.; Panumasvivat, J.; Surawattanasakul, V.; Assavanopakun, P. Effect of N95 Respirator on Oxygen and Carbon Dioxide Physiologic Response: A Systematic Review and Meta-Analysis. *International Journal of Environmental Research and Public Health* **2022**, *19*, 8646, doi:10.3390/ijerph19148646.
310. Kiray, M.; Sisman, A.R.; Camsari, U.M.; Evren, M.; Dayi, A.; Baykara, B.; Aksu, I.; Ates, M.; Uysal, N. Effects of Carbon Dioxide Exposure on Early Brain Development in Rats. *Biotechnic & Histochemistry* **2014**, *89*, 371–383, doi:10.3109/10520295.2013.872298.
311. Uysal, N.; Kiray, M.; Sisman, A.R.; Baykara, B.; Aksu, I.; Dayi, A.; Gencoglu, C.; Evren, M.; Buyuk, E.; Cetin, F.; et al. Effects of Exercise and Poor Indoor Air Quality on Learning, Memory and Blood IGF-1 in Adolescent Mice. *Biotechnic & Histochemistry* **2014**, *89*, 126–135, doi:10.3109/10520295.2013.825318.
312. Vandemark, N.L.; Schanbacher, B.D.; Gomes, W.R. Alterations in Testes of Rats Exposed to Elevated Atmospheric Carbon Dioxide. *J Reprod Fertil* **1972**, *28*, 457–459, doi:10.1530/jrf.0.0280457.
313. *Evaluation of the Health Aspects of Carbon Dioxide as a Food Ingredient.*; Federation of American Societies for Experimental Biology, Bethesda, MD. Life Sciences Research Office.; Food and Drug Administration, Washington, DC. Bureau of Foods., 1979;
314. Howard, W.R.; Wong, B.; Yeager, K.S.B.; Stump, D.G.; Edwards, T.; Arden James, R.; Goodwin, M.R.; Gargas, M.L. Submarine Exposure Guideline Recommendations for Carbon Dioxide Based on the Prenatal Developmental Effects of Exposure in Rats. *Birth Defects Res* **2019**, *111*, 26–33, doi:10.1002/bdr2.1417.
315. Howard, W.R.; Wong, B.; Okolica, M.; Bynum, K.S.; James, R.A. *The Prenatal Development Effects of Carbon Dioxide (CO2) Exposure in Rats (Rattus Norvegicus):*; Defense Technical Information Center: Fort Belvoir, VA, 2012;
316. Casalino-Matsuda, S.M.; Wang, N.; Ruhoff, P.T.; Matsuda, H.; Nlend, M.C.; Nair, A.; Szleifer, I.; Beitel, G.J.; Sznajder, J.I.; Sporn, P.H.S. Hypercapnia Alters Expression of Immune Response, Nucleosome Assembly and Lipid Metabolism Genes in Differentiated Human Bronchial Epithelial Cells. *Sci Rep* **2018**, *8*, 13508, doi:10.1038/s41598-018-32008-x.
317. Sikter, A.; Faludi, G.; Rihmer, Z. The Role of Carbon Dioxide (and Intracellular pH) in the Pathomechanism of Several Mental Disorders. Are the Diseases of Civilization Caused by Learnt Behaviour, Not the Stress Itself? *Neuropsychopharmacol Hung* **2009**, *11*, 161–173.
318. Hoffman, W.E.; Charbel, F.T.; Edelman, G.; Ausman, J.I. Brain Tissue Acid-Base Response to Hypercapnia in Neurosurgical Patients. *Neurol Res* **1995**, *17*, 417–420.
319. Huo, X.; Min, J.; Pan, C.; Zhao, C.; Pan, L.; Gui, F.; Jin, L.; Wang, X. Efficacy of Lovastatin on Learning and Memory Deficits Caused by Chronic Intermittent Hypoxia-Hypercapnia: Through Regulation of NR2B-Containing NMDA Receptor-ERK Pathway. *PLOS ONE* **2014**, *9*, e94278, doi:10.1371/journal.pone.0094278.
320. Dodge, F.A.; Rahamimoff, R. Co-Operative Action a Calcium Ions in Transmitter Release at the Neuromuscular Junction. *J Physiol* **1967**, *193*, 419–432, doi:10.1113/jphysiol.1967.sp008367.
321. Tombaugh, G.C.; Somjen, G.G. Differential Sensitivity to Intracellular pH among High- and Low-Threshold Ca2+ Currents in Isolated Rat CA1 Neurons. *J Neurophysiol* **1997**, *77*, 639–653, doi:10.1152/jn.1997.77.2.639.
322. Hota, K.B.; Hota, S.K.; Chaurasia, O.P.; Singh, S.B. Acetyl-L-Carnitine-Mediated Neuroprotection during Hypoxia Is Attributed to ERK1/2-Nrf2-Regulated Mitochondrial Biosynthesis. *Hippocampus* **2012**, *22*, 723–736, doi:10.1002/hipo.20934.

323. Benjamin Ezraty; Maïalène Chabalier; Adrien Ducret; Etienne Maisonneuve; Sam Dukan CO2 Exacerbates Oxygen Toxicity. *EMBO reports* **2011**, *12*, 321–326, doi:10.1038/embor.2011.7.
324. Guais, A.; Brand, G.; Jacquot, L.; Karrer, M.; Dukan, S.; Grévillot, G.; Molina, T.J.; Bonte, J.; Regnier, M.; Schwartz, L. Toxicity of Carbon Dioxide: A Review. *Chem. Res. Toxicol.* **2011**, *24*, 2061–2070, doi:10.1021/tx200220r.
325. Jacobson, T.A.; Kler, J.S.; Hernke, M.T.; Braun, R.K.; Meyer, K.C.; Funk, W.E. Direct Human Health Risks of Increased Atmospheric Carbon Dioxide. *Nat Sustain* **2019**, *2*, 691–701, doi:10.1038/s41893-019-0323-1.
326. Thom, S.R.; Bhopale, V.M.; Hu, J.; Yang, M. Inflammatory Responses to Acute Elevations of Carbon Dioxide in Mice. *Journal of Applied Physiology* **2017**, *123*, 297–302, doi:10.1152/japplphysiol.00343.2017.
327. Beheshti, A.; Cekanaviciute, E.; Smith, D.J.; Costes, S.V. Global Transcriptomic Analysis Suggests Carbon Dioxide as an Environmental Stressor in Spaceflight: A Systems Biology GeneLab Case Study. *Sci Rep* **2018**, *8*, 4191, doi:10.1038/s41598-018-22613-1.
328. Zappulla, D. Environmental Stress, Erythrocyte Dysfunctions, Inflammation, and the Metabolic Syndrome: Adaptations to CO2 Increases? *Journal of the CardioMetabolic Syndrome* **2008**, *3*, 30–34, doi:10.1111/j.1559-4572.2008.07263.x.
329. Bushinsky, D.A.; Krieger, N.S. Effects of Acid on Bone. *Kidney Int* **2022**, *101*, 1160–1170, doi:10.1016/j.kint.2022.02.032.
330. Adeva-Andany, M.M.; Carneiro-Freire, N.; Donapetry-García, C.; Rañal-Muíño, E.; López-Pereiro, Y. The Importance of the Ionic Product for Water to Understand the Physiology of the Acid-Base Balance in Humans. *BioMed Research International* **2014**, *2014*, e695281, doi:10.1155/2014/695281.
331. Adeva-Andany, M.M.; Fernández-Fernández, C.; Sánchez-Bello, R.; Donapetry-García, C.; Martínez-Rodríguez, J. The Role of Carbonic Anhydrase in the Pathogenesis of Vascular Calcification in Humans. *Atherosclerosis* **2015**, *241*, 183–191, doi:10.1016/j.atherosclerosis.2015.05.012.
332. Tan, S.-I.; Han, Y.-L.; Yu, Y.-J.; Chiu, C.-Y.; Chang, Y.-K.; Ouyang, S.; Fan, K.-C.; Lo, K.-H.; Ng, I.-S. Efficient Carbon Dioxide Sequestration by Using Recombinant Carbonic Anhydrase. *Process Biochemistry* **2018**, *73*, 38–46, doi:10.1016/j.procbio.2018.08.017.
333. Kim, I.G.; Jo, B.H.; Kang, D.G.; Kim, C.S.; Choi, Y.S.; Cha, H.J. Biomineralization-Based Conversion of Carbon Dioxide to Calcium Carbonate Using Recombinant Carbonic Anhydrase. *Chemosphere* **2012**, *87*, 1091–1096, doi:10.1016/j.chemosphere.2012.02.003.
334. Schaefer, K.E.; Douglas, W.H.; Messier, A.A.; Shea, M.L.; Gohman, P.A. Effect of Prolonged Exposure to 0.5% CO2 on Kidney Calcification and Ultrastructure of Lungs. *Undersea Biomed Res* **1979**, *6 Suppl*, S155-161.
335. Abdel Mowla Ahmed Abdel Mowla, H.; Hashem, E.S. Physiological Burdens of Prolonged Use of Surgical Face Masks among Healthcare Workers During the COVID-19 Pandemic Era. *Egyptian Journal of Nursing and Health Sciences* **2022**, *3*, 236–257, doi:10.21608/ejnhs.2022.261796.
336. Alroudhan, I.E.; Ganji, K.K.; Hamza, M.O.; Munisekhar, M.S.; Sghaireen, M.G.; Alam, M.K. Effect of N95 Filtering Facepiece Respirators on Dental Health Professionals with an Emphasis on Pulmonary Function and Heart Rate: An Intrasubject Comparison. *Br J Oral Maxillofac Surg* **2021**, *59*, 1302–1307, doi:10.1016/j.bjoms.2021.07.004.
337. Bayoumi, A.; Shawki, M.; Abdulaziz, A.; Allam, M.; Mosleh, M. Blood Oxygen Level with Long-Term Use of N95 Face Mask in Dental Practice during the Coronavirus Pandemic "Covid-19." *Advances in Medical, Pharmaceutical and Dental Research* **2022**, *2*, 015–021, doi:10.21622/ampdr.2022.02.1.015.
338. Beder, A.; Büyükkoçak, U.; Sabuncuoğlu, H.; Keskil, Z.A.; Keskil, S. Preliminary Report on Surgical Mask Induced Deoxygenation during Major Surgery. *Neurocirugia (Astur)* **2008**, *19*,

121–126, doi:10.1016/s1130-1473(08)70235-5.
339. Cabanillas-Barea, S.; Rodríguez-Sanz, J.; Carrasco-Uribarren, A.; López-de-Celis, C.; González-Rueda, V.; Zegarra-Chávez, D.; Cedeño-Bermúdez, S.; Pérez-Bellmunt, A. Effects of Using the Surgical Mask and FFP2 during the 6-Min Walking Test. A Randomized Controlled Trial. *Int J Environ Res Public Health* **2021**, *18*, 12420, doi:10.3390/ijerph182312420.
340. Cohen, E.R.; Peña, S.; Misztal, C.; Iglesias, T.; Alejandro, M.; Dinh, C.T.; Holt, G.; Thomas, G.R. N95 vs Half-Face Respirator Wear in Surgical Trainees: Physiologic and Psychological Effects of Prolonged Use. *OTO Open* **2021**, *5*, 2473974X211065437, doi:10.1177/2473974X211065437.
341. Demirag, M.E.; Akyil, M.; Karasal, M.; Bayram, S.; Metin, S.K.; Tokgoz, F.A.; Baysungur, V.; Evman, S. Prospective Analysis of the Physiological Changes Caused by Prolonged Use of N95-Type Masks. *Ann Thorac Med* **2023**, *18*, 86–89, doi:10.4103/atm.atm_429_22.
342. Gaikwad, R.P.; Banodkar, A.B.; Nandgaonkar, V.P. Respiratory Consequences of N95 Mask during Covid-19 Pandemic- An Observational Study. *International Journal of Health Sciences and Research* **2021**, *11*, 55, doi:10.52403/ijhsr.20210407.
343. Hussain, S.A.; Mahmood, N.M.A.; Mahmood, T.M.A.; Salih, N.A.M.; Abdulrahman, Z.S. Adverse Effects Associated with the Use of N95 Mask among Health-Care Workers at the COVID-19 Care Units: A Cross-Sectional Study in Sulaimani City, Iraq. *J Educ Health Promot* **2022**, *11*, 198, doi:10.4103/jehp.jehp_1001_21.
344. Kao, T.-W.; Huang, K.-C.; Huang, Y.-L.; Tsai, T.-J.; Hsieh, B.-S.; Wu, M.-S. The Physiological Impact of Wearing an N95 Mask during Hemodialysis as a Precaution against SARS in Patients with End-Stage Renal Disease. *J Formos Med Assoc* **2004**, *103*, 624–628.
345. Kim, J.-H.; Wu, T.; Powell, J.B.; Roberge, R.J. Physiologic and Fit Factor Profiles of N95 and P100 Filtering Facepiece Respirators for Use in Hot, Humid Environments. *Am J Infect Control* **2016**, *44*, 194–198, doi:10.1016/j.ajic.2015.08.027.
346. Kumar, P.; Nath, K.; Prasad, A.; Tiwari, L.K.; Chowdhry, B.K.; Sinha, A.K.; Chaudhary, N. Effects of the Use of N95 Masks on the Vital Signs of Healthy Healthcare Workers During the COVID-19 Pandemic: A Hospital-Based Cross-Sectional Study. *Cureus* **2023**, *15*, e40622, doi:10.7759/cureus.40622.
347. Kurt, A.; Altındal, E.U. Choroidal Thickness Changes in Healthcare Professionals Wearing Surgical Masks or FFP2 Masks: Pilot Study. *Photodiagnosis Photodyn Ther* **2022**, *37*, 102608, doi:10.1016/j.pdpdt.2021.102608.
348. Manerkar, H.U.; Nagarsekar, A.; Gaunkar, R.B.; Dhupar, V.; Khorate, M. Assessment of Hypoxia and Physiological Stress Evinced by Usage of N95 Masks among Frontline Dental Healthcare Workers in a Humid Western Coastal Region of India-A Repeated Measure Observational Study. *Indian J Occup Environ Med* **2021**, *25*, 209–214, doi:10.4103/ijoem.ijoem_446_20.
349. Porcari, J.P.; Probst, L.; Forrester, K.; Doberstein, S.; Foster, C.; Cress, M.L.; Schmidt, K. Effect of Wearing the Elevation Training Mask on Aerobic Capacity, Lung Function, and Hematological Variables. *J Sports Sci Med* **2016**, *15*, 379–386.
350. Saccomanno, S.; Manenti, R.J.; Giancaspro, S.; Paskay, L.C.; Katzenmaier, C.S.; Mastrapasqua, R.F.; Quinzi, V. Evaluation of the Effects on SpO2 of N95 Mask (FFP2) on Dental Health Care Providers: A Cross-Sectional Observational Study. *BMC Health Services Research* **2022**, *22*, 248, doi:10.1186/s12913-022-07648-5.
351. Scarano, A.; Inchingolo, F.; Rapone, B.; Festa, F.; Rexhep Tari, S.; Lorusso, F. Protective Face Masks: Effect on the Oxygenation and Heart Rate Status of Oral Surgeons during Surgery. *International Journal of Environmental Research and Public Health* **2021**, *18*, 2363, doi:10.3390/ijerph18052363.
352. Sinha, D.; Bhandary, S.K.; Aroor, R.; Reddy, R.; Alva, S. Effect of N-95 Mask on Oxygen Saturation Level in Health Care Workers and Their Experience While Using N-95 Mask. *Journal of Health and Allied Sciences NU* **2022**, *12*, 274–276, doi:10.1055/s-0041-1740329.

353. Toprak, E.; Bulut, A.N. The Effect of Mask Use on Maternal Oxygen Saturation in Term Pregnancies during the COVID-19 Process. *Journal of Perinatal Medicine* **2021**, *49*, 148–152, doi:10.1515/jpm-2020-0422.
354. Wojtasz, I.; Jaracz, K.; Sobczyński, P.; Drużdż, A.; Dyk, D.; Kaźmierski, R. The Impact of FFP3 Respirators on the Blood Saturation. *Sci Rep* **2022**, *12*, 1335, doi:10.1038/s41598-022-05319-3.
355. Wulandari, S.M.; Rahmawati, A.; Rohmah, U.N.; Pertiwi, H.; Zakiyah The Dilemma of Using N95 Masks for Health Workers.; Atlantis Press, June 14 2023; pp. 119–127.
356. Yang, S.; Fang, C.; Liu, X.; Liu, Y.; Huang, S.; Wang, R.; Qi, F. Surgical Masks Affect the Peripheral Oxygen Saturation and Respiratory Rate of Anesthesiologists. *Frontiers in Medicine* **2022**, *9*.
357. Bao, R.; Ning, G.; Sun, Y.; Pan, S.; Wang, W. Evaluation of Mask-Induced Cardiopulmonary Stress. *JAMA Netw Open* **2023**, *6*, e2317023, doi:10.1001/jamanetworkopen.2023.17023.
358. Morgan, B.J.; Crabtree, D.C.; Palta, M.; Skatrud, J.B. Combined Hypoxia and Hypercapnia Evokes Long-Lasting Sympathetic Activation in Humans. *Journal of Applied Physiology* **1995**, *79*, 205–213, doi:10.1152/jappl.1995.79.1.205.
359. Choudhury, A.; Singh, M.; Khurana, D.K.; Mustafi, S.M.; Ganapathy, U.; Kumar, A.; Sharma, S. Physiological Effects of N95 FFP and PPE in Healthcare Workers in COVID Intensive Care Unit: A Prospective Cohort Study. *Indian J Crit Care Med* **2020**, *24*, 1169–1173, doi:10.5005/jp-journals-10071-23671.
360. Pongvaramitchai, R.; Nakornchai, S.; Apipan, B. Effect of an N95 Respirator and a Surgical Mask-Covered N95 on the Cardiovascular Responses of Dentists Treating Paediatric Patients: A Crossover Clinical Trial. *Int J Paediatr Dent* **2023**, *33*, 315–324, doi:10.1111/ipd.13068.
361. Shehade, H.; Acolty, V.; Moser, M.; Oldenhove, G. Cutting Edge: Hypoxia-Inducible Factor 1 Negatively Regulates Th1 Function. *The Journal of Immunology* **2015**, doi:10.4049/jimmunol.1402552.
362. Westendorf, A.M.; Skibbe, K.; Adamczyk, A.; Buer, J.; Geffers, R.; Hansen, W.; Pastille, E.; Jendrossek, V. Hypoxia Enhances Immunosuppression by Inhibiting CD4+ Effector T Cell Function and Promoting Treg Activity. *CPB* **2017**, *41*, 1271–1284, doi:10.1159/000464429.
363. G. M. Al-Allaff, R.; M. Y. Al-Taee, S.; T. D. Baker, S. Some Immunological Impacts of Face Mask Usage During the COVID-19 Pandemic. *Pakistan Journal of Biological Sciences* **2021**, *24*, 920–927, doi:10.3923/pjbs.2021.920.927.
364. Sceneay, J.; Parker, B.S.; Smyth, M.J.; Möller, A. Hypoxia-Driven Immunosuppression Contributes to the Pre-Metastatic Niche. *OncoImmunology* **2013**, *2*, e22355, doi:10.4161/onci.22355.
365. McKenna, V.S.; Kendall, C.L.; Patel, T.H.; Howell, R.J.; Gustin, R.L. Impact of Face Masks on Speech Acoustics and Vocal Effort in Healthcare Professionals. *The Laryngoscope* **2022**, *132*, 391–397, doi:10.1002/lary.29763.
366. Sönnichsen, R.; Llorach Tó, G.; Hochmuth, S.; Hohmann, V.; Radeloff, A. How Face Masks Interfere With Speech Understanding of Normal-Hearing Individuals: Vision Makes the Difference. *Otol Neurotol* **2022**, *43*, 282–288, doi:10.1097/MAO.0000000000003458.
367. Truong, T.L.; Beck, S.D.; Weber, A. The Impact of Face Masks on the Recall of Spoken Sentences. *J Acoust Soc Am* **2021**, *149*, 142–144, doi:10.1121/10.0002951.
368. Zarei, N.; Negarandeh, R.; Neshat, H. Communication Challenges Caused by Wearing Masks and Strategies Used by Pediatric Nurses during the COVID-19 Pandemic: A Qualitative Study. *Journal of Pediatric Nursing: Nursing Care of Children and Families* **2024**, *0*, doi:10.1016/j.pedn.2024.03.020.
369. Carbon, C.-C.; Held, M.J.; Schütz, A. Reading Emotions in Faces With and Without Masks Is Relatively Independent of Extended Exposure and Individual Difference Variables. *Frontiers in Psychology* **2022**, *13*.

370. Hua, D.; Xu, Y.; Heiduschka, P.; Zhang, W.; Zhang, X.; Zeng, X.; Zhu, X.; He, T.; Zheng, H.; Xiao, X.; et al. Retina Vascular Perfusion Dynamics During Exercise With and Without Face Masks in Healthy Young Adults: An OCT Angiography Study. *Translational Vision Science & Technology* **2021**, *10*, 23, doi:10.1167/tvst.10.3.23.
371. Janicijevic, D.; Redondo, B.; Jiménez, R.; Lacorzana, J.; García-Ramos, A.; Vera, J. Intraocular Pressure Responses to Walking with Surgical and FFP2/N95 Face Masks in Primary Open-Angle Glaucoma Patients. *Graefes Arch Clin Exp Ophthalmol* **2021**, *259*, 2373–2378, doi:10.1007/s00417-021-05159-3.
372. Jaya, I.P.P.; Astrawan, I.P.; Negara, N.L.G.A.M.; Empuaji, M.G.D. The Effect of Wearing A Mask on Oxygen Saturation and Pulse Rate During Climbing Stair in Public Areas. *Indonesian Journal of Global Health Research* **2024**, *6*, 631–644, doi:10.37287/ijghr.v6i2.2975.
373. Wong, A.Y.-Y.; Ling, S.K.-K.; Louie, L.H.-T.; Law, G.Y.-K.; So, R.C.-H.; Lee, D.C.-W.; Yau, F.C.-F.; Yung, P.S.-H. Impact of the COVID-19 Pandemic on Sports and Exercise. *Asia-Pacific Journal of Sports Medicine, Arthroscopy, Rehabilitation and Technology* **2020**, *22*, 39–44, doi:10.1016/j.asmart.2020.07.006.
374. Shenal, B.V.; Radonovich, L.J.; Cheng, J.; Hodgson, M.; Bender, B.S. Discomfort and Exertion Associated with Prolonged Wear of Respiratory Protection in a Health Care Setting. *J Occup Environ Hyg* **2011**, *9*, 59–64, doi:10.1080/15459624.2012.635133.
375. Balestracci, B.; La Regina, M.; Di Sessa, D.; Mucci, N.; Angelone, F.D.; D'Ecclesia, A.; Fineschi, V.; Di Tommaso, M.; Corbetta, L.; Lachman, P.; et al. Patient Safety Implications of Wearing a Face Mask for Prevention in the Era of COVID-19 Pandemic: A Systematic Review and Consensus Recommendations. *Intern Emerg Med* **2023**, *18*, 275–296, doi:10.1007/s11739-022-03083-w.
376. Barbeito-Caamaño, C.; Bouzas-Mosquera, A.; Peteiro, J.; López-Vázquez, D.; Quintas-Guzmán, M.; Varela-Cancelo, A.; Martínez-Ruiz, D.; Yañez-Wonenburger, J.C.; Piñeiro-Portela, M.; Vázquez-Rodríguez, J.M. Exercise Testing in COVID-19 Era: Clinical Profile, Results and Feasibility Wearing a Facemask. *European Journal of Clinical Investigation* **2021**, *51*, e13509, doi:10.1111/eci.13509.
377. Dacha, S.; Chuatrakoon, B.; Sornkaew, K.; Sutthakhun, K.; Weeranorapanich, P. Effects of Wearing Different Facial Masks on Respiratory Symptoms, Oxygen Saturation, and Functional Capacity during Six-Minute Walk Test in Healthy Subjects. *Can J Respir Ther* **2022**, *58*, 85–90, doi:10.29390/cjrt-2022-014.
378. Gelmez Taş, B.; Zerenozturk, G.; Demir, I.; Acar, I.; Basanmay, M. The Complaints of Health Workers After Mask Usage. *Eurasian Journal of Family Medicine* **2023**, *12*, 125–130, doi:10.33880/ejfm.2023120303.
379. Gyapong, F.; Debrah, E.; Oforiwaa, M.; Isawumi, A.; Mosi, L. Challenges and Adverse Effects of Wearing Face Masks in the COVID-19 Era. *Challenges* **2022**, *13*, 67, doi:10.3390/challe13020067.
380. Hua, W.; Zuo, Y.; Wan, R.; Xiong, L.; Tang, J.; Zou, L.; Shu, X.; Li, L. Short-Term Skin Reactions Following Use of N95 Respirators and Medical Masks. *Contact Dermatitis* **2020**, *83*, 115–121, doi:10.1111/cod.13601.
381. Kizmaz, E.; Unver, F.; Telli Atalay, O. The Effects of Face Masks on Cardiopulmonary Capacity in Healthy Young Individuals. *J Sports Med Phys Fitness* **2022**, *62*, 1301–1305, doi:10.23736/S0022-4707.21.12880-4.
382. Lässing, J.; Falz, R.; Pökel, C.; Fikenzer, S.; Laufs, U.; Schulze, A.; Hölldobler, N.; Rüdrich, P.; Busse, M. Effects of Surgical Face Masks on Cardiopulmonary Parameters during Steady State Exercise. *Sci Rep* **2020**, *10*, 22363, doi:10.1038/s41598-020-78643-1.
383. Mahmud, R.; Joy, K.M.N.I.; Rassel, M.A.; Monayem, F.B.; Datta, P.K.; Hossain, M.S.; Hoque, M.M.; Habib, S.M.H.R.; Munna, N.H.; Ahmed, M.; et al. Health Hazards Related to Using Masks and/or Personal Protective Equipment among Physicians Working in Public

Hospitals in Dhaka: A Cross-Sectional Study. *PLOS ONE* **2022**, *17*, e0274169, doi:10.1371/journal.pone.0274169.
384. Matusiak, Ł.; Szepietowska, M.; Krajewski, P.; Białynicki-Birula, R.; Szepietowski, J.C. Inconveniences Due to the Use of Face Masks during the COVID-19 Pandemic: A Survey Study of 876 Young People. *Dermatologic Therapy* **2020**, *33*, e13567, doi:10.1111/dth.13567.
385. Micheletti Cremasco, M.; Vigoroso, L.; Solinas, C.; Caffaro, F. Discomfort in Use and Physical Disturbance of FFP2 Masks in a Group of Italian Doctors, Nurses and Nursing Aides during the COVID-19 Pandemic. *Safety* **2023**, *9*, 40, doi:10.3390/safety9020040.
386. Moumneh, A.; Kofoed, P.-E.L.; Vahlkvist, S.V. Face Mask Use during the COVID-19 Pandemic Was Associated with Breathing Difficulties in Adolescent Patients with Asthma. *Acta Paediatr* **2023**, *112*, 1740–1746, doi:10.1111/apa.16852.
387. Person, E.; Lemercier, C.; Royer, A.; Reychler, G. Effet du port d'un masque de soins lors d'un test de marche de six minutes chez des sujets sains. *Revue des Maladies Respiratoires* **2018**, *35*, 264–268, doi:10.1016/j.rmr.2017.01.010.
388. Prousa, D. Studie zu psychischen und psychovegetativen Beschwerden mit den aktuellen Mund-Nasenschutz-Verordnungen. **2020**, 1–128, doi:10.23668/psycharchives.3135.
389. Purushothaman, P.K.; Priyangha, E.; Vaidhyswaran, R. Effects of Prolonged Use of Facemask on Healthcare Workers in Tertiary Care Hospital During COVID-19 Pandemic. *Indian Journal of Otolaryngology and Head & Neck Surgery* **2021**, *73*, 59, doi:10.1007/s12070-020-02124-0.
390. Scarano, A.; Inchingolo, F.; Lorusso, F. Facial Skin Temperature and Discomfort When Wearing Protective Face Masks: Thermal Infrared Imaging Evaluation and Hands Moving the Mask. *Int J Environ Res Public Health* **2020**, *17*, doi:10.3390/ijerph17134624.
391. Chaudhary, S.K.; Prasad, N.; Verma, C.; Jaiswal, S.; Jaiswal, A. Headache Associated with PPE During COVID-19 Pandemic in Health Care Workers. *Acta Neurol Taiwan* **2023**, *32(2)*, 57–64.
392. Chmielewska, B.; Barratt, I.; Townsend, R.; Kalafat, E.; Meulen, J. van der; Gurol-Urganci, I.; O'Brien, P.; Morris, E.; Draycott, T.; Thangaratinam, S.; et al. Effects of the COVID-19 Pandemic on Maternal and Perinatal Outcomes: A Systematic Review and Meta-Analysis. *The Lancet Global Health* **2021**, *0*, doi:10.1016/S2214-109X(21)00079-6.
393. Jacobs, J.L.; Ohde, S.; Takahashi, O.; Tokuda, Y.; Omata, F.; Fukui, T. Use of Surgical Face Masks to Reduce the Incidence of the Common Cold among Health Care Workers in Japan: A Randomized Controlled Trial. *Am J Infect Control* **2009**, *37*, 417–419, doi:10.1016/j.ajic.2008.11.002.
394. Marques-Sule, E.; Espí-López, G.V.; Monzani, L.; Suso-Martí, L.; Rel, M.C.; Arnal-Gómez, A. How Does the Continued Use of the Mask Affect the Craniofacial Region? A Cross-Sectional Study. *Brain and Behavior* **2023**, *13*, e3077, doi:10.1002/brb3.3077.
395. Ong, J.J.Y.; Bharatendu, C.; Goh, Y.; Tang, J.Z.Y.; Sooi, K.W.X.; Tan, Y.L.; Tan, B.Y.Q.; Teoh, H.-L.; Ong, S.T.; Allen, D.M.; et al. Headaches Associated With Personal Protective Equipment - A Cross-Sectional Study Among Frontline Healthcare Workers During COVID-19. *Headache* **2020**, *60*, 864–877, doi:10.1111/head.13811.
396. Polivka, B.J.; Eldeirawi, K.; Huntington-Moskos, L.; Nyenhuis, S.M. Mask Use Experiences, COVID-19, and Adults with Asthma: A Mixed-Methods Approach. *The Journal of Allergy and Clinical Immunology: In Practice* **2022**, *10*, 116–123, doi:10.1016/j.jaip.2021.10.071.
397. Ramirez-Moreno, J.M.; Ceberino, D.; Plata, A.G.; Rebollo, B.; Sedas, P.M.; Hariramani, R.; Roa, A.M.; Constantino, A.B. Mask-Associated 'de Novo' Headache in Healthcare Workers during the COVID-19 Pandemic. *Occup Environ Med* **2021**, *78*, 548–554, doi:10.1136/oemed-2020-106956.
398. van Kampen, V.; Marek, E.-M.; Sucker, K.; Jettkant, B.; Kendzia, B.; Strauß, B.; Ulbrich, M.; Deckert, A.; Berresheim, H.; Eisenhawer, C.; et al. Influence of Face Masks on the

Subjective Impairment at Different Physical Workloads. *Sci Rep* **2023**, *13*, 8133, doi:10.1038/s41598-023-34319-0.
399. Waniganayake, Y.C.; Nadeeshani, H.K.D.K.; Buddhadasa, P.S.L.S.; Iresha, G.K.; Jayatilleke, S.K. Difficulties Encountered by Health Care Workers with the Use of N95 Disposable Masks and Elastomeric Respirators during COVID 19 Pandemic. **2021**, *11*, 18, doi:10.4038/sljid.v11i1.8344.
400. Yusof, M.Z.; Mukhtar, N.I. Workers’ Perceptions on the Tolerability of Their Respirators in Malaysia. *Journal of Health Research* **2023**, *37*, doi:10.56808/2586-940X.1025.
401. Athar, M.H.; Zubair, U.B.; Ujala, Z. Biohazards of Prolonged N95 Use: Effects on Arterial Blood Gases, Peak Expiratory Flow Rate and General Well-Being of Healthcare Professionals during COVID-19 Pandemic. *JCPSP, Journal of the College of Physicians and Surgeons Pakistan* **2020**, S134–S135.
402. Hai, C.H.; Hua, K.K.; Wen, C.J.; Wen, C.C.; Ning, K.R.; Wah, Y.C. Comparing Effect of Donning Different Types of Face Masks on Oxygen Saturation, Pulse Rate and Comforts among Healthy Wearers. *Journal of Positive School Psychology* **2022**, *6*, 4058–4068.
403. İpek, S.; Yurttutan, S.; Güllü, U.U.; Dalkıran, T.; Acıpayam, C.; Doğaner, A. Is N95 Face Mask Linked to Dizziness and Headache? *Int Arch Occup Environ Health* **2021**, *94*, 1627–1636, doi:10.1007/s00420-021-01665-3.
404. Vidua, R.K.; Chouksey, V.K.; Bhargava, D.C.; Kumar, J. Problems Arising from PPE When Worn for Long Periods. *Med Leg J* **2020**, *88*, 47–49, doi:10.1177/0025817220935880.
405. Zhou, Z.; Dong, L. Experimental Investigation of the Effect of Surgical Masks on Outdoor Thermal Comfort in Xiamen, China. *Building and Environment* **2023**, *229*, 109893, doi:10.1016/j.buildenv.2022.109893.
406. Wong, C.K.M.; Yip, B.H.K.; Mercer, S.; Griffiths, S.; Kung, K.; Wong, M.C.; Chor, J.; Wong, S.Y. Effect of Facemasks on Empathy and Relational Continuity: A Randomised Controlled Trial in Primary Care. *BMC Family Practice* **2013**, *14*, 200, doi:10.1186/1471-2296-14-200.
407. Anczyk, S.; Stępień, M.; Raczyński, M.; Anczyk, A.; Woźniakowska, M.; Miziołek, B.; Polak, K.; Bergler-Czop, B. The Impact of Face Masks on Acne-prone Skin in Polish Young Adults during COVID-19 Pandemic. *Dermatol Ther* **2022**, *35*, e15922, doi:10.1111/dth.15922.
408. Bakhsh, R. a; Saddeeg, S.Y.; Basaqr, K.M.; Alshammrani, B.M.; Zimmo, B.S. Prevalence and Associated Factors of Mask-Induced Acne (Maskne) in the General Population of Jeddah During the COVID-19 Pandemic. *Cureus* **2022**, *14*, e26394, doi:10.7759/cureus.26394.
409. Berjawi, A.; Salameh, P.; Fadel, N.; El Khoury, J.R. Mask-Acne Prevalence and Risk Factors during the COVID-19 Pandemic: A Cross-Sectional Single Institution Study. *Dermatologic Therapy* **2023**, *2023*, e9470636, doi:10.1155/2023/9470636.
410. Cheng, Y.-F.; Zhao, H.; Li, J.; Lipa, K.E.; Xie, H.-F.; Wang, B.; Huang, Y.-X. Factors Aggravating Acne Vulgaris during the COVID-19 Pandemic in China: A Web-Based Cross-Sectional Survey. *Eur Rev Med Pharmacol Sci* **2022**, *26*, 7305–7312, doi:10.26355/eurrev_202210_29925.
411. Dani, A.; Eseonu, A.; Bibee, K. Risk Factors for the Development of Acne in Healthcare Workers during the COVID-19 Pandemic. *Arch Dermatol Res* **2023**, *315*, 1067–1070, doi:10.1007/s00403-022-02434-z.
412. Falodun, O.; Medugu, N.; Sabir, L.; Jibril, I.; Oyakhire, N.; Adekeye, A. An Epidemiological Study on Face Masks and Acne in a Nigerian Population. *PLOS ONE* **2022**, *17*, e0268224, doi:10.1371/journal.pone.0268224.
413. Foo, C.C.I.; Goon, A.T.J.; Leow, Y.; Goh, C. Adverse Skin Reactions to Personal Protective Equipment against Severe Acute Respiratory Syndrome – a Descriptive Study in Singapore. *Contact Dermatitis* **2006**, *55*, 291–294, doi:10.1111/j.1600-0536.2006.00953.x.
414. Fourie, A.; Muvhali, M.; Carman, H.; Singh, T. Occupational Skin Disease Associated with Personal Protective Equipment: A Case Series. *Current Allergy & Clinical Immunology* **2022**,

35, 223–231, doi:10.10520/ejc-caci-v35-n4-a6.
415. Kumar Shubhanshu; Singh, A. Prolonged Use of N95 Mask a Boon or Bane to Healthcare Workers During Covid–19 Pandemic. *Indian J Otolaryngol Head Neck Surg* **2022**, *74*, 2853–2856, doi:10.1007/s12070-021-02396-0.
416. Lan, J.; Song, Z.; Miao, X.; Li, H.; Li, Y.; Dong, L.; Yang, J.; An, X.; Zhang, Y.; Yang, L.; et al. Skin Damage among Health Care Workers Managing Coronavirus Disease-2019. *J Am Acad Dermatol* **2020**, *82*, 1215–1216, doi:10.1016/j.jaad.2020.03.014.
417. McKenna, K.; Bouchoucha, S.; Redley, B.; Hutchinson, A. Australian Health Care Workers Experience of PPE Related Side-Effects. A Cross-Sectional Survey. *Front. Public Health* **2024**, *12*, doi:10.3389/fpubh.2024.1325376.
418. Nasir, N.A.B.M.; Sarkurunatan, A.A.; Muhammaddun, M.Z.B.Z.; Yu, L.X.; Manan, N.A. The Prevalence of Adverse Skin Reactions Associated with Face Mask Wearing among Students in Malaysia. *Asian Journal of Medicine and Health Sciences* **2023**, *6*, 122–133.
419. Perera, M.H.; Joshi, M.; Govindan, A.K.; Edpuganti, S.; Korrapati, N.H.; Kiladze, N. Impact of Mask Wear on the Skin of Clinical Year Medical Students during the COVID-19 Pandemic: A Cross-Sectional Study. *CSDM* **2022**, *2*, doi:10.25259/CSDM_100_2022.
420. Sliwakowska, K.; Radwanska, J.; Czuwara, J. The Impact of Masks on the Condition of Facial Skin in the Time of the COVID-19 Pandemic. *Our Dermatology Online* **2022**, *13(e):e55*, 1–8, doi:10.7241/ourd.2022e.55.
421. Techasatian, L.; Lebsing, S.; Uppala, R.; Thaowandee, W.; Chaiyarit, J.; Supakunpinyo, C.; Panombualert, S.; Mairiang, D.; Saengnipanthkul, S.; Wichajarn, K.; et al. The Effects of the Face Mask on the Skin Underneath: A Prospective Survey During the COVID-19 Pandemic. *J Prim Care Community Health* **2020**, *11*, 2150132720966167, doi:10.1177/2150132720966167.
422. He, W.; Liu, Y.; Zou, Z.; Cheng, C.; Wang, W.; Huang, Z.; Wu, G.; Zhu, W.; Sun, H. Effects of Prolonged Face Mask Use among Patients with Hypertension or Diabetes during the COVID-19 Pandemic. *J Int Med Res* **2024**, *52*, 03000605241232946, doi:10.1177/03000605241232946.
423. Park, S.-R.; Han, J.; Yeon, Y.M.; Kang, N.Y.; Kim, E. Effect of Face Mask on Skin Characteristics Changes during the COVID-19 Pandemic. *Skin Res Technol* **2021**, *27*, 554–559, doi:10.1111/srt.12983.
424. Naylor, G.; Burke, L.A.; Holman, J.A. Covid-19 Lockdown Affects Hearing Disability and Handicap in Diverse Ways: A Rapid Online Survey Study. *Ear Hear* **2020**, *41*, 1442–1449, doi:10.1097/aud.0000000000000948.
425. Thomas, F.; Allen, C.; Butts, W.; Rhoades, C.; Brandon, C.; Handrahan, D.L. Does Wearing a Surgical Facemask or N95-Respirator Impair Radio Communication? *Air Med J* **2011**, *30*, 97–102, doi:10.1016/j.amj.2010.12.007.
426. Heider, C.A.; Álvarez, M.L.; Fuentes-López, E.; González, C.A.; León, N.I.; Verástegui, D.C.; Badía, P.I.; Napolitano, C.A. Prevalence of Voice Disorders in Healthcare Workers in the Universal Masking COVID-19 Era. *The Laryngoscope* **2020**, *n/a*, doi:10.1002/lary.29172.
427. Forgie, S.E.; Reitsma, J.; Spady, D.; Wright, B.; Stobart, K. The “Fear Factor” for Surgical Masks and Face Shields, as Perceived by Children and Their Parents. *Pediatrics* **2009**, *124*, e777-781, doi:10.1542/peds.2008-3709.
428. Zhang, Y.; Yang, Q.; Ma, Q.; Wu, Y.; Wang, Y.; Chen, C.; Yao, Y.; Feng, Z.; Yuan, X.; Shi, S.; et al. Effect of Surgical Masks and N95 Respirators on Anxiety. *NDT* **2024**, *20*, 551–559, doi:10.2147/NDT.S447428.
429. Hatanaka, N.; Xu, B.; Yasugi, M.; Morino, H.; Tagishi, H.; Miura, T.; Shibata, T.; Yamasaki, S. Chlorine Dioxide Is a More Potent Antiviral Agent against SARS-CoV-2 than Sodium Hypochlorite. *J Hosp Infect* **2021**, *118*, 20–26, doi:10.1016/j.jhin.2021.09.006.
430. U.S. Environmental Protection Agency (US EPA). Integrated Risk Information System (IRIS): Toxicological Review of Chlorine Dioxide and Chlorite (CAS Nos. 10049-04-4 Last

Revised — 10/12/2000. *National Service Center for Environmental Publications, Chemical Assessment Summary, Human health assessment information* **2000**, 1–24.
431. Dourson, M.; Charnley, G.; Scheuplein, R. Differential Sensitivity of Children and Adults to Chemical Toxicity: II. Risk and Regulation. *Regulatory Toxicology and Pharmacology* **2002**, *35*, 448–467, doi:10.1006/rtph.2002.1559.
432. OECD PISA Results: Maths and Reading Skills in “Unprecedented Drop”. Here’s Why That Matters Available online: https://www.weforum.org/agenda/2023/12/oecd-pisa-results-maths-reading-skills-education/ (accessed on 29 August 2024).
433. Kampf, G. Effect of Face Masking on Transmission of SARS-CoV-2. In *The COVID-19 Aftermath: Volume II: Lessons Learned*; Rezaei, N., Ed.; Springer Nature Switzerland: Cham, 2024; pp. 175–199 ISBN 978-3-031-61943-4.
434. Tenenbaum, T.; Doenhardt, M.; Diffloth, N.; Berner, R.; Armann, J.P. High Burden of RSV Hospitalizations in Germany 2021–2022. *Infection* **2022**, *50*, 1587–1590, doi:10.1007/s15010-022-01889-6.
435. Ma, K.C. Increase in Acute Respiratory Illnesses Among Children and Adolescents Associated with Rhinoviruses and Enteroviruses, Including Enterovirus D68 — United States, July–September 2022. *MMWR Morb Mortal Wkly Rep* **2022**, *71*, doi:10.15585/mmwr.mm7140e1.
436. Gier, B. de; Marchal, N.; Beer-Schuurman, I. de; Wierik, M. te; Hooiveld, M.; Group, I.-A.S.; Group, G.S.; Melker, H.E. de; Sorge, N.M. van Increase in Invasive Group A Streptococcal (Streptococcus Pyogenes) Infections (iGAS) in Young Children in the Netherlands, 2022. *Eurosurveillance* **2023**, *28*, 2200941, doi:10.2807/1560-7917.ES.2023.28.1.2200941.
437. Ladhani, S.N.; Guy, R.; Bhopal, S.S.; Brown, C.S.; Lamagni, T.; Sharp, A. Paediatric Group A Streptococcal Disease in England from October to December, 2022. *The Lancet Child & Adolescent Health* **2023**, *7*, e2–e4, doi:10.1016/S2352-4642(22)00374-1.
